# Synthesis of 2‑Phosphorus-Substituted Indoles via Ring Expansion of Benzocyclobutenone Oxime Sulfonates

**DOI:** 10.1021/acs.orglett.5c01778

**Published:** 2025-05-29

**Authors:** Yusuke Kanno, Yumi Yamashita, Akira Sugiyama, Tatsuhiko Kodama, Juri Sakata, Hidetoshi Tokuyama

**Affiliations:** † Graduate School of Pharmaceutical Sciences, 13101 Tohoku University, 6-3 Aoba, Aramaki, Aoba-ku, Sendai 980-8578, Japan; ‡ Department of Clinical Pharmaceutical Sciences, Hoshi University, 2-4-41 Ebara, Shinagawa-Ku, Tokyo 142-8501, Japan; § Research Center for Advanced Science and Technology, The University of Tokyo, 4-6-1 Komaba, Meguro-ku, Tokyo 153-8904, Japan

## Abstract

A method for the synthesis of 2-phosphorus-substituted indoles via ring expansion of benzocyclobutenone oxime sulfonates, which were prepared via the [2 + 2] cycloaddition of benzynes and ketene acetals, and subsequent oximization and sulfonylation was developed. The reaction occurs by addition of the phosphate anion or phosphine oxide anion to the C=N bond of oxime sulfonates, followed by ring expansion to provide 2-phosphorus-substituted indoles. This method was applicable to the synthesis of 2-phosphorus-substituted indoles with a wide variety of substitution patterns on the benzene ring and at the 3-position, as well as to a 2-phosphorus-substituted 4-aza-indole. An indol-2-ylphosphonic acid and a 2-phosphaneylindole were obtained by a transformation of the corresponding products. This protocol was applied to synthesize a duocarmycin SA phosphonate analogue, which exhibited greater cytotoxicity against HeLa S3 and KPL-4 cells than duocarmycin SA.

## Graphical abstract

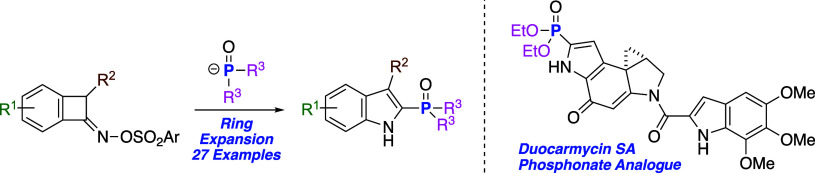

Extensive research has focused on the development of a novel method to synthesize functionalized indoles because these compounds are frequently found in natural products, pharmaceuticals, functional materials, and agrochemicals.^1^ In particular, 2-phosphorus-substituted indoles have recently attracted considerable attention as scaffolds for the design of pharmaceuticals, functional materials, and ligands for transition metal catalysts (Scheme 1).^2–4^

**Scheme 1:**
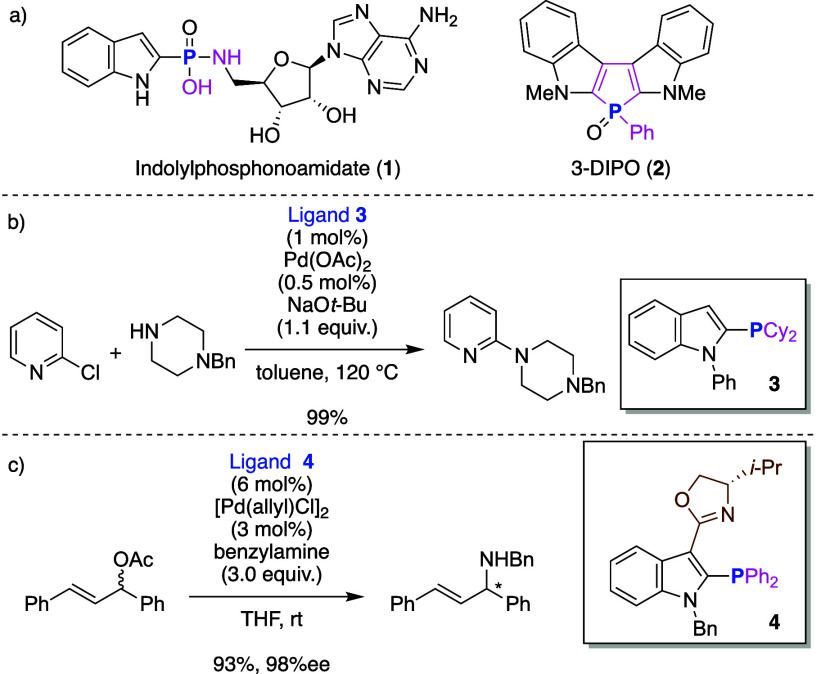
Utilities of 2-Phosphorus-Substituted Indoles

For example, indol-2-ylphosphonamidate **1** inhibits the salicylating enzymes involved in siderophore biosynthesis, which affects the virulence of various bacteria, including those causing plague (Scheme 1a).^2^ Therefore, **1** is a potential lead antimicrobial compound for use against the bacteria causing plague (*Yersinia pestis*) and tuberculosis (*Mycobacterium tuberculosis*). Phosphole heteroacene 3-DIPO (**2**) was developed for use in organic electroluminescence, and its efficiency as an organic light-emitting diode was investigated.^3^ In addition, various indol-2-ylphosphines have been found to be effective ligands for transition metal catalysts.^4^ For example, Beller reported the Pd-catalyzed amination of aryl chloride in the presence of indol-2-ylphosphine ligands **3** (Scheme 1b),^4a^ and Franzén developed Pd-catalyzed asymmetric allylic substitution of allyl acetate by using indol-2-ylphosphine having oxazoline unit (**4**: IndPHOX) (Scheme 1c).^4b^

There are two main strategies to synthesize 2-phosphorus-substituted indoles (Scheme 2).^4–10^ First, a phosphorus functional group can be introduced via reactions involving indole derivatives. For example, the reaction of 2-lithioindoles **6**, which can be generated by either halogen–lithium exchange of 2-haloindoles **5** or direct lithiation of indoles bearing an appropriate protecting group at the N1 position, with phosphorus-containing electrophiles such as ClPPh_2_ affords 2-phosphorus-substituted indoles (Scheme 2a).^6^ However, this reaction, requiring strongly basic conditions, has a narrow substrate scope and poor functional group compatibility. Alternatively, Pd-catalyzed coupling of **5** with phosphite (Scheme 2a)^7^ and oxidative radical addition of a P-centered radical to 2-unsubstituted indoles, which proceed under relatively mild conditions, have been reported (Scheme 2b).^8^ A second strategy to construct 2-phosphorus-substituted indoles involves pyrrole ring formation with simultaneous incorporation of a phosphorus-containing moiety (Scheme 2c).^7,9^ For example, Fischer indole synthesis uses acylphosphonate (**11**) as a counterpart of aryl hydrazine (**10**).^9a^ Bisseret reported Pd-catalyzed tandem C–P coupling/intramolecular cyclization of *O*-(2,2-dibromovinyl)-aniline (**12**),^7^ and Yang described a modified Fukuyama indole synthesis initiated by the generation of a P-centered radical under Ru­(bpy)_3_Cl_2_-catalyzed photoirradiation conditions, followed by addition of the P-centered radical to isocyano-2-styrylbenzenes (**13**) and 5-*exo*-trig cyclization.^9b^ Moreover, Yorimitsu and Oshima synthesized 2-indolyl phosphine oxides (**16**) via Pd-catalyzed annulation of 1-alkynylphosphine oxide (**15**) with 2-iodoaniline (**14**).^9c^

**Scheme 2:**
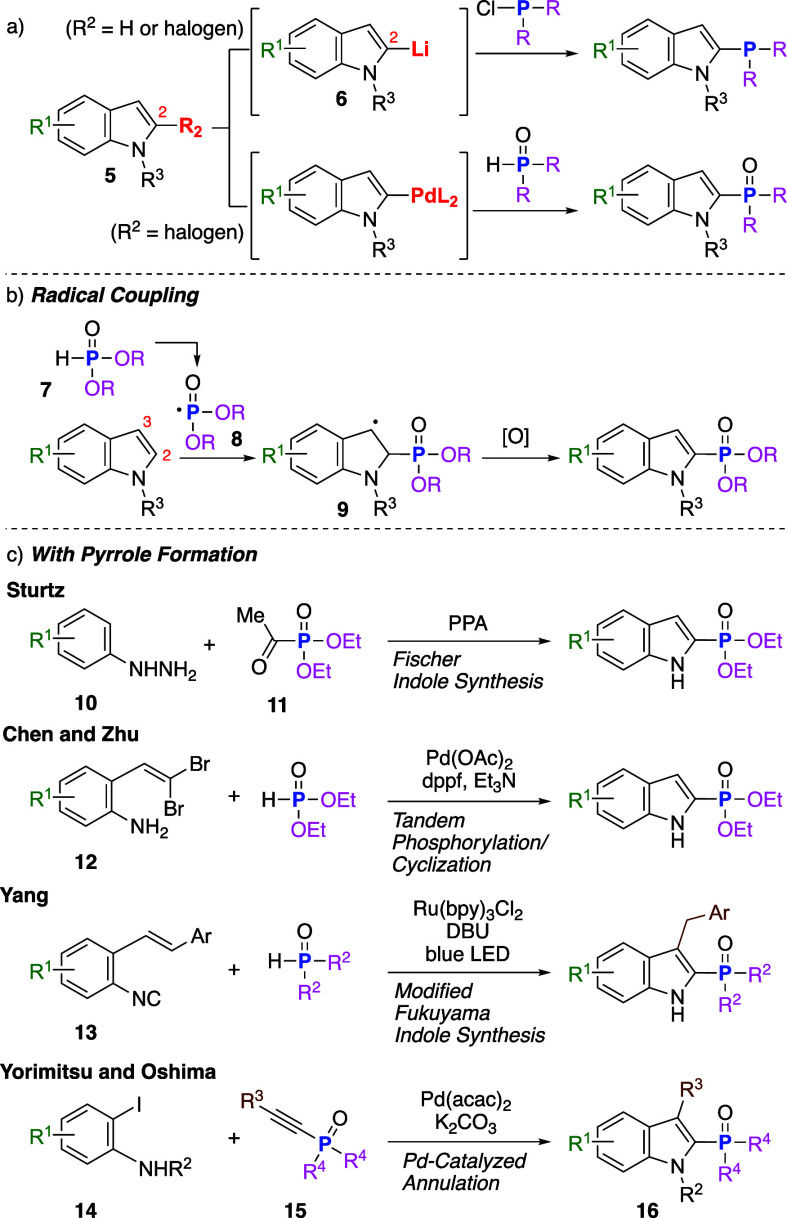
Synthesis of 2-Phosphorus-Substituted Indole

Despite these important advances, there remains room to expand the scope of accessible 2-phosphorus-substituted indoles. In this research, we focused on our indole synthesis via ring expansion of benzocyclobutenone oxime^11^ sulfonates^12^ (Scheme 3). This reaction was initiated by the addition of a nucleophile, such as hydride,^12a^ cyanide,^12a^ and thiolate,^12b^ to oxime sulfonates **17** to generate tetrahedral intermediates **18**, followed by ring expansion via migration of the C­(sp^2^)–C­(sp^3^) bond to the nitrogen atom with concomitant cleavage of the N–O bond, affording 2-substituted indoles **19** (Scheme 3a). We hypothesized that a phosphorus functional group could be introduced at the 2 position of the indole by adding nucleophilic P-centered anion species **20** to **17** (Scheme 3b).^13^ Herein, we report a novel synthetic method for 2-phosphorus-substituted indoles via ring expansion of benzocyclobutenone oxime sulfonates and the synthesis of an unnatural duocarmycin SA phosphonate analog, demonstrating the utility of this method for the synthesis of highly fused 2-phosphorus-substituted indoles.

**Scheme 3:**
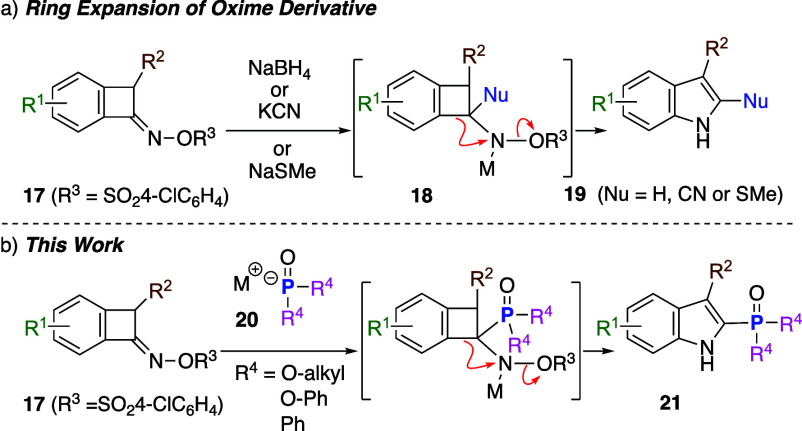
2-Substituted Indole Synthesis by Ring Expansion of Oxime Sulfonate

First, we tested our working hypothesis by using benzocyclobutenone oxime sulfonate **17a** as a test substrate to generate 2-substituted indoles because **17a** provided indole products most efficiently in the previously developed reactions using other nucleophiles (Table 1).^12^ To a 0.1 M THF solution of **17a**, which had been prepared via [2 + 2] cycloaddition of a benzyne derivative with dimethyl ketene acetal,^12^ and diethyl phosphite 7a (2.5 equiv) was added NaH^14^ at room temperature. As expected, 2-phosphorus-substituted indole 21aa was formed in 64% yield (Table 1, entry 1). When NaH was replaced with *t*-BuOK, the reaction afforded **21aa** in a comparable yield (Table 1, entry 2). Then, we selected *t*-BuOK as the base and screened various solvents (Table 1, entries 3–8). Reactions in DMSO and CH_3_CN generated the product in slightly increased yields, whereas the reaction provided **21aa** in lower yields in 1,4-dioxane, cyclopentyl methyl ether (CPME), DMF, or toluene. Decreasing the temperature or increasing the amount of base and phosphite did not improve the yield (Table 1, entries 9 and 10).

**Table 1:**
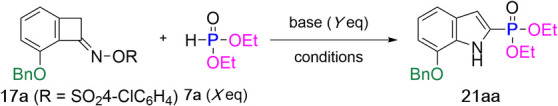
Optimization of the Reaction Conditions

entry	solvent^a^	*X* (equiv)	base	*Y* (equiv)	temp (°C)	time (h)	**21aa** (%)^b^	recovery of **17a** (%)^b^
1	THF	2.5	NaH	2.5	rt	2	64	<23
2	THF	2.5	*t*-BuOK	2.5	rt	2	66	<10
3	1,4-dioxane	2.5	*t-*BuOK	2.5	rt	2	57	<10
4	CPME	2.5	*t*-BuOK	2.5	rt	2	43	<42
5	DMF	2.5	*t*-BuOK	2.5	rt	2	43	<10
6	DMSO	2.5	*t*-BuOK	2.5	rt	2	72	<7
7	toluene	2.5	*t*-BuOK	2.5	rt	2	61	<3
8	CH_3_CN	2.5	*t*-BuOK	2.5	rt	2	70	<3
9	CH_3_CN	2.5	*t*-BuOK	2.5	0	2	71	<9
10	CH_3_CN	5.0	*t*-BuOK	5.0	0	2	69	<6

a Reactions were conducted at a concentration of 0.1 M.

b Isolated yield.

To improve the yield of **21aa**, we further investigated the experimental conditions. When *t*-BuOK was used (Table 1, entries 2–10), unidentified byproducts were generated. To determine whether these byproducts formed via decomposition of **17a** under basic conditions, we conducted a control experiment to evaluate the stability of **17a** in the presence of *t*-BuOK. Treatment of **17a** with *t*-BuOK in CH_3_CN at 0 °C without 7a resulted in complete decomposition of **17a** to the unidentified byproducts. To prevent this decomposition, we investigated the order of addition of the reagents and found that the addition of a solution of phosphonate anion effectively suppressed byproduct generation. Thus, when a freshly prepared CH_3_CN solution of anionic **20a** from **7a** (5.0 equiv) and *t*-BuOK (5.0 equiv) was added to a CH_3_CN solution of **17a** via cannula, the desired reaction proceeded cleanly, generating indole **21aa** in 83% yield without byproduct generation (Scheme 4).^15^

**Scheme 4:**
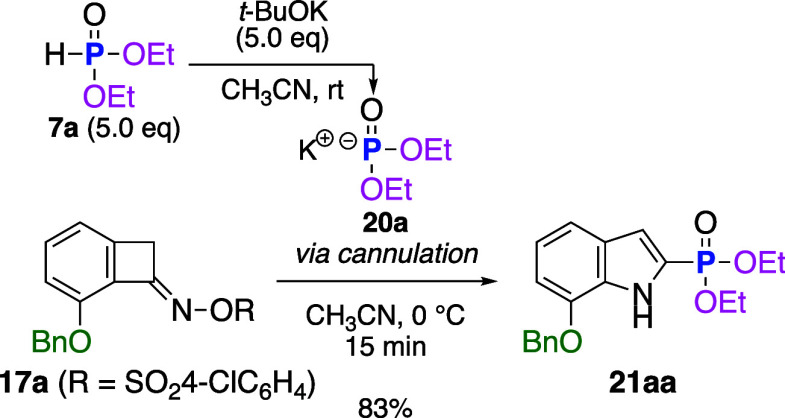
Improved Reaction by Addition of a Stock Solution of the Phosphorous Anion

After establishing the reaction protocol, we next investigated the scope of phosphorus nucleophiles and benzocyclobutenone oxime sulfonates (Table 2). For the phosphonate, methyl ester **7b**, *n*-butyl ester **7c**, *i*-propyl ester **7d**, *t*-butyl ester **7e**, benzyl ester **7f**, and phenyl ester **7g** were suitable for the reaction, and the corresponding 2- phosphorus-substituted indoles were generated in good yields. In addition, an anionic species generated from diphenylphosphine oxide smoothly reacted with **17a** to provide indol-2-ylphosphine oxide **21ah** in good yield. Because anionic species **20d**, **20e**, **20g**, and **20h**, which were generated from *i*-propyl, *t*-butyl, and phenyl esters **7d**, **7e**, and **7g** and diphenylphosphine oxide **7h**, were poorly soluble in CH_3_CN, the reaction was conducted by adding NaH to a CH_3_CN solution of the phosphite or phosphine oxide (**7d–e**, **7g–h**) and **17a**. Next, the generality of benzocyclobutenone oxime sulfonates was examined by using phosphonate anion **20a** generated from **7a**. Substrates **17a–f**,^16^ bearing electron-donating groups, including methyl, phenyl, trimethylsilyl (TMS), morpholino, and *n*-C_12_H_25_S groups, at the 7 position of the benzene ring, reacted to form desired products **21ba–fa** in good to high yields. Conversely, the reaction of substrates **17g–i**,^16^ bearing electron-withdrawing groups, such as CF_3_, Br, and triflate (OTf), afforded **21ga–ia** in low to modest yields. Furthermore, unsubstituted (**21ja**), 6-substituted (**21ma** and **21na**), and 6,7-disubstituted (**21oa**) compounds were obtained in good to high yields, whereas 5-substituted compounds **21ka** and **21la** were generated in lower yields. Notably, 3-substituted 2-phosphorus-substituted indoles could be synthesized by this reaction. Thus, products bearing a methyl group (**21pa**), a *tert*-butyldimethylsilyl (TBS)-protected hydroxyethyl group (**21qa**), and even a sterically hindered *t*-Bu group (**21ra**) were obtained in high yields.

**Table 2:**
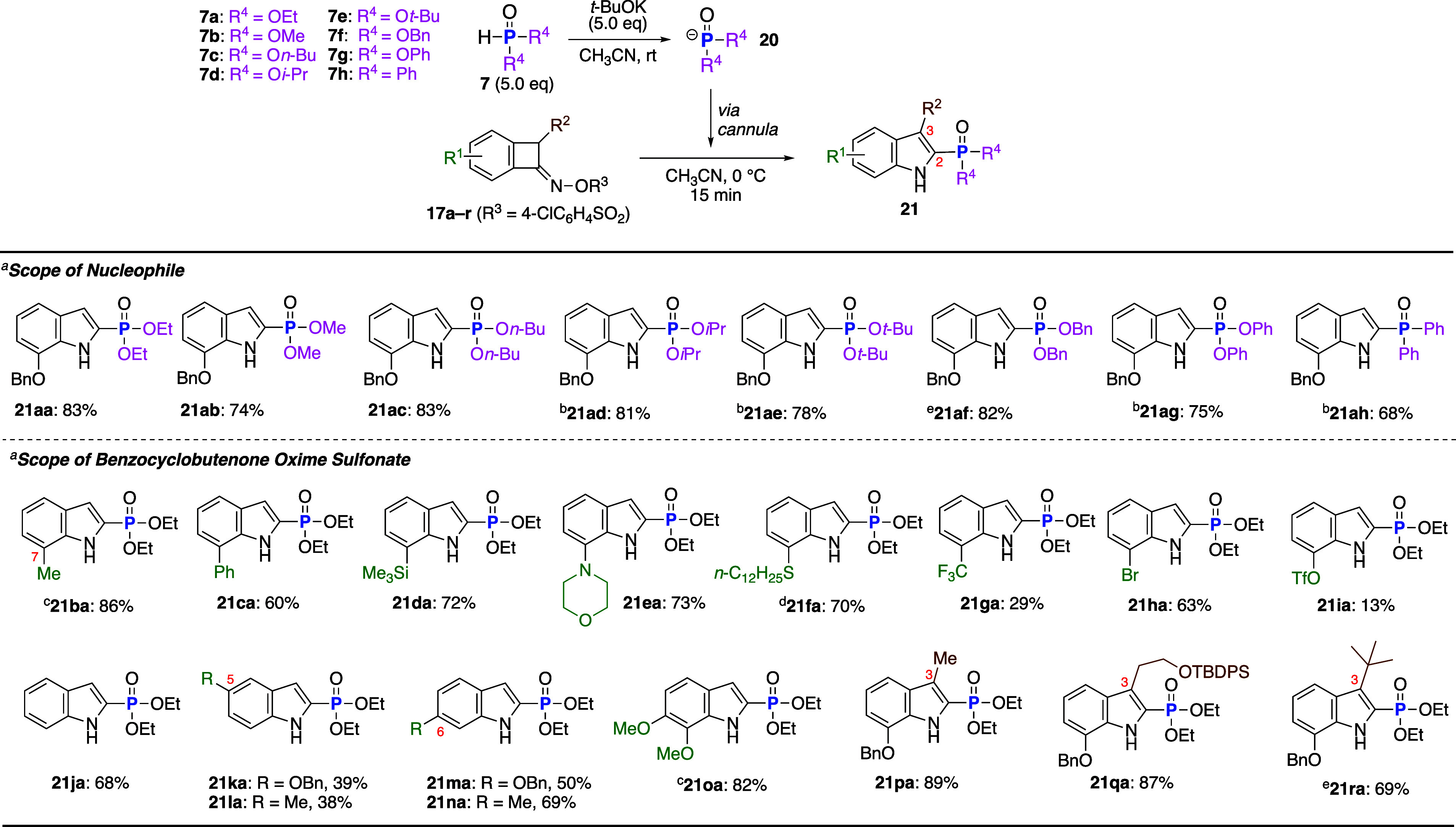
Scope of the Nucleophile and Benzocyclobutenone Oxime Sulfonate

a Isolated yield.

b Since substrates **20d**, **20e**, **20g**, and **20h** have poor solubility in CH_3_CN generating white suspensions, reactions were conducted by adding NaH to a mixture of **7d**, **7e**, **7g**, **7h**,and **17a** in CH_3_CN.

c The reactions were conducted in DMSO.

d The reaction was conducted in DMSO/1,4-dioxane (3:1).

e Additional **20** (2.5 equiv) was required to complete the reaction.

Furthermore, the utility of this reaction was expanded to synthesize a 4-*aza*-indole derivative, indol-2-ylphosphonic acid, and 2-phosphaneylindole (Scheme 5a–5c). Ring expansion of pyridine-fused cyclobutenone oxime sulfonate **17s**,^16^ which was prepared via [2 + 2] cycloaddition of 2,3-pyridyne species with dimethyl ketene acetal, proceeded in the presence of **7a** and *t*-BuOK in CH_3_CN, affording 2-phosphorus-substituted 4-*aza*-indole **21sa** in modest yield (Scheme 5a). Indol-2-ylphosphonic acid **22** was generated by TMSBr-mediated deethylation of phosphate **21aa** (Scheme 5b).^17^ Reduction of indol-2-ylphosphine oxide **21ah** by treatment with either LiAlH_4_ ^18^ or a combination of trichlorosilane and triethylamine afforded 2-phosphaneylindole **23** (Scheme 5c).^19^

**Scheme 5:**
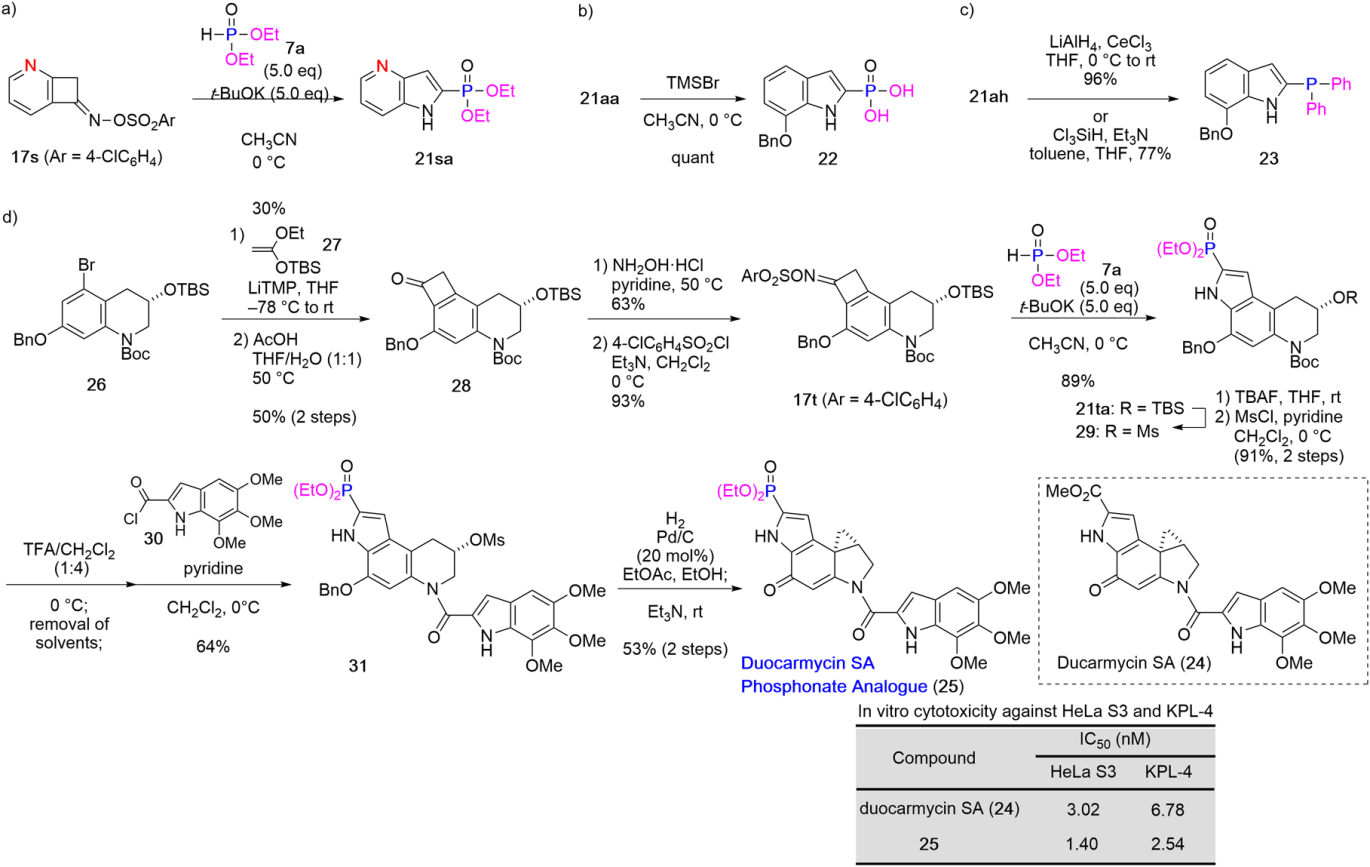
Applications of the 2-Phosphorus-Substituted Indole Synthesis

Finally, the high functional group compatibility of this 2-phosphorus-substituted indole synthesis was demonstrated by the synthesis of a duocarmycin SA phosphonate analog (Scheme 5d). Duocarmycin SA and the related compounds,^20^ which have been isolated from *Streptomyces* sp., exhibit extremely potent antitumor activity by alkylating DNA at the dienone cyclopropane moiety in the cyclopropa­[*e*]­pyrroloindole (CPI) segment.^21^ Boger et al. investigated the structure–activity relationships of duocarmycins and their derivatives^21a^ and found that the methoxy carbonyl group in the CPI segment resulted in enhanced binding and was thus crucial for efficient DNA alkylation. In this study, we designed an unnatural duocarmycin SA derivative by replacing the methoxy carbonyl group in the CPI segment with a phosphonate group, aiming to increase the antitumor activity by enhancing the binding.

The duocarmycin SA phosphonate analog was synthesized via completely regioselective [2 + 2] cycloaddition^12,22^ of a highly functionalized benzyne intermediate, which had been generated by treatment of a 5-bromotetrahydroquinoline derivative **26** ^12a,23a^ with lithium tetramethylpiperidide, with ketene silyl acetal, followed by treatment with acetic acid to afford benzocyclobutenone **28**. After conversion of **28** to oxime sulfonate **17t** in two steps, ring expansion of **17t** under standard conditions furnished the desired tricyclic 2-phosphorus-substituted indole **21ta** in high yield (89%). Then, the TBS group was replaced with a mesyl (Ms) group, and the *tert*-butyloxycarbonyl group was removed, affording a cyclic secondary amine, which was then condensed with acid chloride **30** ^23b^ to generate amide **31**. Finally, transannular cyclopropanation via one-pot Pd/C-catalyzed debenzylation and treatment of the resulting phenol with triethylamine^12a^ generated the desired duocarmycin SA phosphonate analog **25**. The antitumor activities of **25** and duocarmycin SA (**24**) were compared via an in vitro assay based on the inhibition of HeLa S3 and KPL-4 cell growth. Interestingly, the IC_50_ values of **25** for HeLa S3 and KPL-4 cells were 2 and 2.7 times lower (indicating greater potency), respectively, than that of duocarmycin SA (**24**).

In conclusion, we developed a method to synthesize 2-phosphorus-substituted indoles via ring expansion of benzocyclobutenone oxime sulfonates with broad functional group compatibility. The utility of this protocol was demonstrated by the construction of a highly functionalized piperidine-fused 2-phosphorus-substituted indole derivative, which was converted into an unnatural duocarmycin SA phosphonate analog that exhibited greater antitumor activity than its parent duocarmycin SA. The 2-phosphorus-substituted indoles synthesized by this method are expected to be widely applicable in drug discovery, organic synthesis, and materials science.

## Supplementary Material



## Data Availability

The data underlying this study are available in the published article and its Supporting Information.

## Notes

Full experimental procedure and ^1^H and ^13^C NMR spectra of compounds **17**, **21**–**23**, **25**, **28**, **29**, and **31** (PDF)
