# Supplementary material for: Synthesis of 2‑Phosphorus-Substituted Indoles via Ring Expansion of Benzocyclobutenone Oxime Sulfonates
Source: Org Lett. 2025 May 29;27(28):7507–12. doi: 10.1021/acs.orglett.5c01778 (PMC12281575; doi:10.1021/acs.orglett.5c01778)
Supplement: Supplementary file 1 [file ol5c01778_si_001.pdf]

Supporting Information for

Synthesis of 2-Phosphorus-Substituted Indoles via Ring Expansion of Benzocyclobutenone Oxime Sulfonates

Yusuke Kanno,[1] Yumi Yamashita,[1] Akira Sugiyama,[2] Tatsuhiko Kodama,[3] Juri Sakata,[1] and Hidetoshi Tokuyama[1]*

[1]Graduate School of Pharmaceutical Sciences, Tohoku University, 6-3 Aoba, Aramaki, Aoba-ku, Sendai 980-8578, Japan; [2]Department of Clinical Pharmaceutical Sciences, Hoshi University, 2-4-41 Ebara, Shinagawa-Ku, Tokyo 142-8501, Japan; [3]Research Center for Advanced Science and Technology, The University of Tokyo, 4-6-1 Komaba, Meguro-ku, Tokyo, 153-8904, Japan.

Contents

1. General Remarks S1
2. Experimental Procedures S2
3. Biological Experiment: Methods and Supplementary Data S37
4. References S39
5. ^{1}H and ^{13}C NMR Spectra S41
6. ^{31}P NMR Spectra S99

1. General Remarks

All moisture or air sensitive reactions were carried out under a positive pressure of atmosphere of argon in a dried glassware. Materials were obtained from commercial suppliers and used without further purification unless otherwise mentioned. DMSO, and Et_3N were distilled from CaH_2. Anhydrous THF, MeCN, and CH_2Cl_2 were purchased from commercial suppliers and used after passing a Glass Contour Solvent System. Flash column chromatography was performed on Silica Gel 60N (spherical neutral, 40-50 μm) using the indicated solvent. Preparative TLC was performed on 60 F_{254} glass plates precoated with a 0.25 mm or thickness of silica gel. Analytical TLC was performed on 60 F_{254} glass plates precoated with a 0.25 mm or thickness of silica gel. Chemical shifts for ^{1}H NMR are reported in parts per million (ppm) downfield from tetramethylsilane (0 ppm), chloroform (7.26 ppm), or DMSO (2.49 ppm) as the internal standard and coupling constants are in Hertz (Hz). The following abbreviations are used for spin multiplicity: s = singlet, d = doublet, t = triplet, q = quartet, m = multiplet, and br = broad. Chemical shifts for ^{13}C NMR are reported in ppm, relative to the central line of a triplet at 77.0 ppm for deuteriochloroform or the line at 39.7 ppm for deuteriodimethyl sulfoxide. Chemical shifts for ^{31}P NMR are reported in ppm downfield from triphenyl phosphine (–6 ppm) or H_3PO_4 (0 ppm) as the external standard. IR spectra were measured on a FT/IR spectrometer. High resolution mass spectra (HRMS) were recorded on EI or FAB or ESI. EI and FAB mass spectra were recorded on JEOL JMS-DX303, JEOL JMS-700 and JEOL JMS-T 100 GC with magnetic sector TOF mass analyzer. ESI mass spectra were recorded on a Bruker micrOTOF II with TOF mass analyzer. Optical rotation was measured on a polarimeter. All melting points were determined on a micro melting points apparatus and were uncorrected.

2. Experimental Procedures

Preparation of Benzocyclobutenone Oxime Sulfonates

Oxime sulfonate 17b

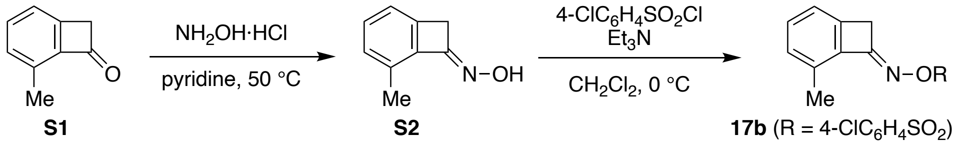

To a solution of benzocyclobutenone **S1**[1] (661 mg, 5.00 mmol) in pyridine (5.0 mL) was added hydroxylamine hydrochloride (1.39 g, 20.0 mmol) at room temperature. After stirring at 50 °C for 1.5 h, the reaction was quenched with 1 M HCl aq., and the mixture was extracted with EtOAc three times. The combined organic extracts were washed with 1 M HCl aq. and brine, dried over anhydrous sodium sulfate, and filtered. The organic solvents were removed under reduced pressure to give a crude oxime **S2**, which was used in the next reaction without further purification.

To a solution of crude oxime **S2** in CH_2Cl_2 (10.9 mL) was added 4-$ClC_6H_4SO_2Cl$ (2.30 g, 10.9 mmol) and Et_3N (3.64 mL, 26.1 mmol) at 0 °C. After stirring at 0 °C for 15 min, the reaction was quenched with 1 M HCl aq., and the mixture was extracted with CH_2Cl_2 three times. The combined organic extracts were washed with 1 M HCl aq. and brine, dried over anhydrous sodium sulfate, and filtered. The organic solvents were removed under reduced pressure to give a crude material, which was purified by flash silica gel column chromatography (hexanes-EtOAc = 10:1) to afford oxime sulfonate **17b** (469 mg, 1.46 mmol, 29%, 2 steps from **S1**). A white crystalline solid; mp: 147–148 ºC (hexanes-EtOAc); IR (film): 1584, 1396, 1376, 1191, 1174, 1095, 1087, 827, 755, 689, 614, 547 cm^{-1}; ^1H NMR (400 MHz, $CDCl_3$): δ 7.99 (2H, d, J = 8.8 Hz), 7.54 (2H, d, J = 8.8 Hz), 7.32 (1H, dd, J = 7.2, 7.2 Hz), 7.09 (2H, d, J = 7.2 Hz), 3.94 (2H, s), 2.31 (3H, s); ^{13}C NMR (100 MHz, $CDCl_3$): δ 162.1, 144.2, 140.7, 137.3, 134.0, 133.8, 130.4, 129.8, 129.3, 120.4, 39.9, 18.2 (one signal is missing due to overlap); HRMS (ESI) *m/z*: $[M+Na]^+$ Calcd for $C_{15}H_{12}ClNNaO_3S$ 344.0119; Found 344.0111.

Oxime sulfonate 17c

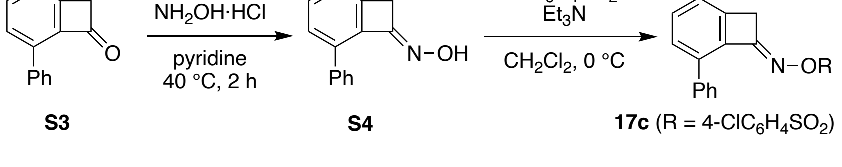

To a solution of benzocyclobutenone **S3**[2] (755 mg, 3.89 mmol) in pyridine (3.9 mL) was added hydroxylamine hydrochloride (675 mg, 9.72 mmol) at room temperature. After stirring at 40 °C for 2 h, the reaction was quenched with 1 M HCl aq., and the mixture was extracted with EtOAc three times. The combined organic extracts were washed with 1 M HCl aq. and brine, dried over anhydrous

sodium sulfate, and filtered. The organic solvents were removed under reduced pressure to give a crude oxime **S4**, which was used in the next reaction without further purification.

To a solution of the crude oxime **S4** in CH_2Cl_2 (5.0 mL) was added 4-$ClC_6H_4SO_2Cl$ (1.59 g, 7.53 mmol) and Et_3N (2.51 mL, 18.0 mmol) at 0 °C. After stirring at 0 °C for 30 min, the reaction was quenched with 1 M HCl aq., and the mixture was extracted with CH_2Cl_2 three times. The combined organic extracts were washed with 1 M HCl aq. and brine, dried over anhydrous sodium sulfate, and filtered. The organic solvents were removed under reduced pressure to give a crude material, which was purified by flash silica gel column chromatography (hexanes-EtOAc = 6:1) to afford oxime sulfonate **17c** (1.02 g, 2.65 mmol, 89%, 2 steps from **S3**). A pale brown crystalline solid; mp: 113–115 ºC (EtOAc); IR (film): 1376, 1191, 1174, 1095, 1087, 827, 755, 689, 614 cm^{-1}; ^1H NMR (400 MHz, $CDCl_3$): δ 7.98 (2H, d, J = 8.8 Hz), 7.79–7.76 (2H, m), 7.59 (1H, d, J = 7.6 Hz), 7.53–7.49 (3H, m), 7.44–7.42 (3H, m), 7.24 (1H, d, J = 8.0 Hz), 4.00 (2H, s); ^{13}C NMR (100 MHz, $CDCl_3$): δ ,161.9, 145.1, 140.8, 136.3, 135.4, 134.8, 134.3, 133.9, 130.7, 129.1, 128.9, 128.6, 127.7, 126.9, 121.9, 39.5; HRMS (ESI) *m/z*: $[M+Na]^+$ Calcd for $C_{20}H_{14}ClNNaO_3S$ 406.0275; Found 406.0294.

Oxime sulfonate 17d

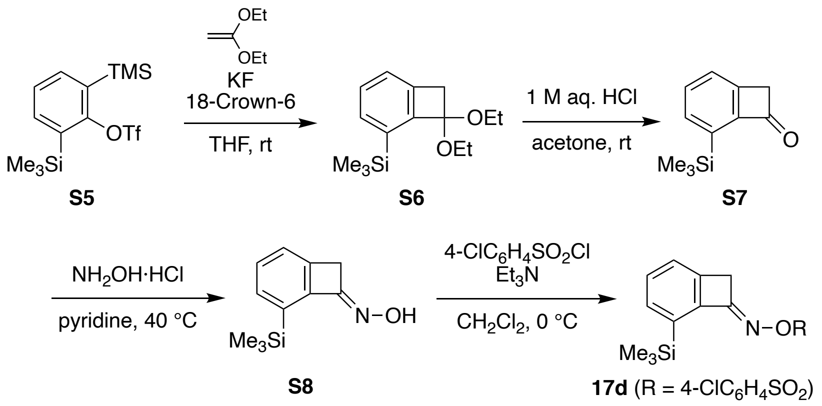

To a solution of triflate **S5**[3] (254 mg, 685 μmol) in THF (3.4 mL) was added ketene diethyl acetal (463 μL, 3.43 mmol), potassium fluoride (119 mg, 2.06 mmol), and 18-crown-6 (543 mg, 2.06 mmol) at room temperature. After stirring at room temperature for 18 h, the reaction was quenched with H_2O and the mixture was extracted with EtOAc three times. The combined organic extracts were washed with H_2O and brine, dried over anhydrous sodium sulfate, and filtered. The organic solvents were removed under reduced pressure to give a crude acetal **S6**, which was used in the next reaction without further purification.

To a solution of the crude **S6** in acetone (6.0 mL) was added 1 M HCl aq. (857 μL) at room temperature. After stirring at room temperature for 3.5 h, the solvent was removed under reduced pressure and the residue was extracted with EtOAc three times. The combined organic extracts were washed with H_2O and brine, dried over anhydrous sodium sulfate, and filtered. The organic solvents

were removed under reduced pressure to give a crude **S7**, which was used in the next reaction without further purification.

To a solution of the crude benzocyclobutenone **S7** in pyridine (0.85 mL) was added hydroxylamine hydrochloride (148 mg, 2.14 mmol) at room temperature. After stirring at 40 °C for 5 h, additional hydroxylamuine hydroxhloride (36.0 mg, 518 µmol) was added to the reaction mixture. After the solution was stirred for 1 h, the reaction was quenched with 1 M HCl aq. and the mixture was extracted with EtOAc three times. The combined organic extracts were washed with 1 M HCl aq. and brine, dried over anhydrous sodium sulfate, and filtered. The organic solvents were removed under reduced pressure to give a crude **S8**, which was used in the next reaction without further purification.

To a solution of the crude oxime **S8** in CH_2Cl_2 (1.4 mL) was added 4-$ClC_6H_4SO_2Cl$ (451 mg, 2.14 mmol) and Et_3N (714 µL, 5.12 mmol) at 0 °C. After stirring at 0 °C for 25 min, the reaction was quenched with 1 M HCl aq. The mixture was extracted with CH_2Cl_2 three times. The combined organic extracts were washed with 1 M HCl aq. and brine, dried over anhydrous sodium sulfate, and filtered. The organic solvents were removed under reduced pressure to give a crude material, which was purified by flash silica gel column chromatography (hexanes-EtOAc = 6:1) to afford oxime sulfonate **17d** (157 mg, 413 µmol, 48%, 4 steps from **S5**). A white crystalline solid; mp: 236–237 °C, decomposition (EtOAc); IR (film): 2955, 1558, 1507, 1396, 1377, 1192, 841, 816, 755 cm^{-1}; ^1H NMR (400 MHz, $CDCl_3$): δ 7.97 (2H, d, *J* = 8.8 Hz), 7.52 (2H, d, *J* = 8.8 Hz), 7.43 (1H, d, *J* = 8.0 Hz), 7.37 (1H, dd, *J* = 8.0, 5.6 Hz), 7.27 (1H, d, *J* = 5.6 Hz), 3.95 (2H, s), 0.24 (9H, s); ^{13}C NMR (100 MHz, $CDCl_3$): δ 162.4, 144.2, 143.1, 140.7, 137.3, 134.2, 134.0, 132.7, 130.6, 129.2, 123.4, 39.5, –2.2; HRMS (ESI) *m/z*: $[M+H]^+$ Calcd for $C_{17}H_{19}ClNO_3SSi$ 380.0538; Found 380.0531.

Oxime sulfonate 17e

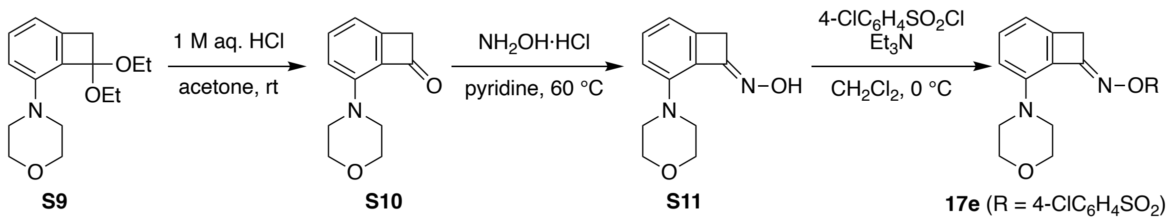

To a solution of acetal **S9**[4] (35.0 mg, 126 µmol) in acetone (0.70 mL) was added 1 M HCl aq. (0.10 mL) at room temperature. After stirring at room temperature for 5 h, the solvent was removed under reduced pressure and the residue was extracted with EtOAc three times. The combined organic extracts were washed with H_2O and brine, dried over anhydrous sodium sulfate, and filtered. The organic solvents were removed under reduced pressure to give a crude benzocyclobutenone **S10**, which was used in the next reaction without further purification.

To a solution of the crude benzocyclobutenone **S10** in pyridine (0.12 mL) was added hydroxylamine hydrochloride (21.5 mg, 309 μmol) at room temperature. After stirring at 60 °C for 6.5 h, the reaction was quenched with 1 M HCl aq., and the mixture was extracted with EtOAc three times. The combined organic extracts were washed with 1 M HCl aq. and brine, dried over anhydrous sodium sulfate, and filtered. The organic solvents were removed under reduced pressure to give a crude oxime **S11**, which was used in the next reaction without further purification.

To a solution of the crude oxime **S11** in CH_2Cl_2 (0.89 mL) were added 4-$ClC_6H_4SO_2Cl$ (46.9 mg, 222 μmol) and Et_3N (74 μL, 0.53 mmol) at 0 °C. After stirring at 0 °C for 30 min, the reaction was quenched with 1 M HCl aq., and the mixture was extracted with CH_2Cl_2 three times. The combined organic extracts were washed with 1 M HCl aq. and brine, dried over anhydrous sodium sulfate, and filtered. The organic solvents were removed under reduced pressure to give a crude material, which was purified by flash silica gel column chromatography (hexanes-EtOAc = 3:1) to afford oxime sulfonate **17e** (15.5 mg, 39.5 μmol, 31%, 3 steps from **S9**). A yellow crystalline solid; mp: 91.8–93.1 ºC, decomposition (hexanes-EtOAc); IR (film): 2963, 2855, 1670, 1477, 1449, 1279, 1218, 1016, 989, 890, 728, 637, 629, 553 cm^{-1}; ^{1}H NMR (400 MHz, $CDCl_3$): δ 7.91 (2H, d, J = 8.8 Hz), 7.53 (2H, d, J = 8.8 Hz), 7.31 (1H, dd, J = 8.8, 7.2 Hz), 6.66 (1H, d, J = 7.2 Hz), 6.57 (1H, d, J = 8.8 Hz), 3.84 (2H, s), 3.77 (4H, t, J = 4.8 Hz), 3.37 (4H, t, J = 4.8 Hz); ^{13}C NMR (100 MHz, $CDCl_3$): δ 160.9, 145.1, 144.7, 140.4, 135.7, 133.7, 130.1, 128.9, 121.7, 112.4, 112.3, 66.1, 47.2, 38.5; HRMS (ESI) *m/z*: $[M+H]^+$ Calcd for $C_{18}H_{18}ClN_2O_4S$ 393.0670; Found 393.0665.

Oxime sulfonate 17f

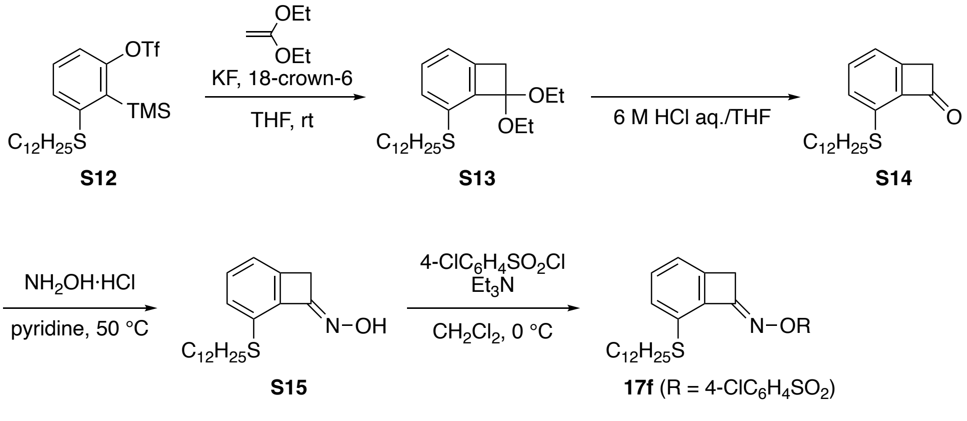

To a solution of triflate **S12**[5] (227 mg, 0.46 mmol) and ketene diethyl acetal (246.7 μL, 1.82 mmol) in THF (2.3 ml) was added 18-crown-6 (361 mg, 1.37 mmol) and potassium fluoride (79 mg, 1.37 mmol). After stirring at room temperature for 13 h, the reaction was quenched with H_2O and the mixture was extracted with EtOAc three times. The combined organic extracts were washed with brine, dried over anhydrous sodium sulfate, and filtered. The organic solvents were removed under reduced pressure to give a crude **S13**, which was used in the next reaction without further purification.

To a solution of crude **S13** in THF (4.6 mL) was added 6 M HCl aq. (0.920 mL). After stirring at room temperature for 12 h, the reaction was quenched with saturated aqueous $NaHCO_3$, and the mixture was extracted with EtOAc three times. The combined organic extracts were washed with saturated aqueous $NaHCO_3$ and brine, dried over anhydrous sodium sulfate, and filtered. The organic solvents were removed under reduced pressure to give a crude **S14**, which was used in the next reaction without further purification.

To a solution of crude **S14** in pyridine (1.5 mL) was added $NH_2OH{\cdot}HCl$ (79.0 mg, 1.14 mmol) at 50 °C. After stirring at 50 °C for 2.0 h, the reaction mixture was diluted with EtOAc, and the mixture was washed with 1 M HCl aq. and brine, dried over anhydrous sodium sulfate, and filtered. The organic solvents were removed under reduced pressure to give a crude **S15**, which was used in the next reaction without further purification.

To a solution of crude **S15** in CH_2Cl_2 (2.3 mL) were added 4-$ClC_6H_4SO_2Cl$ (252 mg, 1.19 mmol) and Et_3N (0.381 mL, 2.73 mmol) at 0 °C. After stirring at 0 °C for 1 h, the reaction was quenched with 1 M HCl aq., and the mixture was extracted with CH_2Cl_2 three times. The combined organic extracts were washed with brine, dried over anhydrous sodium sulfate, and filtered. The organic solvents were removed under reduced pressure to give a crude material, which was purified by flash silica gel column chromatography (hexane-CH_2Cl_2 = 2:1) to afford oxime sulfonate **17f** (63 mg, 0.125 mmol, 27%, 4 steps from **S12**); A white crystalline solid; mp: 70–71 ºC (CH_2Cl_2); IR (film): 2920, 2852, 1573, 1458, 1378, 1192, 1085, 822, 610 cm^{-1}; ^1H NMR (600 MHz, $CDCl_3$): δ 7.99 (2H, d, *J* = 9.0 Hz), 7.53 (2H, d, *J* = 9.0 Hz), 7.29 (1H, dd, *J* = 7.8, 7.8 Hz), 7.16 (1H, d, *J* = 7.8 Hz), 7.01 (1H, d, *J* = 7.8 Hz), 3.91 (2H, s), 3.02 (2H, t, *J* = 7.2 Hz), 1.59–1.54 (2H, m), 1.36–1.34 (2H, m), 1.31–1.26 (16H, m), 0.88 (3H, t, *J* = 6.6 Hz); ^{13}C NMR (150 MHz, $CDCl_3$): δ 160.6, 145.4, 140.7, 135.1, 135.0, 134.0, 133.8, 130.5, 129.2, 127.0, 119.0, 39.4, 31.9, 31.3, 29.61, 29.59, 29.56, 29.4, 29.3, 29.2, 29.1, 28.6, 22.6, 14.1; HRMS (ESI) *m/z*: $[M+Na]^+$ Calcd for $C_{26}H_{34}ClNNaO_3S_2$ 530.1561; Found 530.1564.

Oxime sulfonate 17g

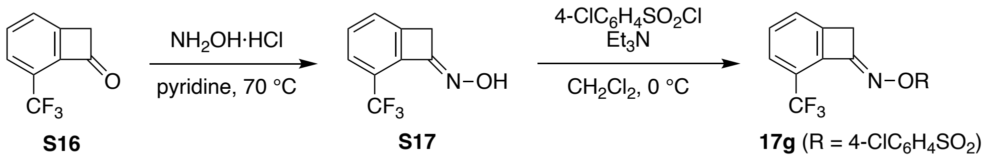

To a solution of benzocyclobutenone **S16**[6] (127 mg, 682 μmol) in pyridine (1.0 mL) was added hydroxylamine hydrochloride (119 mg, 1.71 mmol) at room temperature. After stirring at 70 °C for 12 h, the reaction was quenched with 1 M HCl aq., and the mixture was extracted with EtOAc three times. The combined organic extracts were washed with 1 M aq. HCl and brine, dried over anhydrous

sodium sulfate, and filtered. The organic solvents were removed under reduced pressure to give a crude oxime **S17**, which was used in the next reaction without further purification.

To a solution of crude oxime **S17** in CH_2Cl_2 (3.2 mL) was added 4-$ClC_6H_4SO_2Cl$ (333 mg, 1.58 mmol) and Et_3N (527 μL, 3.79 mmol) at 0 °C. After stirring at 0 °C for 20 min, the reaction was quenched with 1 M HCl aq., and the mixture was extracted with CH_2Cl_2 three times. The combined organic extracts were washed with 1 M HCl aq. and brine, dried over anhydrous sodium sulfate, and filtered. The organic solvents were removed under reduced pressure to give a crude material, which was purified by flash silica gel column chromatography (hexanes-EtOAc = 5:1 to 3:1) to afford oxime sulfonate **17g** (157 mg, 417 μmol, 61%, 2 steps from **S16**). A white crystalline solid; mp: 143–144 ºC ($CHCl_3$); IR (film): 3098, 1379, 1169, 1137, 1119, 837, 748, 645 cm^{-1}; ^{1}H NMR (400 MHz, $CDCl_3$): δ 7.99 (2H, d, J = 7.6 Hz), 7.56–7.53 (5H, m), 4.03 (2H, s); ^{13}C NMR (100 MHz, $CDCl_3$): δ 145.2, 140.9, 135.1, 133.7, 133.5, 130.6, 129.8 (q, J = 91.4 Hz), 129.2, 127.0, 125.8 (q, J = 4.1 Hz), 124.0 (q, J = 79.9 Hz), 122.0 (q, J = 271 Hz), 40.5; HRMS (FAB) *m/z*: $[M+H]^+$ Calcd for $C_{15}H_{10}ClF_3NO_3S$ 376.0017; Found 376.0023.

Oxime sulfonate 17h

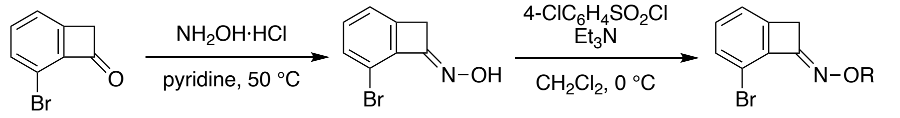

S18 **S19** **17h** (R = 4-$ClC_6H_4SO_2$)

To a solution of benzocyclobutenone **S18**[7] (129 mg, 658 μmol) in pyridine (3.3 mL) was added hydroxylamine hydrochloride (183 mg, 2.63 mmol) at room temperature. After stirring at 50 °C for 2 h, the reaction was quenched with 1 M HCl aq., and the mixture was extracted with EtOAc three times. The combined organic extracts were washed with 1 M HCl aq. and brine, dried over anhydrous sodium sulfate, and filtered. The organic solvents were removed under reduced pressure to give a crude material, which was used in the next reaction without further purification.

To a solution of oxime **S19** in CH_2Cl_2 (4.2 mL) were added 4-$ClC_6H_4SO_2Cl$ (329 mg, 1.56 mmol) and Et_3N (520 μL, 3.74 mmol) at 0 °C. After stirring at 0 °C for 2 h, the reaction was quenched with 1 M HCl aq., and the mixture was extracted with CH_2Cl_2 three times. The combined organic extracts were washed with 1 M HCl aq. and brine, dried over anhydrous sodium sulfate, and filtered. The organic solvents were removed under reduced pressure to give a crude material, which was purified by flash silica gel column chromatography (hexanes-EtOAc = 9:1) to afford oxime sulfonate **17h** (134 mg, 347 μmol, 53%, 2 steps from **S18**). A white solid; IR (film): 3096, 2919, 1371, 1192, 823, 754, 650 cm^{-1}; ^{1}H NMR (600 MHz, $CDCl_3$): δ 8.01 (2H, d, J = 9.0 Hz), 7.55 (2H, d, J = 9.0 Hz), 7.45 (1H, d, J = 7.8 Hz), 7.30 (1H, dd, J = 7.8, 7.8 Hz), 7.25 (1H, d, J = 7.8 Hz), 3.97 (2H, s); ^{13}C NMR (100 MHz, $CDCl_3$): δ 159.6, 146.6, 140.9, 138.6, 134.8, 133.6, 132.3, 130.6, 129.3, 122.1,

114.3, 40.2; HRMS (FAB) *m/z*: [M+H]$^+$ Calcd for $C_{14}H_{10}BrClNO_3S$ 385.9248; Found 385.9265.

Oxime sulfonate 17i

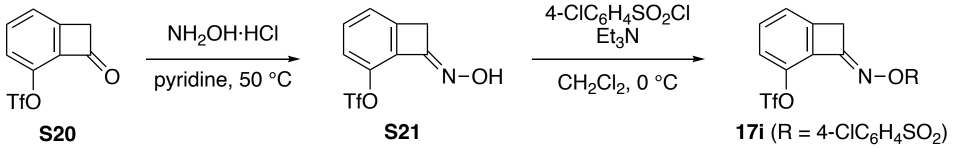

To a solution of benzocyclobutenone **S20**[8] (143 mg, 0.539 mmol) in pyridine (2.5 mL) was added $NH_2OH \cdot HCl$ (93.7 mg, 1.35 mmol) at 50 °C. After stirring at 50 °C for 4 h, the reaction mixture was diluted with EtOAc, and the mixture was washed with 1 M HCl aq. and brine, dried over anhydrous sodium sulfate, and filtered. The organic solvents were removed under reduced pressure to give a crude **S17**, which was used in the next reaction without further purification.

To a solution of crude **S21** in CH_2Cl_2 (6.2 mL) were added 4-$ClC_6H_4SO_2Cl$ (239 mg, 1.13 mmol) and Et_3N (451 µL, 3.23 mmol) at –78 °C. After stirring at 0 °C for 1 h, the reaction was quenched with 1 M HCl aq., and the mixture was extracted with CH_2Cl_2 three times. The combined organic extracts were washed with brine, dried over anhydrous sodium sulfate, and filtered. The organic solvents were removed under reduced pressure to give a crude material, which was purified by flash silica gel column chromatography (hexane-EtOAc = 3:1) to afford oxime sulfonate **17i** (51.4 mg, 0.113 mmol, 21%, 2 steps from **S20**); A white crystalline solid; mp: 148–150 ºC (hexanes-EtOAc); IR (film): 3092, 2980, 1585, 1475, 1427, 1380, 1216, 1192, 1139, 1093, 987, 820, 756 cm^{-1}; ^{1}H NMR (600 MHz, $CDCl_3$): δ 8.00 (2H, d, *J* = 9.0 Hz), 7.63 (1H, dd, *J* = 7.2, 8.4 Hz), 7.54 (2H, d, 9.0 Hz), 7.39 (1H, d, *J* = 7.8 Hz), 7.28 (1H, d, *J* = 4.8 Hz), 3.97 (2H, s); ^{13}C NMR (150 MHz, $CDCl_3$): δ 156.8, 146.6, 141.1, 139.8, 135.7, 133.2, 130.7, 130.1, 129.3, 123.9, 121.8, 116.9, 40.8; HRMS (ESI) *m/z*: [M+Na]$^+$ Calcd for $C_{15}H_9ClF_3NNaO_6S_2$ 477.9404; Found 477.9414.

Oxime sulfonate 17j

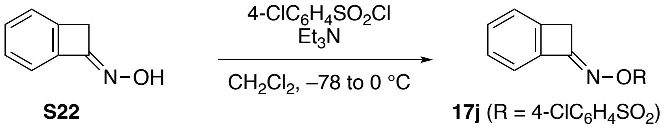

To a solution of oxime **S22**[9] (79.9 mg, 0.600 mmol) in CH_2Cl_2 (2.0 mL) were added 4-$ClC_6H_4SO_2Cl$ (200 mg, 0.948 mmol) and Et_3N (0.167 mL, 3.60 mmol) at –78 °C. After stirring at 0 °C for 30 min, the reaction was quenched with 1 M HCl aq., and the mixture was extracted with CH_2Cl_2 three times. The combined organic extracts were washed with brine, dried over anhydrous sodium sulfate, and filtered. The organic solvents were removed under reduced pressure to give a crude material, which was purified by flash silica gel column chromatography (hexane-EtOAc = 4:1) to afford oxime sulfonate **17j** (45.1 mg, 0.149 mmol, 24%); A white solid; IR (film): 2917, 1683,

1577, 1474, 1376, 1191, 1093, 791, 753, 636 cm^{-1}; ^{1}H NMR (400 MHz, $CDCl_3$): δ 7.98 (2H, d, J = 8.8 Hz), 7.54 (2H, d, J = 8.8 Hz), 7.44 (1H, ddd, J = 7.2, 7.2, 2.0 Hz), 7.35–7.30 (3H, m); ^{13}C NMR (150 MHz, $CDCl_3$): δ 161.7, 144.6, 140.8, 138.2, 134.0, 133.8, 130.3, 129.4, 129.0, 123.5, 121.5, 40.2; HRMS (ESI) *m/z*: [M+H]$^+$ Calcd for $C_{14}H_{11}ClNO_3S$ 308.0143; Found 308.0148.

Oxime S24

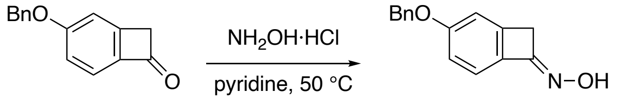

To a solution of benzocyclobutenone **S23**[10] (300 mg, 1.34 mmol) in pyridine (2.2 mL) was added $NH_2OH{\cdot}HCl$ (233 mg, 3.35 mmol) at 50 °C. After stirring at 50 °C for 1 h, the reaction mixture was diluted with EtOAc, and the mixture was washed with 1 M HCl aq. and brine, dried over anhydrous sodium sulfate, and filtered. The organic solvents were removed under reduced pressure to give a crude material, which was purified by flash silica gel column chromatography (hexane-EtOAc = 4:1) to afford oxime **S24** (210 mg, 0.880 mmol, 66%) as a mixture of isomer (major isomer/minor isomer = 5:3); A white solid; IR (film): 3226, 2866, 1702, 1591, 1453, 1270, 1247, 1125, 1025, 943, 696 cm^{-1}; ^{1}H NMR (600 MHz, $CDCl_3$): δ 7.43–7.38 (4.62H, m), 7.35–7.33 (1H, m), 7.22 (0.38H, d, J = 8.4 Hz), 6.95 (0.62H, d, J = 7.8 Hz), 6.93–6.89 (2H, m), 6.69 (0.38H, s), 5.11 (1.24H, s), 5.09 (0.76H, s), 3.86 (0.76H, s), 3.77 (1.24H, s); ^{13}C NMR (150 MHz, $CDCl_3$): δ 162.0, 161.7, 153.1, 150.5, 146.9, 145.5, 136.44, 136.37, 133.2, 132.2, 128.6, 128.14, 128.11, 127.4, 124.7, 121.3, 116.4, 116.3, 109.4, 109.0, 70.3, 38.6, 38.1 (Three signals are missing due to overlap); HRMS (ESI) *m/z*: [M+H]$^+$ Calcd for $C_{15}H_{14}NO_2$ 240.1019; Found 240.1023.

Oxime sulfonate 17k

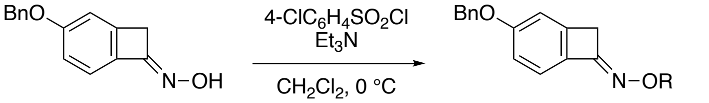

To a solution of oxime **S24** (136 mg, 0.607 mmol) in CH_2Cl_2 (6.1 mL) were added 4-$ClC_6H_4SO_2Cl$ (202 mg, 0.957 mmol) and Et_3N (508 μL, 3.64 mmol) at 0 °C. After stirring at 0 °C for 1 h, the reaction was quenched with 1 M HCl aq., and the mixture was extracted with CH_2Cl_2 three times. The combined organic extracts were washed with brine, dried over anhydrous sodium sulfate, and filtered. The organic solvents were removed under reduced pressure to give a crude material, which was purified by flash silica gel column chromatography (hexane-EtOAc = 4:1) to afford oxime sulfonate **17k** (23.2 mg, 56.1 μmol, 9%); A white solid; IR (film): 3033, 2920, 2873, 1757, 1580, 1461, 1325, 1276, 1243, 1082, 1004, 960, 739 cm^{-1}; ^{1}H NMR (400 MHz, $CDCl_3$): δ 7.97 (2H, d, J =

8.8 Hz), 7.54 (2H, d, *J* = 8.8 Hz), 7.40–7.34 (5H, m), 7.24 (1H, d, *J* = 8.8 Hz), 6.94 (1H, d, *J* = 8.8 Hz), 6.88 (1H, s), 5.09 (2H, s), 3.90 (2H, s); ^{13}C NMR (150 MHz, $CDCl_3$): δ 163.6, 160.6, 146.2, 140.6, 135.9, 134.1, 130.3, 129.8, 129.3, 128.7, 128.3, 127.4, 123.4, 117.7, 109.0, 70.4, 39.5; HRMS (ESI) *m/z*: $[M+H]^+$ Calcd for $C_{21}H_{17}ClNO_4S$ 414.0561; Found 414.0552.

Oxime sulfonate 17l

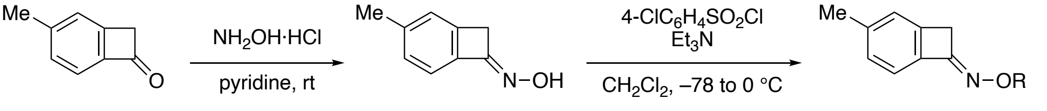

S25 **S26** **17l** (R = 4-ClC6H4SO2)

To a solution of benzocyclobutenone **S25**[11] (187 mg, 1.41 mmol) in pyridine (2.0 mL) was added $NH_2OH·HCl$ (245 mg, 3.53 mmol) at room temperature. After stirring at room temperature for 4 h, the reaction mixture was diluted with EtOAc, and the mixture was washed with 1 M HCl aq. and brine, dried over anhydrous sodium sulfate, and filtered. The organic solvents were removed under reduced pressure to give a crude **S26**, which was used in the next reaction without further purification.

To a solution of the crude **S26** in CH_2Cl_2 (4.7 mL) were added 4-$ClC_6H_4SO_2Cl$ (781 mg, 3.70 mmol) and Et_3N (1.18 mL, 8.46 mmol) at –78 °C. After stirring at 0 °C for 1 h, the reaction was quenched with 1 M HCl aq., and the mixture was extracted with CH_2Cl_2 three times. The combined organic extracts were washed with brine, dried over anhydrous sodium sulfate, and filtered. The organic solvents were removed under reduced pressure to give a crude material, which was purified by flash silica gel column chromatography (hexane-EtOAc = 4:1) to afford oxime sulfonate **17l** (56.8 mg, 0.176 mmol, 13%, 2 steps from **S25**); A white solid; IR (film): 2916, 2846, 1758, 1585, 1540, 1376, 1191, 1171, 1086, 784, 612 cm^{-1}; ^{1}H NMR (400 MHz, $CDCl_3$): δ 7.98 (2H, d, *J* = 8.8 Hz), 7.54 (2H, d, *J* = 8.8 Hz), 7.20 (1H, d, *J* = 7.6 Hz), 7.16–7.12 (2H, m), 3.93 (2H, s), 2.40 (3H, s); ^{13}C NMR (150 MHz, $CDCl_3$): δ 161.5, 145.0, 144.6, 140.7, 135.1, 134.1, 130.3, 130.1, 129.4, 123.9, 121.3, 39.8, 22.7; HRMS (ESI) *m/z*: $[M+Na]^+$ Calcd for $C_{15}H_{12}ClNNaO_3S$ 344.0119; Found 344.0123.

Oxime S28

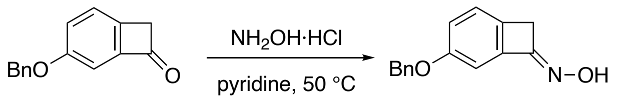

S27 **S28**

To a solution of benzocyclobutenone **S27**[12] (699 mg, 3.12 mmol) in pyridine (5.2 mL) was added $NH_2OH·HCl$ (542 mg, 7.80 mmol) at 50 °C. After stirring at 50 °C for 1 h, the reaction mixture was diluted with EtOAc, and the mixture was washed with 1 M HCl aq. and brine, dried over anhydrous sodium sulfate, and filtered. The organic solvents were removed under reduced pressure to give a crude material, which was purified by flash silica gel column chromatography (CH_2Cl_2-MeOH =

20:1) to afford oxime **S28** (585 mg, 2.45 mmol, 78%) as a mixture of isomers (major isomer/minor isomer = 12.5:1); A white solid; IR (film): 3230, 2926, 1601, 1453, 1269, 1238, 1023, 945, 734, 696 cm^{-1}; ^{1}H NMR (600 MHz, $CDCl_3$): δ 8.03 (0.93H, br s), 7.89 (0.07H, br s), 7.43–7.42 (2.00H, m), 7.40–7.36 (2.07H, m), 7.34–7.32 (0.93H, m), 7.23 (0.93H, d, *J* = 7.8 Hz), 7.18 (0.07H, d, *J* = 7.8 Hz), 7.15 (0.93H, d, *J* = 1.8 Hz), 7.06 (0.93H, dd, *J* = 7.8, 2.4 Hz), 6.99 (0.07H, dd, *J* = 7.8, 1.8 Hz), 6.93 (0.07H, d, *J* = 1.8 Hz), 5.06 (1.86H, s), 5.04 (0.14H, s), 3.85 (0.14H, s), 3.77(1.86H, m); ^{13}C NMR (150 MHz, $CDCl_3$): δ 159.3, 151.0, 141.6, 139.8, 137.6, 136.7, 136.6, 136.4, 128.6, 128.1, 127.5, 127.5, 124.4, 124.1, 120.8, 120.1, 108.1, 105.1, 70.5, 70.4, 38.3, 37.8 (Four signals are missing due to overlap); HRMS (ESI) *m/z*: [M+H]$^+$ Calcd for $C_{15}H_{14}NO_2$ 240.1019; Found 240.1013.

Oxime sulfonate 17m

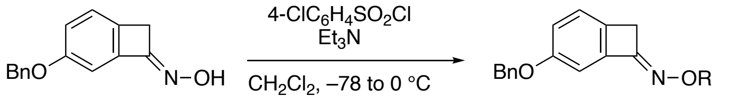

To a solution of oxime **S28** (26.3 mg, 0.110 mmol) in CH_2Cl_2 (1.1 mL) were added 4-$ClC_6H_4SO_2Cl$ (60.9 mg, 0.289 mmol) and Et_3N (0.919 mL, 0.659 mmol) at –78 °C. After stirring at 0 °C for 1 h, the reaction was quenched with 1 M HCl aq., and the mixture was extracted with CH_2Cl_2 three times. The combined organic extracts were washed with brine, dried over anhydrous sodium sulfate, and filtered. The organic solvents were removed under reduced pressure to give a crude material, which was purified by flash silica gel column chromatography (hexane-EtOAc = 8:1) to afford oxime sulfonate **17m** (24.5 mg, 0.0592 mmol, 54%); A white solid; IR (film): 3093, 2926, 1672, 1477, 1377, 1275, 1192, 1094, 1014, 810, 753 cm^{-1}; ^{1}H NMR (600 MHz, $CDCl_3$): δ 7.97 (2H, d, *J* = 8.6 Hz), 7.54 (2H, d, *J* = 8.6 Hz), 7.39–7.32 (5H, m), 7.20 (1H, d, *J* = 8.4 Hz), 7.08 (1H, dd, *J* = 8.4, 2.4 Hz), 6.89 (1H, d, *J* = 2.4 Hz), 5.02 (2H, s), 3.89 (2H, s); ^{13}C NMR (150 MHz, $CDCl_3$): δ 161.2, 159.7, 140.7, 139.0, 136.9, 136.2, 134.0, 130.3, 129.4, 128.7, 128.2, 127.3, 124.7, 123.2, 106.1, 70.4, 39.2; HRMS (ESI) *m/z*: [M+H]$^+$ Calcd for $C_{21}H_{17}ClNO_4S$ 414.0561; Found 414.0562.

Oxime sulfonate 17n

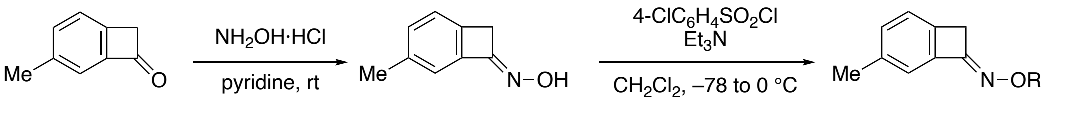

To a solution of benzocyclobutenone **S29**[11] (162 mg, 1.23 mmol) in pyridine (2.5 mL) was added $NH_2OH \cdot HCl$ (213 mg, 3.08 mmol) at room temperature. After stirring for 4 h, the reaction mixture was diluted with EtOAc, and the mixture was washed with 1 M HCl aq. and brine, dried over

anhydrous sodium sulfate, and filtered. The organic solvents were removed under reduced pressure to give a crude **S30**, which was used in the next reaction without further purification.

To a solution of crude **S30** in CH_2Cl_2 (6.2 mL) were added 4-$ClC_6H_4SO_2Cl$ (545 mg, 2.58 mmol) and Et_3N (1.00 mL, 7.38 mmol) at –78 °C. After stirring at 0 °C for 1 h, the reaction was quenched with 1 M HCl aq., and the mixture was extracted with CH_2Cl_2 three times. The combined organic extracts were washed with brine, dried over anhydrous sodium sulfate, and filtered. The organic solvents were removed under reduced pressure to give a crude material, which was purified by flash silica gel column chromatography (hexane-EtOAc = 4:1) to afford oxime sulfonate **17n** (43.1 mg, 0.134 mmol, 11%, 2 steps from **S29**); A white solid; IR (film): 3095, 2928, 1676, 1577, 1397, 1281, 1192, 1144, 1095, 1014, 870, 808, 721 cm^{-1}; ^{1}H NMR (400 MHz, $CDCl_3$): δ 7.98 (2H, d, J = 8.8 Hz), 7.54 (2H, d, J = 8.8 Hz), 7.26–7.25 (1H, m), 7.18 (1H, d, J = 7.6 Hz), 7.14 (1H, s), 3.92 (2H, s), 2.35 (3H, s); ^{13}C NMR (150 MHz, $CDCl_3$): δ 161.6, 141.6, 140.7, 139.1, 138.1, 134.8, 134.0, 130.3, 129.4, 123.1, 121.6, 39.6, 21.8; HRMS (ESI) *m/z*: $[M+Na]^+$ Calcd for $C_{15}H_{12}ClNNaO_3S$ 344.0119; Found 344.0122.

Oxime sulfonate 17o

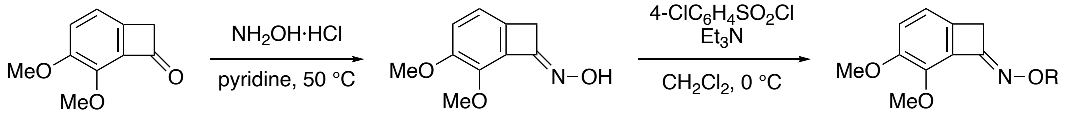

S31 **S32** **17o** (R = 4-ClC6H4SO2)

To a solution of benzocyclobutenone **S31**[13] (204 mg, 1.15 mmol) in pyridine (2.9 mL) was added $NH_2OH{\cdot}HCl$ (199 mg, 2.87 mmol) at 50 °C. After stirring at 50 °C for 4 h, the reaction mixture was diluted with EtOAc, and the mixture was washed with 1 M HCl aq. and brine, dried over anhydrous sodium sulfate, and filtered. The organic solvents were removed under reduced pressure to give a crude **S32**, which was used in the next reaction without further purification.

To a solution of the crude **S32** in CH_2Cl_2 (5.3 mL) were added 4-$ClC_6H_4SO_2Cl$ (583 mg, 2.76 mmol) and Et_3N (0.880 mL, 6.31 mmol) at 0 °C. After stirring at 0 °C for 1 h, the reaction was quenched with 1 M HCl aq., and the mixture was extracted with CH_2Cl_2 three times. The combined organic extracts were washed with brine, dried over anhydrous sodium sulfate, and filtered. The organic solvents were removed under reduced pressure to give a crude material, which was recrystallized from $CHCl_3$ to afford oxime sulfonate **17o** (209 mg, 0.57 mmol, 54%, 2 steps from **S31**); Colorless crystals, mp: 157–159 °C ($CHCl_3$); IR (film): 3097, 1496, 1474, 1375, 1260, 1193, 1174, 1084, 839, 805 cm^{-1}; ^{1}H NMR (600 MHz, $CDCl_3$): δ 7.92 (2H, d, J = 9.0 Hz), 7.53 (2H, d, J = 9.0 Hz), 6.95 (1H, d, J = 7.2 Hz), 6.77 (1H, d, J = 7.2 Hz), 4.09 (3H, s), 3.85 (2H, s), 3.84 (3H, s); ^{13}C NMR (150 MHz, $CDCl_3$): δ 160.4, 149.0, 144.4, 140.8, 135.3, 134.0, 130.3, 129.3 121.4, 118.2,

115.2, 59.3, 56.7, 38.5; HRMS (ESI) *m/z*: $[M+Na]^+$ Calcd for $C_{16}H_{14}ClNNaO_5S$ 390.0173; Found 390.0156.

Oxime S34

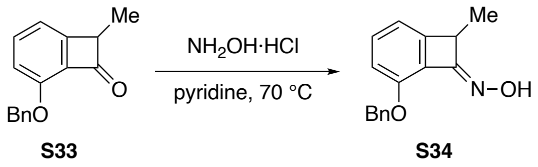

To a solution of benzocyclobutenone **S33**[14] (1.00 g, 4.20 mmol) in pyridine (20 mL) was added NH_2OH·HCl (730 mg, 10.5 mmol) at 70 °C. After stirring at 70 °C for 17 h, the reaction mixture was diluted with EtOAc, and the mixture was washed with 1 M HCl aq. and brine, dried over anhydrous sodium sulfate, and filtered. The organic solvents were removed under reduced pressure to give a crude material, which was recrystallized from hexane-CH_2Cl_2 to afford oxime **S34** (875 mg, 3.45 mmol, 82%); A white crystals; mp 112–114 °C (hexane-CH_2Cl_2); IR (film): 3291, 2960, 1699, 1581, 1480, 1388, 1268, 1167, 935, 744 cm^{-1}; ^1H NMR (600 MHz, $CDCl_3$): δ 7.72 (1H, br s), 7.44 (2H, d, *J* = 7.2 Hz), 7.36 (2H, dd, *J* = 7.2, 7.2 Hz), 7.30 (1H, t, *J* = 7.2 Hz), 7.26 (1H, dd, *J* = 7.8 Hz), 6.85–6.83 (2H, m), 5.40 (2H, s), 4.26 (1H, q, *J* = 7.2 Hz), 1.58 (3H, d, *J* = 7.2 Hz); ^{13}C NMR (150 MHz, $CDCl_3$): δ 157.2, 152.1, 151.5, 136.9, 133.4, 128.4, 127.9, 127.6, 123.7, 116.2, 114.3, 72.0, 47.4, 16.7; HRMS (ESI) *m/z*: $[M+H]^+$ Calcd for $C_{16}H_{16}NO_2$ 254.1176; Found 254.1174.

Oxime sulfonate 17p

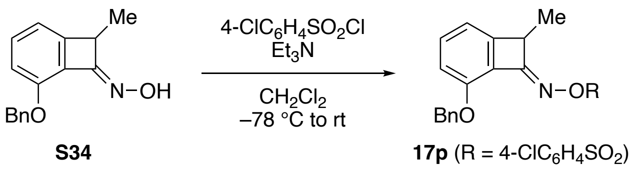

To a solution of oxime **S34** (400 mg, 1.58 mmol) in CH_2Cl_2 (15 mL) were added 4-$ClC_6H_4SO_2Cl$ (833 mg, 3.95 mmol) and Et_3N (1.32 mL, 9.47 mmol) at –78 °C. After stirring at room temperature for 1 h, the reaction was quenched with 1 M HCl aq., and the mixture was extracted with CH_2Cl_2 three times. The combined organic extracts were washed with brine, dried over anhydrous sodium sulfate, and filtered. The organic solvents were removed under reduced pressure to give a crude material, which was purified by flash silica gel column chromatography (hexane-EtOAc = 3:1) to afford oxime sulfonate **17p** (568 mg, 1.33 mmol, 84%); A white crystalline solid; mp: 102–103 ºC (CH_2Cl_2); IR (film): 3030, 2965, 1670, 1584, 1477, 1378, 1278, 1191, 1086, 819, 746 cm^{-1}; ^1H NMR (600 MHz, $CDCl_3$): δ 7.89 (2H, d, *J* = 9.0 Hz), 7.41–7.26 (8H, m), 6.85–6.84 (2H, m), 5.28 (1H, d, *J* = 12.0 Hz), 5.25 (1H, d, *J* = 12.0 Hz), 4.31 (1H, q, *J* = 7.2 Hz), 1.57 (3H, d, *J* = 7.2 Hz); ^{13}C NMR (150 MHz,

$CDCl_3$): δ 164.3, 153.3, 151.4, 140.7, 136.3, 136.1, 133.9, 130.4, 129.2, 128.6, 128.3, 127.6, 120.5, 117.3, 114.3, 72.3, 48.7, 16.8; HRMS (ESI) *m/z*: $[M+Na]^+$ Calcd for $C_{22}H_{18}ClNNaO_4S$ 450.0537; Found 450.0522.

Oxime S39

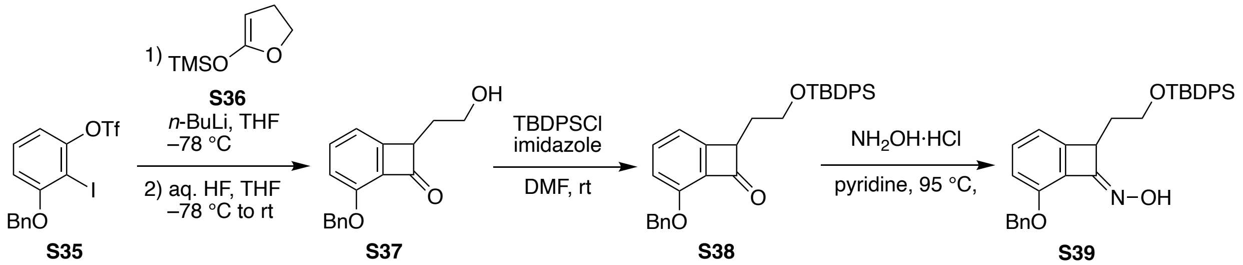

To a solution of triflate **S35**[14] (501 mg, 1.09 mmol) in THF (5.5 mL) was added a cyclic ketene silyl acetal **S36**[15] (257 μL, 1.53 mmol) at room temperature. After the mixture was cooled to –78 °C, *n*-BuLi (1.56 M in *n*-hexane, 1.40 mL, 2.18 mmol) was added dropwise to the reaction mixture. After stirring for 30 min, aq. HF (46% in H_2O, 1.04 mL, 21.8 mmol) was added and the mixture was stirred for 10 min at room temperature. Then, the reaction was quenched with sat. aq. $NaHCO_3$, and the mixture was extracted with EtOAc three times. The combined organic extracts were washed with H_2O and brine, dried over anhydrous sodium sulfate, and filtered. The organic solvents were removed under reduced pressure to give a crude benzocyclobutenone **S37**, which was used in the next reaction without further purification.

To a solution of the crude benzocyclobutenone **S37** in DMF (11 mL) were added imidazole (112 mg, 1.64 mmol) and TBDPSCl (426 μL, 1.64 mmol) at room temperature. After stirring at room temperature for 10 min, the reaction was quenched with H_2O, and the mixture was extacted with hexanes-EtOAc = 1:1 three times. The combined organic extracts were washed with H_2O and brine, dried over anhydrous sodium sulfate, and filtered. The organic solvents were removed under reduced pressure to give a crude material, which was purified by flash silica gel column chromatography (hexanes-EtOAc = 19:1) to afford benzocyclobutenone **S38** with unidentified byproducts, which was used in the next reaction without further purification.

To a solution of the crude benzocyclobutenone **S38** in pyridine (1.1 mL) was added hydroxylamine hydrochloride (190 mg, 2.73 mmol) at room temperature. After stirring at 95 °C for 3.5 h, the reaction was quenched with 1 M HCl aq., and the mixture was extracted with EtOAc three times. The combined organic extracts were washed with 1 M HCl aq. and brine, dried over anhydrous sodium sulfate, and filtered. The organic solvents were removed under reduced pressure to give a crude material, which was purified by flash silica gel column chromatography (hexanes-EtOAc = 10:1) to afford oxime **S39** (194 mg, 371 μmol, 34%, 4 steps from **S35**). A colorless oil; IR (film): 3317, 3311, 3069, 2953, 2930, 2857, 1582, 1474, 1427, 1268, 1111, 1092, 737, 702, 503 cm^{-1}; ^1H

NMR (400 MHz, $CDCl_3$): δ 7.68 (2H, d, *J* = 6.4 Hz), 7.65 (2H, d, *J* = 6.4 Hz), 7.60 (1H, br s), 7.60–7.27 (11H, m), 7.18 (1H, dd, *J* = 8.8, 7.2 Hz), 6.81 (1H, d, *J* = 8.8 Hz), 6.65 (1H, d, *J* = 7.2 Hz), 5.39 (2H, s), 4.42 (1H, dd, *J* = 8.8, 4.4 Hz), 3.88–3.84 (2H, m), 2.52 (1H, dt, *J* = 6.8, 4.4 Hz), 2.02–1.92 (1H, m), 1.06 (9H, s); ^{13}C NMR (100 MHz, $CDCl_3$): δ 156.2, 152.0, 149.9, 136.9, 135.6, 134.7, 133.8, 133.2, 129.6, 128.4, 127.9, 127.7, 124.2, 116.2, 115.2, 72.0, 61.9, 49.7, 33.8, 26.9, 19.2; HRMS (ESI) *m/z*: $[M+H]^+$ Calcd for $C_{33}H_{36}NO_3Si$ 522.2459; Found 522.2433.

Oxime sulfonate 17q

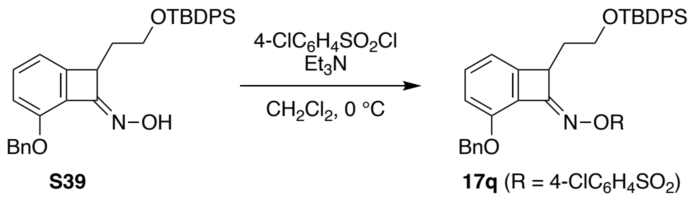

To a solution of oxime **S39** (194 mg, 0.371 mmol) in CH_2Cl_2 (1.9 mL) were added 4-$ClC_6H_4SO_2Cl$ (196 mg, 0.928 mmol) and Et_3N (310 μL, 2.23 mmol) at 0 °C. After stirring at 0 °C for 3 h, the reaction was quenched with 1 M HCl aq., and the mixture was extracted with CH_2Cl_2 three times. The combined organic extracts were washed with 1 M HCl aq. and brine, dried over anhydrous sodium sulfate, and filtered. The organic solvents were removed under reduced pressure to give a crude material, which was purified by flash silica gel column chromatography (hexanes-CH_2Cl_2 = 6:1 to hexanes-EtOAc = 15:1) to afford oxime sulfonate **17q** (194 mg, 279 μmol, 75%). A white crystalline solid; mp: 106–107 ºC (EtOAc); IR (film): 2952, 2928, 1473, 1379, 1277, 1191, 765, 742, 702 cm^{-1}; ^{1}H NMR (400 MHz, $CDCl_3$): δ 7.85 (2H, d, *J* = 8.0 Hz), 7.67 (2H, d, *J* = 6.4 Hz), 7.62 (2H, d, *J* = 6.4 Hz), 7.40–7.30 (13H, m), 7.24 (1H, dd, *J* = 8.8, 6.8 Hz), 6.81 (1H, d, *J* = 8.8 Hz), 6.21 (1H, d, *J* = 6.8 Hz), 5.27 (1H, d, *J* = 12.4 Hz), 5.24 (1H, d, *J* = 12.4 Hz), 4.47 (1H, dd, *J* = 9.5, 4.4 Hz), 3.84–3.78 (2H, m), 2.51–2.43 (1H, m), 1.95–1.90 (1H, m), 1.04 (9H, s); ^{13}C NMR (100 MHz, $CDCl_3$): δ 163.4, 153.0, 149.6, 140.5, 136.2, 135.8, 135.5, 134.7, 133.9, 133.4, 130.3, 129.6, 129.1, 128.5, 128.2, 127.7, 120.9, 117.2, 115.3, 72.2, 61.2, 50.9, 33.4, 26.8, 19.1; HRMS (ESI) *m/z*: $[M+H]^+$ Calcd for $C_{39}H_{39}ClNO_5SSi$ 696.2001; Found 696.2006.

Oxime sulfonate 17r

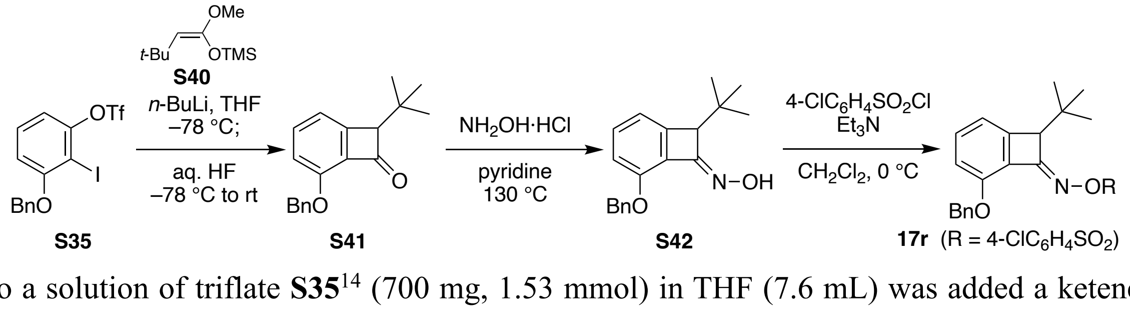

To a solution of triflate **S35**[14] (700 mg, 1.53 mmol) in THF (7.6 mL) was added a ketene silyl

acetal **S40** (340 mg, 1.68 mmol) at room temperature. After the mixture was cooled to –78 °C, *n*-BuLi (2.6 M in *n*-hexane, 705 μL, 1.83 mmol) was added dropwise to the reaction mixture. After stirring for 40 min, aq. HF (46% in H_2O, 670 μL, 15.3 mmol) was added, and the mixture was stirred for 20 min at room temperature. Then, the reaction was quenched with sat. aq. $NaHCO_3$, and the mixture was extracted with EtOAc three times. The combined organic extracts were washed with H_2O and brine, dried over anhydrous sodium sulfate, and filtered. The organic solvents were removed under reduced pressure to give a crude material, which was purified by flash silica gel column chromatography (hexanes-toluene = 4:1) to afford benzocyclobutenone **S41** with unidentified byproducts, which was used in the next reaction without further purification.

To a solution of the crude benzocyclobutenone **S41** in pyridine (1.1 mL) was added hydroxylamine hydrochloride (187 mg, 2.70 mmol) at room temperature. After stirring at 130 °C for 26 h, the reaction was quenched with 1 M HCl aq., and the mixture was extracted with EtOAc three times. The combined organic extracts were washed with 1 M HCl aq. and brine, dried over anhydrous sodium sulfate, and filtered. The organic solvents were removed under reduced pressure to give a crude oxime **S42**, which was used in the next reaction without further purification.

To a solution of crude oxime **S42** in CH_2Cl_2 (1.9 mL) was added 4-$ClC_6H_4SO_2Cl$ (198 mg, 0.938 mmol) and Et_3N (313 μL, 2.25 mmol) at 0 °C. After stirring at 0 °C for 30 min, the reaction was quenched with 1 M HCl aq., and the mixture was extracted with CH_2Cl_2 three times. The combined organic extracts were washed with 1 M HCl aq. and brine, dried over anhydrous sodium sulfate, and filtered. The organic solvents were removed under reduced pressure to give a crude material, which was purified by flash silica gel column chromatography (hexanes-EtOAc = 20:1 to 10:1) to afford oxime sulfonate **17r** (91.2 mg, 194 μmol, 13%, 3 steps from **S35**). A colorless oil; IR (film): 2961, 1589, 1379, 1192, 842, 764 cm^{-1}; ^1H NMR (400 MHz, $CDCl_3$): δ 7.89 (2H, d, *J* = 8.8 Hz), 7.37–7.31 (8H, m), 6.85 (2H, d, *J* = 7.2 Hz), 5.31 (1H, d, *J* = 12.0 Hz), 5.24 (1H, d, *J* = 12.0 Hz), 4.14 (1H, s), 1.04 (9H, s); ^{13}C NMR (100 MHz, $CDCl_3$): δ 161.6, 152.6, 149.0, 140.6, 136.4, 135.3, 134.1, 130.4, 129.2, 128.5, 128.2, 127.6, 123.2, 117.2, 116.2, 72.1, 66.1, 33.3, 28.1; HRMS (EI) *m/z*: $[M]^+$ Calcd for $C_{25}H_{24}ClNO_4S$ 469.1109; Found 469.1126.

Oxime S46

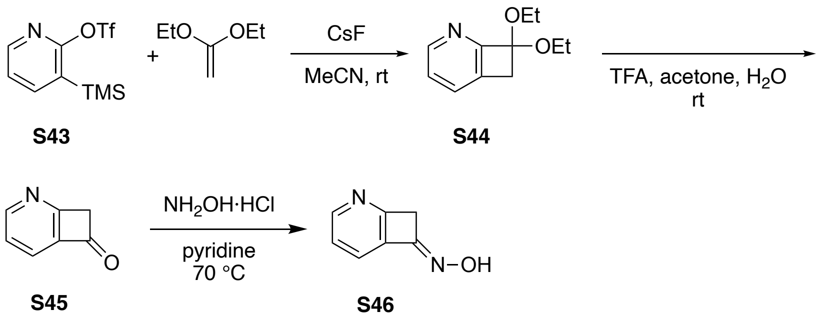

To a solution of pyridine derivative **S43**[16] (648 mg, 2.16 mmol) and ketene diethyl acetal (0.860 mL, 6.43 mmol) in MeCN (22 mL) was added CsF (1.64 g, 10.8 mmol) at room temperature. After stirring at room temperature for 4.5 h, the reaction mixture was filtered through silica gel with EtOAc. The filtrate was concentrated under reduced pressure to afford a crude adduct **S44**, which was used to the next reaction without further purification.

To a mixture of the crude acetal **S44** in acetone (3.6 mL) and H_2O (0.36 mL) was added TFA (18 mL) at room temperature. After stirring at room temperature for 3 h, the reaction mixture was concentrated under reduced pressure to afford a crude ketone **S45**, which was used to the next reaction without further purification.

To a solution of the crude ketone **S45** in pyridine (22 mL) was added hydroxylamine hydrochloride (450 mg, 6.48 mmol) at room temperature. After stirring at 70 ºC for 2 h, the reaction was quenched with 1 M aqueous HCl, and the mixture was extracted with EtOAc three times. The combined organic extracts were washed with brine, dried over anhydrous Na_2SO_4, and filtered. The organic solvents were removed under reduced pressure to give a crude material, which was purified by flash silica gel column chromatography (hexanes-EtOAc = 1:1) to afford oxime **S46** (96.6 mg, 0.720 mmol, 33%, 3 steps from **S43**) as a mixture of isomer (*E*/*Z* = 1.3/1). An orange powder; mp: 154–157 ºC (hexanes-CH_2Cl_2); IR (film): 2925, 2222, 1577, 1558, 1472, 1456, 1281, 1058, 1029, 793, 742, 695 cm^{-1}; ^{1}H NMR (400 MHz, CDCl3): δ 8.77 (br s, 1H), 8.56 (d, 0.57H, *J* = 5.2 Hz), 8.50 (d, 0.43H, *J* = 5.0 Hz), 7.72 (d, 0.57H, *J* = 5.2 Hz), 7.56 (d, 0.43H, *J* = 8.0 Hz), 7.32–7.22 (m, 1H), 4.15 (s, 0.86H), 4.08 (s, 1.14H); ^{13}C NMR (100 MHz, $CDCl_3$) δ 164.2, 163.4, 152.5, 152.3, 150.9, 148.3, 136.1, 134.0, 129.9, 127.0, 124.3, 43.6, 43.3 (One signal is missing due to overlap.); HRMS (ESI) *m/z*: $[M+H]^+$ Calcd for $C_7H_7N_2O$ 135.0553; Found: 135.0558.

Oxime sulfonate 17s

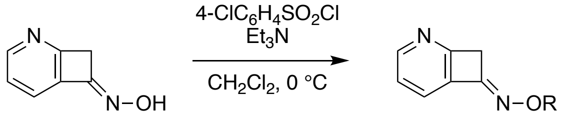

S46 **17s** (R = 4-ClC$_6$H$_4$SO$_2$)

To a solution of oxime **S46** (31.1 mg, 0.232 μmol) in CH_2Cl_2 (2.3 mL) were added Et_3N (50.0 μL, 0.358 μmol) and 4-$ClC_6H_4SO_2Cl$ (64.0 mg, 0.303 mmol) at 0 ºC. After stirring at 0 ºC for 2 h, the reaction was quenched with 1 M aqueous HCl, and the mixture was extracted with CH_2Cl_2 three times. The combined organic extracts were washed with brine, dried over anhydrous Na_2SO_4, and filtered. The organic solvents were removed under reduced pressure to give a crude material, which was purified by flash silica gel column chromatography (hexanes-EtOAc = 1:1) to afford oxime sulfonate **17s** (43.5 mg, 0.141 μmol, 61%) as a mixture of isomer (*E*/*Z* = 1:1.4). A brown powder;

mp: 124–127 ºC (hexanes-CH_2Cl_2); IR (film): 3092, 1574, 1378, 1192, 799 cm^{-1}; ^{1}H NMR (400 MHz, CDCl3): δ 8.66 (d, 0.58H, *J* = 4.8 Hz), 8.59 (d, 0.42H, *J* = 4.8 Hz), 7.99 (d, 1.16H, *J* = 8.8 Hz), 7.97 (d, 0.84H, *J* = 8.8 Hz), 7.81 (d, 0.58H, *J* = 8.0 Hz), 7.61–7.53 (m, 0.42H), 7.56 (d, 1.16H, *J* = 8.8 Hz), 7.55 (d, 0.84H, *J* = 8.8 Hz), 7.34 (t, 0.58H, *J* = 6.4 Hz), 7.29 (t, 0.42H, *J* = 5.6 Hz), 4.19 (s, 0.84H), 4.13 (s, 1.16H); ^{13}C NMR (100 MHz, CDCl3): δ 163.9, 163.2, 158.7, 156.1, 155.6, 155.2, 141.02, 140.99, 133.7, 133.4, 132.3, 131.3, 130.4, 130.3, 129.50, 129.47, 129.0, 125.00, 124.96, 44.9, 44.3 (One signal is missing due to overlap); HRMS (FAB) *m/z*: [M+H]$^+$ Calcd for $C_{13}H_{10}ClN_2O_3S$ 309.0095; Found: 309.0109.

Ring Expansion Reaction

General Procedure A:

Indole 21aa

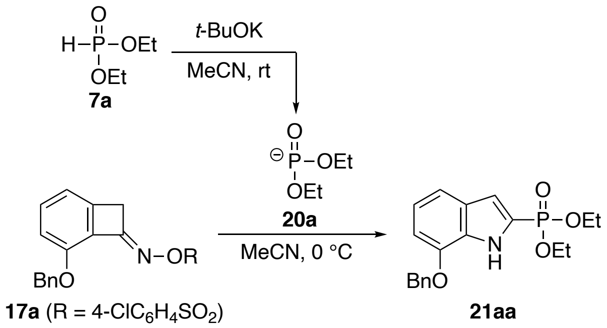

To a solution of diethyl phosphite **7a** (71.6 μL, 0.556 mmol) in MeCN (0.60 mL) was added *t*-BuOK (62.3 mg, 0.556 mmol) at room temperature. After stirring at room temperature for 5 min, the mixture was added to a solution of oxime sulfonate **17a**[17] (50.0 mg, 0.120 mmol) in MeCN (0.60 mL) at 0 °C, and the mixture was stirred for 15 min at 0 °C. The reaction was quenched with H_2O, and the mixture was roughly purified by flash silica gel column chromatography (hexanes-EtOAc = 1:1) to afford crude material. The crude material was further purified by preparative TLC (hexanes-EtOAc = 3:2) to afford indole **21aa** (36.0 mg, 0.100 mmol, 83%). A white crystalline solid; mp: 93–95 °C (CH_2Cl_2);IR (film): 3735, 3164, 2918, 2850, 1522, 1457, 1239, 1021, 974, 733 cm^{-1}; ^{1}H NMR (400 MHz, $CDCl_3$): δ 8.94 (1H, br s), 7.49 (2H, d, *J* = 6.8 Hz), 7.45–7.40 (3H, m), 7.29 (1H, d, *J* = 8.0 Hz), 7.09–7.05 (2H, m), 6.80 (1H, d, *J* = 7.6 Hz), 5.21 (2H, s), 4.20–4.26 (4H, m), 1.33 (6H, t, *J* = 6.8 Hz); ^{13}C NMR (100 MHz, $CDCl_3$): δ 145.6 (d, *J* = 2.3 Hz), 136.5, 129.2 (d, *J* = 2.2 Hz), 128.8 (d, *J* = 5.2 Hz), 128.6, 128.2, 127.7, 123.5 (d, *J* = 219.0 Hz), 121.1 (d, *J* = 1.5 Hz), 114.3, 112.4 (d, *J* = 17.5 Hz), 104.8, 70.2, 62.6 (d, *J* = 4.6 Hz), 16.2 (d, *J* = 6.9 Hz); ^{31}P NMR (240 MHz, $CDCl_3$): δ 10.5 (Chemical shift is reported in ppm downfield from H_3PO_4 (0 ppm) as the external standard); HRMS (ESI) *m/z*: [M+H]$^+$ Calcd for $C_{19}H_{23}NO_4P$ 360.1359; Found 360.1360.

Indole 21ab

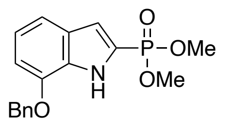

According to the general procedure A, oxime sulfonate **17a** (45.4 mg, 0.110 mmol) was converted to indole **21ab** (26.9 mg, 81.2 μmol, 74%). A white crystalline solid; mp: 158–159 °C (hexanes-CH_2Cl_2); IR (film): 3172, 2917, 1577, 1522, 1255, 1026, 773, 733 cm^{-1}; ^1H NMR (400 MHz, $CDCl_3$): δ 9.13 (1H, br s), 7.48 (2H, d, *J* = 6.8 Hz), 7.42 (2H, dd, *J* = 8.0, 6.8 Hz), 7.38 (1H, d, *J* = 8.0 Hz), 7.29, (1H, d, *J* = 8.0 Hz), 7.09–7.7.03 (2H, m), 6.79 (1H, d, *J* = 8.0 Hz), 5.20 (2H, s), 3.77 (6H, d, *J* = 11.6 Hz); ^{13}C NMR (100 MHz, $CDCl_3$): δ 145.7, 136.5, 129.4 (d, *J* = 12.4 Hz), 128.8 (d, *J* = 5.7 Hz), 128.6, 128.2, 127.8, 122.0 (d, *J* = 221.4 Hz), 121.3, 114.4, 112.9 (d, *J* = 17.3 Hz), 105.0, 70.3, 53.1 (d, *J* = 5.7 Hz); ^{31}P NMR (160 MHz, $CDCl_3$): δ 13.5 (Chemical shift is reported in ppm downfield from triphenyl phosphine (–6 ppm) as the external standard); HRMS (EI) *m/z*: $[M]^+$ Calcd for $C_{17}H_{18}NO_4P$ 331.0968; Found 331.0978.

Indole 21ac

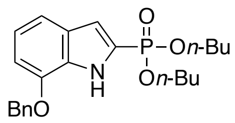

According to the general procedure A, oxime sulfonate **17a** (49.9 mg, 0.121 mmol) was converted to indole **21ac** (41.8 mg, 0.101 mmol, 83%). A white solid; IR (film): 3164, 2960, 2872, 1578, 1522, 1255, 1116, 1022, 772, 733 cm^{-1}; ^1H NMR (400 MHz, $CDCl_3$): δ 9.09 (1H, br s), 7.47 (2H, d, *J* = 7.2 Hz), 7.41 (2H, dd, *J* = 7.2, 7.2 Hz), 7.37 (1H, d, *J* = 7.2 Hz), 7.29 (1H, d, *J* = 8.0 Hz), 7.08–7.03 (2H, m), 6.78 (1H, d, *J* = 7.2 Hz), 5.20 (2H, s), 4.13–3.97 (4H, m), 1.69–1.64 (4H, m), 1.62–1.36 (4H, m), 0.90 (6H, t, *J* = 8.0 Hz); ^{13}C NMR (100 MHz, $CDCl_3$): δ 145.7, 136.6, 129.2 (d, *J* = 12.4 Hz), 129.0 (d, *J* = 15.6 Hz), 128.6, 128.2, 127.7, 123.7 (d, *J* = 220.5 Hz), 121.1, 114.4, 112.2 (d, *J* = 17.3 Hz), 104.9, 70.3 (d, *J* = 15.8 Hz), 66.4 (d, *J* = 6.5 Hz), 32.3 (d, *J* = 6.6 Hz), 18.7, 13.5; ^{31}P NMR (160 MHz, $CDCl_3$): δ 10.7 (Chemical shift is reported in ppm downfield from triphenyl phosphine (–6 ppm) as the external standard); HRMS (EI) *m/z*: $[M]^+$ Calcd for $C_{23}H_{30}NO_4P$ 415.1907; Found 415.1930.

General Procedure B:

Indole 21ad

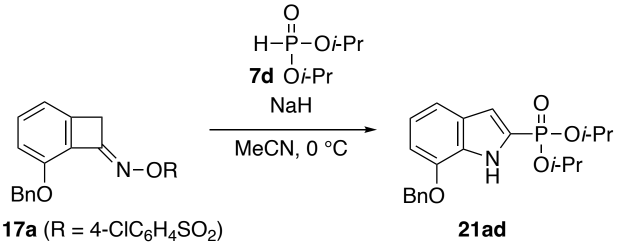

To a solution of oxime sulfonate **17a** (50.8 mg, 0.123 mmol) and di-*iso*-propyl phosphite **7d** (102 μL, 0.614 mmol) in MeCN (1.2 mL) was added NaH (24.6 mg, 0.615 mmol) at 0 °C. After stirring at 0 °C for 15 min, the reaction was quenched with H_2O, and the mixture was roughly purified by flash silica gel column chromatography (hexanes-EtOAc = 1:1) to afford a crude material. The crude material was further purified by preparative TLC (hexanes-EtOAc = 4:1) to afford indole **21ad** (38.5 mg, 0.0994 mmol, 81%). A white crystalline solid; mp: 160–161 ºC (CH_2Cl_2); IR (film): 3156, 2917, 2856, 1716, 1541, 1457, 1241, 1116, 987, 736 cm^{-1}; ^1H NMR (400 MHz, $CDCl_3$): δ 8.94 (1H, br s), 7.49 (2H, d, *J* = 6.8 Hz), 7.45–7.36 (3H, m), 7.29 (1H, d, *J* = 8.0 Hz), 7.08–7.02 (2H, m), 6.79 (1H, d, *J* = 7.6 Hz), 5.21 (2H, s), 4.72–4.64 (2H, m), 1.38 (6H, d, *J* = 6.0 Hz), 1.24 (6H, d, *J* = 6.0 Hz); ^{13}C NMR (100 MHz, $CDCl_3$): δ 145.6 (d, *J* = 1.7 Hz), 136.6, 129.1 (d, *J* = 2.5 Hz), 128.9, 128.6, 128.2, 127.7, 125.4 (d, *J* = 220.0 Hz), 121.0, 114.4, 111.8 (d, *J* = 17.3 Hz), 104.7, 71.4 (d, *J* = 4.9 Hz), 70.2, 24.0 (d, *J* = 4.1 Hz), 23.7 (d, *J* = 4.9 Hz); ^{31}P NMR (240 MHz, $CDCl_3$): δ 8.3 (Chemical shift is reported in ppm downfield from H_3PO_4 (0 ppm) as the external standard); HRMS (ESI) *m/z*: $[M+H]^+$ Calcd for $C_{21}H_{27}NO_4P$ 388.1672; Found 388.1659.

Indole 21ae

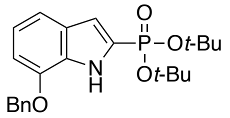

According to the general procedure B, oxime sulfonate **17a** (49.7 mg, 0.120 mmol) was converted to indole **21ae** (39.4 mg, 94.8 μmol, 78%). A yellow crystalline solid; mp: 109–110 °C (hexanes-CH_2Cl_2); IR (film): 3153, 2981, 1577, 1521, 1371, 1250, 1113, 986, 771 cm^{-1}; ^1H NMR (400 MHz, $CDCl_3$): δ 8.86 (1H, br s), 7.49 (2H, d, *J* = 7.2 Hz), 7.43 (2H, dd, *J* = 7.2, 7.2 Hz), 7.38 (1H, d, *J* = 7.2 Hz), 7.26–7.22 (1H, m), 7.04 (1H, dd, *J* = 8.0, 8.0 Hz), 6.98 (1H, dd, *J* = 1.6, 1.6 Hz), 6.77 (1H, d, *J* = 8.0 Hz), 5.21 (2H, s), 1.48 (18H, s); ^{13}C NMR (100 MHz, $CDCl_3$): δ 145.6 (d, *J* = 1.7 Hz), 136.8, 129.6 (d, *J* = 227.1 Hz), 129.2 (d, *J* = 15.6 Hz), 128.6, 128.5 (d, *J* = 12.3 Hz), 128.2, 127.8, 120.8, 114.5, 110.7 (d, *J* = 17.3 Hz), 104.5, 83.2 (d, *J* = 8.4 Hz), 70.3, 30.4 (d, *J* = 5.0 Hz); ^{31}P NMR (240 MHz, $CDCl_3$): δ 1.1 (Chemical shift is reported in ppm downfield from H_3PO_4 (0 ppm) as

the external standard); HRMS (EI) *m/z*: $[M]^+$ Calcd for $C_{23}H_{30}NO_4P$ 415.1907; Found 415.1921.

Indole 21af

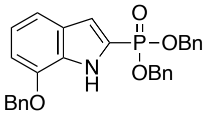

According to the modified general procedure A using additional amount of $KP(O)(OBn)_2$ (2.5 eq) to complete the reaction, oxime sulfonate **17a** (47.2 mg, 0.114 mmol) was converted to indole **21af** (45.1 mg, 0.0933 mmol, 82%). A white crystalline solid; mp: 84–85 °C (hexanes-CH_2Cl_2); IR (film): 3178, 2921, 2849, 1749, 1684, 1521, 1396, 1245, 995, 696 cm^{-1}; ^1H NMR (400 MHz, $CDCl_3$): δ 8.81 (1H, br s), 7.48–7.28 (16H, m), 7.08–7.04 (2H, m), 6.79 (1H, d, J = 8.0 Hz), 5.20 (2H, s), 5.16–5.03 (4H, m); ^{13}C NMR (100 MHz, $CDCl_3$): δ 145.6, 136.6, 135.8 (d, J = 6.6 Hz), 129.4 (d, J = 12.3 Hz), 128.8 (d, J = 16.5 Hz), 128.6, 128.5, 128.4, 128.2, 127.9, 127.7, 123.0 (d, J = 223.1 Hz), 121.2, 114.5, 113.1 (d, J = 17.3 Hz), 105.0, 70.3 (t, J = 7.4 Hz), 68.1(m); ^{31}P NMR (240 MHz, $CDCl_3$): δ 11.3 (Chemical shift is reported in ppm downfield from H_3PO_4 (0 ppm) as the external standard); HRMS (ESI) *m/z*: $[M+H]^+$ Calcd for $C_{29}H_{27}NO_4P$, 484.1672; Found 484.1683.

Indole 21ag

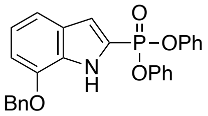

According to the general procedure B, oxime sulfonate **17a** (35.1 mg, 84.8 μmol) was converted to indole **21ag** (28.7 mg, 63.0 μmol, 75%). A yellow crystalline solid; mp: 120–121 °C (hexanes-CH_2Cl_2); IR (film): 3207, 2918, 2848, 1540, 1489, 1260, 1187, 945, 773 cm^{-1}; ^1H NMR (400 MHz, $CDCl_3$): δ 9.05 (1H, br s), 7.49–7.41 (5H, m), 7.32–7.28 (5H, m), 7.22–7.16 (7H, m), 7.08 (1H, dd, J = 8.0, 8.0 Hz), 6.81 (1H, d, J = 8.0 Hz), 5.20 (2H, s); ^{13}C NMR (150 MHz, $CDCl_3$): δ 150.1 (d, J = 6.9), 145.7 (d, J = 2.1), 136.4, 129.8, 129.6 (d, J = 12.9 Hz), 128.8, 128.7, 128.3, 127.9, 125.4, 121.9 (d, J = 228.6 Hz), 121.5, 120.6 (d, J = 5.0 Hz), 114.7, 114.2 (d, J = 18 Hz), 105.3, 70.4; ^{31}P NMR (240 MHz, $CDCl_3$): δ 3.1 (Chemical shift is reported in ppm downfield from triphenyl phosphine (–6 ppm) as the external standard); HRMS (EI) *m/z*: $[M]^+$ Calcd for $C_{27}H_{22}NO_4P$ 455.1281; Found 455.1279.

Indole 21ah

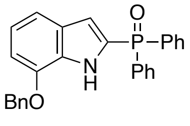

According to the general procedure B, oxime sulfonate **17a** (41.5 mg, 0.100 mmol) was converted to indole **21ah** (28.7 mg, 67.8 μmol, 68%). A white crystalline solid; mp: 221–222 °C (hexanes-CH_2Cl_2); IR (film): 3061, 2917, 2848, 1577, 1518, 1437, 1256, 1181, 1118, 727 cm^{-1}; ^{1}H NMR (400 MHz, $CDCl_3$): δ 9.23 (1H, br s), 7.74 (4H, dd, *J* = 12.8, 7.2 Hz), 7.57 (2H, dd, *J* = 8.4, 8.4 Hz), 7.50–7.44 (6H, m), 7.39 (2H, dd, *J* = 7.2, 7.2 Hz), 7.36 (1H, d, *J* = 7.2 Hz), 7.35 (1H, d, *J* = 8.0 Hz), 7.05 (1H, dd, *J* = 8.0, 8.0 Hz), 6.78 (1H, d, *J* = 8.0 Hz), 6.64 (1H, s), 5.19 (2H, s); ^{13}C NMR (100 MHz, $CDCl_3$): δ 145.7, 136.5, 132.2 (d, *J* = 3.1 Hz), 132.0 (d, *J* = 109.4 Hz), 131.8 (d, *J* = 10.7 Hz), 130.0 (d, *J* = 9.1 Hz), 128.9 (d, *J* = 12.2 Hz), 128.6, 128.5 (d, *J* = 9.1 Hz), 128.1, 127.6, 127.3, 121.1, 114.2, 113.4 (d, *J* = 15.2 Hz), 104.9, 70.2; ^{31}P NMR (160 MHz, $CDCl_3$): δ 22.0 (Chemical shift is reported in ppm downfield from H_3PO_4 (0 ppm) as the external standard); HRMS (EI) *m/z*: $[M]^+$ Calcd for $C_{27}H_{22}NO_2P$ 423.1383; Found 423.1378.

Indole 21ba

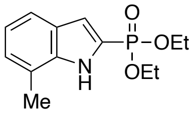

According to the modified general procedure A using DMSO instead of MeCN as solvent, oxime sulfonate **17b** (47.1 mg, 0.146 mmol) was converted to indole **21ba** (33.3 mg, 0.125 mmol, 86%). A white crystalline solid; mp: 106–107 °C (hexanes-CH_2Cl_2); IR (film): 3178, 2982, 1517, 1241, 1119, 1023, 747 cm^{-1}; ^{1}H NMR (400 MHz, $CDCl_3$): δ 8.67 (1H, br s), 7.54 (1H, d, *J* = 6.8 Hz), 7.11–7.07 (3H, m), 4.22–4.08 (4H, m), 2.53 (3H, s), 1.35 (6H, t, *J* = 7.2 Hz); ^{13}C NMR (100 MHz, $CDCl_3$): δ 138.2 (d, *J* = 12.1 Hz), 127.0 (d, *J* = 16.0 Hz), 124.7, 123.4 (d, *J* = 218.9 Hz), 121.9 (d, *J* = 1.5 Hz), 120.7, 119.3 (d, *J* = 1.5 Hz), 112.1 (d, *J* = 16.7Hz), 62.7 (d, *J* = 4.5 Hz), 16.9, 16.2 (d, *J* = 6.8 Hz); ^{31}P NMR (240 MHz, $CDCl_3$): δ 11.0 (Chemical shift is reported in ppm downfield from H_3PO_4 (0 ppm) as the external standard); HRMS (EI) *m/z*: $[M]^+$ Calcd for $C_{13}H_{18}NO_3P$ 267.1019; Found 267.1021. These data are identical with those reported.[18]

Indole 21ca

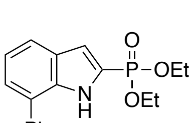

According to the general procedure A, oxime sulfonate **17c** (50.0 mg, 0.130 mmol) was converted to indole **21ca** (25.6 mg, 77.7 μmol, 60%). A white crystalline solid; mp: 99–100 °C (hexanes-CH_2Cl_2); IR (film): 3164, 2980, 1241, 1052, 1024, 972, 757 cm^{-1}; ^{1}H NMR (400 MHz, $CDCl_3$): δ 8.85 (1H, br s), 7.69 (1H, d, *J* = 7.6 Hz), 7.62 (2H, d, *J* = 7.2 Hz), 7.53 (2H, dd, *J* = 7.6,

7.6 Hz), 7.44 (1H, dd, J = 7.6, 7.6 Hz), 7.33 (1H, d, J = 7.2 Hz), 7.30–7.24 (1H, m), 7.14 (1H, dd, J = 1.6, 4.4 Hz), 4.23–4.06 (4H, m), 1.34 (6H, t, J = 7.2 Hz); ^{13}C NMR (150 MHz, $CDCl_3$): δ 138.3, 136.0 (d, J = 12.0 Hz), 129.3, 128.2, 128.0 (d, J = 15.0 Hz), 126.6, 125.8 (d, J = 193.5 Hz), 124.5, 123.6, 121.3, 121.2, 112.6 (d, J = 16.1 Hz), 62.8 (d, J = 5.1 Hz), 16.3 (d, J = 6.0 Hz); ^{31}P NMR (240 MHz, $CDCl_3$): δ 10.4 (Chemical shift is reported in ppm downfield from H_3PO_4 (0 ppm) as the external standard); HRMS (EI) *m/z*: $[M]^+$ Calcd for $C_{18}H_{20}NO_3P$ 329.1175; Found 329.1167.

Indole 21da

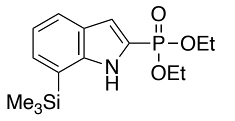

According to the general procedure A, oxime sulfonate **17d** (42.2 mg, 0.111 mmol) was converted to indole **21da** (26.1 mg, 80.2 μmol, 72%). A red oil; IR (film): 3212, 2980, 2905, 1505, 1252, 1114, 1030, 971, 838 cm^{-1}; ^{1}H NMR (400 MHz, $CDCl_3$): δ 8.61 (1H, br s), 7.71 (1H, d, J = 7.6 Hz), 7.43 (1H, d, J = 7.6 Hz), 7.18 (1H, dd, J = 7.6, 7.6 Hz), 7.10 (1H, dd, J = 2.0, 6.0 Hz), 4.22–4.09 (4H, m), 1.35 (6H, t, J = 6.8 Hz), 0.44 (9H, s); ^{13}C NMR (100 MHz, $CDCl_3$): δ 141.7 (d, J = 11.6 Hz), 130.9, 126.5 (d, J = 15.7 Hz), 123.9 (d, J = 219.7 Hz), 123.1, 122.2 (d, J = 1.6 Hz), 120.7, 112.3 (d, J = 16.4 Hz), 62.7 (d, J = 4.9 Hz), 16.2 (d, J = 6.6 Hz), –0.6; ^{31}P NMR (160 MHz, $CDCl_3$): δ 9.7 (Chemical shift is reported in ppm downfield from triphenyl phosphine (–6 ppm) as the external standard); HRMS (EI) *m/z*: $[M]^+$ Calcd for $C_{15}H_{24}NO_3PSi$ 325.1258; Found 325.1255.

Indole 21ea

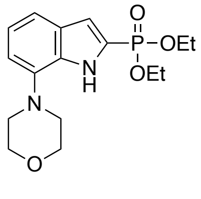

According to the general procedure A, oxime sulfonate **17e** (48.5 mg, 0.123 mmol) was converted to indole **21ea** (30.2 mg, 89.3 μmol, 73%). A brown crystalline solid; mp: 133–134 °C (hexanes-CH_2Cl_2); IR (film): 3179, 2919, 1581, 1241, 1052, 1019, 972, 896 cm^{-1}; ^{1}H NMR (400 MHz, $CDCl_3$): δ 8.92 (1H, br s), 7.42 (1H, d, J = 8.4 Hz), 7.13 (1H, dd, J = 8.4, 8.4 Hz), 7.06 (1H, dd, J = 2.0, 4.4 Hz), 6.95 (1H, J = 8.4 Hz), 4.24–4.09 (4H, m), 3.93 (4H, m), 3.11 (4H, m), 1.35 (6H, t, J = 6.8 Hz); ^{13}C NMR (100 MHz, $CDCl_3$): δ 138.7, 133.0 (d, J = 12.3 Hz), 128.7 (d, J = 15.8 Hz), 123.6 (d, J = 221.5 Hz), 121.4, 117.3, 113.1, 112.8 (d, J = 16.4 Hz), 67.3, 62.8 (d, J = 10.7 Hz), 52.0, 16.2 (d, J = 17.4 Hz); ^{31}P NMR (240 MHz, $CDCl_3$): δ 10.9 (Chemical shift is reported in ppm

downfield from H_3PO_4 (0 ppm) as the external standard); HRMS (EI) *m/z*: $[M]^+$ Calcd for $C_{16}H_{23}N_2O_4P$ 338.1390; Found 338.1384.

Indole 21fa

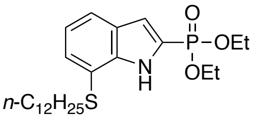

According to the modified general procedure A using DMSO/1,4-dioxane (3:1) as solvent instead of MeCN, oxime sulfonate **17f** (36.2 mg, 71.2 μmol) was converted to indole **21fa** (22.6 mg, 49.8 μmol, 70%); A white crystalline solid; mp: 51–52 ºC (CH_2Cl_2); IR (film): 3099, 2923, 2852, 1542, 1457, 1241, 1024, 967 cm^{-1}; ^{1}H NMR (400 MHz, $CDCl_3$): δ 9.00 (1H, br s), 7.61 (1H, d, *J* = 6.0 Hz), 7.40 (1H, d, *J* = 6.0 Hz), 7.16–7.10 (2H, m), 4.23–4.10 (4H, m), 2.89 (2H, t, *J* = 7.6 Hz), 1.64–1.60 (2H, m), 1.37–1.34 (8H, m), 1.31–1.24 (16H, m), 0.88 (3H, t, *J* = 6.8 Hz); ^{13}C NMR (150 MHz, $CDCl_3$): δ 139.2 (d, *J* = 12.0 Hz), 128.9, 127.3 (d, *J* = 15.0 Hz), 124.3 (d, *J* = 217.5 Hz), 121.4 (d, *J* = 12.9 Hz), 118.5 (d, *J* = 2.0 Hz), 113.0, 112.9, 62.8 (d, *J* = 5.0 Hz), 35.4, 31.9, 29.8, 29.64, 29.63, 29.58, 29.50, 29.3, 29.2, 28.7, 22.7, 16.3 (d, *J* = 7.1 Hz), 14.1; ^{31}P NMR (240 MHz, $CDCl_3$): δ 10.2 (Chemical shift is reported in ppm downfield from H_3PO_4 (0 ppm) as the external standard); HRMS (ESI) *m/z*: $[M+H]^+$ Calcd for $C_{24}H_{41}NO_3PS$ 454.2539; Found 454.2560.

Indole 21ga

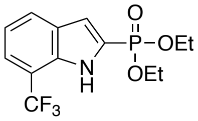

According to general procedure A, oxime sulfonate **17g** (49.8 mg, 0.133 mmol) was converted to indole **21ga** (12.3 mg, 38.3 μmol, 29%). A white crystalline solid; mp: 148–149 °C (hexanes-CH_2Cl_2); IR (film): 3178, 2983, 1395, 1228, 1113, 1023, 973, 828, 688 cm^{-1}; ^{1}H NMR (400 MHz, $CDCl_3$): δ 8.95 (1H, br s), 7.88 (1H, d, *J* = 7.6 Hz), 7.59 (1H, d, *J* = 7.6 Hz), 7.24 (1H, dd, *J* = 7.6, 7.6 Hz), 7.16 (1H, dd, *J* = 6.0, 2.0 Hz), 4.27–4.01 (4H, m), 1.37 (6H, t, *J* = 7.2 Hz) ; ^{13}C NMR (150 MHz, $CDCl_3$): δ 133.2 (*J* = 12.0, 2.0 Hz), 129.0, 126.3 (*J* = 312.3, 218.6 Hz), 126.0, 125.4, 122.2 (*J* = 5.0, 9.0 Hz), 120.0, 114.3 (33.0, 2.0 Hz), 112.3 (*J* = 15.9 Hz), 63.0 (5.0 Hz), 16.2 (7.1 Hz); ^{31}P NMR (160 MHz, $CDCl_3$): δ 9.20 (Chemical shift is reported in ppm downfield from triphenyl phosphine (–6 ppm) as the external standard); HRMS (ESI) *m/z:* [M+] Calcd for $C_{13}H_{15}F_3NO_3P$ 321.0736; Found 321.0743.

Indole 21ha

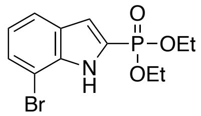

According to the general procedure A, oxime sulfonate **17h** (51.9 mg, 0.134 mmol) was converted to indole **21ha** (28.1 mg, 84.6 μmol, 63%). A white crystalline solid; mp: 149–150 °C (hexanes-CH_2Cl_2); IR (film): 3139, 2917, 2848, 1540, 1233, 1021, 974, 772 cm^{-1}; ^1H NMR (400 MHz, $CDCl_3$): δ 8.76 (1H, br s), 7.57 (1H, d, *J* = 8.0 Hz), 7.42 (1H, d, *J* = 8.0 Hz), 7.09 (1H, dd, *J* = 4.4, 2.4 Hz), 7.00 (1H, dd, *J* = 8.0, 8.0 Hz), 4.21–4.03 (4H, m), 1.31 (6H, t, *J* = 6.8 Hz); ^{13}C NMR (100 MHz, $CDCl_3$): δ 136.7 (d, *J* = 12.9 Hz), 128.5 (d, *J* = 15.3 Hz), 127.0, 125.2 (d, *J* = 218.9 Hz), 121.8, 121.1, 113.0 (d, *J* = 16.7 Hz), 105.1 (d, *J* = 2.2 Hz), 62.9 (d, *J* = 5.3 Hz), 16.2 (d, *J* = 6.9 Hz); ^{31}P NMR (160 MHz, $CDCl_3$): δ 9.6 (Chemical shift is reported in ppm downfield from triphenyl phosphine (–6 ppm) as the external standard); HRMS (EI) *m/z*: $[M]^+$ Calcd for $C_{12}H_{15}BrNO_3P$ 330.9967; Found 330.9982.

Indole 21ia

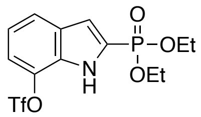

According to the general procedure A, oxime sulfonate **17i** (28.0 mg, 61.4 μmol) was converted to indole **21ia** (3.1 mg, 7.73 μmol, 13%). A white solid; IR (film): 3151, 3113, 3046, 2974, 1520, 1426, 1243, 1224, 1210, 1145, 1019, 985, 832, 736 cm^{-1}; ^1H NMR (400 MHz, $CDCl_3$): δ 9.51 (1H, br s), 7.69 (1H, d, *J* = 8.4 Hz), 7.28–7.26 (1H, m), 7.18 (1H, d, *J* = 8.0 Hz), 7.15 (1H, d, *J* = 3.6 Hz), 4.26–4.13 (4H, m), 1.36 (6H, t, *J* = 7.2 Hz); ^{13}C NMR (150 MHz, $CDCl_3$): δ 135.2 (d, *J* = 2.0 Hz), 131.2 (*J* = 15.9 Hz), 129.7 (*J* = 12.9 Hz), 126.9 (d, *J* = 218.6 Hz), 122.3 (d, *J* = 2.1 Hz), 120.7, 118.7 (d, *J* = 319.4 Hz), 116.3, 112.7 (d, *J* = 15.9 Hz), 63.0 (d, *J* = 5.0 Hz), 16.2 (d, *J* = 6.0 Hz); ^{31}P NMR (240 MHz, $CDCl_3$): δ 8.5 (Chemical shift is reported in ppm downfield from H_3PO_4 (0 ppm) as the external standard); HRMS (ESI) *m/z*: $[M+Na]^+$ Calcd for $C_{13}H_{16}F_3NO_6PS$ 402.0383; Found 402.0394.

Indole 21ja

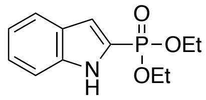

According to the general procedure A, oxime sulfonate **17j** (43.3 mg, 0.141 mmol) was converted to indole **21ja** (24.3 mg, 96.0 μmol, 68%). A red crystalline solid; mp: 77–78 °C (hexanes-CH_2Cl_2); IR (film): 3179, 2919, 2850, 1581, 1520, 1241, 1116, 1019, 972, 896 cm^{-1}; ^1H NMR (400 MHz,

$CDCl_3$): δ 9.55 (1H, br s), 7.67 (1H, d, J = 8.0 Hz), 7.47 (1H, d, J = 8.0 Hz), 7.29 (1H, dd, J = 8.0 Hz), 7.14 (1H, dd, J = 8.0 Hz), 7.04 (1H, br s), 4.22–4.06 (4H, m), 1.33 (6H, t, J = 6.4 Hz); ^{13}C NMR (100 MHz, $CDCl_3$): δ 138.4 (d, J = 13.0 Hz), 127.4 (d, J = 15.9 Hz), 124.4, 123.6 (d, J = 218.9 Hz), 121.7, 120.4, 112.0 (d, J = 2.0 Hz), 111.5 (d, J = 17.0 Hz), 62.8 (d, J = 5.0 Hz), 16.3 (d, J = 7.0 Hz); ^{31}P NMR (160 MHz, $CDCl_3$): δ 10.7 (Chemical shift is reported in ppm downfield from triphenyl phosphine (–6 ppm) as the external standard); HRMS (EI) *m/z*: $[M]^+$ Calcd for $C_{12}H_{16}NO_3P$ 253.0862; Found 253.0864. These data are identical with those reported. [18]

Indole 21ka

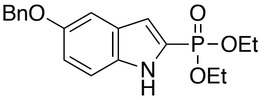

According to the general procedure A, oxime sulfonate **17k** (35.3 mg, 85.3 μmol) was converted to indole **21ka** (11.9 mg, 33.1 μmol, 39%). A yellow crystalline solid; mp: 147–148 °C (hexanes-CH_2Cl_2); IR (film): 3193, 2982, 1509, 1224, 1171, 1023, 801 cm^{-1}; ^{1}H NMR (400 MHz, $CDCl_3$): δ 8.88 (1H, br s), 7.47 (2H, d, J = 7.6 Hz), 7.41–7.33 (4H, m), 7.16 (1H, s), 7.07 (1H, dd, J = 8.8, 1.6 Hz), 6.97 (1H, s), 5.11 (2H, s), 4.22–4.05 (4H, m), 1.32 (6H, t, J = 6.8 Hz); ^{13}C NMR (100 MHz, $CDCl_3$): δ 153.8, 137.4, 133.7 (d, J = 12.9 Hz), 128.5, 127.8, 127.5, 124.2, (d, J = 218.9 Hz), 116.7, 112.9, 111.3 111.2, 103.7, 70.7, 62.8, 16.3 (d, J = 17.6 Hz); ^{31}P NMR (240 MHz, $CDCl_3$): δ 10.9 (Chemical shift is reported in ppm downfield from H_3PO_4 (0 ppm) as the external standard); HRMS (EI) *m/z*: $[M]^+$ Calcd for $C_{19}H_{22}NO_4P$ 359.1281; Found 359.1278. These data are identical with those reported. [18]

Indole 21la

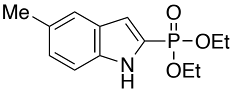

According to the general procedure A, oxime sulfonate **17l** (40.0 mg, 0.124 mmol) was converted to indole **21la** (12.7 mg, 47.5 μmol, 38%). A yellow crystalline solid; mp: 74–75 ºC (CH_2Cl_2); IR (film): 3178, 2983, 1395, 1228, 1113, 1023, 973, 828, 688 cm^{-1}; ^{1}H NMR (400 MHz, $CDCl_3$): δ 8.77 (1H, br s), 7.47 (1H, s), 7.34 (1H, d, J = 8.4 Hz), 7.15 (1H, dd, J = 8.4 Hz), 7.00–6.98 (1H, m), 4.20–4.07 (4H, m), 2.45 (3H, s), 1.34 (6H, t, J = 7.2 Hz); ^{13}C NMR (150 MHz, $CDCl_3$): δ 136.5 (d, J = 12.0 Hz), 129.9, 127.8 (J = 15.0 Hz), 126.5, 122.9 (d, J = 210.0 Hz), 121.1, 111.7, 111.3 (d, J = 17.0 Hz), 62.7 (d, J = 5.0 Hz), 21.4, 16.2 (d, J = 7.1 Hz); ^{31}P NMR (240 MHz, $CDCl_3$): δ 10.8 (Chemical shift is reported in ppm downfield from H_3PO_4 (0 ppm) as the external standard); HRMS (ESI) *m/z*: $[M+H]^+$ Calcd for $C_{13}H_{19}NO_3P$ 268.1103; Found 268.1097. These data are identical with those reported. [18]

Indole 21ma

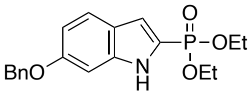

According to the general procedure A, oxime sulfonate **17m** (39.1 mg, 96.6 μmol) was converted to indole **21ma** (17.3 mg, 48.1 mmol, 50%). A yellow crystalline solid; mp: 151–152 °C (hexanes-CH_2Cl_2); IR (film): 3207, 2982, 1625, 1506, 1235, 11688, 1023, 976 cm^{-1}; ^{1}H NMR (400 MHz, $CDCl_3$): δ 8.60 (1H, br s), 7.56 (1H, d, *J* = 9.2 Hz), 7.46 (2H, d, *J* = 7.2 Hz), 7.40 (2H, dd, *J* = 7.2 Hz, 7.2 Hz), 7.34 (1H, d, *J* = 7.2 Hz), 7.01 (1H, d, *J* = 2.4 Hz), 6.93–6.92 (2H, m), 5.12 (2H, s), 4.19–4.06 (4H, m), 1.34 (6H, t, *J* = 7.2 Hz); ^{13}C NMR (100 MHz, $CDCl_3$): δ 157.4, 139.2 (d, *J* = 13.2 Hz), 137.1, 128.5, 127.9, 127.4, 122.4, 122.3 (d, *J* = 223.1 Hz), 122.1 (d, *J* = 16.5 Hz), 112.5, 112.0 (d, *J* = 7.2 Hz), 95.6, 70.4, 62.6 (d, *J* = 4.9 Hz), 16.2 (d, *J* = 6.6 Hz); ^{31}P NMR (160 MHz, $CDCl_3$): δ 10.9 (Chemical shift is reported in ppm downfield from triphenyl phosphine (–6 ppm) as the external standard); HRMS (EI) *m/z*: [M]$^+$ Calcd for $C_{19}H_{22}NO_4P$ 359.1281; Found 359.1276.

Indole 21na

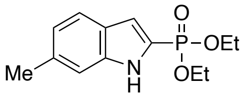

According to the general procedure A, oxime sulfonate **17n** (36.7 mg, 0.114 mmol) was converted to indole **21na** (21.0 mg, 78.5 μmol, 69%). A yellow crystalline solid; mp: 84–85 ºC (CH_2Cl_2); IR (film): 3211, 3085, 2982, 1627, 1497, 1392, 1297, 1246, 1164, 1108, 1023, 974, 822, 768 cm^{-1}; ^{1}H NMR (400 MHz, $CDCl_3$): δ 8.79 (1H, br s), 7.56 (1H, d, *J* = 8.0 Hz), 7.23 (1H, s), 7.03–6.99 (2H, m), 4.20–4.07 (4H, m), 2.48 (3H, s), 1.34 (6H, t, *J* = 7.2 Hz); ^{13}C NMR (150 MHz, $CDCl_3$): δ 138.8 (d, *J* = 12.0 Hz), 134.6, 125.3 (*J* = 15.0 Hz), 122.8 (d, *J* = 218.4 Hz), 122.6, 121.3 (d, *J* = 2.1 Hz), 111.8 (d, *J* = 5.0 Hz), 111.6, 62.7 (d, *J* = 5.0 Hz), 21.8, 16.2 (d, *J* = 7.1 Hz); ^{31}P NMR (240 MHz, $CDCl_3$): δ 10.9 (Chemical shift is reported in ppm downfield from H_3PO_4 (0 ppm) as the external standard); HRMS (ESI) *m/z*: [M+H]$^+$ Calcd for $C_{13}H_{19}NO_3P$ 268.1103; Found 268.1097.

Indole 21oa

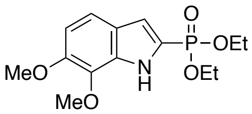

According to the modified general procedure A using DMSO was used instead of MeCN, oxime sulfonate **17o** (38.0 mg, 0.103 mmol) was converted to indole **21oa** (26.4 mg, 84.3 μmol, 82%); A white crystalline solid; mp: 94–95 °C (hexanes-CH_2Cl_2); IR (film): 3178, 2984, 1627, 1505, 1248, 1021, 971 cm^{-1}; ^{1}H NMR (400 MHz, $CDCl_3$): δ 8.71 (1H, br s), 7.34 (1H, d, *J* = 8.4 Hz), 7.02 (1H,

dd, *J* = 4.4, 2.0 Hz), 6.92 (1H, d, *J* = 8.4 Hz), 4.22–4.01 (4H, m), 4.01 (3H, s), 3.94 (3H, s), 1.35 (6H, t, *J* = 7.2 Hz); ^{13}C NMR (100 MHz, $CDCl_3$): δ 148.6, 134.4, 133.1 (d, *J* = 12.3 Hz), 124.0, 123.7 (d, *J* = 222.2 Hz), 116.7, 112.6 (d, *J* = 17.2 Hz), 110.0, 62.7, 60.9, 57.3, 16.2 (d, *J* = 6.6 Hz); ^{31}P NMR (240 MHz, $CDCl_3$): δ 10.6 (Chemical shift is reported in ppm downfield from H_3PO_4 (0 ppm) as the external standard); HRMS (EI) *m/z*: $[M]^+$ Calcd for $C_{14}H_{20}NO_5P$ 313.1074; Found 313.1067.

Indole 21pa

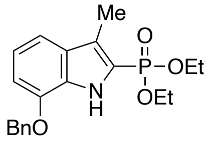

According to the general procedure A, oxime sulfonate **17p** (50.0 mg, 0.117 mmol) was converted to indole **21pa** (39.0 mg, 0.104 mmol, 89%); A yellow crystalline solid; mp: 74–75 °C (hexanes-CH_2Cl_2); IR (film): 3177, 2917, 1577, 1243, 1080, 1022, 967, 780 cm^{-1}; ^{1}H NMR (400 MHz, $CDCl_3$): δ 8.78 (1H, br s), 7.47 (2H, d, *J* = 7.2 Hz), 7.42 (2H, dd, *J* = 7.2, 7.2 Hz), 7.39 (1H, d, *J* = 7.2 Hz), 7.26–7.24 (1H, m), 7.06 (1H, dd, *J* = 8.0, 8.0 Hz), 6.79 (1H, d, *J* = 8.0 Hz), 5.20 (2H, s), 4.19–4.02 (4H, m), 2.49 (3H, s), 1.32 (6H, t, *J* = 7.2 Hz); ^{13}C NMR (150 MHz, $CDCl_3$): δ 145.5, 136.6, 129.9(d, *J* = 15.8 Hz) , 128.6 (d, *J* = 11.6 Hz), 128.5, 128.2, 127.7, 121.8 (d, *J* = 13.8 Hz), 120.4, 119.7 (d, *J* = 216.3 Hz), 112.7, 104.9, 70.2, 62.2 (d, *J* = 4.5 Hz), 16.2 (d, *J* = 7.2 Hz), 9.7; ^{31}P NMR (160 MHz, $CDCl_3$): δ 12.1 (Chemical shift is reported in ppm downfield from triphenyl phosphine (–6 ppm) as the external standard); HRMS (EI) *m/z*: $[M]^+$ Calcd for $C_{20}H_{24}NO_4P$ 373.1437; Found 373.1426.

Indole21qa

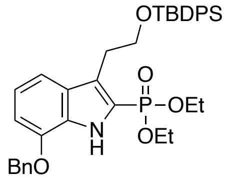

According to the general procedure A, oxime sulfonate **17q** (58.4 mg, 0.0836 mmol) was converted to indole **21qa** (46.8 mg, 0.0729 mmol, 87%); A white solid; IR (film): 3175, 3069, 2930, 2857, 1577, 1472, 1389, 1244, 1086, 1021, 972, 784, 737, 702 cm^{-1}; ^{1}H NMR (400 MHz, $CDCl_3$): δ 8.81 (1H, br s), 7.65–7.62 (4H, m), 7.47–7.33 (11H, m), 6.99 (1H, d, *J* = 8.0 Hz), 6.93 (1H, t, *J* = 7.8 Hz), 6.73 (1H, d, *J* = 7.6 Hz), 5.17 (2H, s), 4.13–4.03 (2H, m), 3.98–3.85 (4H, m), 3.24 (2H, t, *J* = 7.6 Hz), 1.23 (6H, t, *J* = 7.0 Hz), 1.03 (9H, s); ^{13}C NMR (100 MHz, $CDCl_3$): δ 145.5, 136.6, 135.6, 133.9, 129.8 (d, *J* = 16.4 Hz), 129.5, 128.6, 128.4, 128.2, 127.7, 127.6, 122.6 (d, *J* = 14.8 Hz), 120.4, 120.4 (d, *J* = 218.1 Hz), 113.3, 104.9, 70.2 (d, *J* = 5.0 Hz), 64.6, 62.3 (d, *J* = 5.0 Hz), 28.6, 26.9, 19.1,

16.2 (d, $J = 6.6$ Hz); ^{31}P NMR (240 MHz, $CDCl_3$): δ 11.8 (Chemical shift is reported in ppm downfield from H_3PO_4 (0 ppm) as the external standard); HRMS (ESI) *m/z*: $[M+H]^+$ Calcd for $C_{37}H_{45}NO_5PSi$ 642.2799; Found 642.2808.

Indole 21ra

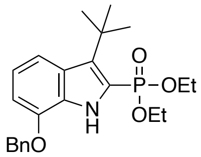

According to the modified general procedure A using the additional amount of nucleophile (2.5 eq.) to complete consumption of the substrate, oxime sulfonate **17r** (43.6 mg, 0.093 mmol) was converted to indole **21ra** (39.0 mg, 0.0645 mmol, 69%); A yellow oil; IR (film): 3179, 2928, 2894, 1698, 1528, 1456, 1365, 1251, 1164, 996 cm^{-1}; ^{1}H NMR (400 MHz, $CDCl_3$): δ 9.15 (1H, br s), 7.55 (1H, d, $J = 8.8$ Hz), 7.47 (2H, d, $J = 7.2$ Hz), 7.42–7.35 (2H, m), 7.36 (1H, d, $J = 6.8$ Hz), 6.99 (1H, t, $J = 8.2$ Hz), 6.72 (1H, d, $J = 7.6$ Hz), 5.20 (2H, s), 4.21–4.03 (4H, m), 1.59 (9H, s), 1.35 (6H, t, $J = 7.2$ Hz); ^{13}C NMR (100 MHz, $CDCl_3$): δ 145.7, 136.7, 134.3 (d, $J = 17.3$ Hz) , 129.0 (d, $J = 16.5$ Hz), 128.8, 128.6, 128.1, 127.6, 119.7, 117.8 (d, $J = 214.8$ Hz), 116.2, 103.9, 70.3 (d, $J = 5.7$ Hz), 62.4, 33.5, 31.8, 16.1 (d, $J = 6.6$ Hz); ^{31}P NMR (160 MHz, $CDCl_3$): δ 14.3 (Chemical shift is reported in ppm downfield from triphenyl phosphine (–6 ppm) as the external standard); HRMS (EI) *m/z*: $[M]^+$ Calcd for $C_{23}H_{30}NO_4P$ 415.1907; Found 415.1901.

4-Aza Indole 21sa

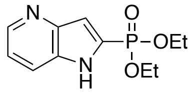

According to the general procedure A, oxime sulfonate **17s** (31.0 mg, 0.100 mmol) was converted to indole **21sa** (7.5 mg, 29.5 μmol, 30%). A yellow oil; IR (film): 3410, 3245, 2983, 1652, 1404, 1240, 1163, 1102, 965, 795, 615 cm^{-1}; ^{1}H NMR (600 MHz, $CDCl_3$): δ 10.75 (1H, br s), 8.56 (1H, dd, $J =$ 4.8, 1.2 Hz), 7.84 (1H, d, $J = 8.4$ Hz), 7.24–7.21 (2H, m), 4.24–4.15 (4H, m), 1.36 (6H, t, $J = 7.2$ Hz); ^{13}C NMR (150 MHz, $CDCl_3$): δ 145.1 (d, $J = 17.3$ Hz), 144.8, 131.4 (d, $J = 11.4$ Hz), 127.1 (d, $J =$ 219.2 Hz), 120.1, 119.0, 111.5 (d, $J = 15.8$ Hz), 63.3 (d, $J = 5.7$ Hz), 16.3 (d, $J = 7.2$ Hz); ^{31}P NMR (160 MHz, $CDCl_3$): δ 9.9 (Chemical shift is reported in ppm downfield from H_3PO_4 (0 ppm) as the external standard); HRMS (ESI) *m/z*: $[M+H]^+$ Calcd for $C_{11}H_{16}N_2O_3P$ 255.0893; Found 255.0919.

Phosphonic Acid 22

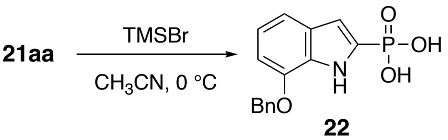

22

To a solution of indole **21aa** (93.8 mg, 0.261 mmol) in MeCN (1.7 ml) was added TMSBr (0.36 ml, 2.61 mmol) at room temperature. After stirring at room temperature for 1 h, the solvents were removed under reduced pressure. To the residue were added Et_2O (1.7 ml) and H_2O (0.1 ml), and the mixture was stirred for 30 min at room temperature. The solvents were removed under reduced pressure to give a crude material, which was washed with EtOAc to afford phosphonic acid **22** (78.8 mg, 0.260 mmol, quant.) A white crystalline solid; mp: 127 °C, decomposition (hexane-MeOH); IR (film): 3394, 2925, 1716, 1653, 1558, 1522, 1457, 1220, 1118, 772 cm^{-1}; ^{1}H NMR (400 MHz, DMSO): δ 11.42 (1H, br s), 10.44 (2H, br s),7.55 (2H, d, J = 7.2 Hz), 7.31 (2H, dd, J = 7.2, 7.2 Hz), 7.23 (1H, dd, J = 7.2, 7.2 Hz), 7.12 (1H, d, J = 8.0 Hz), 6.87 (1H, dd, J = 8.0, 8.0 Hz), 6.80–6.79 (1H, m), 6.73 (1H, d, J = 8.0 Hz), 5.18 (2H, s); ^{13}C NMR (100 MHz, DMSO): δ 145.5 (d, J = 1.7 Hz), 137.4, 130.7 (d, J = 210.7 Hz), 128.9 (d, J = 12.4 Hz), 128.6 (d, J = 14.8 Hz), 128.3, 127.6, 127.4, 120.1, 113.9, 110.0 (d, J = 17.3 Hz), 104.6, 69.3; ^{31}P NMR (160 MHz, DMSO): δ 4.4 (Chemical shift is reported in ppm downfield from H_3PO_4 (0 ppm) as the external standard); HRMS (ESI) *m/z*: $[M+H]^+$ Calcd for $C_{15}H_{15}NO_4P$ 304.0733; Found 304.0759.

2-Phosphino Indole 23

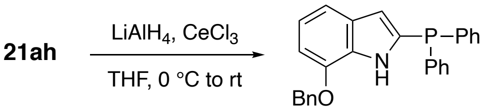

23

To a solution of indole **21ah** (10.6 mg, 25.0 mmol) and $CeCl_3$ (75.0 mmol) in THF (490 μL) was added LAH (5.4 mg, 0.150 mmol) at 0 °C. After stirring at room temperature for 16 h, the reaction was quenched with H_2O, 1 M HCl aq. and the mixture was extracted with EtOAc three times. The combined organic extracts were washed with brine, dried over anhydrous sodium sulfate, and filtered. The organic solvents were removed under reduced pressure to give a crude material, which was purified by flash silica gel column chromatography (hexane-EtOAc = 2:1) to afford indole **23** (9.8 mg, 0.0241 mmol, 96%); A white solid; IR (film): 3618, 3189, 3066, 1683, 1577, 1434, 1393, 1316, 1251, 1100, 805, 731, 695 cm^{-1}; ^{1}H NMR (600 MHz, $CDCl_3$): δ 8.29 (1H, br s), 7.41–7.32 (15H, m), 7.20 (1H, d, J = 7.8 Hz), 6.99 (1H, t, J = 8.4 Hz), 6.70 (1H, d, J = 8.4 Hz), 6.61 (1H, t, J = 2.4 Hz), 5.16 (2H, s); ^{13}C NMR (150 MHz, $CDCl_3$): δ 146.1, 136.9, 136.1 (d, J = 7.2 Hz), 133.3 (d, J = 18.6 Hz), 132.6 (d, J = 12.9 Hz), 130.0 (d, J = 7.1 Hz), 129.7 (d, J = 4.4 Hz), 129.0, 128.7, 128.6 (d, J = 7.1 Hz), 128.1, 127.6, 120.3, 113.7, 113.0 (d, J = 18.8 Hz), 103.9, 70.3; ^{31}P NMR (160 MHz, $CDCl_3$):

δ –24.0 (Chemical shift is reported in ppm downfield from H_3PO_4 (0 ppm) as the external standard); HRMS (ESI) *m/z*: $[M+H]^+$ Calcd for $C_{27}H_{23}NOP$ 408.1512; Found 408.1503.

2-Phosphino Indole 23

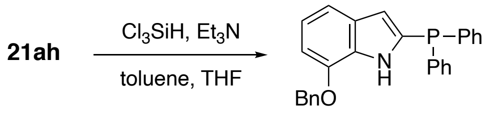

To a solution of indole **21ah** (9.4 mg, 22.2 μmol) and Et_3N (15.5 μL, 0.111 mmol) in toluene (450 μL), THF (200 μL) was added Cl_3SiH (6.7 μL, 22.3 μmol) at 60 °C. After stirring at room temperature for 16 h, the reaction was quenched with H_2O and the mixture was extracted with EtOAc three times. The combined organic extracts were washed with brine, dried over anhydrous sodium sulfate, and filtered. The organic solvents were removed under reduced pressure to give a crude material, which was purified by flash silica gel column chromatography (hexane-EtOAc = 2:1) to afford indole **23** (7.0 mg, 17.2 μmol, 77%).

Application to Synthesis of Duocarmycin SA Phosphate Analog

Benzocyclobutenone 28

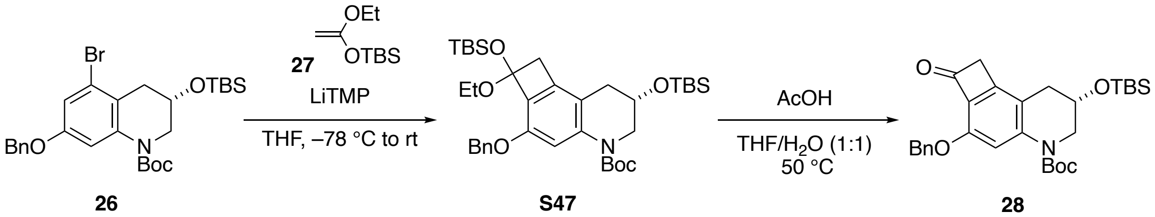

To a solution of aryl bromide **26**[19] (571 mg, 1.04 mmol) and ketene silyl acetal **27** (1.23 mL, 5.20 mmol) in THF (2.6 ml) was added LiTMP (6.24 mL, 0.50 M in *n*-hexane-THF, 3.12 mmol) over 10 min at –78 °C. After stirring at room temperature for 45 min, the reaction was quenched with saturated $NaHCO_3$ aq., and the mixture was extracted with EtOAc three times. The combined organic extracts were washed with brine, dried over anhydrous sodium sulfate, and filtered. The organic solvents were removed under reduced pressure to give a crude **S47**, which was used in the next reaction without further purification.

To a solution of the crude **S47** in THF (1.0 mL) and H_2O (1.0 mL) was added AcOH (3.0 mL). After stirring at 50 °C for 5 h, the reaction was quenched with saturated aqueous $NaHCO_3$, and the mixture was extracted with EtOAc three times. The combined organic extracts were washed with saturated aqueous $NaHCO_3$ and brine, dried over anhydrous sodium sulfate, and filtered. The organic solvents were removed under reduced pressure to give a crude material, which was purified by flash

silica gel column chromatography (hexane-EtOAc = 16:1) to afford benzocyclobutenone **28** (264 mg, 0.519 mmol, 50%, 2 steps from **26**); A colorless oil; $[\alpha]_D^{18}$ +30.9 (*c* 0.440 , $CHCl_3$); IR (film): 2929, 1757, 1709, 1622, 1562, 1486, 1367, 1252, 1151, 1100, 838 cm^{-1}; ^1H NMR (400 MHz, $CDCl_3$): δ 7.43 (2H, d, *J* = 7.2 Hz), 7.37–7.30 (4H, m), 5.41 (2H, s), 4.16–4.10 (1H, m), 3.91 (1H, dd, *J* = 13.2, 3.2 Hz), 3.76 (2H, s), 3.49 (1H, dd, *J* = 13.2, 8.0 Hz), 2.93 (1H, dd, *J* = 16.4, 5.6 Hz), 2.62 (1H, dd, *J* = 16.4, 6.8 Hz), 1.52 (9H, s), 0.90 (9H,s), 0.10 (6H, s); ^{13}C NMR (100 MHz, $CDCl_3$): δ 182.6, 153.0, 150.6, 148.9, 146.6, 136.6, 128.2, 127.7, 127.5, 127.4, 114.9, 111.7, 81.7, 73.5, 64.6, 50.5, 49.0, 33.1, 28.0, 25.6, 18.0, –4.9, –5.0; HRMS (EI) *m/z*: $[M]^+$ Calcd for $C_{29}H_{39}NO_5Si$ 509.2592; Found 509.2590.

Oxime S48

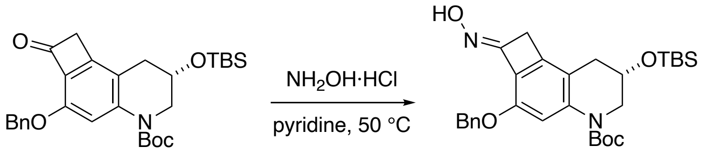

28 **S48**

To a solution of benzocyclobutenone **28** (264 mg, 0.519 mmol) in pyridine (2.6 mL) was added $NH_2OH{\cdot}HCl$ (144 mg, 2.07 mmol) at 50 °C. After stirring at 50 °C for 7 h, the reaction mixture was diluted with EtOAc, and the mixture was washed with 1 M HCl aq. and brine, dried over anhydrous sodium sulfate, and filtered. The organic solvents were removed under reduced pressure to give a crude material, which was purified by flash silica gel column chromatography (hexane-EtOAc = 10:1) to afford oxime **S48** (171 mg, 0.326 mmol, 63%); A white solid; $[\alpha]_D^{18}$ +43.796 (*c* 1.00, $CHCl_3$); IR (film): 3359, 2928, 1698, 1615, 1488, 1365, 1220, 1100 cm^{-1}; ^1H NMR (400 MHz, $CDCl_3$): δ 7.43 (2H, d, *J* = 7.6 Hz), 7.35 (2H, dd, *J* = 7.6 Hz), 7.30 (1H, d, *J* = 7.6 Hz), 7.23 (1H, d, *J* = 7.6 Hz), 6.69 (1H, s), 5.36 (2H, s), 4.13–4.06 (1H, m), 3.95 (1H, dd, *J* = 12.8, 2.8 Hz), 3.72 (2 H, s), 3.38 (1H, dd, *J* = 12.8, 8.0 Hz), 2.88 (1H, dd, *J* = 16.8, 6.0 Hz), 2.55 (1H, dd, *J* = 16.8, 7.2 Hz), 1.51 (9H, s), 0.90 (9H, s), 0.12 (6H, s); ^{13}C NMR (100 MHz, $CDCl_3$): δ 153.4, 151.5, 149.9, 142.9, 142.4, 137.1, 128.2, 127.6, 127.4, 120.7, 115.5, 112.1, 81.3, 71.8, 65.0, 50.4, 36.3, 32.7, 28.2, 25.8, 18.1, –4.9, –4.8; HRMS (EI) *m/z*: $[M]^+$ Calcd for $C_{29}H_{40}N_2O_5Si$ 524.2701; Found 524.2705.

Oxime sulfonate 17t

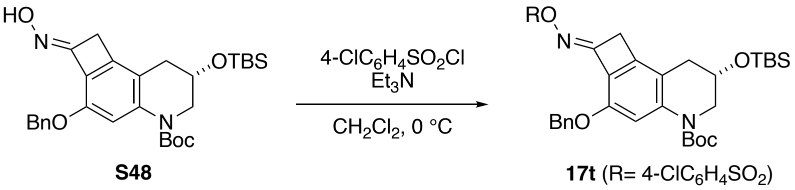

S48 **17t** (R= 4-$ClC_6H_4SO_2$)

To a solution of oxime **S48** (171 mg, 0.326 mmol) in CH_2Cl_2 (3.3 mL) was added 4-$ClC_6H_4SO_2Cl$ (172 mg, 0.815 mmol) and Et_3N (0.273 mL, 1.96 mmol) at 0 °C. After stirring at 0 °C for 1 h, the

reaction was quenched with 1 M HCl aq., and the mixture was extracted with CH_2Cl_2 three times. The combined organic extracts were washed with brine, dried over anhydrous sodium sulfate, and filtered. The organic solvents were removed under reduced pressure to give a crude material, which was purified by flash silica gel column chromatography (hexane-EtOAc = 4:1) to afford oxime sulfonate **17t** (213 mg, 0.304 mmol, 93%); A white amorphous; $[\alpha]_D^{18}$ –6.191 (*c* 0.680, $CHCl_3$); IR (film): 2917, 1715, 1488, 1372, 1220, 1191, 1153 cm^{-1}; ^1H NMR (400 MHz, $CDCl_3$): δ 7.90 (2H, d, *J* = 8.8 Hz), 7.42 (2H, d, *J* = 8.8 Hz), 7.36–7.29 (6H, m), 5.23 (2H, s), 4.11–4.06 (1H, m), 3.86 (1H, dd, *J* = 12.8, 3.2 Hz), 3.77 (2H, s), 3.43 (1H, dd, *J* = 12.8, 8.0 Hz), 2.82 (1H, dd, *J* = 16.8, 5.2 Hz), 2.51 (1H, dd, *J* = 16.8, 6.4 Hz), 1.50 (9H, s), 0.88 (9H, s), 0.10 (6H, s); ^{13}C NMR (100 MHz, $CDCl_3$): δ 158.9, 153.0, 151.1, 145.1, 142.8, 140.4, 136.5, 133.9, 130.2, 129.1, 128.3, 127.9, 127.3, 116.8, 115.3, 112.5, 81.6, 71.8, 64.4, 50.4, 37.8, 32.4, 28.1, 25.7, 18.0, –4.9, –5.0; HRMS (ESI) *m/z*: $[M+H]^+$ Calcd for $C_{35}H_{44}ClN_2O_7SSi$ 699.2322; Found 699.2308.

Indole 21ta

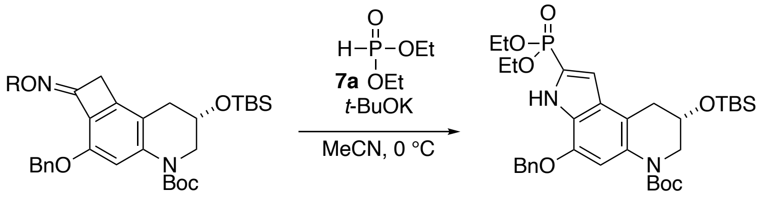

To a solution of diethyl phosphite **7a** (63.8 μL, 0.494 mmol) in MeCN (0.5 mL) was added *t*-BuOK (55.4 mg, 0.494 mmol) at room temperature. After stirring at room temperature for 5 min, the mixture was added to a solution of oxime sulfonate **17t** (69.1 mg, 0.0988 mmol) in MeCN (0.50 ml) at 0 °C. After the mixture was stirred at 0 °C for 15 min, the reaction was quenched with H_2O, and the mixture was extracted with EtOAc three times. The combined organic extracts were washed with brine, dried over anhydrous sodium sulfate, and filtered. The organic solvents were removed under reduced pressure to give a crude material, which was purified by flash silica gel column chromatography (hexane-EtOAc = 1:1 to 1:2) to afford indole **21ta** (56.6 mg, 0.0877 mmol, 89%); A white amorphous; $[\alpha]_D^{18}$ +37.8 (*c* 0.160, $CHCl_3$); IR (film): 3171, 2926, 2849, 1697, 1529, 1366, 1249, 1161, 1024, 973, 837 cm^{-1}; ^1H NMR (600 MHz, $CDCl_3$): δ 8.90 (1H, br s), 7.49 (2H, d, *J* = 6.6 Hz), 7.43 (2H, dd, *J* = 6.6, 6.6 Hz), 7.39–7.37 (1H, m), 7.19 (1H, br s), 7.00 (1H, m), 5.19 (1H, d, *J* = 11.4 Hz), 5.15 (1H, d, *J* = 11.4 Hz), 4.21–4.06 (6H, m), 3.34 (1H, br s), 3.22 (1H, dd, *J* = 16.8, 6.0 Hz), 2.83 (1H, dd, *J* = 16.8, 7.2 Hz), 1.54 (9H, s), 1.33 (6H, t, *J* = 7.2 Hz), 0.94 (9H, s), 0.16 (6H, s); ^{13}C NMR (100 MHz, $CDCl_3$): δ 154.0, 143.1 (d, *J* = 1.6 Hz), 136.5, 131.5 (d, *J* = 1.6 Hz), 128.6, 128.2, 127.9, 127.3 (d, *J* = 14.8 Hz), 126.3 (d, *J* = 13.2 Hz), 123.3 (d, *J* = 220.5 Hz), 112.5, 110.6 (d, *J* = 18.1 Hz), 103.7, 80.6, 70.4, 65.8, 62.6 (d, *J* = 4.9 Hz), 50.8, 34.4, 28.4, 25.9, 18.2, 16.2, –4.7, –

4.8; ^{31}P NMR (240 MHz, $CDCl_3$): δ 10.5 (Chemical shift is reported in ppm downfield from H_3PO_4 (0 ppm) as the external standard); HRMS (EI) *m/z*: $[M]^+$ Calcd for $C_{33}H_{49}N_2O_7PSi$ 644.3041; Found 644.3022.

Mesylate 29

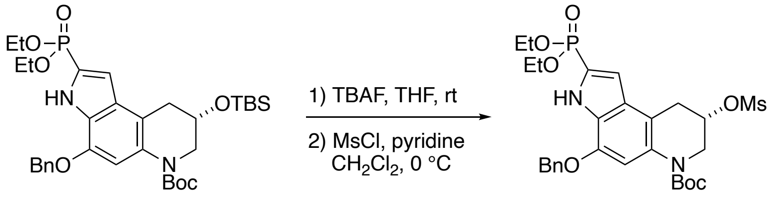

21ta **29**

To a solution of indole **21ta** (56.6 mg, 0.0877 mmol) in THF (1.8 mL) was added TBAF (0.219 mL, 1.0 M solution in THF, 0.219 mmol) at room temperature. After stirring at room temperature for 2 h, the reaction was quenched with H_2O, and the mixture was extracted with EtOAc three times. The combined organic extracts were washed with brine, dried over anhydrous sodium sulfate, and filtered. The organic solvents were removed under reduced pressure to give a crude material, which was used in the next reaction without further purification. To a solution of the crude alcohol in CH_2Cl_2 (1.2 mL) were added pyridine (66.5 μL, 0.824 mmol) and MsCl (32.1 μL, 0.412 mmol) at 0 °C. After stirring at 0 °C for 30 min, the reaction was quenched with 1 M HCl aq., and the mixture was extracted with CH_2Cl_2 three times. The combined organic extracts were washed with brine, dried over anhydrous sodium sulfate, and filtered. The organic solvents were removed under reduced pressure to give a crude material, which was purified by flash silica gel column chromatography (hexane-EtOAc = 1:3) to afford mesylate **29** (48.8 mg, 0.0802 mmol, 91%, 2 steps from **21ta**); A yellow amorphous; $[\alpha]_D^{18}$ –18.5 (*c* 0.710, $CHCl_3$); IR (film): 2980, 2917, 1698, 1540, 1369, 1246, 1173, 1025, 976, 770 cm^{-1}; ^{1}H NMR (400 MHz, $CDCl_3$): δ 8.99 (1H, br s), 7.50–7.37 (5H, m), 7.13 (1H, br s), 6.95 (1H, dd, *J* = 4.0, 2.0 Hz), 5.31–5.29 (1H, m), 5.18 (2H, s), 4.30 (1H, dd, *J* = 13.2, 6.0 Hz), 4.20–4.06 (4H, m), 3.76 (1H, d, *J* = 13.2 Hz), 3.40 (1H, dd, *J* = 17.6, 6.0 Hz), 3.23 (1H, dd, *J* = 17.6, 3.6 Hz), 3.08 (3H, s), 1.54 (9H, s), 1.33 (6H, t, *J* = 7.2 Hz); ^{13}C NMR (150 MHz, $CDCl_3$): δ 154.2, 143.6 (d, *J* = 2.0 Hz), 136.3, 131.5, 128.7, 128.4, 127.9, 127.0 (d, *J* = 16.1 Hz), 126.5 (d, *J* = 12.0 Hz), 124.0 (d, *J* = 218.6 Hz), 110.0 (d, *J* = 17.0 Hz), 109.8, 103.8, 81.4, 73.7, 70.6, 62.7 (d, *J* = 5.0 Hz), 47.8, 38.7, 31.0, 28.3, 16.2 (d, *J* = 7.1 Hz); ^{31}P NMR (160 MHz, $CDCl_3$): δ 10.9 (Chemical shift is reported in ppm downfield from triphenyl phosphine (–6 ppm) as the external standard); HRMS (ESI) *m/z*: $[M+H]^+$ Calcd for $C_{28}H_{38}N_2O_9PS$ 609.2030; Found 609.2006.

Amide 31

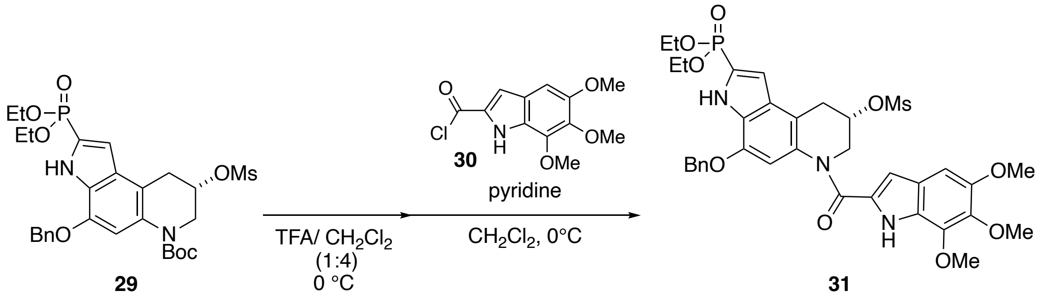

To a solution of mesylate **29** (22.2 mg, 0.0365 mmol) in CH_2Cl_2 (365 μL) was added TFA (91 μL) at 0 °C. After stirring at 0 °C for 15 min, the reaction was quenched with saturated aqueous $NaHCO_3$, and the mixture was extracted with EtOAc three times. The combined organic extracts were washed with brine, dried over anhydrous sodium sulfate, and filtered. The organic solvents were removed under reduced pressure to give a crude material, which was used in the next reaction without further purification. To a solution of the crude amine in CH_2Cl_2 (160 μL) were added pyridine (58.9 μL, 0.730 mmol) and **30**[20] (19.7 mg, 0.0731 mmol) in CH_2Cl_2 (80 μL) at 0 °C. After stirring at 0 °C for 30 min, the reaction was quenched with 1 M HCl aq., and the mixture was extracted with CH_2Cl_2 three times. The combined organic extracts were washed with saturated aqueous $NaHCO_3$ and brine, dried over anhydrous sodium sulfate, and filtered. The organic solvents were removed under reduced pressure to give a crude material, which was purified by flash silica gel column chromatography (hexane-EtOAc = 1:1 to 1:3) to afford amide **31** (17.3 mg, 0.0233 mmol, 64%, 2 steps from **29**); A yellow amorphous; $[\alpha]_D^{18}$ –88.3 (*c* 0.210, $CHCl_3$); IR (film): 3195, 2988, 2936, 1614, 1528, 1364, 1313, 1244, 1172, 1104, 1048, 1021, 972, 744 cm^{-1}; ^1H NMR (600 MHz, $CDCl_3$): δ 9.26 (1H, br s), 9.18 (1H, br s), 7.23–7.21 (5H, m), 7.04 (1H, dd, *J* = 4.2, 1.8 Hz) , 6.66 (1H, s), 6.62 (1H, s), 6.19 (1H, br s), 5.44–5.41 (1H, m), 4.91 (1H, d, *J* = 11.4 Hz) 4.88 (1H, d, *J* = 11.4 Hz), 4.66 (1H, m), 4.24–4.12 (4H, m), 4.06 (3H, s), 3.99–3.97 (1H, m), 3.92 (3H, s), 3.83 (3H, s), 3.47 (1H, dd, *J* = 17.4, 6.0 Hz), 3.35 (1H, dd, *J* = 17.4, 4.2 Hz), 2.95 (3H, s), 1.37 (6H, m); ^{13}C NMR (150 MHz, $CDCl_3$): δ 145.9, 135.3, 130.4, 127.6, 126.4, 124.2, 120.8, 118.7, 118.2, 117.9, 117.4, 117.3, 117.2 (d, *J* = 11.4 Hz), 115.8, 115.2, (d, *J* = 176.6 Hz), 113.8, 104.6, 103.4 (d, *J* = 12.6 Hz), 102.2, 99.0, 93.5, 75.2, 71.9, 65.7 (d, *J* = 3.5 Hz), 64.5, 64.2, 60.3, 54.6, 46.4, 39.9, 28.4 (d, *J* = 5.7 Hz); ^{31}P NMR (240 MHz, $CDCl_3$): δ 9.7 (Chemical shift is reported in ppm downfield from H_3PO_4 (0 ppm) as the external standard); HRMS (ESI) *m/z*: $[M+H]^+$ Calcd for $C_{35}H_{40}N_3O_{11}PS$ 742.2194; Found 742.2186.

Duocarmycin derivative 25

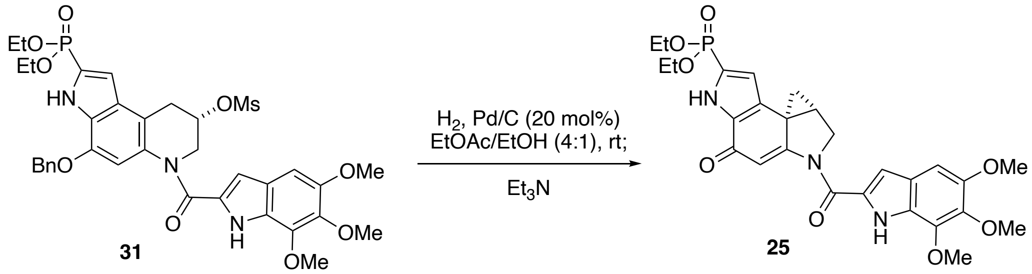

To a solution of amide **31** (5.1 mg, 6.9 μmol) and palladium on activated carbon (Pd 10%, wetted with ca. 50% H_2O, 2.7 mg) in EtOAc (110 μL) and EtOH (28 μl) was stirred under hydrogen atmosphere (1 atm). After stirring at room temperature for 3 h, Et_3N (28.9 μL, 0.206 mmol) was added to the mixture. After stirring at room temperature for 4 h, palladium on activated carbon was removed by filtration and concentrated under reduced pressure to give a crude material, which was purified by flash silica gel column chromatography (hexane-EtOAc = 4:1) to afford duocarmycin analog **25** (2.0 mg, 3.6 μmol, 52%); A white amorphous; $[\alpha]_D^{18}$ –88.3 (*c* 0.210, $CHCl_3$); IR (film): 3204, 2982, 2934, 1635, 1457, 1386, 1249, 1108, 1048, 975 cm^{-1}; ^1H NMR (600 MHz, $CDCl_3$): δ 10.12 (1H, br s), 9.46 (1H, br s), 7.01 (1H, s), 6.95 (1H, d, *J* = 2.4 Hz), 6.78 (1H, s), 6.51 (1H, d, *J* = 3.0 Hz), 4.45 (1H, dd, *J* = 4.8, 10.2 Hz), 4.39 (1H, d, *J* = 10.2 Hz), 4.21–4.10 (4H, m), 4.07 (3H, s), 3.94 (3H, s), 3.89 (3H, s), 2.79–2.76 (1H, m), 1.76 (1H, dd, *J* = 4.2, 7.2 Hz), 1.57 (1H, t, *J* = 10.8 Hz), 1.33 (6H, t, *J* = 7.2 Hz); ^{13}C NMR (150 MHz, $CDCl_3$): δ 177.8, 161.4, 161.2, 150.4, 141.0, 138.9, 133.0 (d, *J* = 11.4 Hz), 129.9 (d, *J* = 15.8 Hz), 128.5, 126.4, 125.4 (d, *J* = 220.5 Hz), 123.2, 112.4, 111.2 (d, *J* = 18.6 Hz), 107.8, 97.6, 62.8 (d, *J* = 4.2 Hz), 61.5, 61.2, 56.3, 54.9, 31.4, 25.9, 23.5, 16.3 (d, *J* = 5.7 Hz); ^{31}P NMR (240 MHz, $CDCl_3$): δ 8.6 (Chemical shift is reported in ppm downfield from H_3PO_4 (0 ppm) as the external standard); HRMS (ESI) *m/z*: $[M+H]^+$ Calcd for $C_{27}H_{31}N_3O_8P$ 556.1843; Found 556.1852.

---

3. Biological Experiment: Methods and Supplementary Data

a) Structures of duocarmycin phosphonate analog (**25**) and duocarmycin SA (**24**)

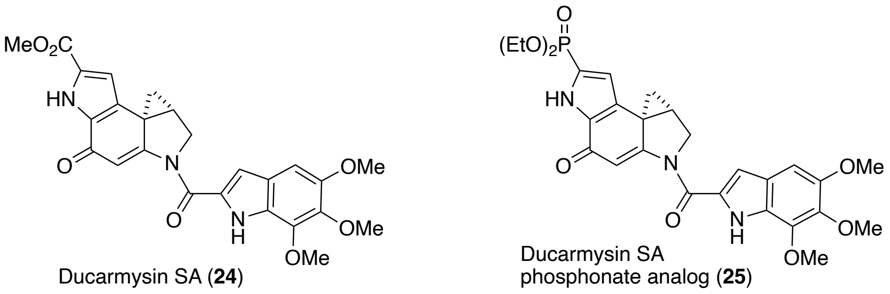

b) Cytotoxicity against HeLa S3 cell line

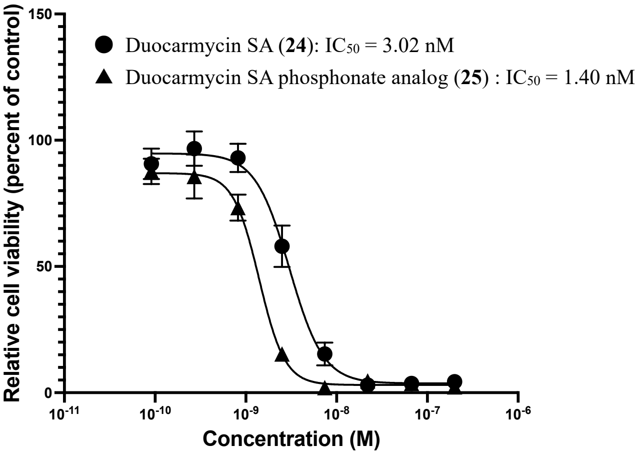

c) Cytotoxicity against KPL-4 cell line

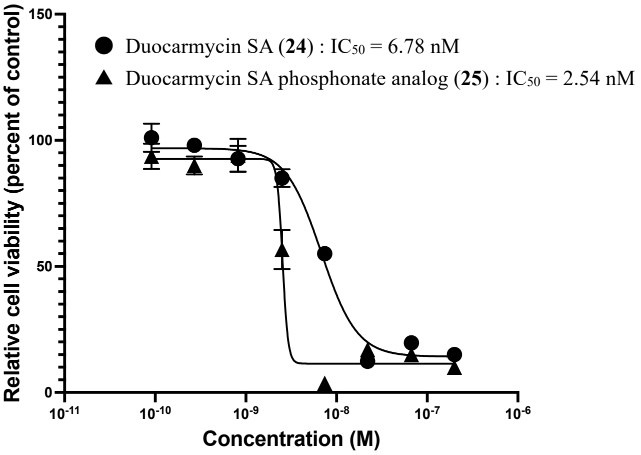

Figure S1. Cytotoxicity of Duocarmycin SA (**24**) and Duocarmycin SA phosphonate analog (**25**)

The cytotoxicity assay using the HeLa S3 cells and KPL-4 cells was performed as follows. The HeLa S3 cell line, sourced from ATCC, was cultured in Ham's F-12 Medium (#087-08335; FUJIFILM Wako Pure Chemical Corporation, Osaka, Japan) supplemented with 10% fetal bovine serum. Human breast cancer KPL-4 cells, generously provided by Professor Kurebayashi (Kawasaki Medical School, Kurashiki, Japan)[21], were maintained in low-glucose Dulbecco's Modified Eagle Medium (DMEM; #041–29775; FUJIFILM Wako Pure Chemical Corporation, Osaka, Japan) also supplemented with 10% fetal bovine serum.

The cytotoxicity assay was conducted following previously described protocols.[22] Briefly, 5×10^3 cells per well in 50 μL of medium were seeded into 96-well plates and cultured overnight. The cells were subsequently exposed to different concentrations of duocarmycin analogs, prepared through a 3-fold serial dilution starting from 200 nM, in 50 μL medium. Incubations were performed separately for 48 hours at 37 °C. Following this, 10 μL of CCK-8 reagent (#341-08001; Dojindo Laboratories, Kumamoto, Japan) was added to each well, and the plates were incubated at 37 °C for an additional 1.5 hours. Absorbance was measured at 450 nm using a microplate reader (EnSpire, PerkinElmer).

Data analysis included measurements from blank wells (containing only culture medium and CCK-8 reagent) and control wells (untreated cells with culture medium and CCK-8 reagent). Graphing and IC_{50} calculations were carried out using Prism 10 software (GraphPad, Boston, MA, USA).

4. References

1) Stevens, R. V.; Bisacchi, G. S. An Efficient Remarkably Regioselective Synthesis of Benzocyclobutenones from Benzynes and 1,1-Dimethoxyethylene. *J. Org. Chem.* **1982**, *47,* 2393-2396.
2) Guo, J.-H.; Liu, Y.; Lin, X.-C.; Tang, T.-M.; Wang, B.-Q.; Hu, P.; Zhao, K.-Q.; Song, F.; Shi, Z.-J. Site-Selective C-C Cleavage of Benzocyclobutenones Enabled by a Blocking Strategy Using Nickel Catalysis. *Angew. Chem. Int. Ed.* **2021**, *60,* 19079-19084.
3) Chen, Y.; Willis, M. C. An Aryne-Based Route to Substituted Benzoisothiazoles. *Org. Lett.* **2015**, *17,* 4786-4789.
4) Yoshida, Y.; Nakmura, Y.; Uchida, K.; Hazama, Y.; Hosoya, T. Aryne Relay Chemistry en Route to Aminoarenes: Synthesis of 3-Aminoaryne Precursors via Regioselective Silylamination of 3-(Triflyloxy)arynes. *Org. Lett.* **2016**, *18,* 6212-6215.
5) Nakamura, Y.; Miyata, Y.; Uchida, K.; Yoshida, S.; Hosoya, T. 3-Thioaryne Intermediates for the Synthesis of Diverse Thioarenes. *Org. Lett.* **2019**, *21*, 5252-5258.
6) Kohser, S. C.; Dongol, K. G.; Butenschön, H. Synthesis of spiro annelated isochromanones by ring expansion of benzocyclobutenones in the presence of lithium diisopropylphosphide. *Heterocycles* **2017**, *74*, 339-350.
7) Gokhale, A.; Schiess, P. Regioselectivity of the Base-induced Ring Cleavage of 1-Oxygenated Derivatives of Cyclobutabenzene. *Helv. Chim. Acta* **1998**, *81,* 251-267.
8) Uchida, K.; Yoshida, S.; Hosoya, T. Three-Component Coupling of Triflyloxy-Substituted Benzocyclobutenones, Organolithium Reagents, and Arynophiles Promoted by Generation of Aryne via Carbon-Carbon Bond Cleavage. *Org. Lett.* **2017**, *19,* 1184-1187.
9) Cho, H.; Iwama, Y.; Sugimoto, K.; Mori, S.; Tokuyama, H. Regioselective Synthesis of Heterocycles Containing Nitrogen Neighboring an Aromatic Ring by Reductive Ring Expansion Using Diisobutylaluminum Hydride and Studies on the Reaction Mechanism. *J. Org. Chem.* **2010**, *75,* 627-636.
10) Brown, S. P.; Dransfield, P. J.; Houze, J.; Kohn, T. J.; Liu, J.; Medina, J.; Pattaropong, V.; Shen, W.; Vimolratana, M.; Wang, Y.; Yu, M. Zhu, L. Preparation of conformationally constrained cyclic carboxylic acid derivatives useful as GPR40 modulators for treating metabolic disorders. WO2009111056 A1 2009-09-11.
11) Yano, T.; Kawasaki, T.; Yuhki, T.; Ishida, N.; Murakami, M. Synthetic Approach to Benzocyclobutenones Using Visible Light and a Phosphonate Auxiliary. *Org. Lett.* **2018**, *20,* 1224-1227.
12) Wang, Y.; Liu, J.; Drasfield, P. J.; Zhu, L.; Wang, Z.; Du, X.; Jiao, X.; Su, Y.; Li, A.; Brown, S. P.; Kasparian, A.; Vimolratana, M.; Yu, M.; Pattaropong, V.; Houze, J. B.; Swaminath, G.; Tran, T.;

Nguyen, K.; Guo, Q.; Zhang, J.; Zhuang, R.; Li, F.; Miao, L.; Bartberger, M. D.; Correll, T. L.; Chow, D.; Wong, S.; Luo, J.; Lin, D. C.-H.; Medina, J. C. Discovery and Optimization of Potent GPR40 Full Agonists Containing Tricyclic Spirocycles. *ACS Med. Chem. Lett.* **2013**, *4,* 551-555.

13) Liebeskind, L. S.; Lescosky, L. J.; McSwain Jr., C. M. Synthesis of Substituted Benzocyclobutenediones. *J. Org. Chem.* **1989**, *54,* 1435-1439.

14) Hosoya, T.; Hasegawa, T.; Kuriyama, Y.; Suzuki, K.; Stereospecificity in [2 + 2] Cycloaddition of Benzyne and Ketene Silyl Acetal. *Tetrahedron Lett.* **1995**, *36,* 3377-3380.

15) Oisaki, K.; Suto, Y.; Kanai, M.; Shibasaki, M. A New Method for the Catalytic Aldol Reaction to Ketones. *J. Am. Chem. Soc*. **2003**, *125*, 5644-5645.

16) Carroll, F. I.; Robinson, T. P.; Brieaddy, L. E.; Atkinson, R. N.; Mascarella, S. W.; Damaj, M. I.; Martin, B. R.; Navarro, H. A. Synthesis and Nicotinic Acetylcholine Receptor Binding Properties of Bridged and Fused Ring Analogues of Epibatidine. *J. Med. Chem.* **2007**, *50,* 6383-6391.

17) Imaizumi, T.; Yamashita, Y.; Nakazawa, Y.; Okano, K.; Sakata, J.; Tokuyama, H. Total Synthesis of (+)-CC-1065 Utilizing Ring Expansion Reaction of Benzocyclobutenone Oxime Sulfonate. *Org. Lett.* **2019**, *21*, 6185-6189.

18) Deng, Y.; You, S.; Ruan, M.; Wang, Y.; Chen, Z.; Yang, G.; Gao, M. Electrochemical Regioselective Phosphorylation of Nitrogen-Containing Heterocycles and Related Derivatives. *Adv. Synth. Catal*. **2021**, *363*, 464 – 469.

19) Okano, K.; Tokuyama, H.; Fukuyama, T. Total Synthesis of (+)-Yatakemycin. *J. Am. Chem. Soc*. **2006**, *128*, 7136-7137.

20) Yamada, K.; Kurokawa, T.; Tokuyama, H.; Fukuyama, T. Total Synthesis of Duocarmycins. *J. Am. Chem. Soc*. **2003**, *125*, 6630-6631.

21) Kurebayashi, J.; Otsuki, T.; Tang, C. K.; Kurosumi, M.; Yamamoto, S.; Tanaka, K.; Mochizuki, M.; Nakamura, H.; Soono, H. Isolation and Characterization of a New Human Breast Cancer Cell Line, KPL-4, Expressing the Erb B Family Receptors and Interleukin-6. *British J. Cancer* **1999**, *79*, 707-717.

22) Sakata, J.; Tatsumi, T.; Sugiyama, A.; Shimizu, A.; Inagaki, Y.; Katoh, H.; Yamashita, T.; Takahashi, K.; Aki, S.; Kaneko, Y.; Kawamura, T.; Miura, M.; Ishii, M.; Osawa, T.; Tanaka, T.; Ishikawa, S.; Tsukagoshi, M.; Chansler, M.; Kodama, T.; Kanai, M.; Tokuyama, H.; Yamatsugu, K. Antibody-Mimetic Drug Conjugate with Efficient Internalization Activity using Anti-HER2 VHH and Duocarmycin, *Protein Expr. Purif.* **2024**, *214*, 106375-106385.

5. ^{1}H and ^{13}C NMR Spectra

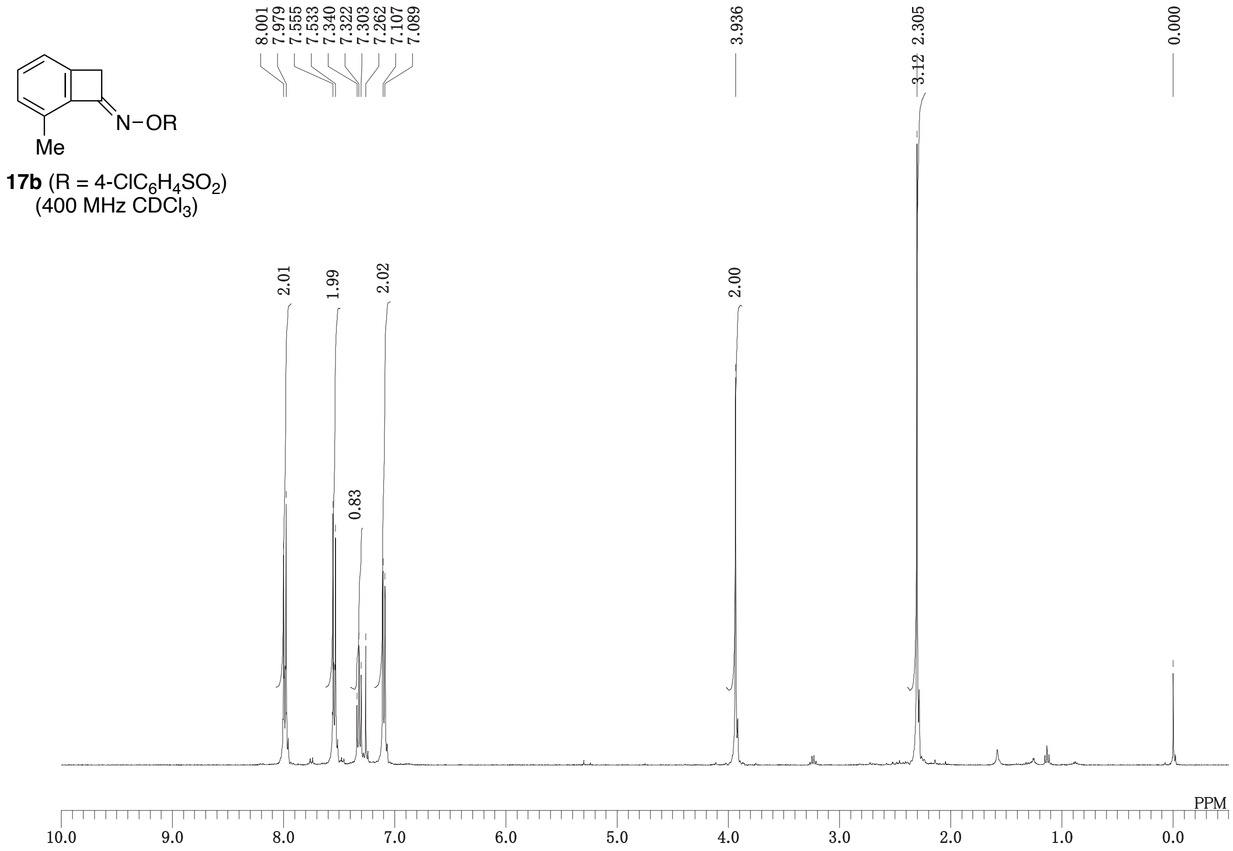

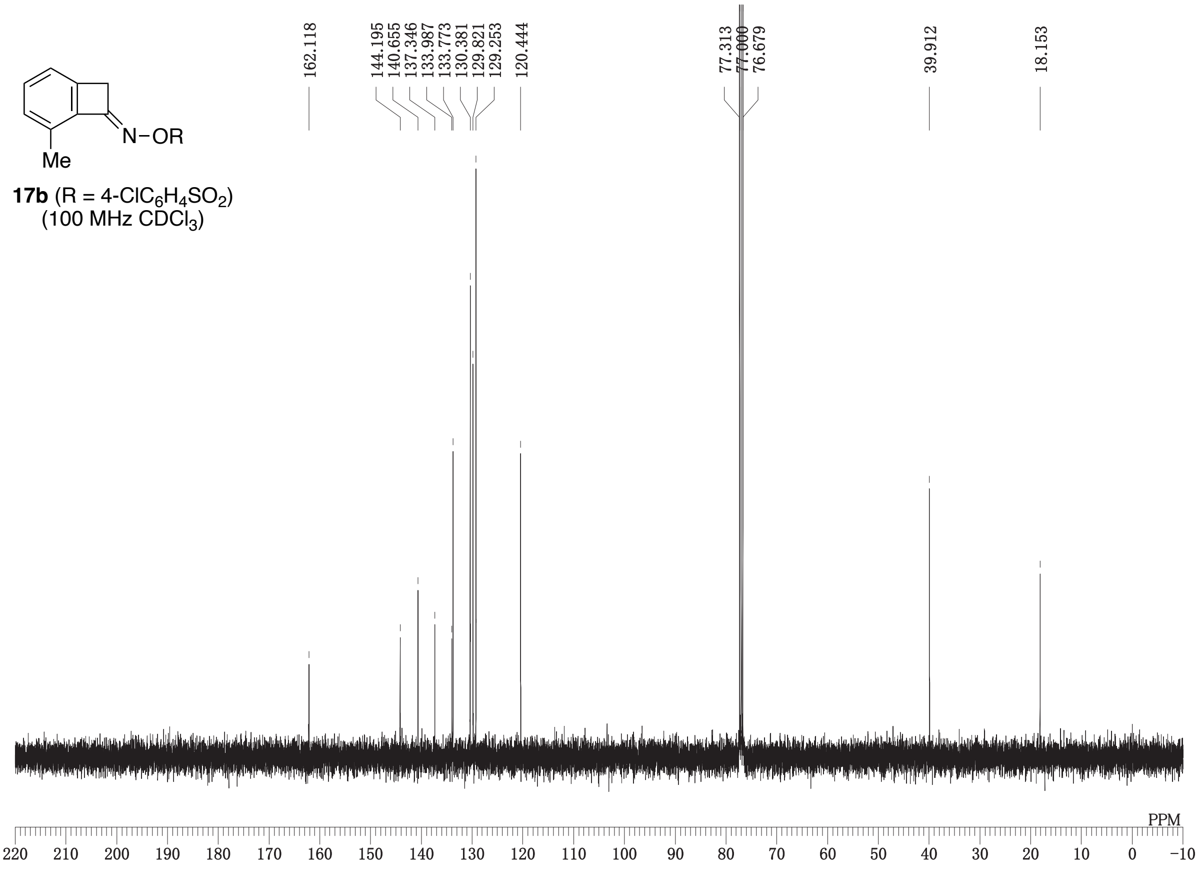

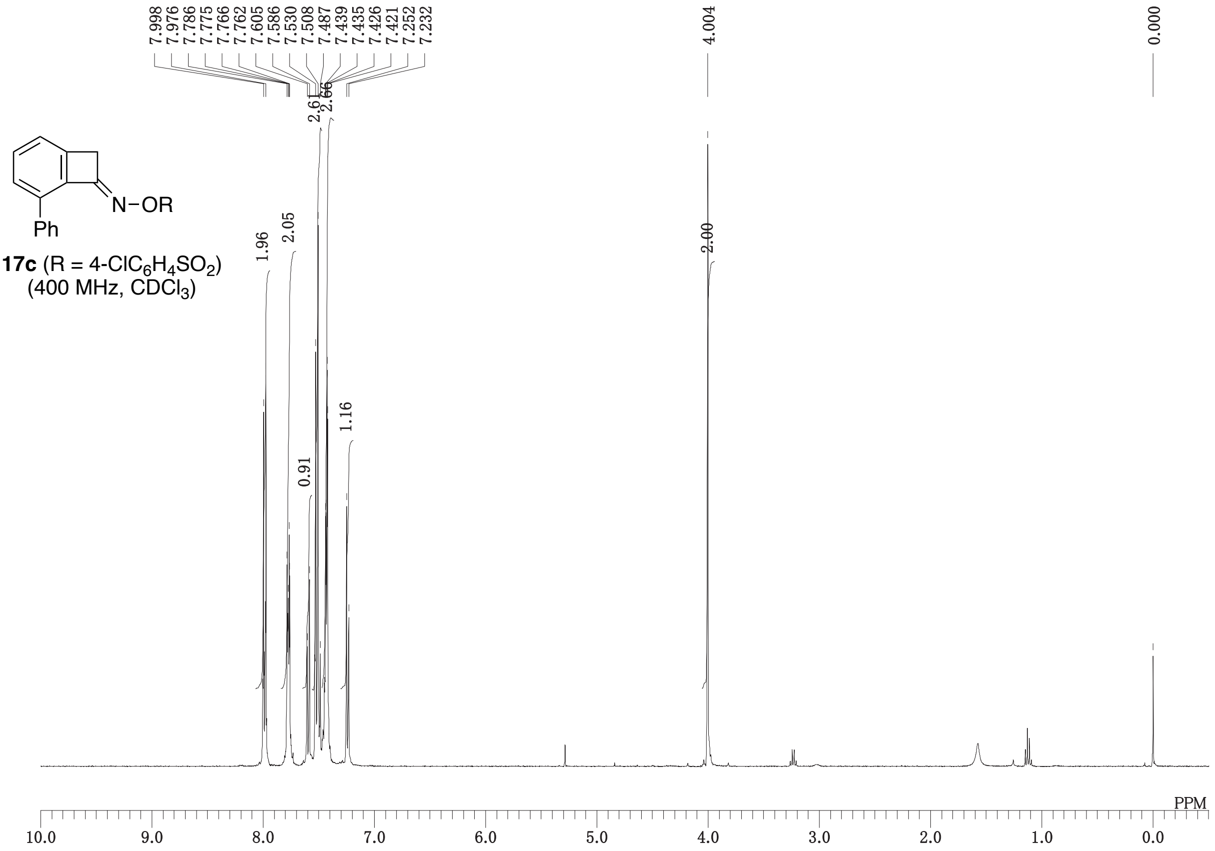
7.998
7.976
7.786
7.775
7.766
7.762
7.605
7.586
7.530
7.508
7.487
7.439
7.435
7.426
7.421
7.252
7.232
4.004
0.000
N−OR
Ph
17c (R = 4-ClC6H4SO2)
(400 MHz, CDCl3)
1.96
2.05
2.61
2.66
0.91
1.16
2.00
PPM
10.0
9.0
8.0
7.0
6.0
5.0
4.0
3.0
2.0
1.0
0.0

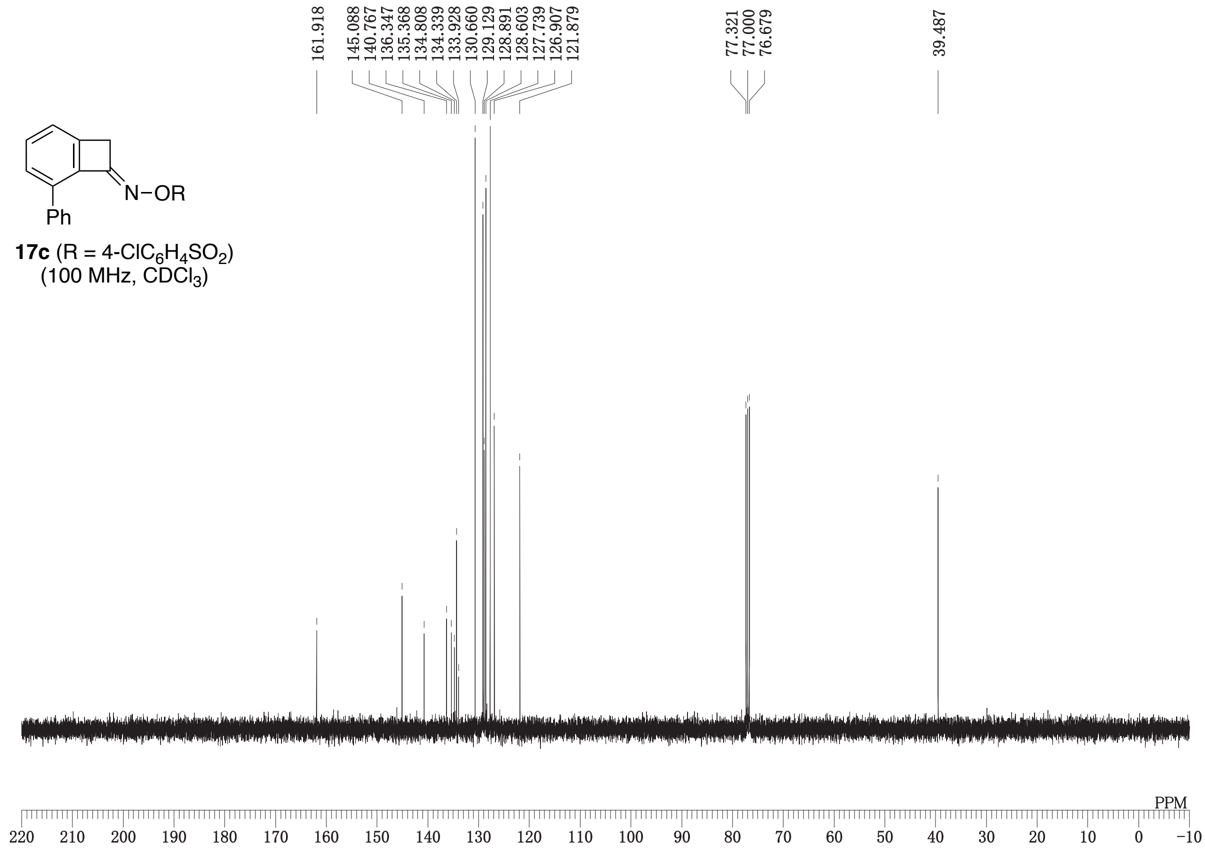
161.918
145.088
140.767
136.347
135.368
134.808
134.339
133.928
130.660
129.129
128.891
128.603
127.739
126.907
121.879
77.321
77.000
76.679
39.487
N−OR
Ph
17c (R = 4-ClC6H4SO2)
(100 MHz, CDCl3)
PPM
220
210
200
190
180
170
160
150
140
130
120
110
100
90
80
70
60
50
40
30
20
10
0
−10

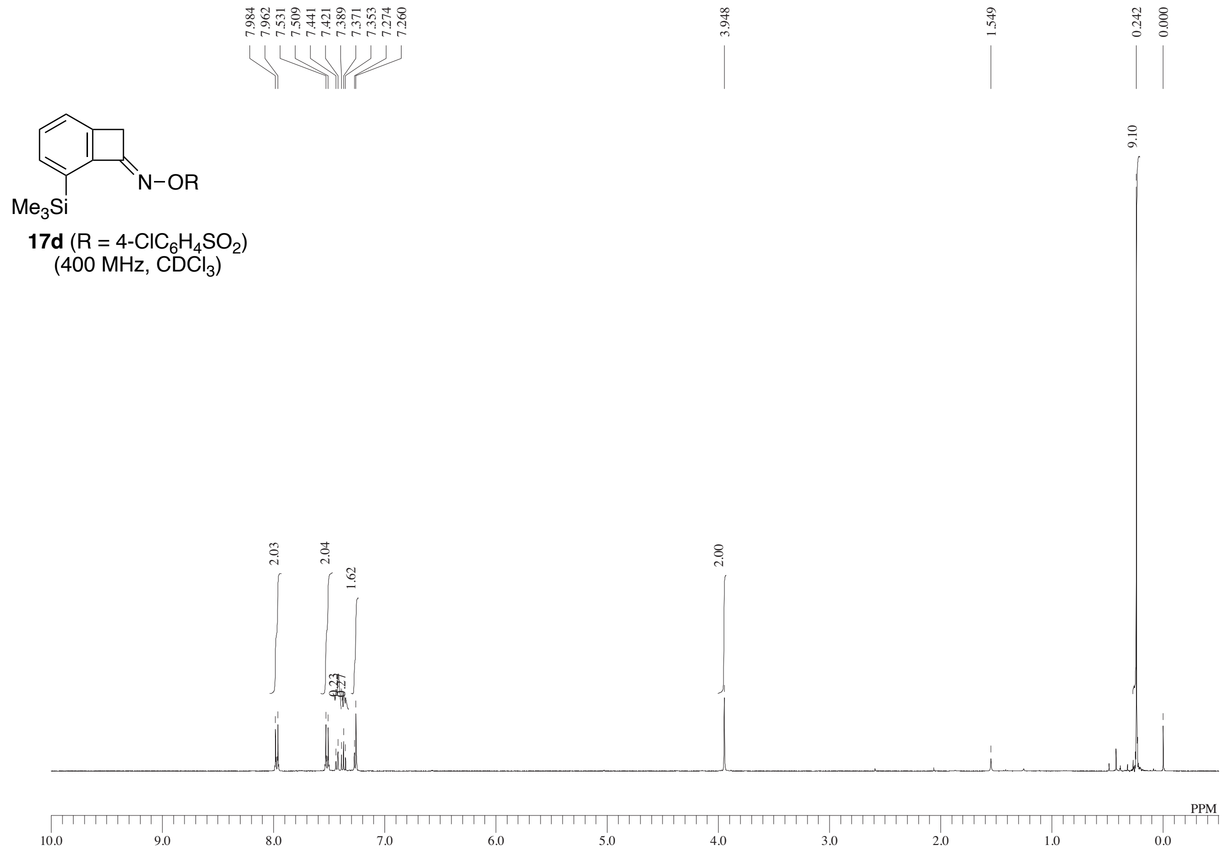
7.984
7.962
7.531
7.509
7.441
7.421
7.389
7.371
7.353
7.274
7.260
3.948
1.549
0.242
0.000
9.10
N−OR
Me3Si
17d (R = 4-ClC6H4SO2)
(400 MHz, CDCl3)
2.03
2.04
1.62
2.00
PPM
10.0
9.0
8.0
7.0
6.0
5.0
4.0
3.0
2.0
1.0
0.0

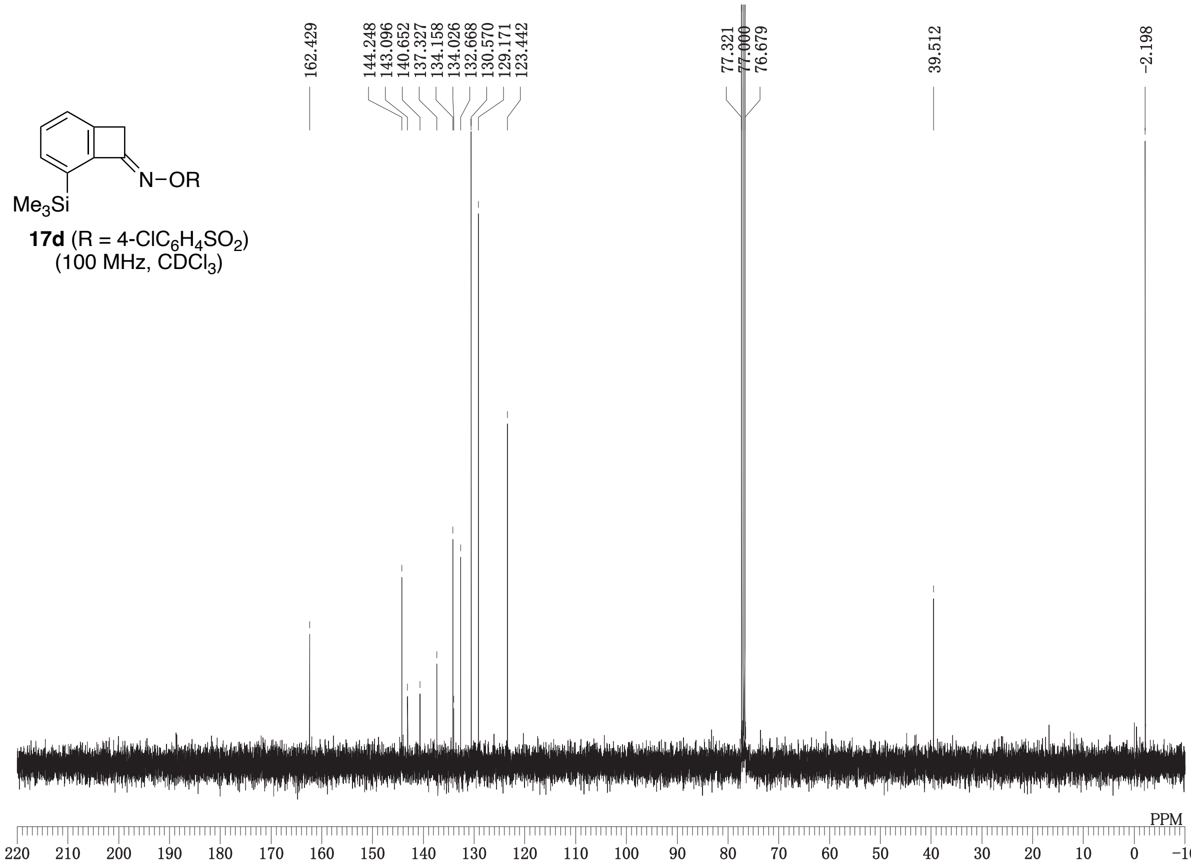
162.429
144.248
143.096
140.652
137.327
134.158
134.026
132.668
130.570
129.171
123.442
77.321
77.000
76.679
39.512
−2.198
N−OR
Me3Si
17d (R = 4-ClC6H4SO2)
(100 MHz, CDCl3)
PPM
220
210
200
190
180
170
160
150
140
130
120
110
100
90
80
70
60
50
40
30
20
10
0
−10

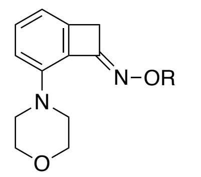

17e (R = 4-ClC$_6$H$_4$SO$_2$)
(400 MHz, CDCl$_3$)

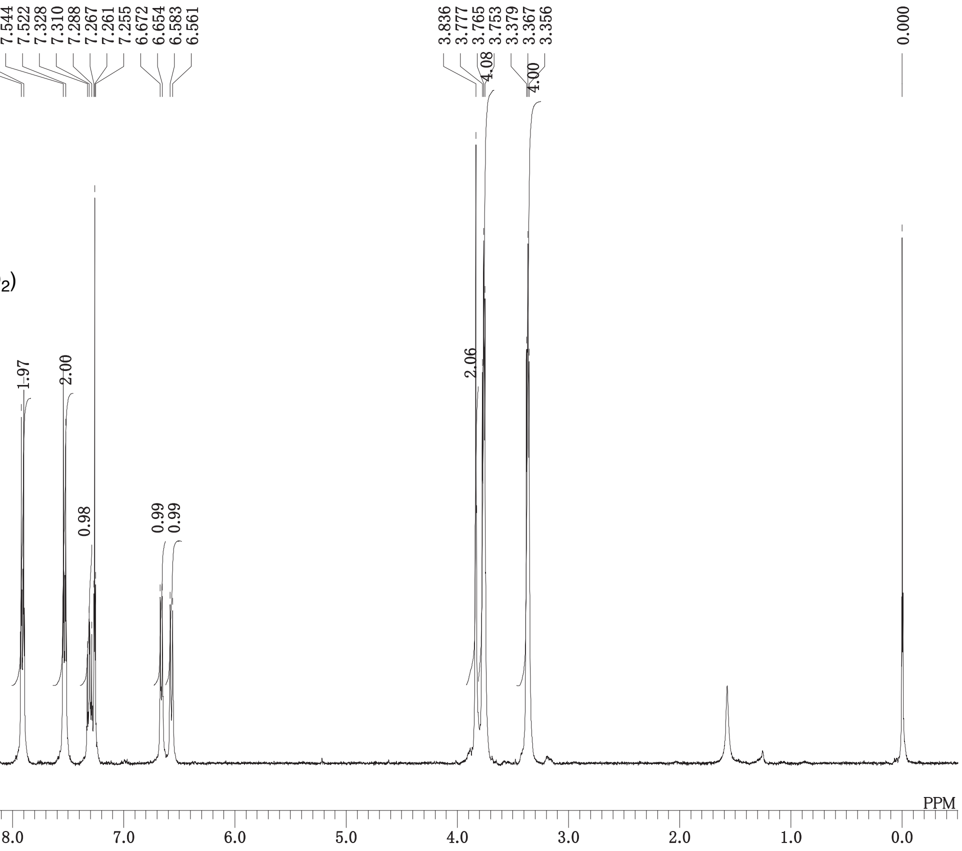

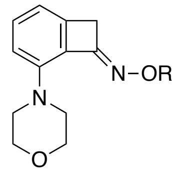

17e (R = 4-ClC$_6$H$_4$SO$_2$)
(100 MHz, CDCl$_3$)

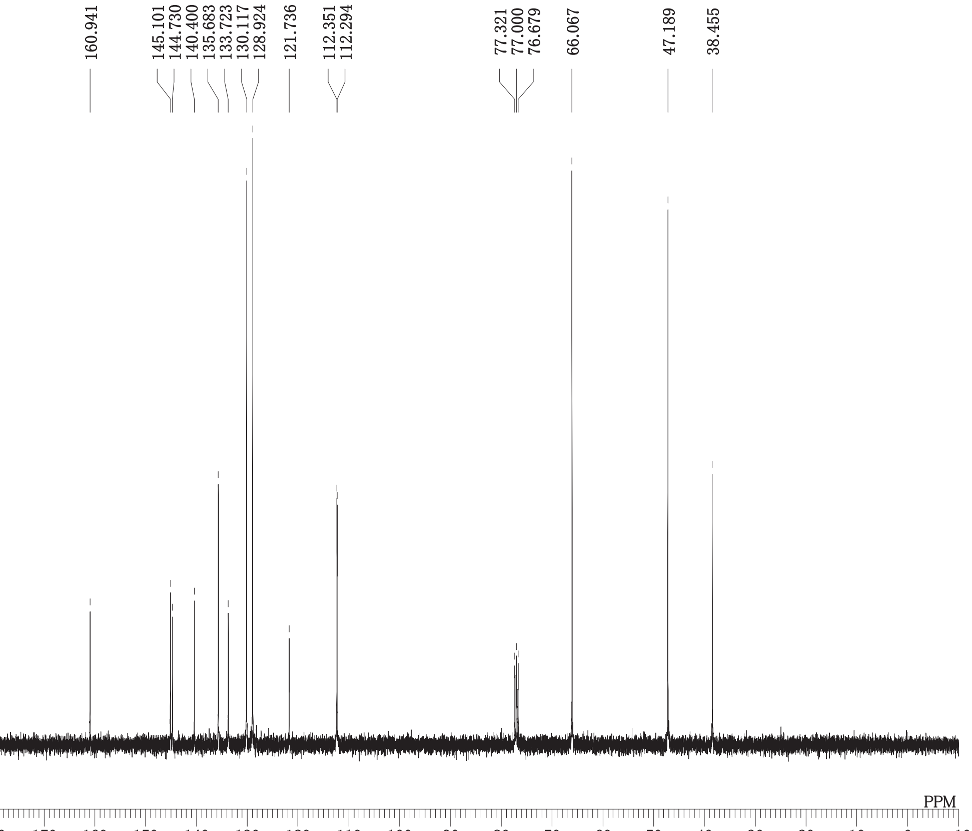

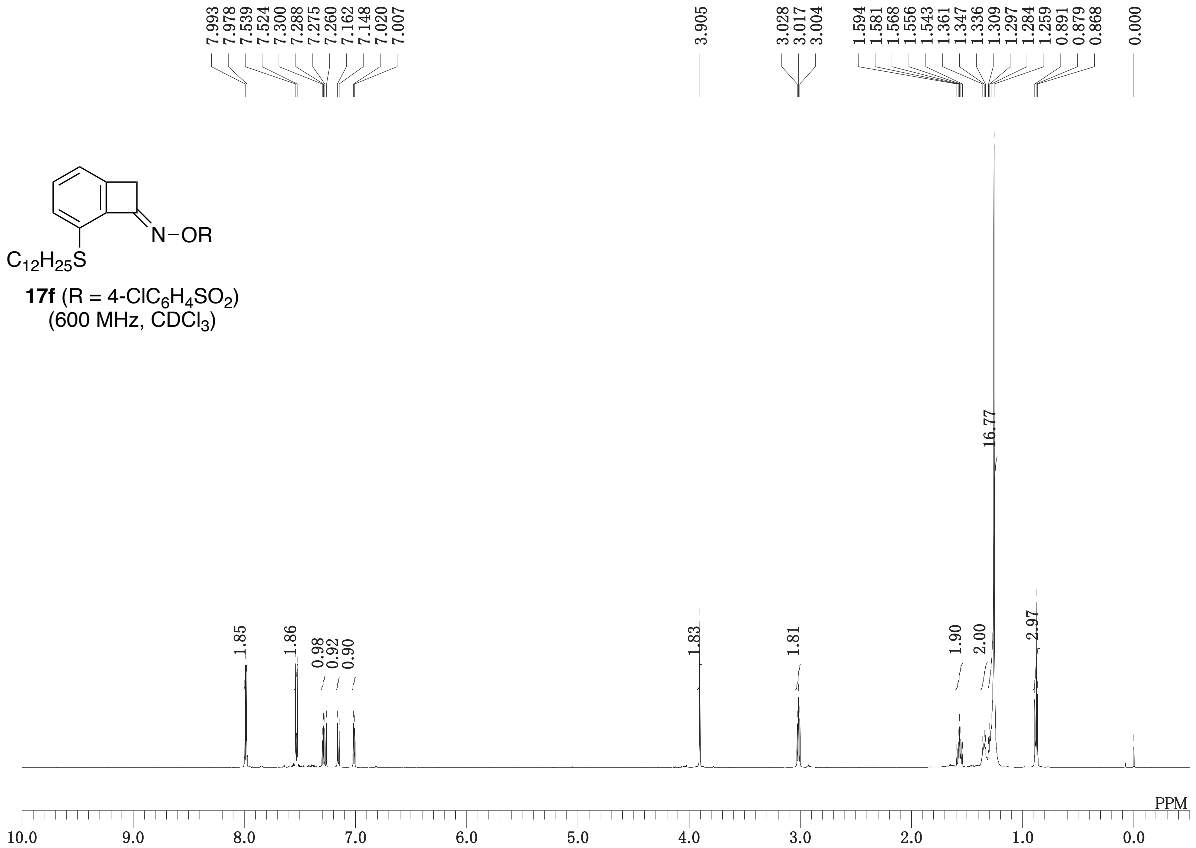
7.993 7.978 7.539 7.524 7.300 7.288 7.275 7.260 7.162 7.148 7.020 7.007
3.905
3.028 3.017 3.004
1.594 1.581 1.568 1.556 1.543 1.361 1.347 1.336 1.309 1.297 1.284 1.259 0.891 0.879 0.868
0.000
N−OR
$C_{12}H_{25}S$
17f (R = 4-$ClC_6H_4SO_2$)
(600 MHz, $CDCl_3$)
16.77
1.85 1.86 0.98 0.92 0.90 1.83 1.81 1.90 2.00 2.97
PPM
10.0 9.0 8.0 7.0 6.0 5.0 4.0 3.0 2.0 1.0 0.0

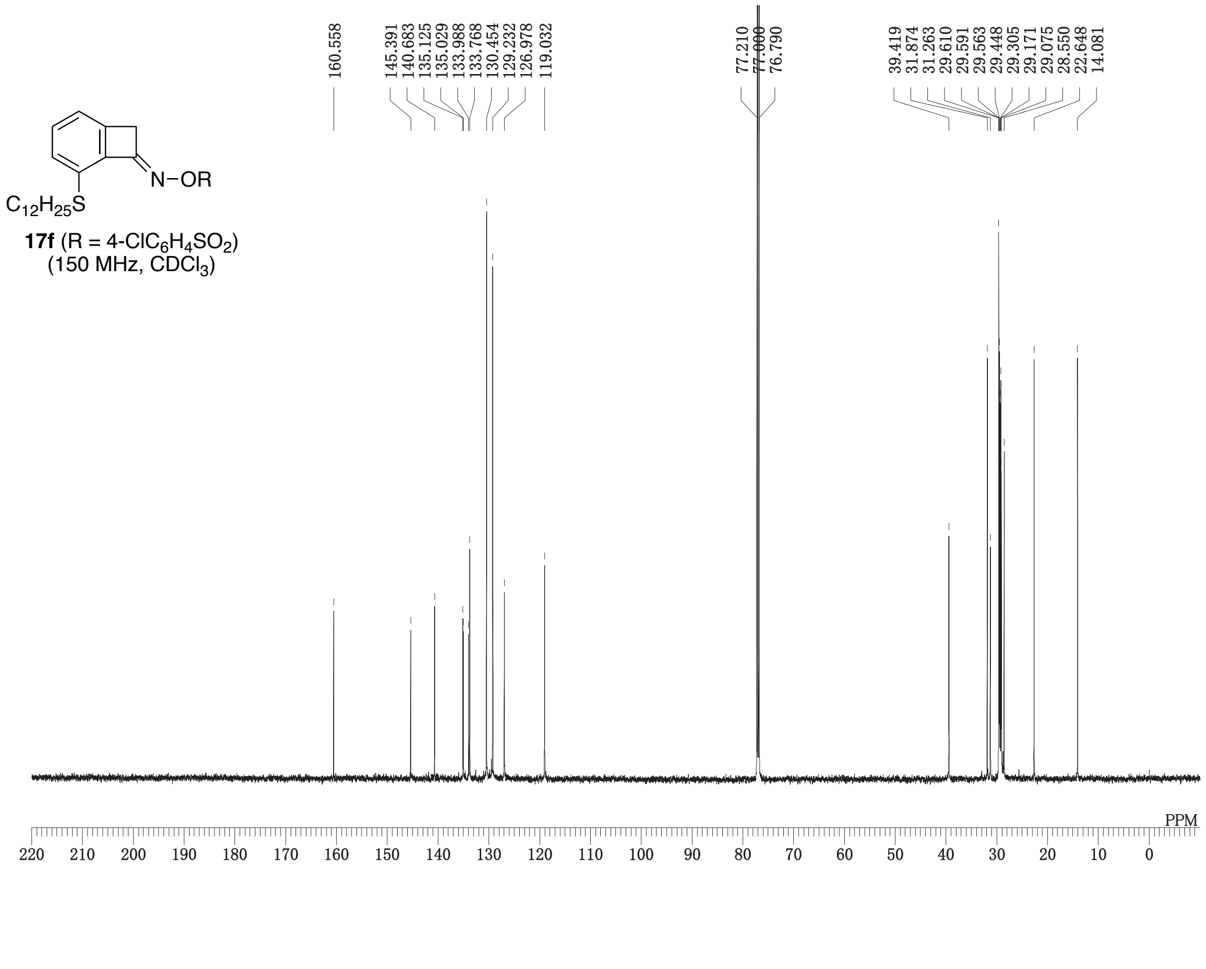
160.558
145.391 140.683 135.125 135.029 133.988 133.768 130.454 129.232 126.978 119.032
77.210 77.000 76.790
39.419 31.874 31.263 29.610 29.591 29.563 29.448 29.305 29.171 29.075 28.550 22.648 14.081
N−OR
$C_{12}H_{25}S$
17f (R = 4-$ClC_6H_4SO_2$)
(150 MHz, $CDCl_3$)
PPM
220 210 200 190 180 170 160 150 140 130 120 110 100 90 80 70 60 50 40 30 20 10 0

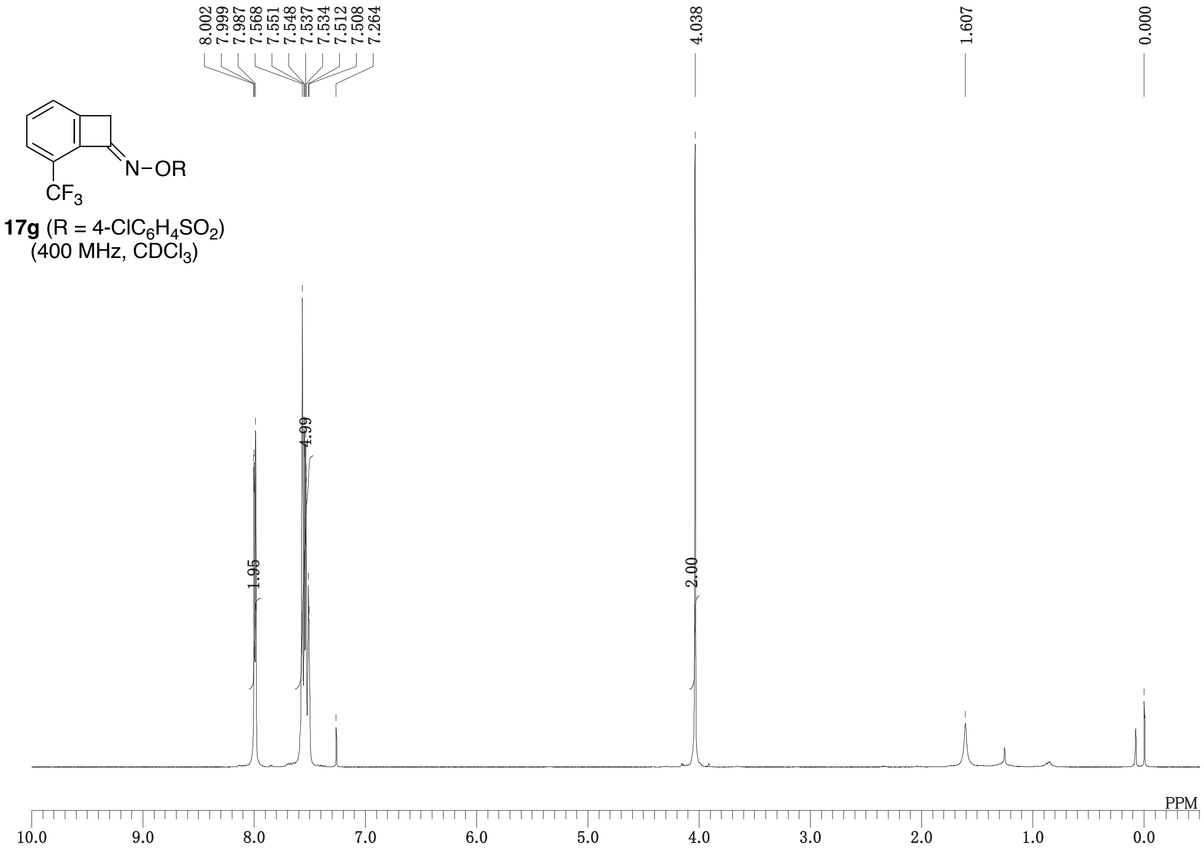
8.002
7.999
7.987
7.568
7.551
7.548
7.537
7.534
7.512
7.508
7.264
4.038
1.607
0.000
N−OR
CF_3
17g (R = 4-$ClC_6H_4SO_2$)
(400 MHz, $CDCl_3$)
1.95
4.99
2.00
PPM
10.0
9.0
8.0
7.0
6.0
5.0
4.0
3.0
2.0
1.0
0.0

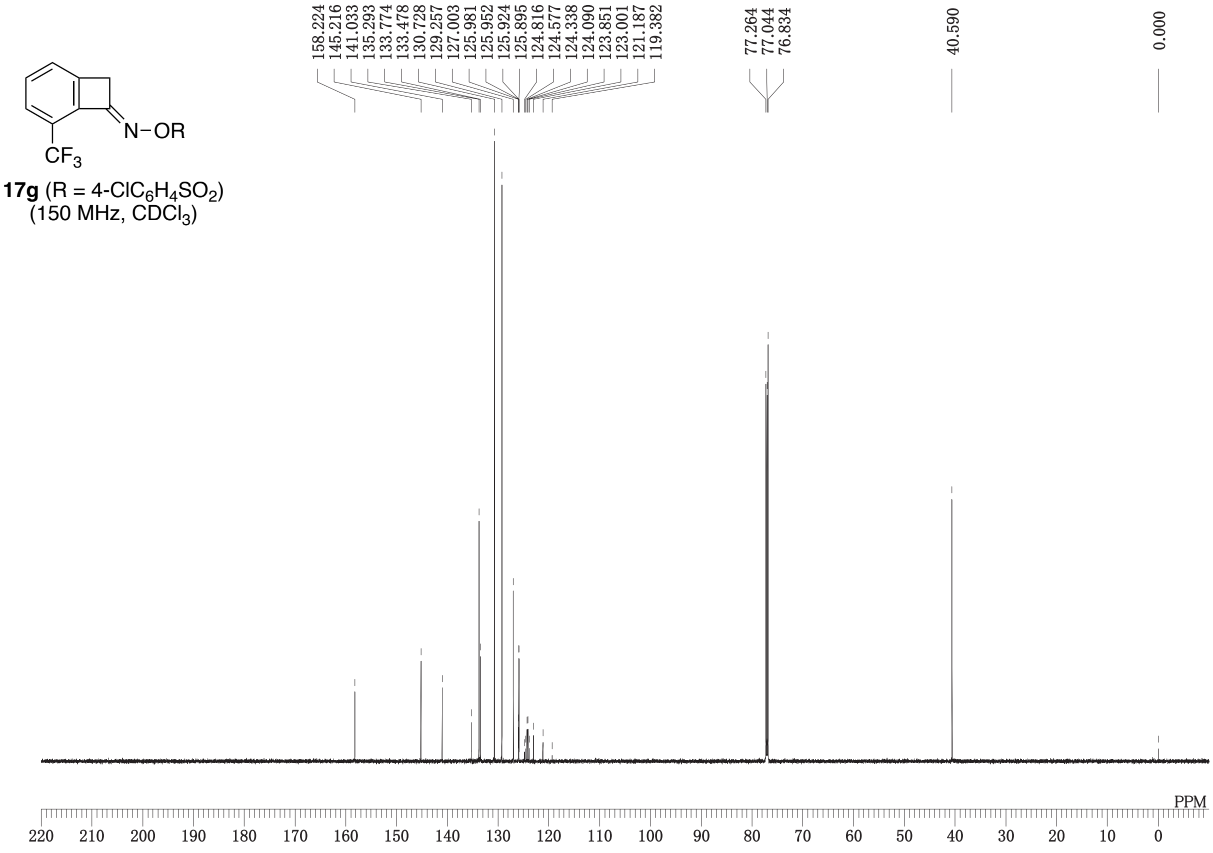
158.224
145.216
141.033
135.293
133.774
133.478
130.728
129.257
127.003
125.981
125.952
125.924
125.895
124.816
124.577
124.338
124.090
123.851
123.001
121.187
119.382
77.264
77.044
76.834
40.590
0.000
N−OR
CF_3
17g (R = 4-$ClC_6H_4SO_2$)
(150 MHz, $CDCl_3$)
PPM
220
210
200
190
180
170
160
150
140
130
120
110
100
90
80
70
60
50
40
30
20
10
0

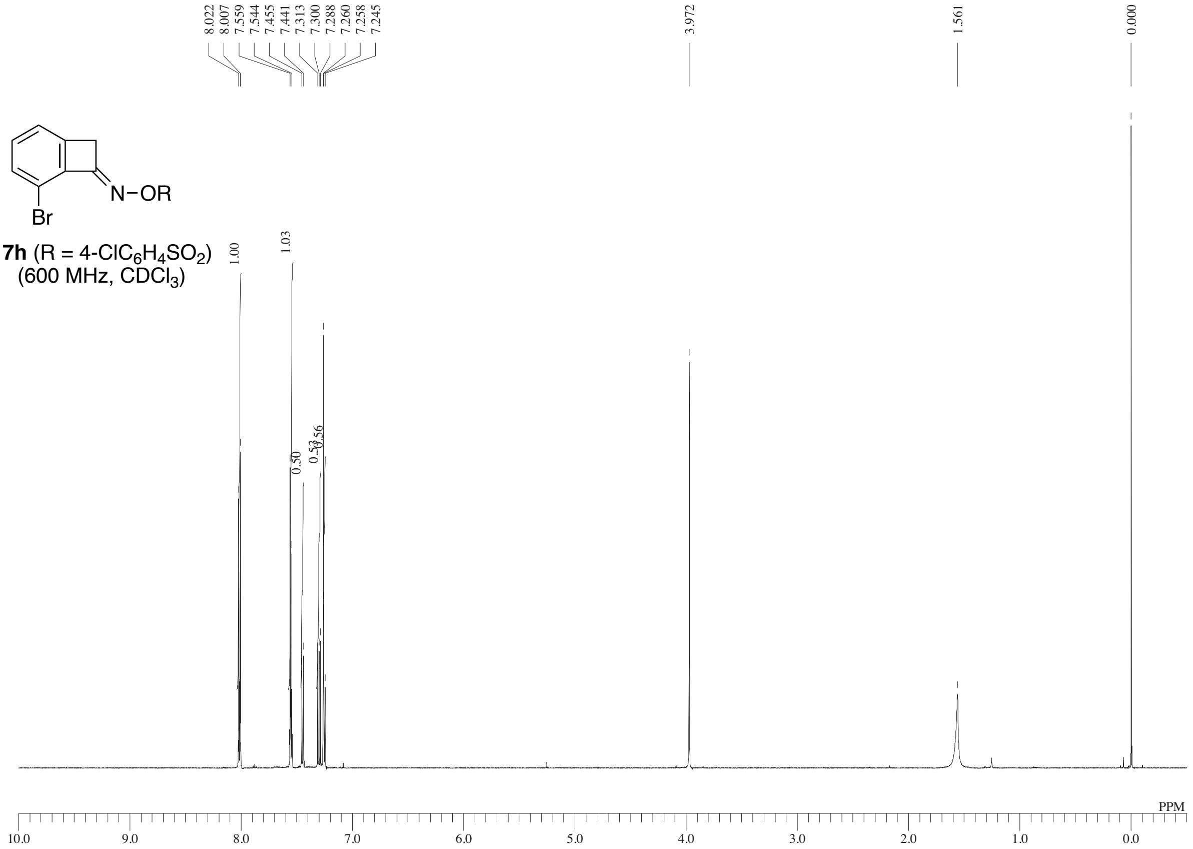

17h (R = 4-ClC$_6$H$_4$SO$_2$)
(600 MHz, CDCl$_3$)

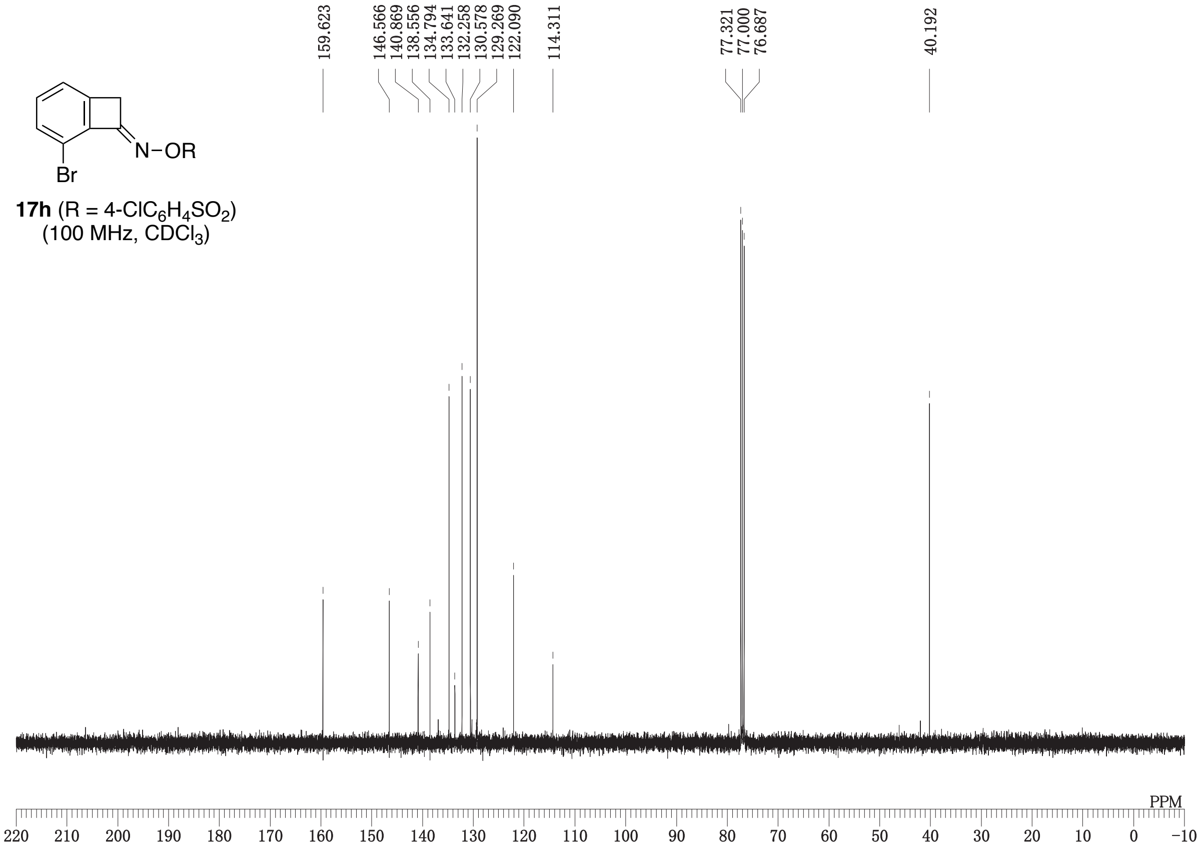

17h (R = 4-ClC$_6$H$_4$SO$_2$)
(100 MHz, CDCl$_3$)

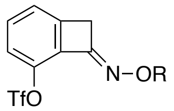

17i (R = 4-ClC$_6$H$_4$SO$_2$)
(600 MHz, CDCl$_3$)

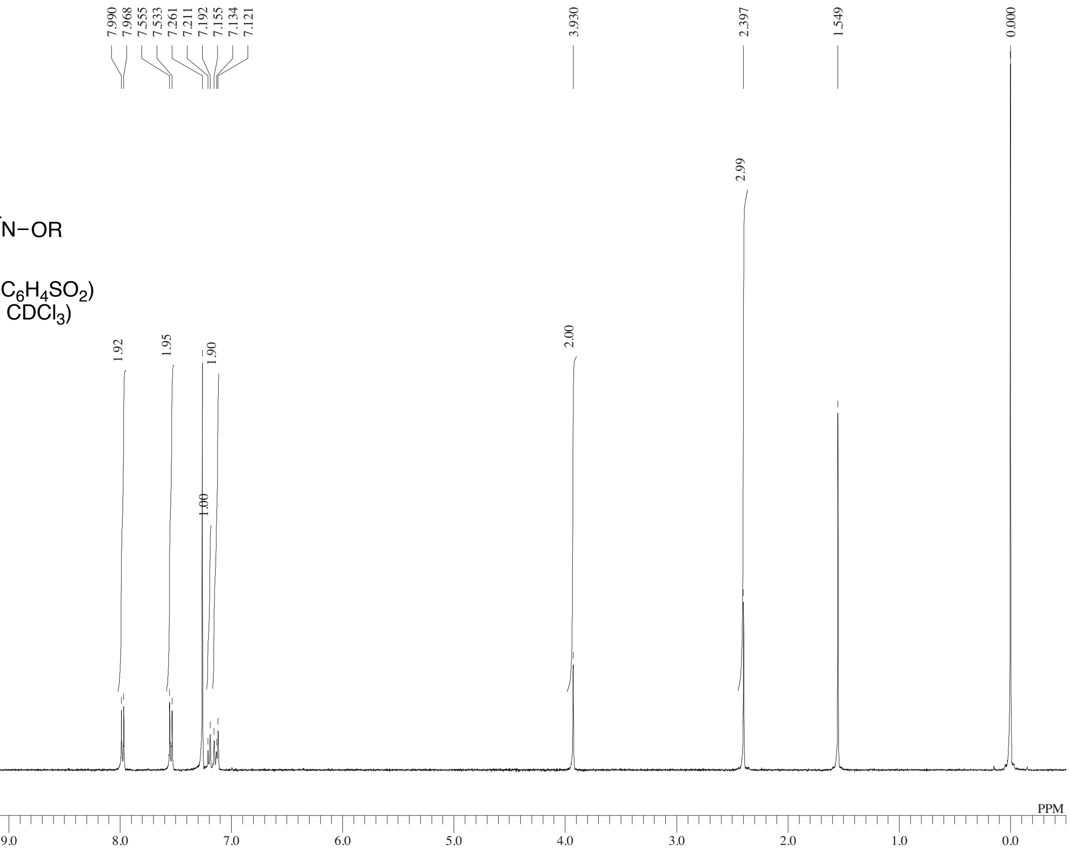

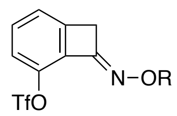

17i (R = 4-ClC$_6$H$_4$SO$_2$)
(150 MHz, CDCl$_3$)

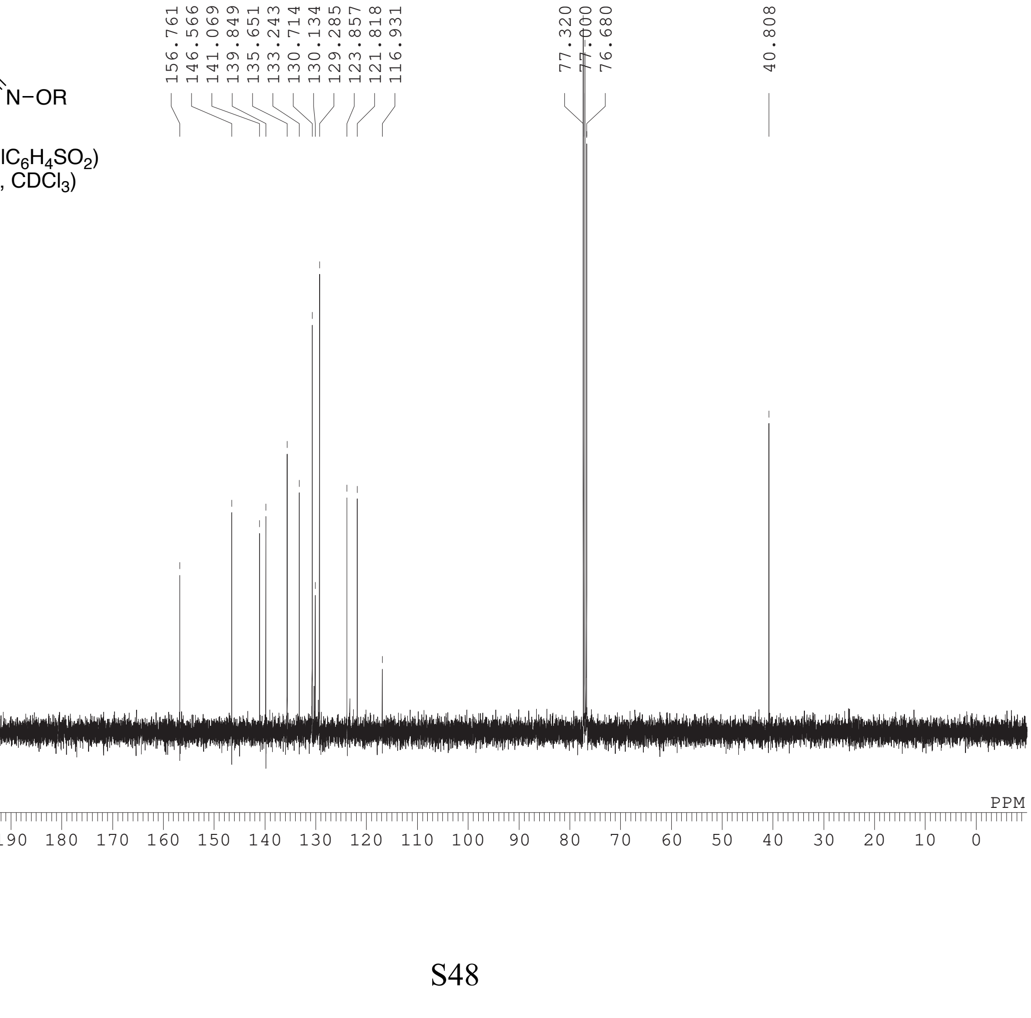

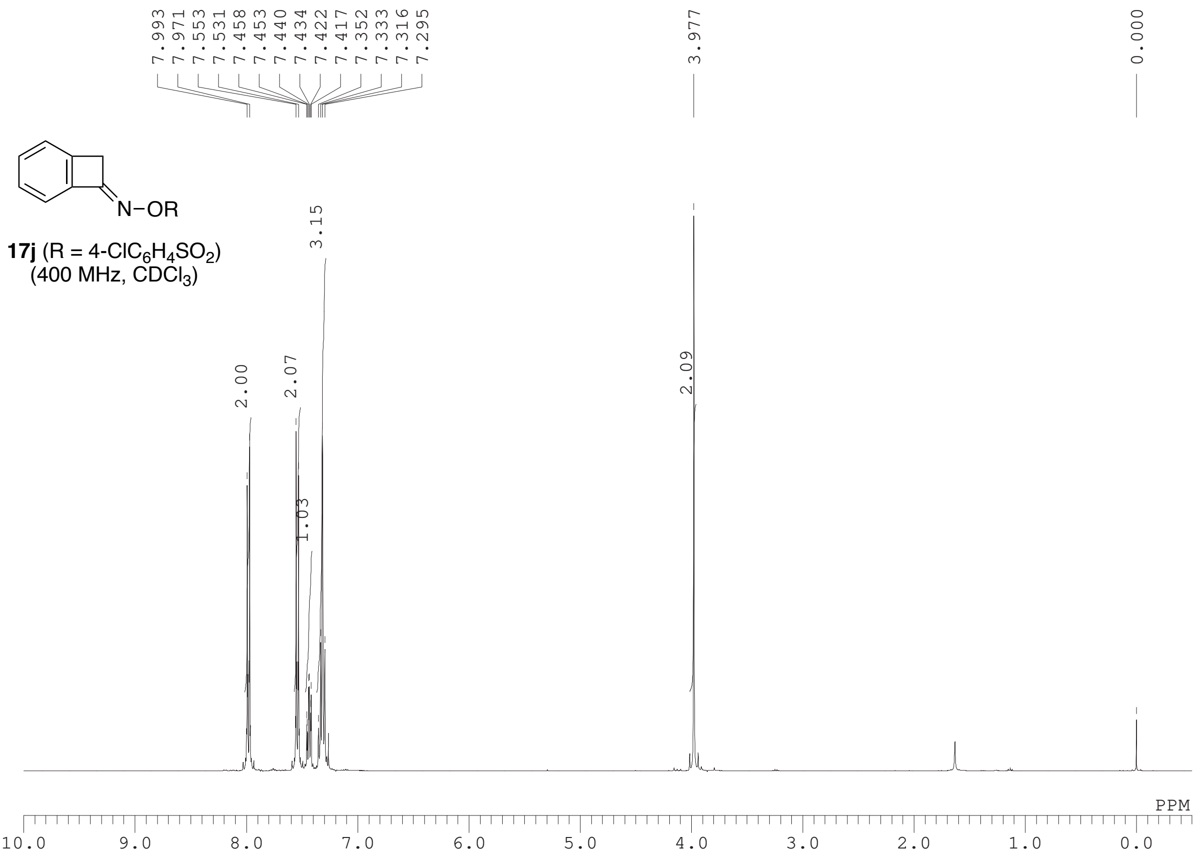
7.993
7.971
7.553
7.531
7.458
7.453
7.440
7.434
7.422
7.417
7.352
7.333
7.316
7.295
3.977
0.000
N–OR
17j (R = 4-ClC$_6$H$_4$SO$_2$)
(400 MHz, CDCl$_3$)
3.15
2.00
2.07
1.03
2.09
PPM
10.0
9.0
8.0
7.0
6.0
5.0
4.0
3.0
2.0
1.0
0.0

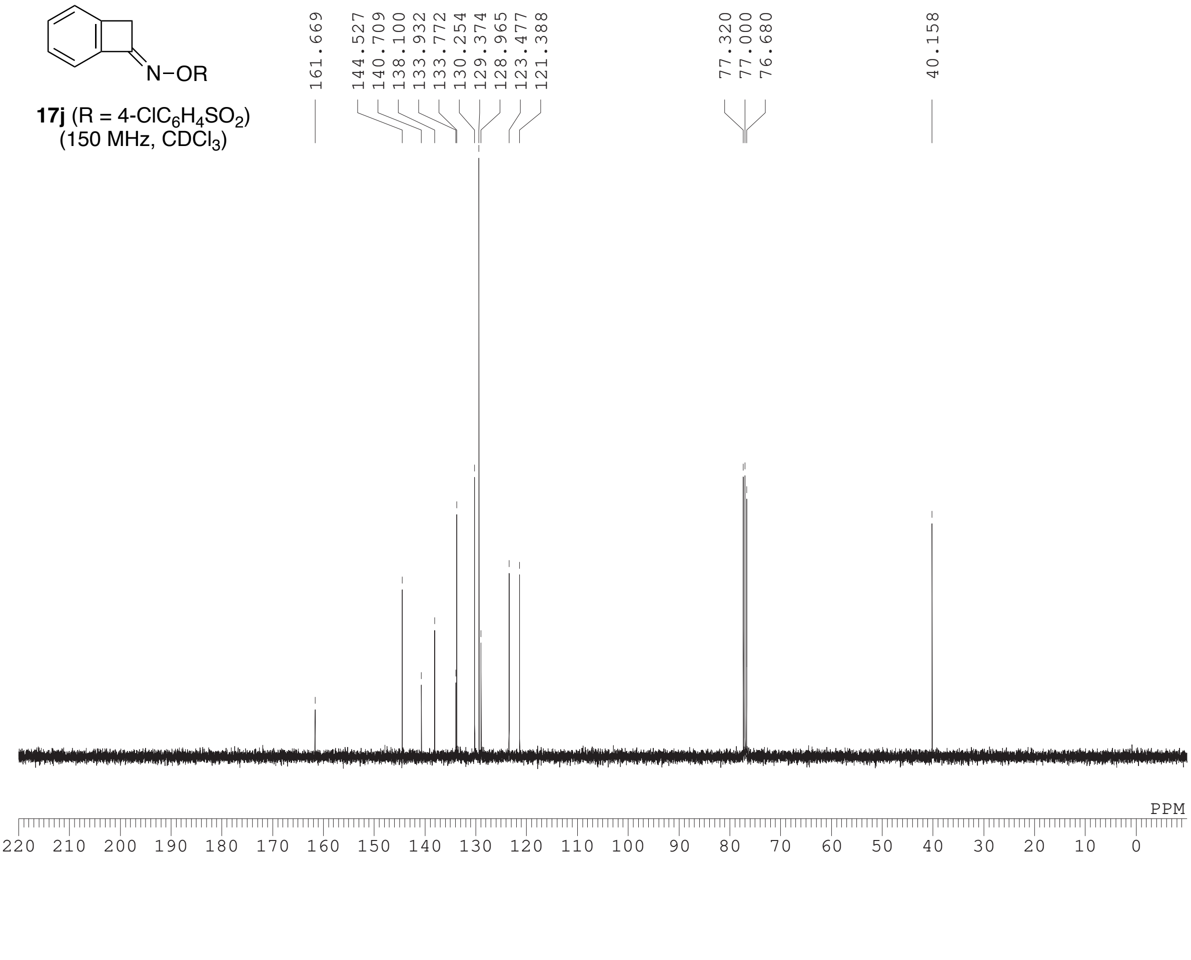
N–OR
17j (R = 4-ClC$_6$H$_4$SO$_2$)
(150 MHz, CDCl$_3$)
161.669
144.527
140.709
138.100
133.932
133.772
130.254
129.374
128.965
123.477
121.388
77.320
77.000
76.680
40.158
PPM
220 210 200 190 180 170 160 150 140 130 120 110 100 90 80 70 60 50 40 30 20 10 0

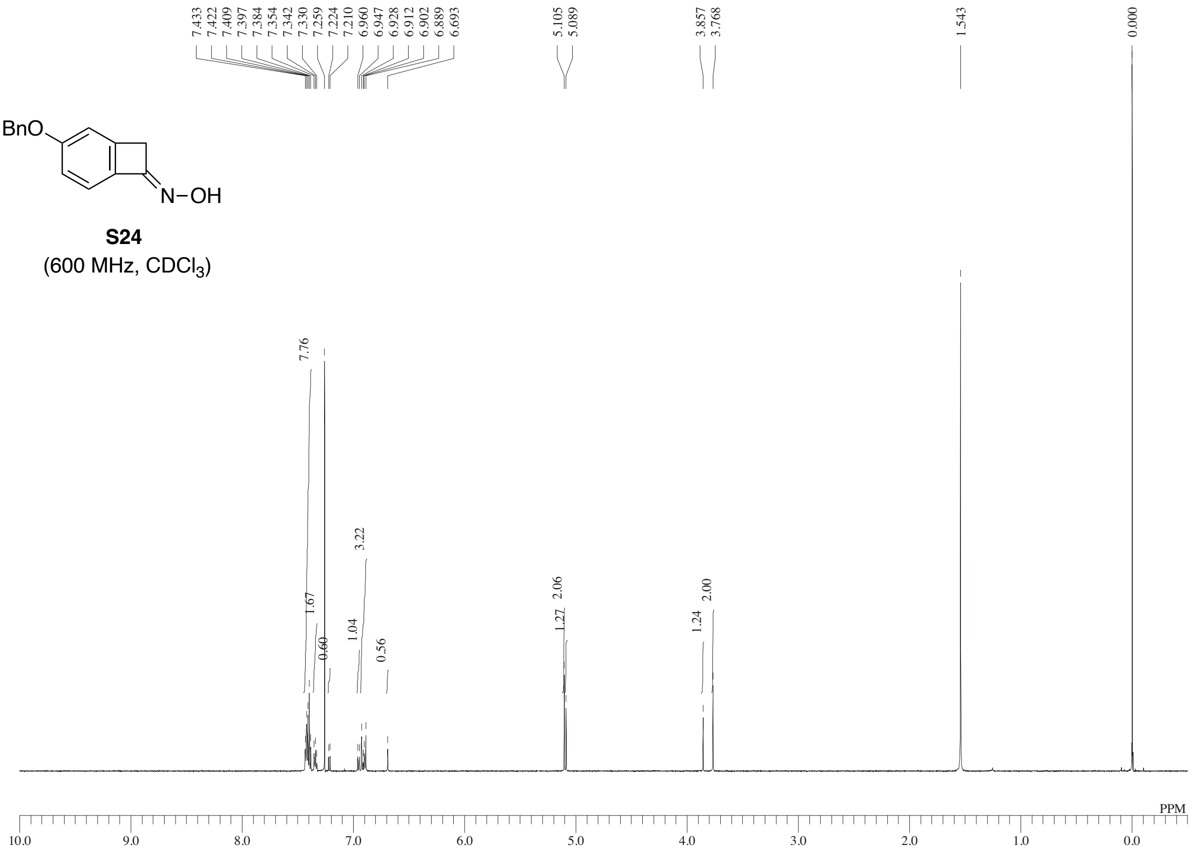
BnO
N–OH
S24
(600 MHz, CDCl3)
7.433 7.422 7.409 7.397 7.384 7.354 7.342 7.330 7.259 7.224 7.210 6.960 6.947 6.928 6.912 6.902 6.889 6.693
5.105 5.089
3.857 3.768
1.543
0.000
7.76 1.67 0.60 1.04 3.22 0.56 1.27 2.06 1.24 2.00
PPM
10.0 9.0 8.0 7.0 6.0 5.0 4.0 3.0 2.0 1.0 0.0

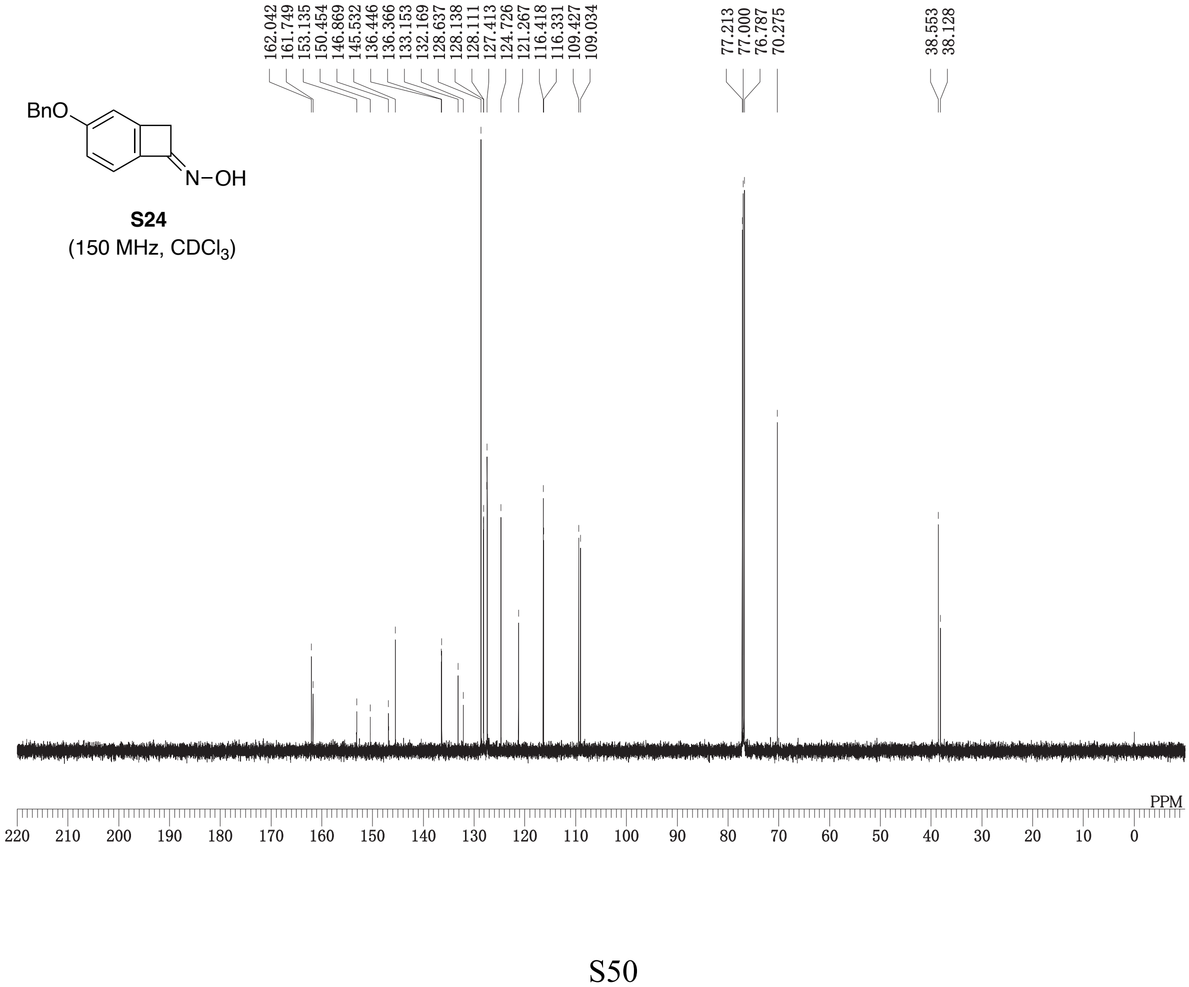
BnO
N–OH
S24
(150 MHz, CDCl3)
162.042 161.749 153.135 150.454 146.869 145.532 136.446 136.366 133.153 132.169 128.637 128.138 128.111 127.413 124.726 121.267 116.418 116.331 109.427 109.034
77.213 77.000 76.787 70.275
38.553 38.128
PPM
220 210 200 190 180 170 160 150 140 130 120 110 100 90 80 70 60 50 40 30 20 10 0

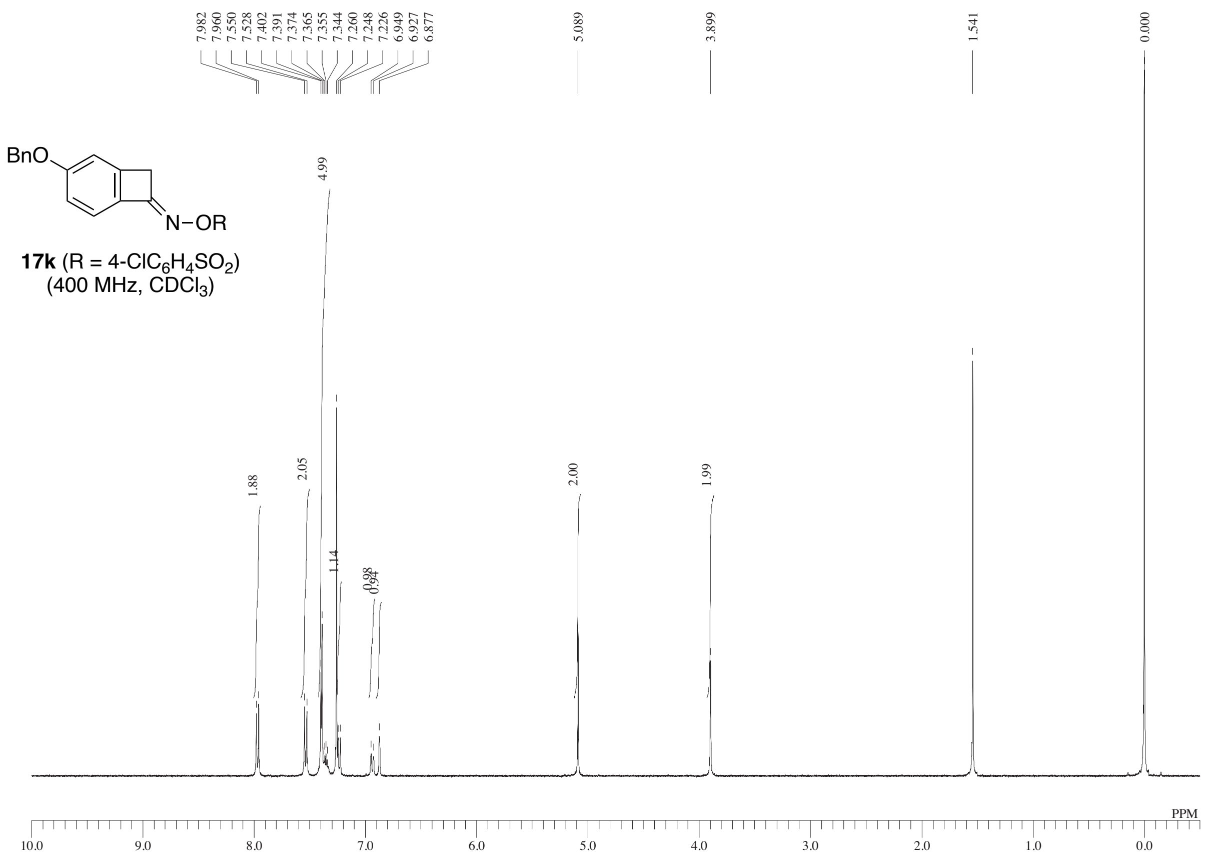
7.982 7.960 7.550 7.528 7.402 7.391 7.374 7.365 7.355 7.344 7.260 7.248 7.226 6.949 6.927 6.877
5.089
3.899
1.541
0.000
BnO
N−OR
17k (R = 4-ClC$_6$H$_4$SO$_2$)
(400 MHz, CDCl$_3$)
4.99
1.88
2.05
1.14
0.98
0.94
2.00
1.99
PPM
10.0 9.0 8.0 7.0 6.0 5.0 4.0 3.0 2.0 1.0 0.0

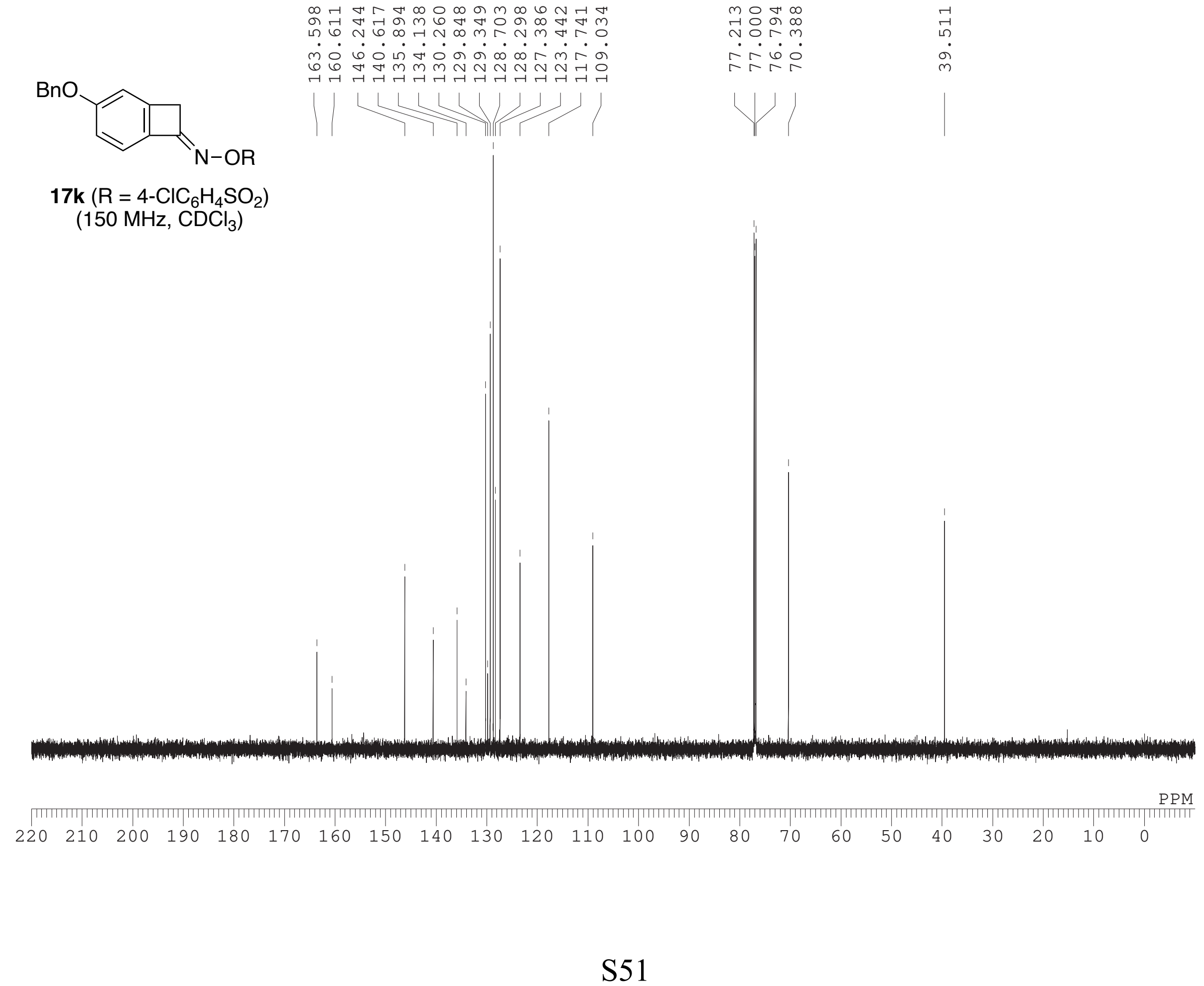
163.598 160.611 146.244 140.617 135.894 134.138 130.260 129.848 129.349 128.703 128.298 127.386 123.442 117.741 109.034
77.213 77.000 76.794 70.388
39.511
BnO
N−OR
17k (R = 4-ClC$_6$H$_4$SO$_2$)
(150 MHz, CDCl$_3$)
PPM
220 210 200 190 180 170 160 150 140 130 120 110 100 90 80 70 60 50 40 30 20 10 0

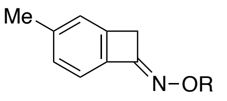

17l (R = 4-ClC$_6$H$_4$SO$_2$)
(400 MHz, CDCl$_3$)

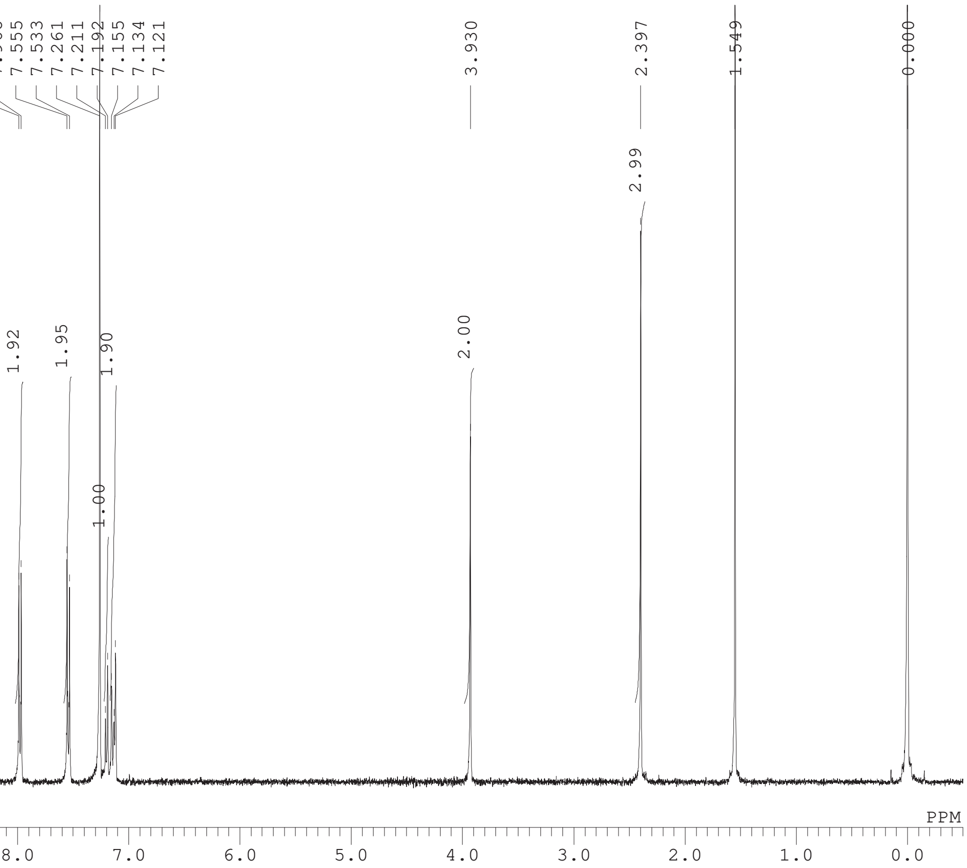

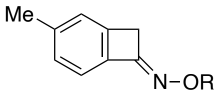

17l (R = 4-ClC$_6$H$_4$SO$_2$)
(150 MHz, CDCl$_3$)

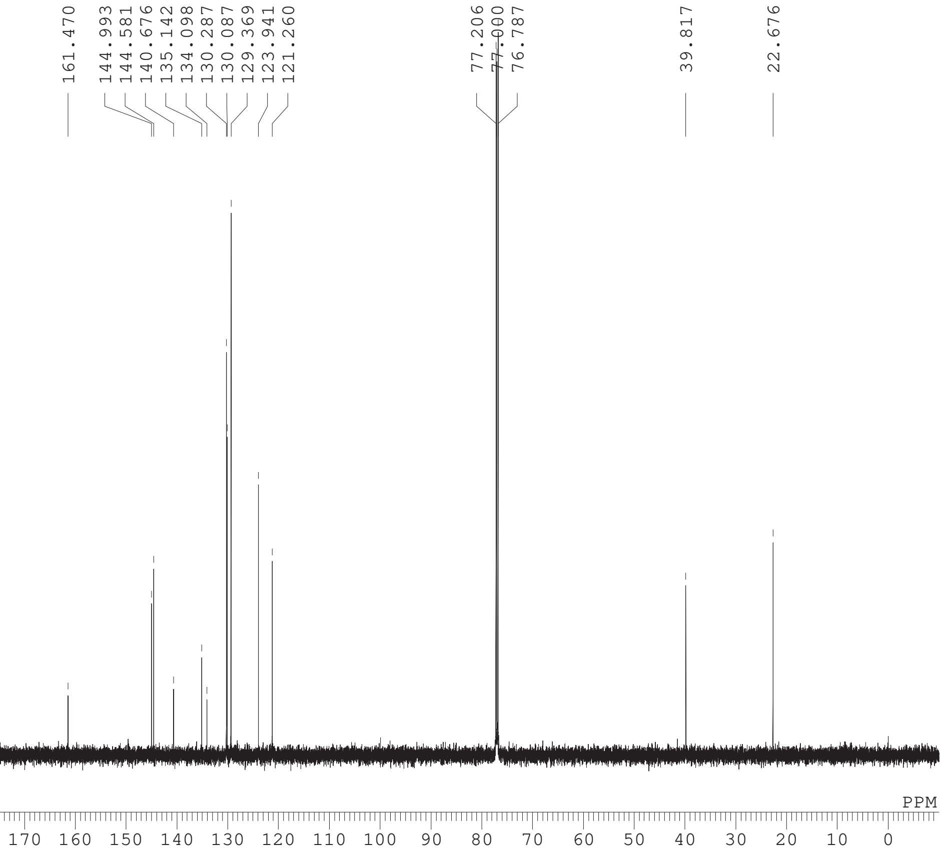

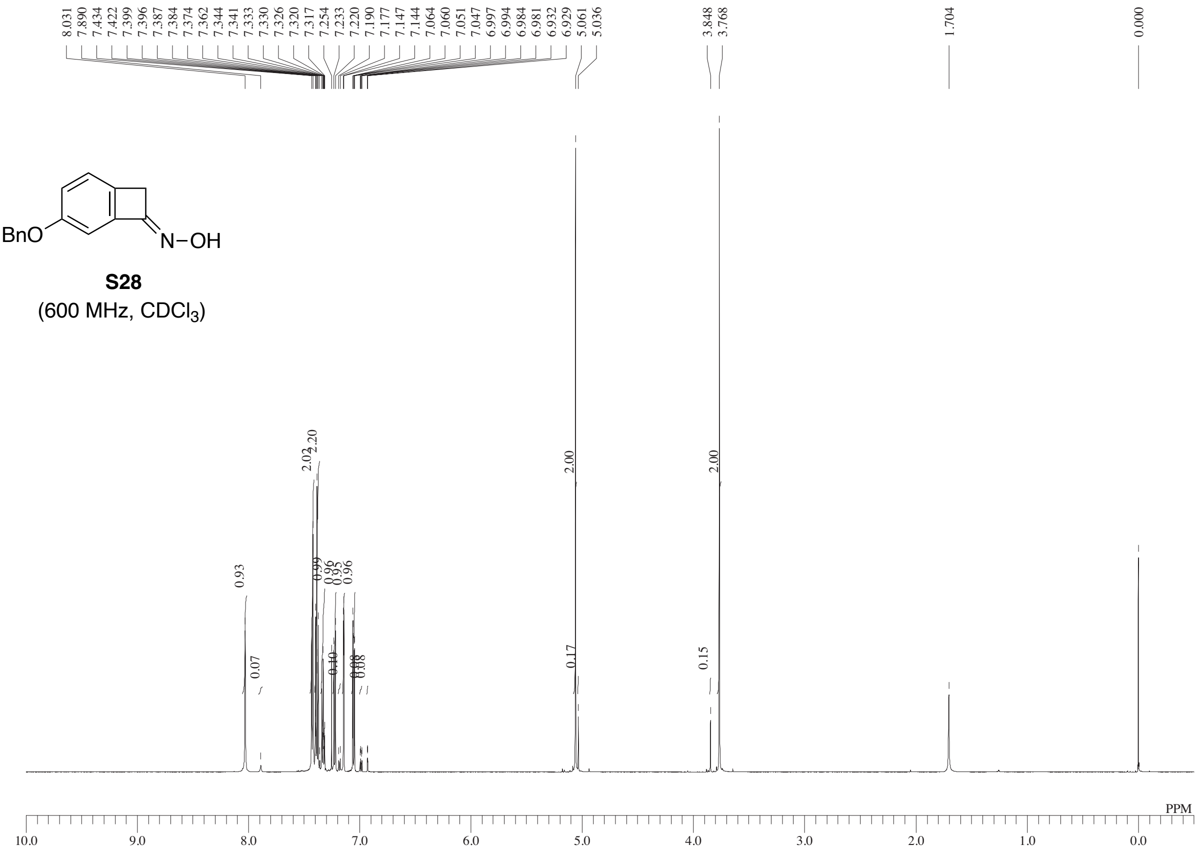
8.031
7.890
7.434
7.422
7.399
7.396
7.387
7.384
7.374
7.362
7.344
7.341
7.333
7.330
7.326
7.320
7.317
7.254
7.233
7.220
7.190
7.177
7.147
7.144
7.064
7.060
7.051
7.047
6.997
6.994
6.984
6.981
6.932
6.929
5.061
5.036
3.848
3.768
1.704
0.000
BnO
N−OH
S28
(600 MHz, CDCl3)
0.93
0.07
2.02
2.20
0.99
0.96
0.95
0.96
0.10
0.08
0.08
2.00
0.17
2.00
0.15
PPM
10.0
9.0
8.0
7.0
6.0
5.0
4.0
3.0
2.0
1.0
0.0

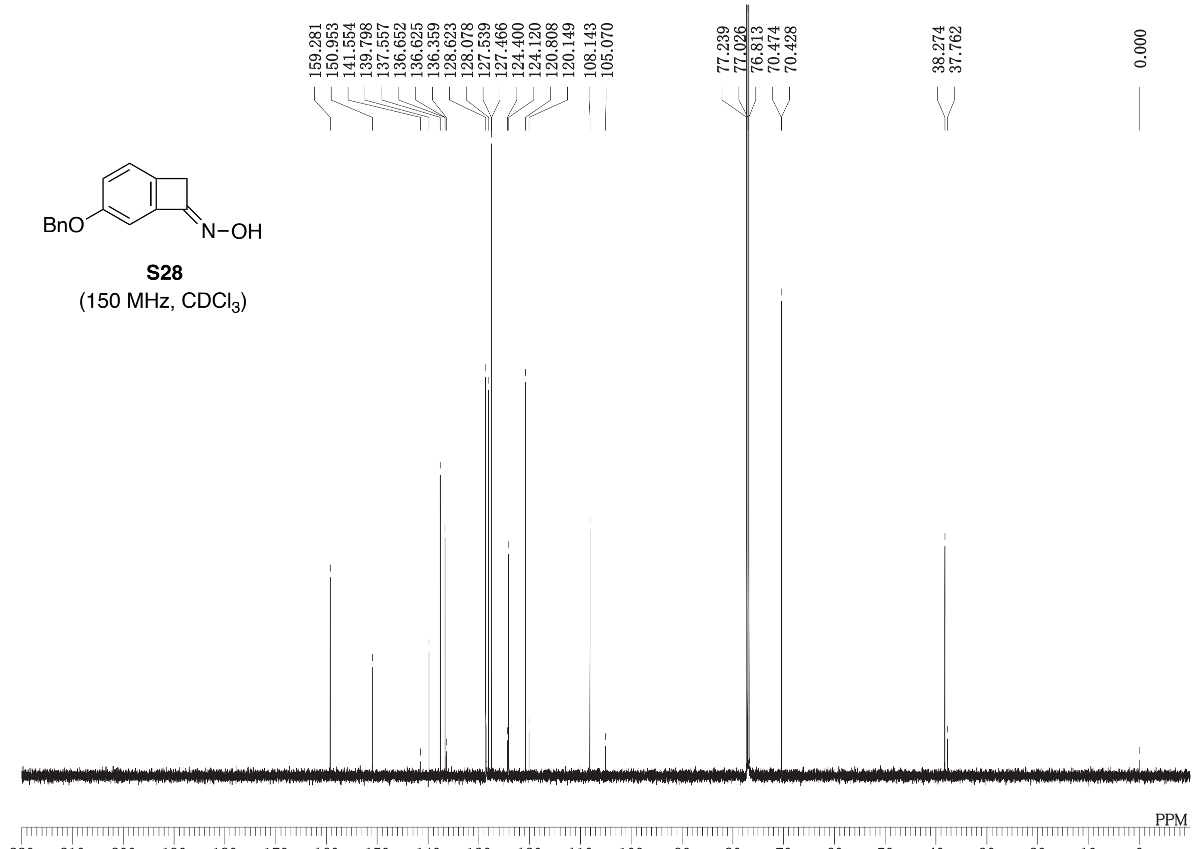
159.281
150.953
141.554
139.798
137.557
136.652
136.625
136.359
128.623
128.078
127.539
127.466
124.400
124.120
120.808
120.149
108.143
105.070
77.239
77.026
76.813
70.474
70.428
38.274
37.762
0.000
BnO
N−OH
S28
(150 MHz, CDCl3)
PPM
220
210
200
190
180
170
160
150
140
130
120
110
100
90
80
70
60
50
40
30
20
10
0

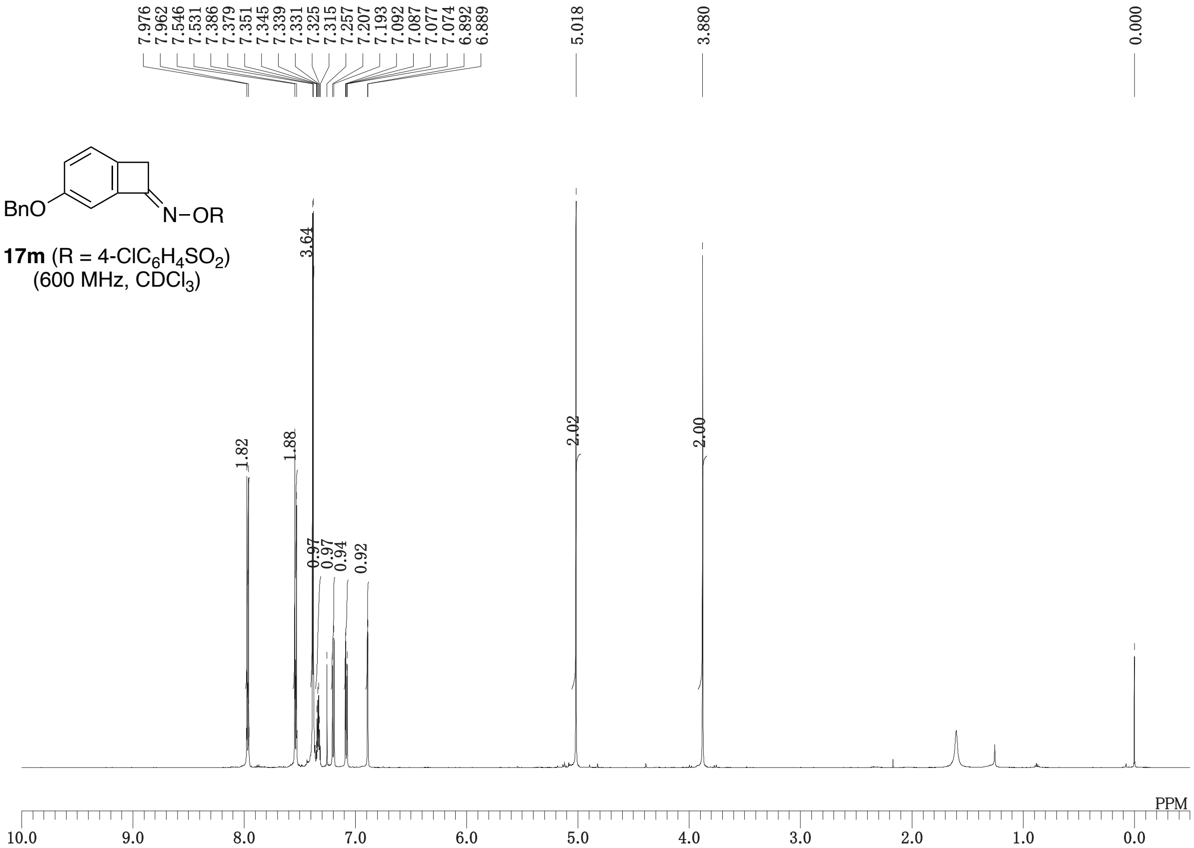

17m (R = 4-ClC$_6$H$_4$SO$_2$)
(600 MHz, CDCl$_3$)

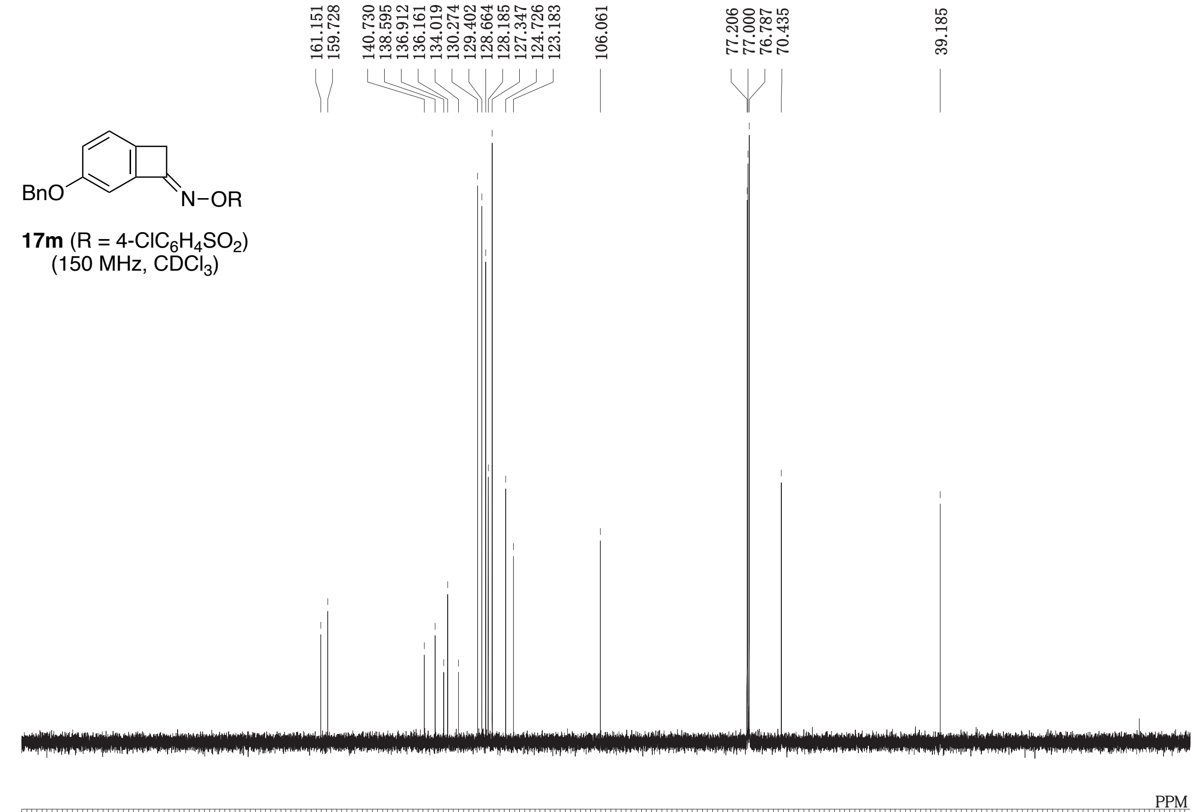

17m (R = 4-ClC$_6$H$_4$SO$_2$)
(150 MHz, CDCl$_3$)

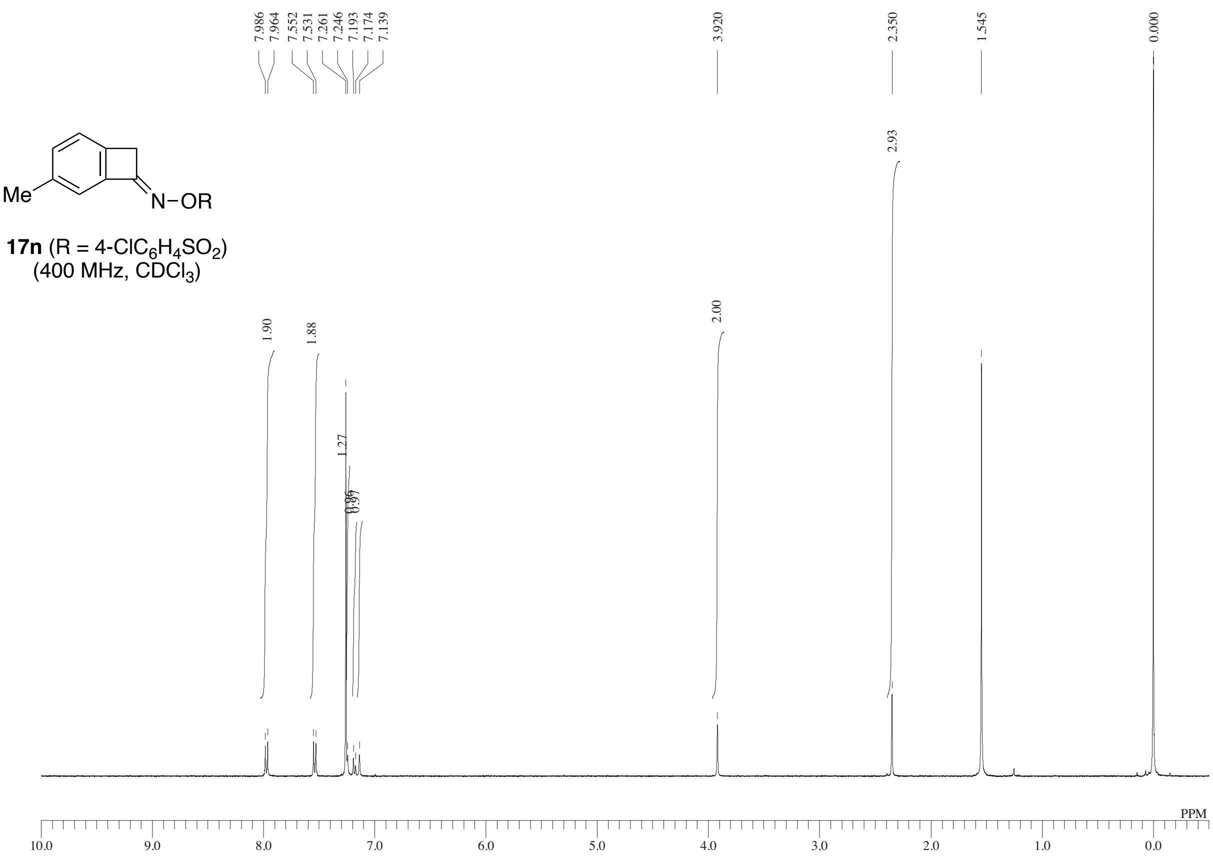

17n (R = 4-ClC$_6$H$_4$SO$_2$)
(400 MHz, CDCl$_3$)

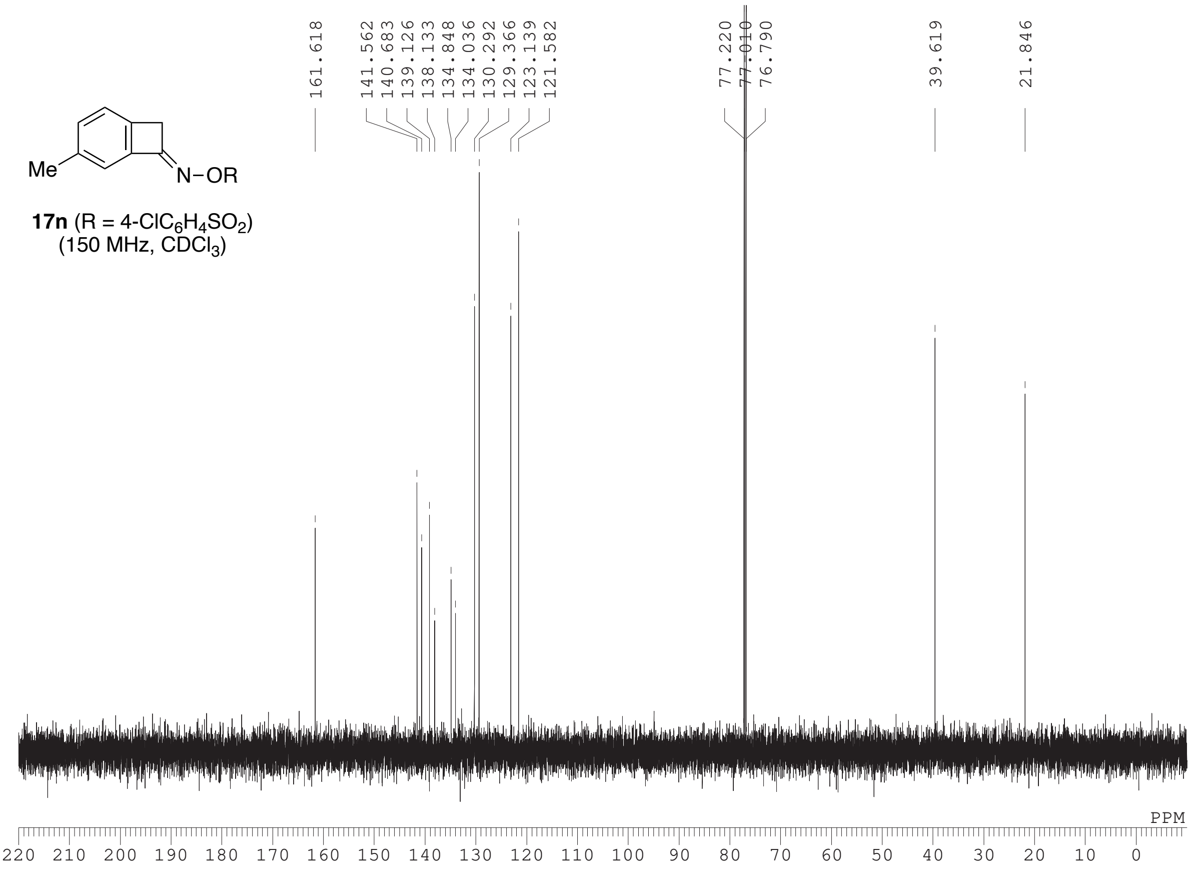

17n (R = 4-ClC$_6$H$_4$SO$_2$)
(150 MHz, CDCl$_3$)

17o (R = 4-ClC$_6$H$_4$SO$_2$)
(600 MHz, CDCl$_3$)

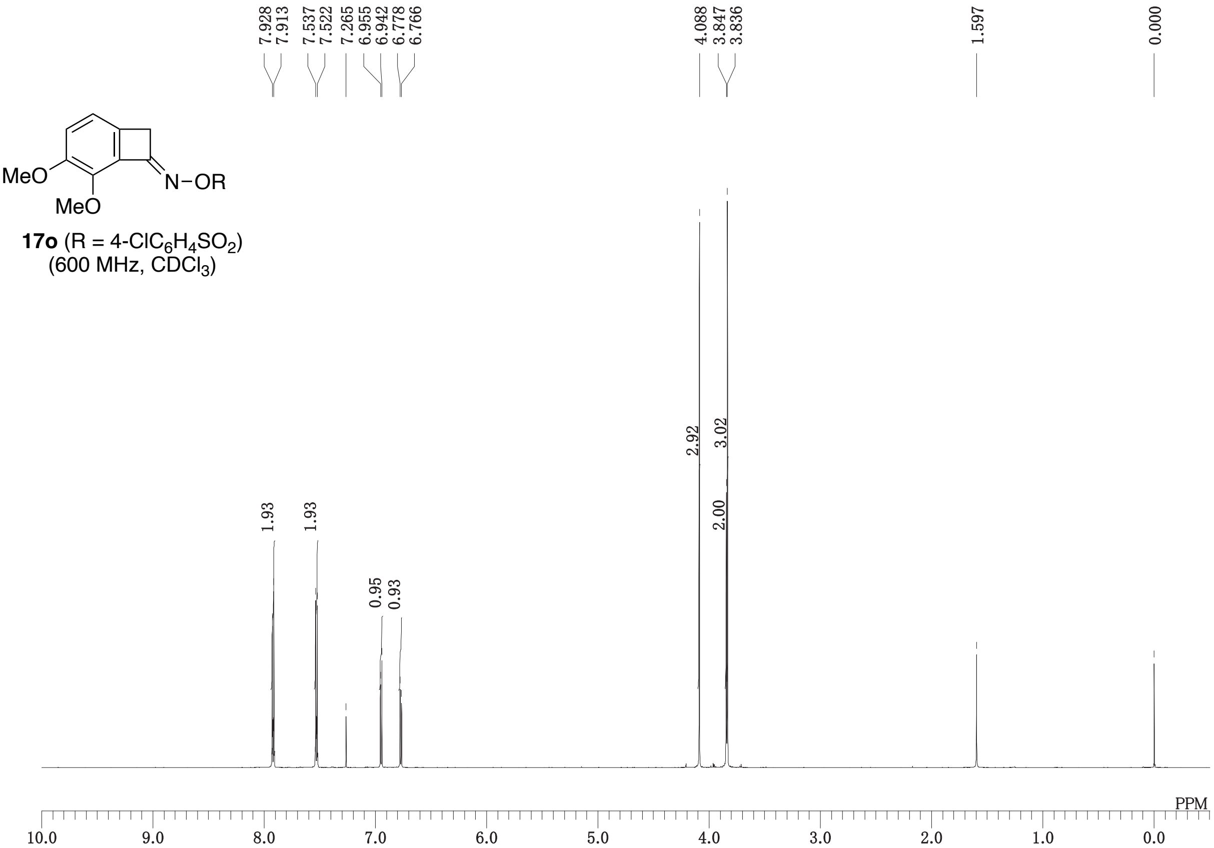

17o (R = 4-ClC$_6$H$_4$SO$_2$)
(150 MHz, CDCl$_3$)

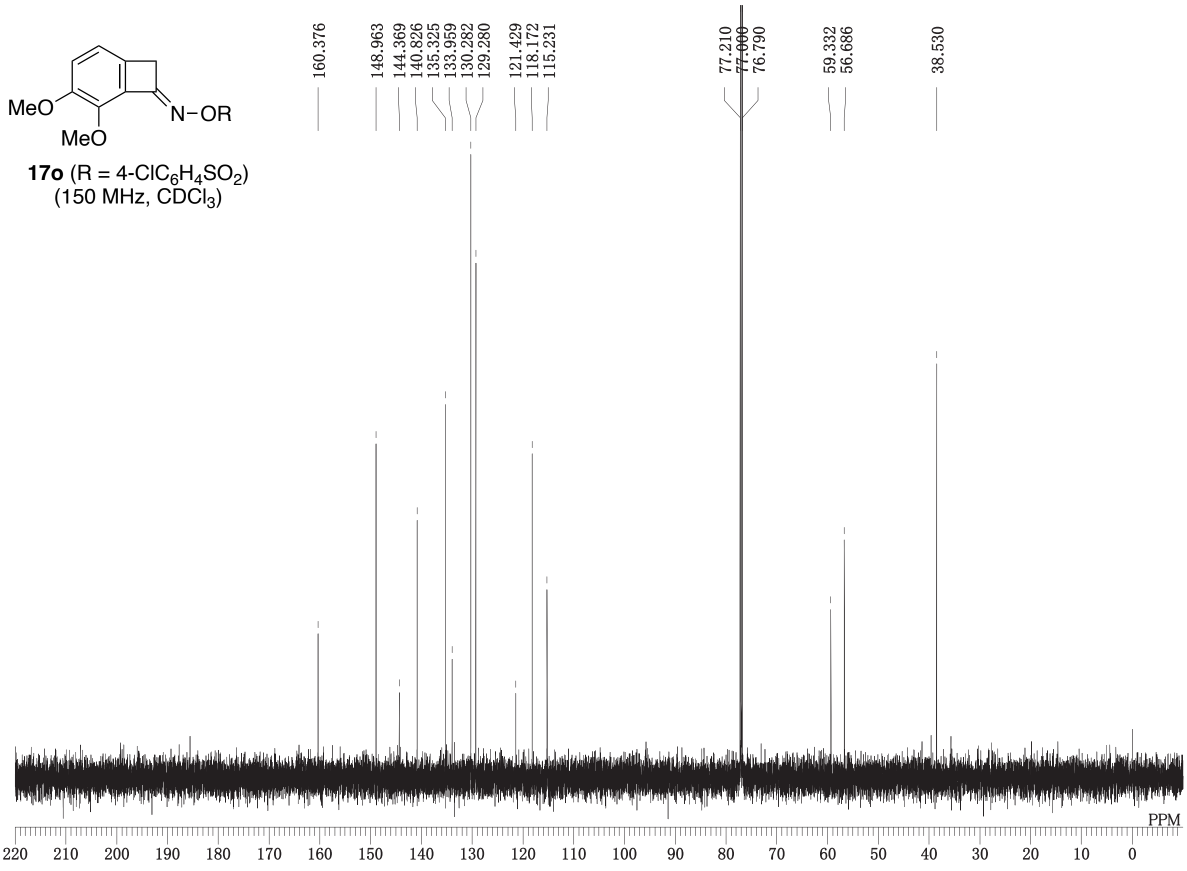

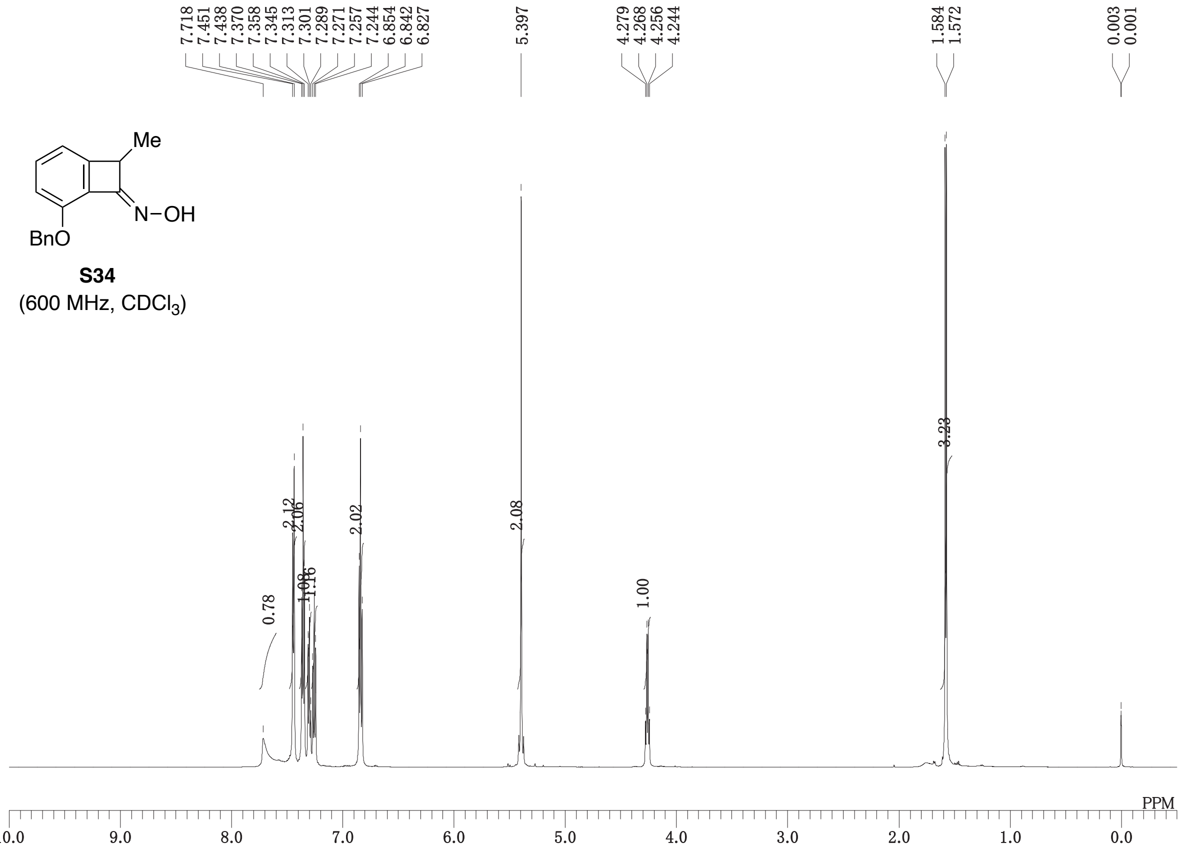
7.718
7.451
7.438
7.370
7.358
7.345
7.313
7.301
7.289
7.271
7.257
7.244
6.854
6.842
6.827
5.397
4.279
4.268
4.256
4.244
1.584
1.572
0.003
0.001
Me
N−OH
BnO
S34
(600 MHz, CDCl3)
0.78
2.12
2.06
1.08
1.16
2.02
2.08
1.00
3.23
PPM
10.0
9.0
8.0
7.0
6.0
5.0
4.0
3.0
2.0
1.0
0.0

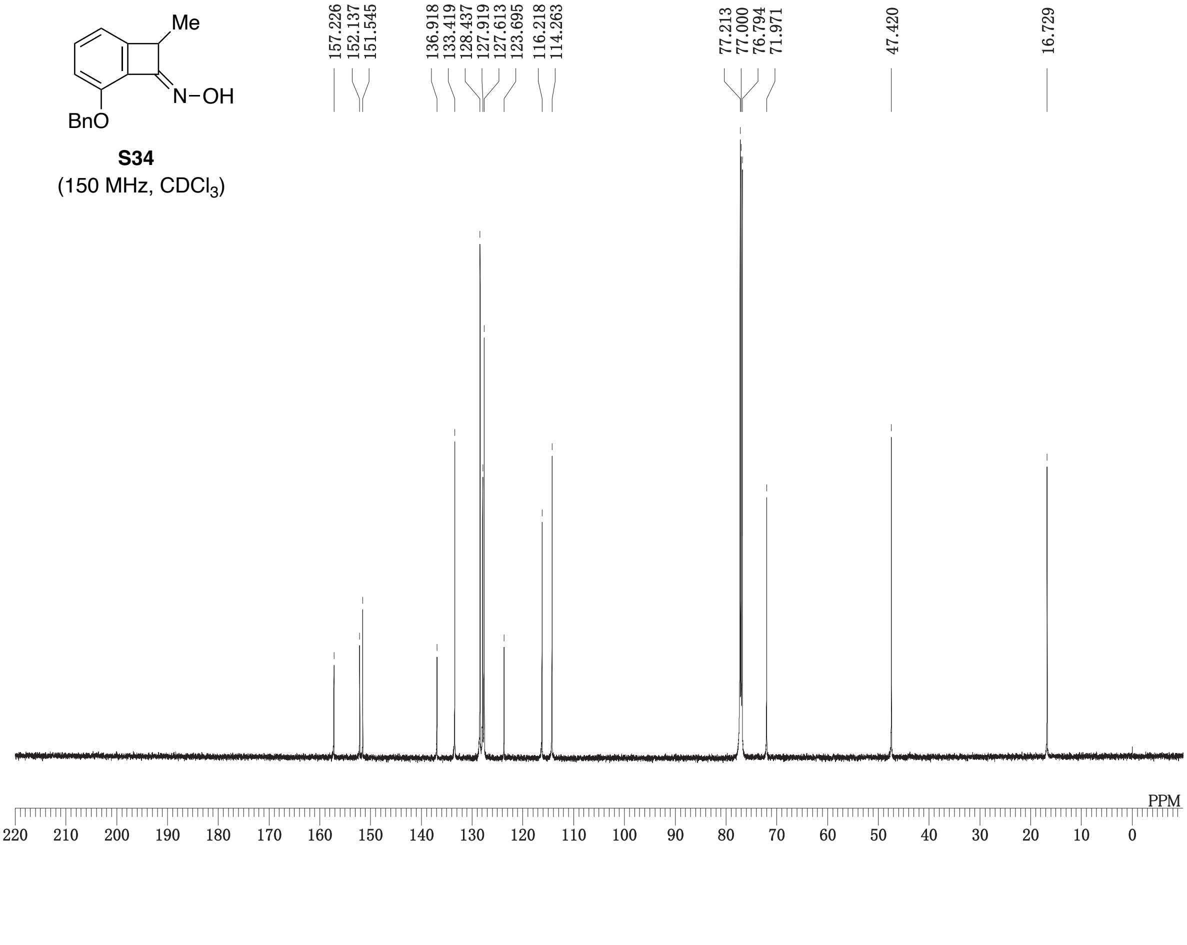
Me
N−OH
BnO
S34
(150 MHz, CDCl3)
157.226
152.137
151.545
136.918
133.419
128.437
127.919
127.613
123.695
116.218
114.263
77.213
77.000
76.794
71.971
47.420
16.729
PPM
220
210
200
190
180
170
160
150
140
130
120
110
100
90
80
70
60
50
40
30
20
10
0

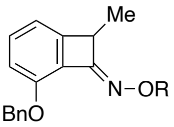

17p (R = 4-$ClC_6H_4SO_2$)
(600 MHz, $CDCl_3$)

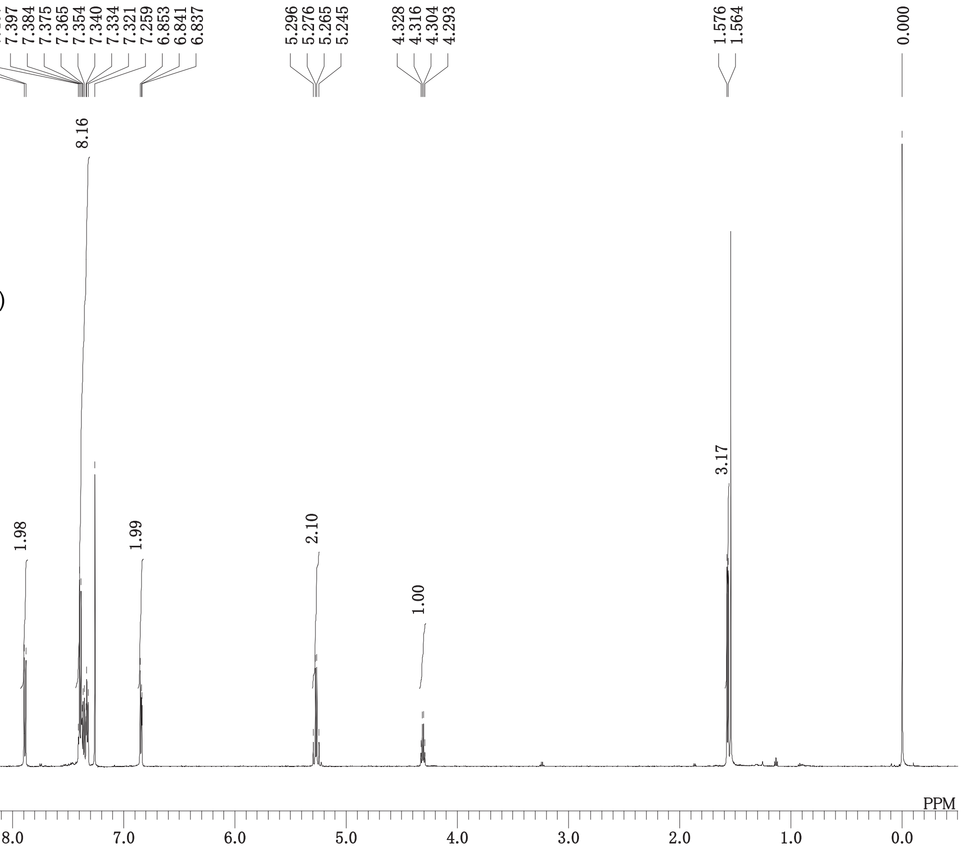

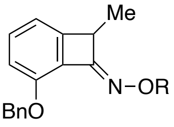

17p (R = 4-$ClC_6H_4SO_2$)
(150 MHz, $CDCl_3$)

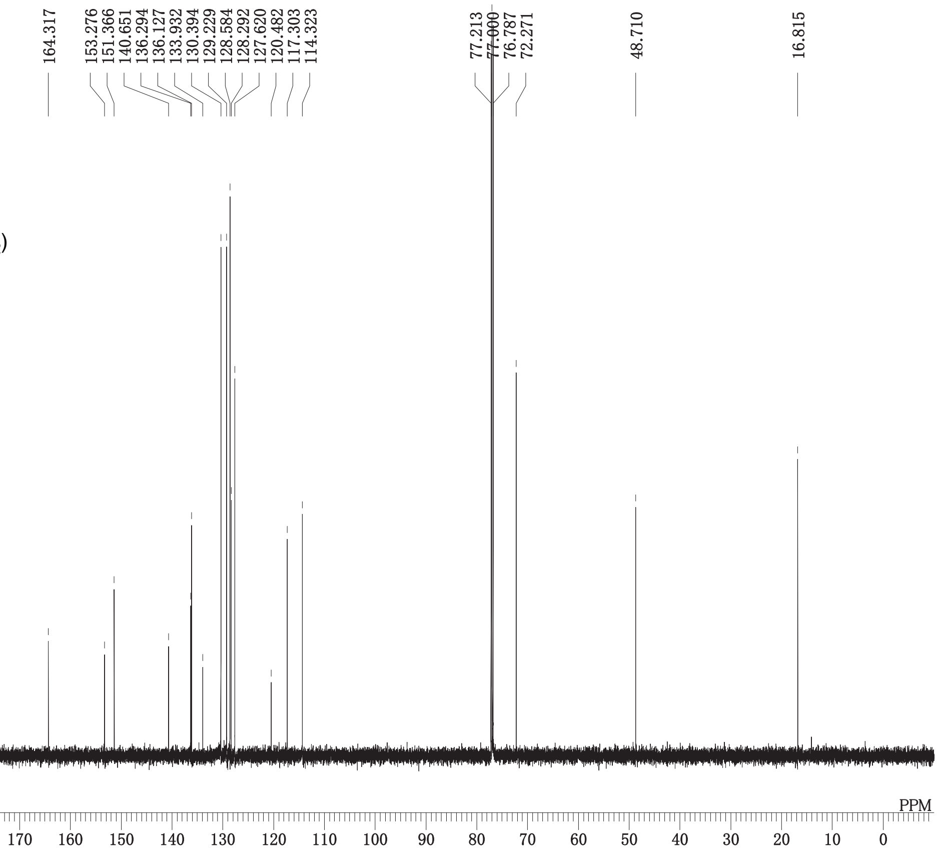

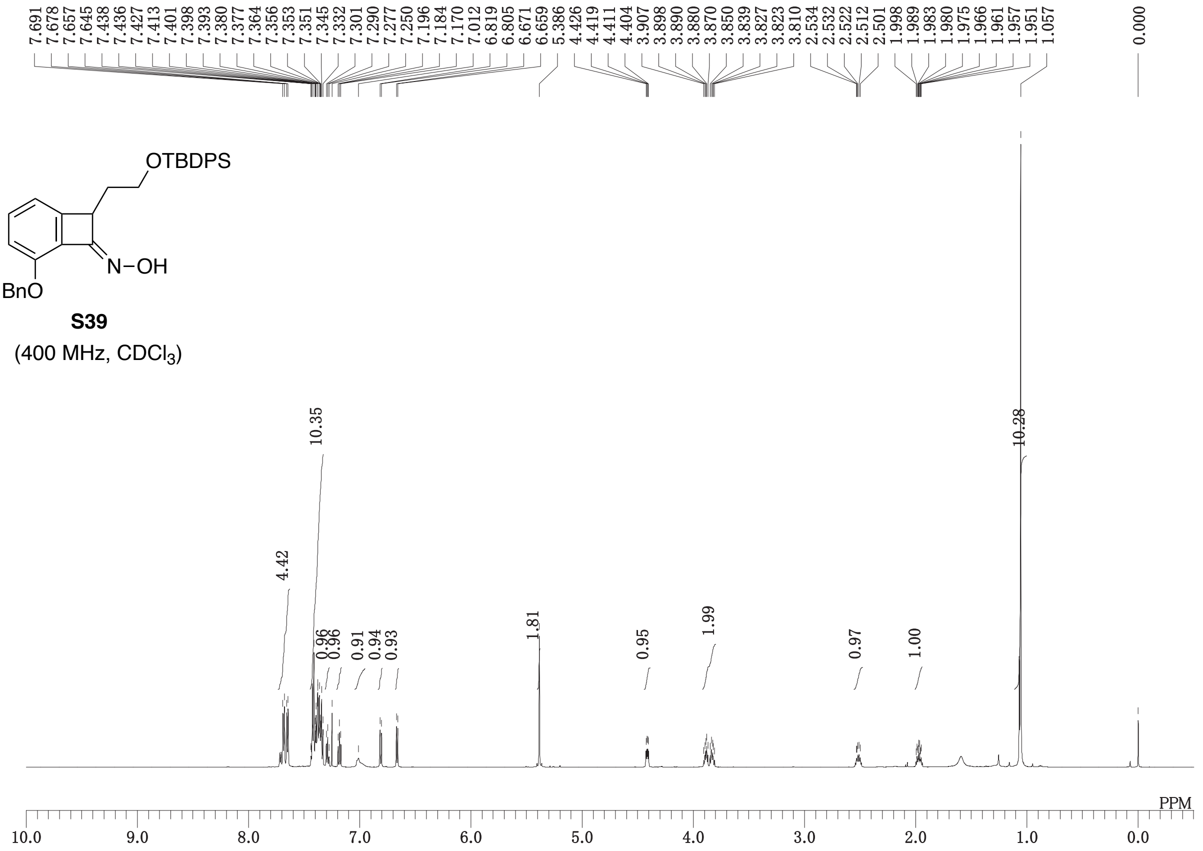

S39

(400 MHz, $CDCl_3$)

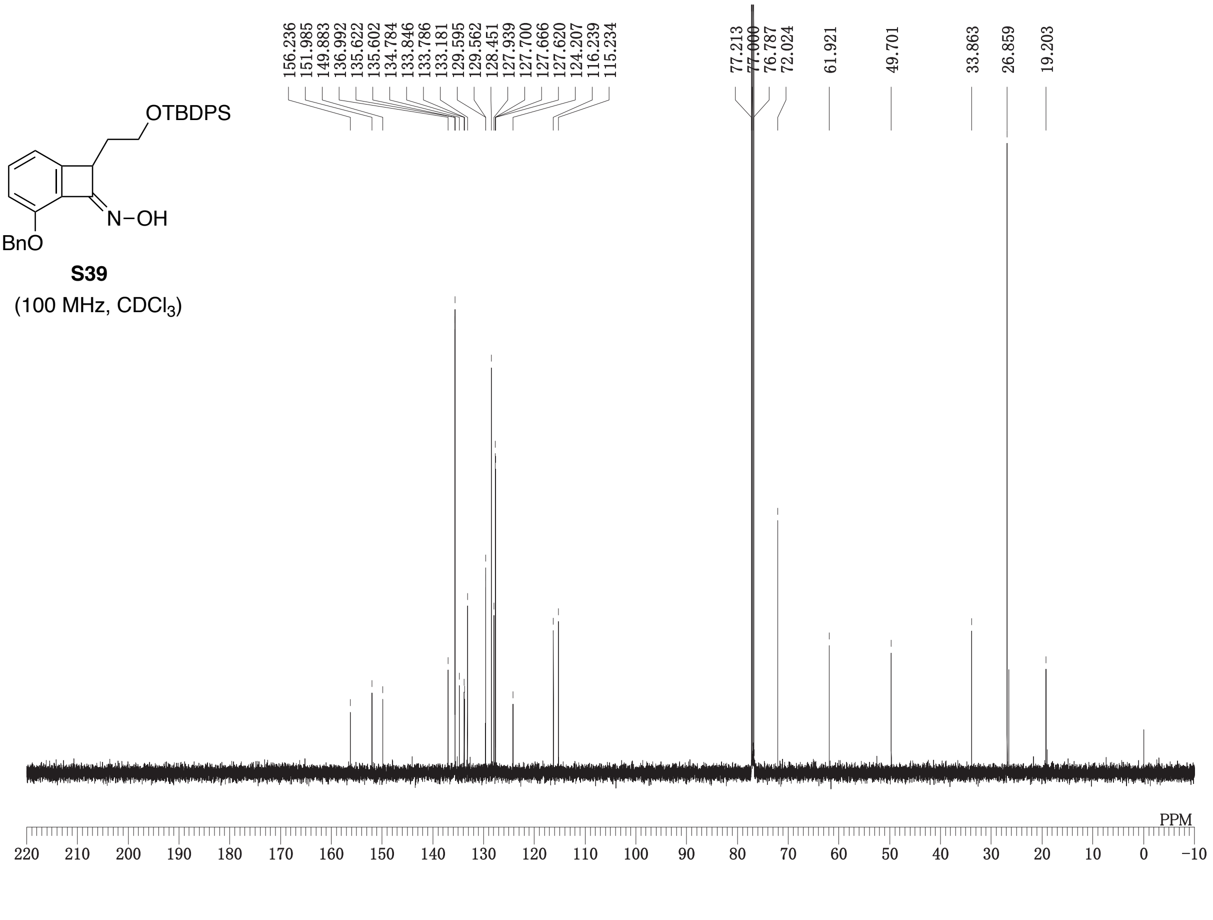

S39

(100 MHz, $CDCl_3$)

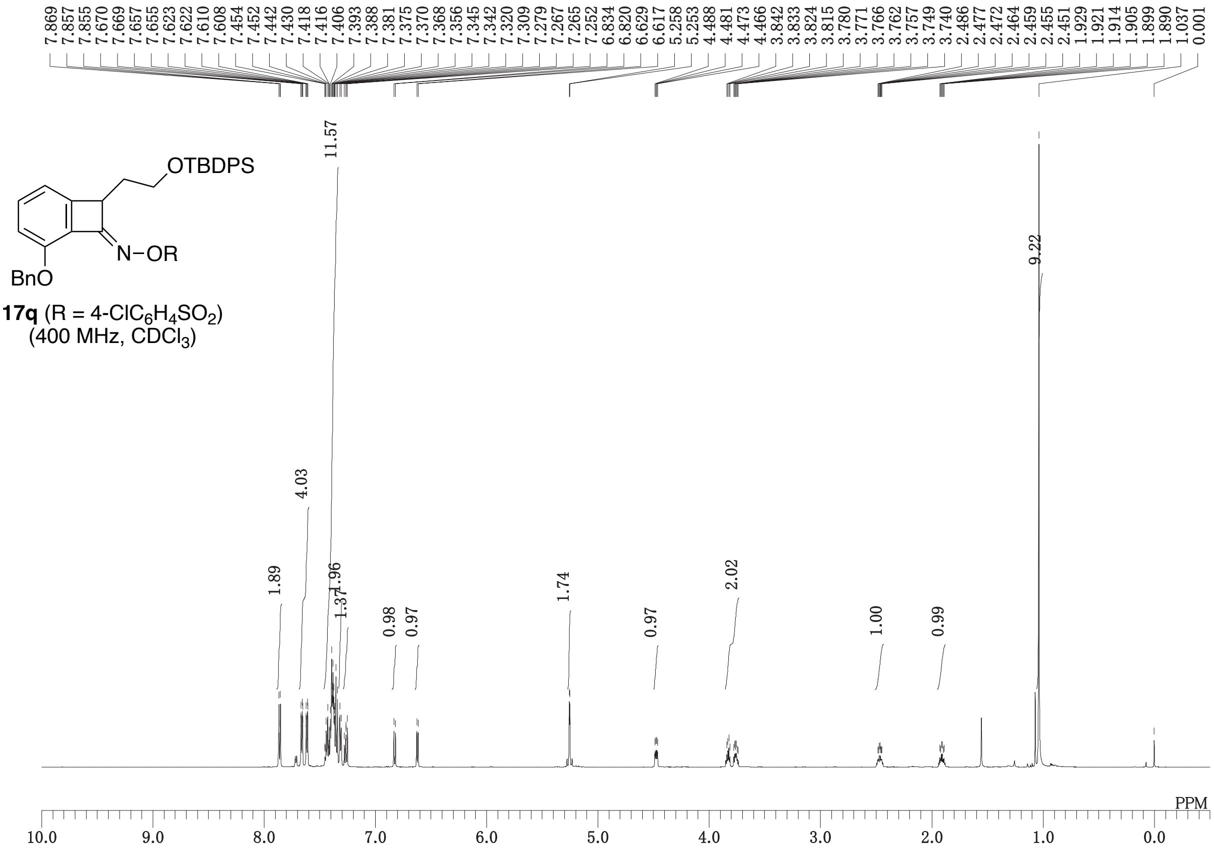
OTBDPS
N–OR
BnO
17q (R = 4-ClC$_6$H$_4$SO$_2$)
(400 MHz, CDCl$_3$)
7.869 7.857 7.670 7.669 7.657 7.655 7.623 7.622 7.610 7.608 7.454 7.452 7.442 7.430 7.418 7.416 7.406 7.393 7.388 7.381 7.375 7.370 7.368 7.356 7.345 7.342 7.320 7.309 7.279 7.267 7.265 7.252 6.834 6.820 6.629 6.617 5.258 5.253 4.488 4.481 4.473 4.466 3.842 3.833 3.824 3.815 3.780 3.771 3.766 3.762 3.757 3.749 3.740 2.486 2.477 2.472 2.464 2.459 2.455 2.451 1.929 1.921 1.914 1.905 1.899 1.890 1.037 0.001
11.57
9.22
4.03
1.89
1.96
1.37
0.98
0.97
1.74
0.97
2.02
1.00
0.99
PPM
10.0 9.0 8.0 7.0 6.0 5.0 4.0 3.0 2.0 1.0 0.0

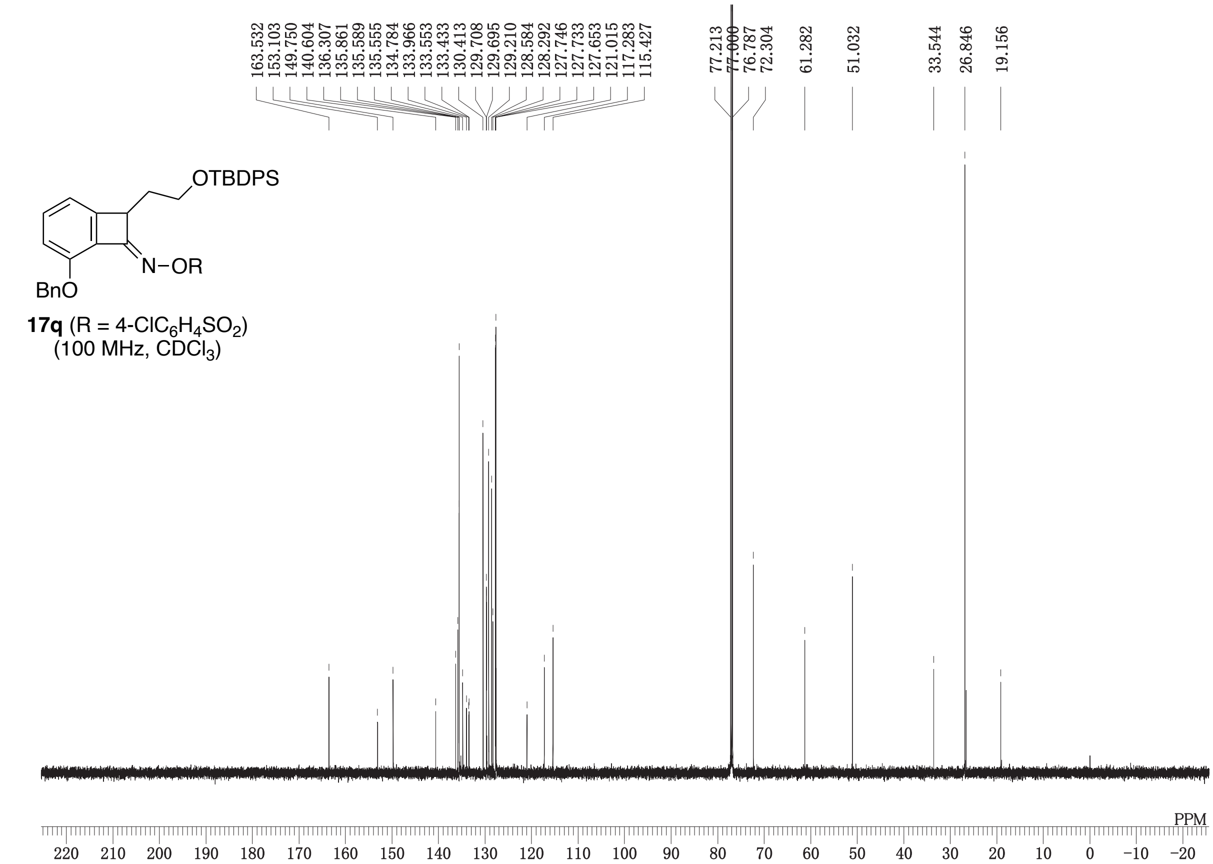
163.532 153.103 149.750 140.604 136.307 135.861 135.589 135.555 134.784 133.966 133.553 133.433 130.413 129.708 129.695 129.210 128.584 128.292 127.746 127.733 127.653 121.015 117.283 115.427
77.213 77.000 76.787 72.304
61.282
51.032
33.544
26.846
19.156
OTBDPS
N–OR
BnO
17q (R = 4-ClC$_6$H$_4$SO$_2$)
(100 MHz, CDCl$_3$)
PPM
220 210 200 190 180 170 160 150 140 130 120 110 100 90 80 70 60 50 40 30 20 10 0 −10 −20

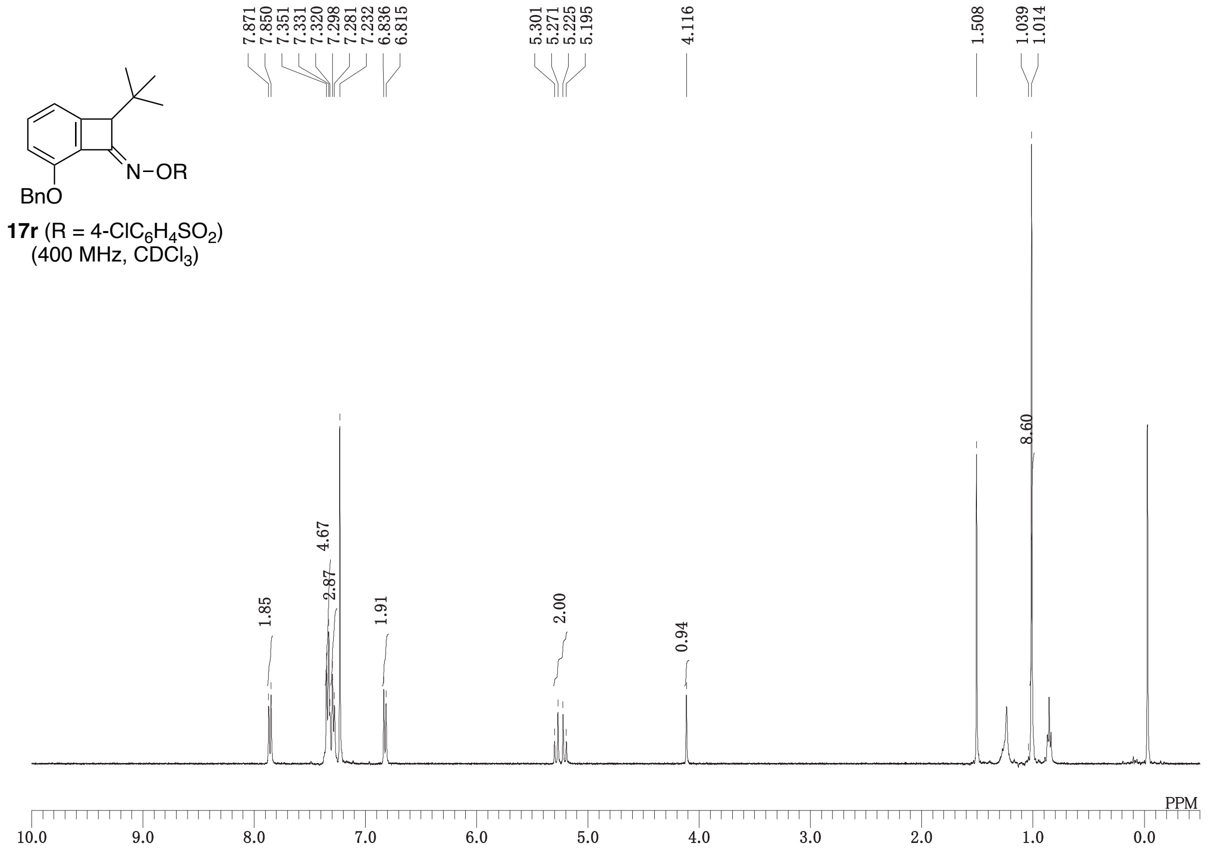
BnO
N−OR
17r (R = 4-ClC$_6$H$_4$SO$_2$)
(400 MHz, CDCl$_3$)
7.871 7.850 7.351 7.331 7.320 7.298 7.281 7.232 6.836 6.815
5.301 5.271 5.225 5.195
4.116
1.508
1.039 1.014
1.85 2.87 4.67 1.91 2.00 0.94 8.60
PPM
10.0 9.0 8.0 7.0 6.0 5.0 4.0 3.0 2.0 1.0 0.0

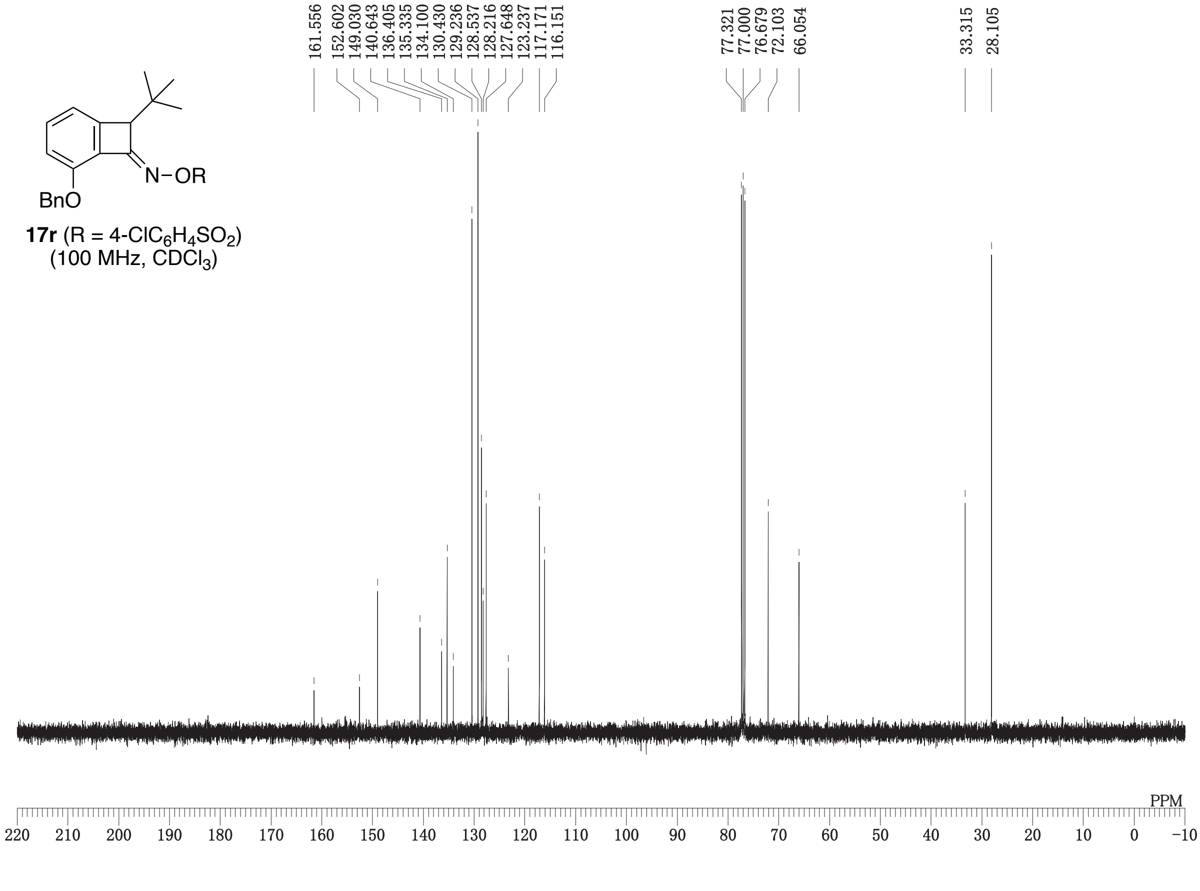
BnO
N−OR
17r (R = 4-ClC$_6$H$_4$SO$_2$)
(100 MHz, CDCl$_3$)
161.556 152.602 149.030 140.643 136.405 135.335 134.100 130.430 129.236 128.537 128.216 127.648 123.237 117.171 116.151
77.321 77.000 76.679 72.103 66.054
33.315 28.105
PPM
220 210 200 190 180 170 160 150 140 130 120 110 100 90 80 70 60 50 40 30 20 10 0 −10

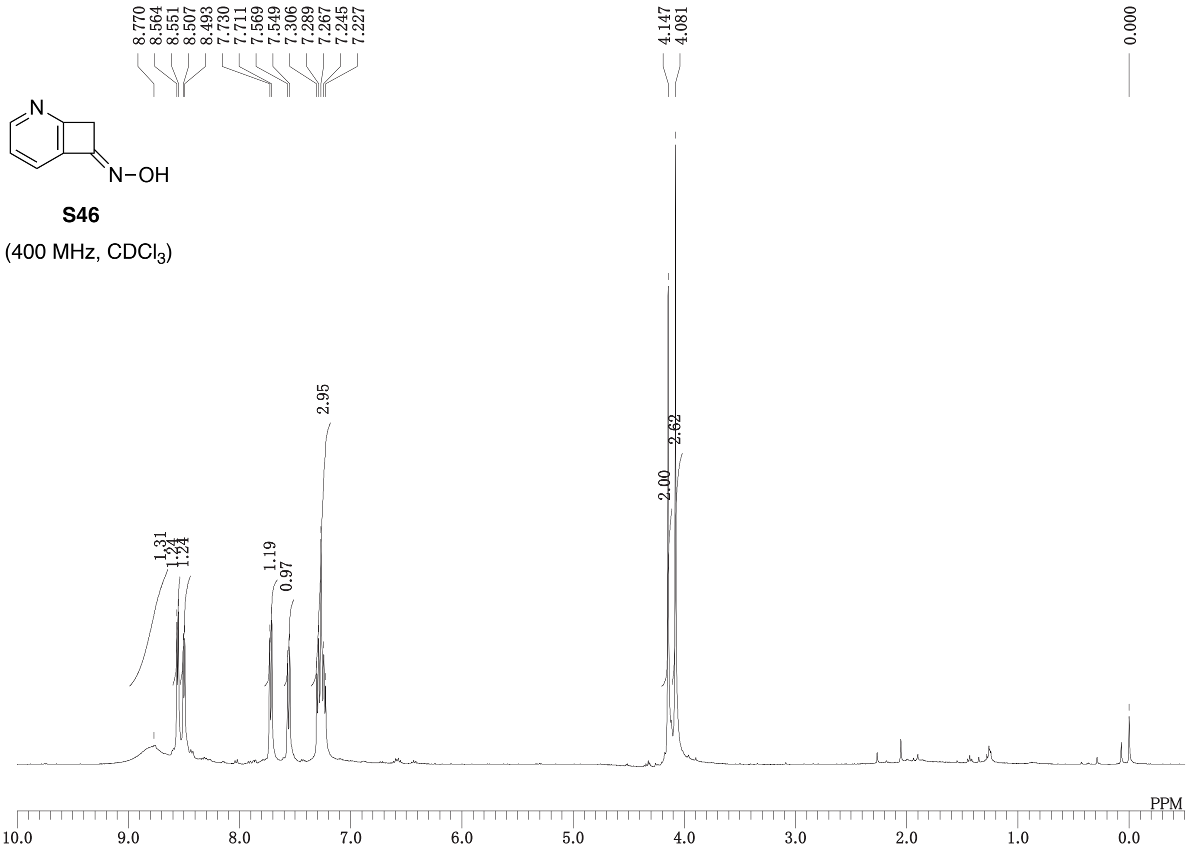
8.770
8.564
8.551
8.507
8.493
7.730
7.711
7.569
7.549
7.306
7.289
7.267
7.245
7.227
4.147
4.081
0.000
N
N−OH
S46
(400 MHz, $CDCl_3$)
1.31
1.24
1.24
1.19
0.97
2.95
2.00
2.62
PPM
10.0
9.0
8.0
7.0
6.0
5.0
4.0
3.0
2.0
1.0
0.0

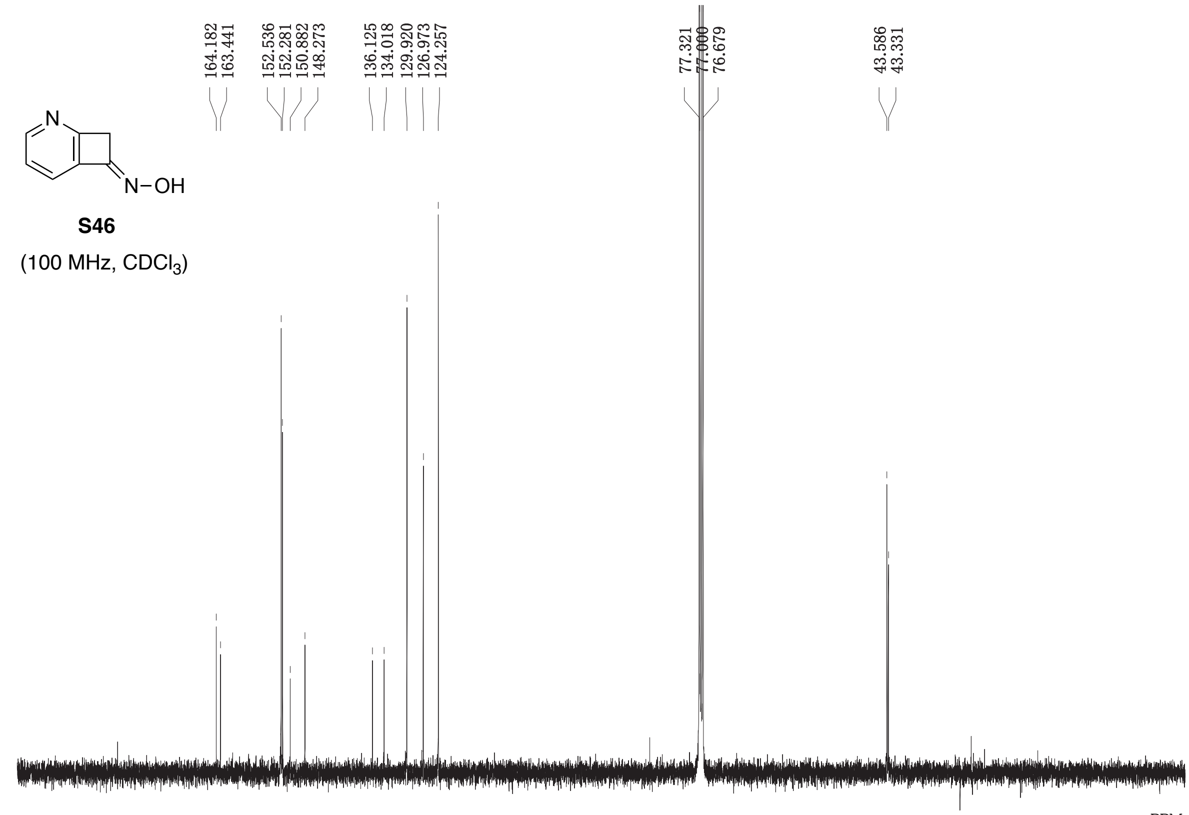
164.182
163.441
152.536
152.281
150.882
148.273
136.125
134.018
129.920
126.973
124.257
77.321
77.000
76.679
43.586
43.331
N
N−OH
S46
(100 MHz, $CDCl_3$)
PPM
200
190
180
170
160
150
140
130
120
110
100
90
80
70
60
50
40
30
20
10
0
−10

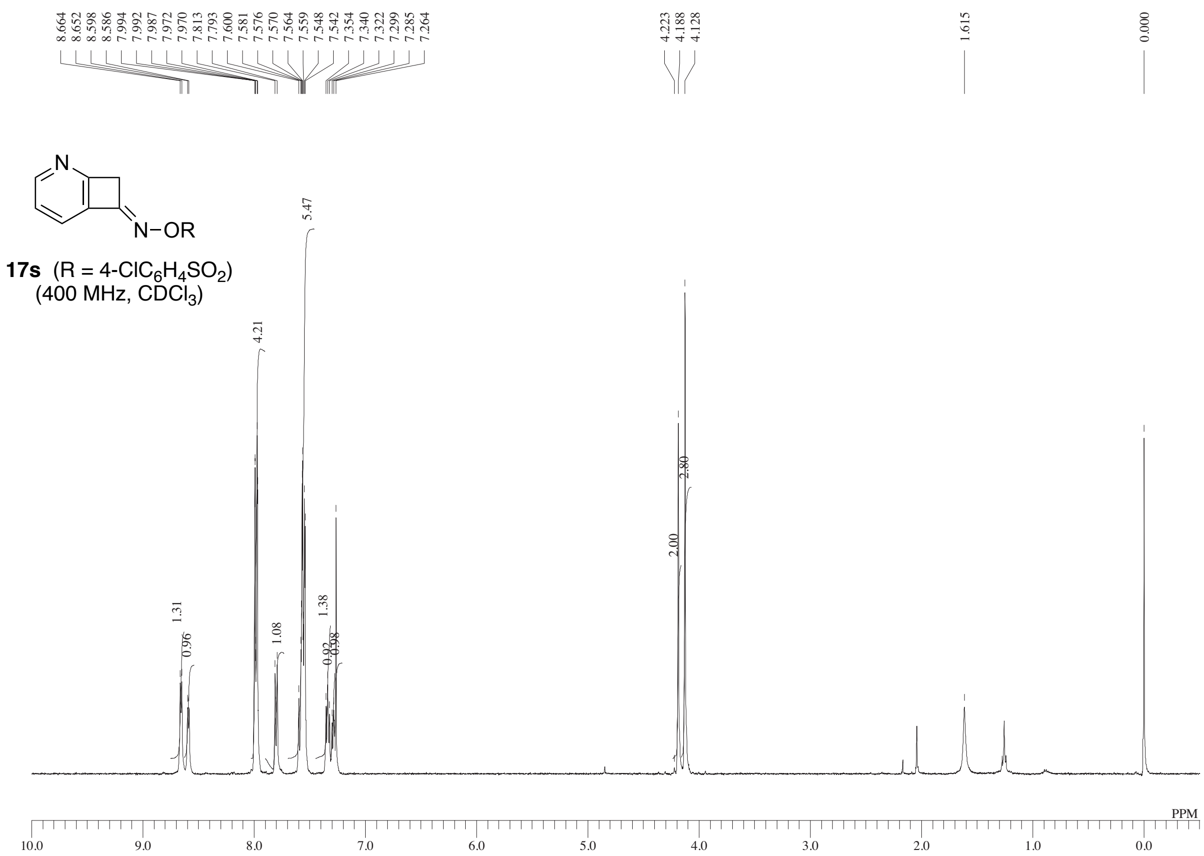
8.664
8.652
8.598
8.586
7.994
7.992
7.987
7.972
7.970
7.813
7.793
7.600
7.581
7.576
7.570
7.564
7.559
7.548
7.542
7.354
7.340
7.322
7.299
7.285
7.264
4.223
4.188
4.128
1.615
0.000
N
N−OR
17s (R = 4-ClC6H4SO2)
(400 MHz, CDCl3)
5.47
4.21
2.80
2.00
1.31
0.96
1.08
1.38
0.92
0.98
PPM
10.0 9.0 8.0 7.0 6.0 5.0 4.0 3.0 2.0 1.0 0.0

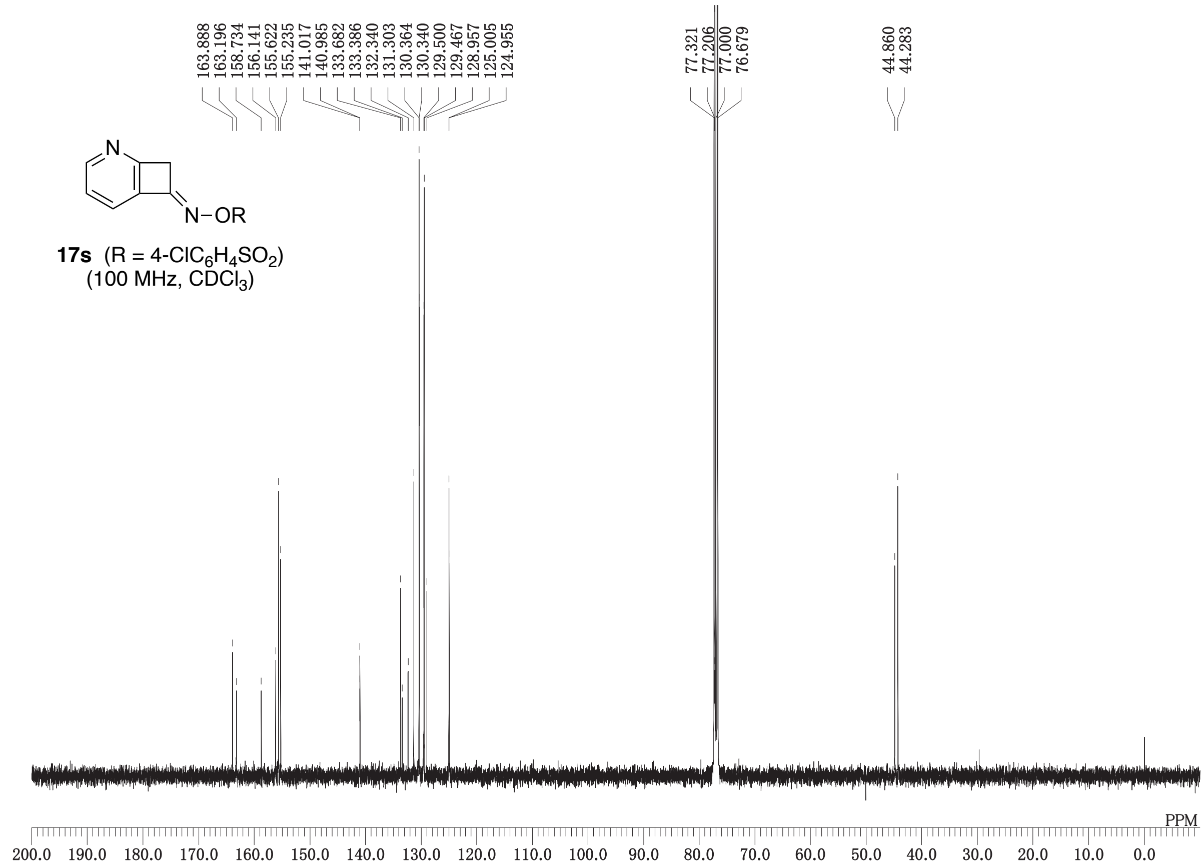
163.888
163.196
158.734
156.141
155.622
155.235
141.017
140.985
133.682
133.386
132.340
131.303
130.364
130.340
129.500
129.467
128.957
125.005
124.955
77.321
77.206
77.000
76.679
44.860
44.283
N
N−OR
17s (R = 4-ClC6H4SO2)
(100 MHz, CDCl3)
PPM
200.0 190.0 180.0 170.0 160.0 150.0 140.0 130.0 120.0 110.0 100.0 90.0 80.0 70.0 60.0 50.0 40.0 30.0 20.0 10.0 0.0

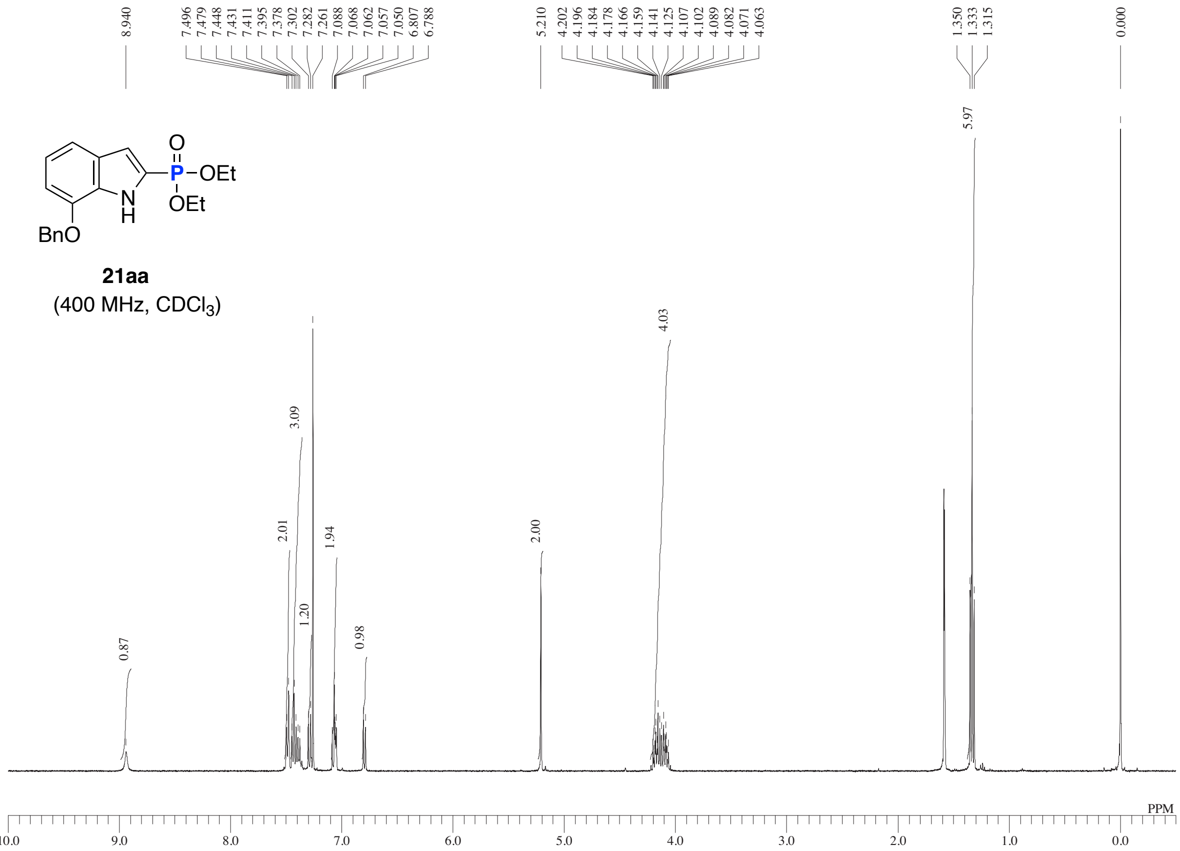

21aa
(400 MHz, $CDCl_3$)

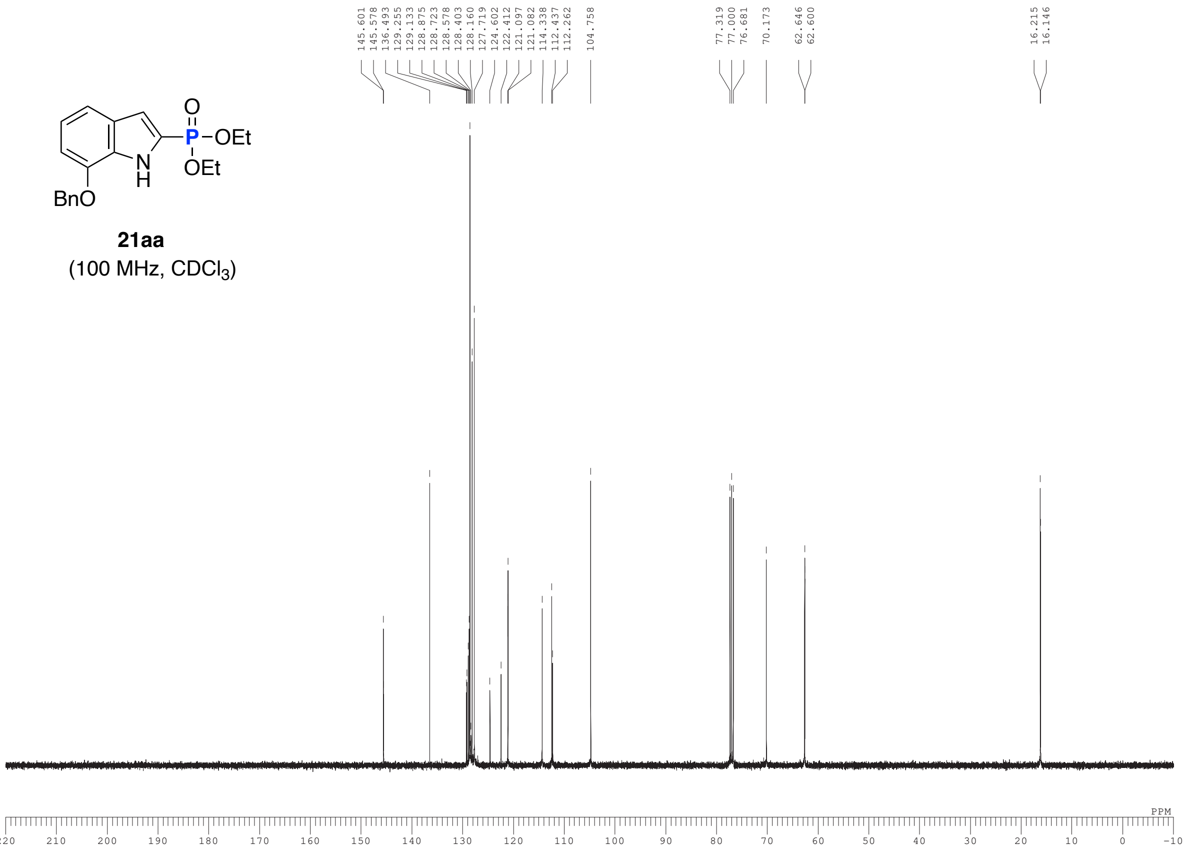

21aa
(100 MHz, $CDCl_3$)

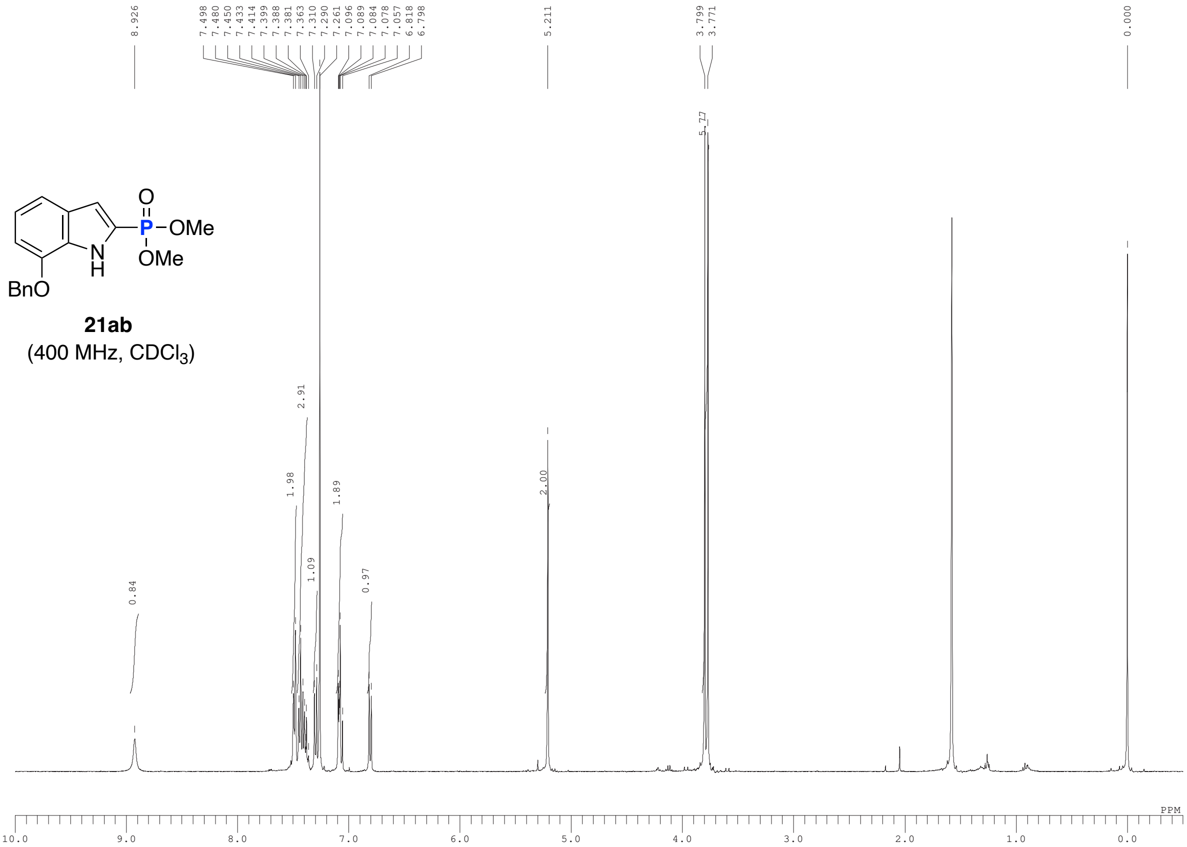

21ab
(400 MHz, $CDCl_3$)

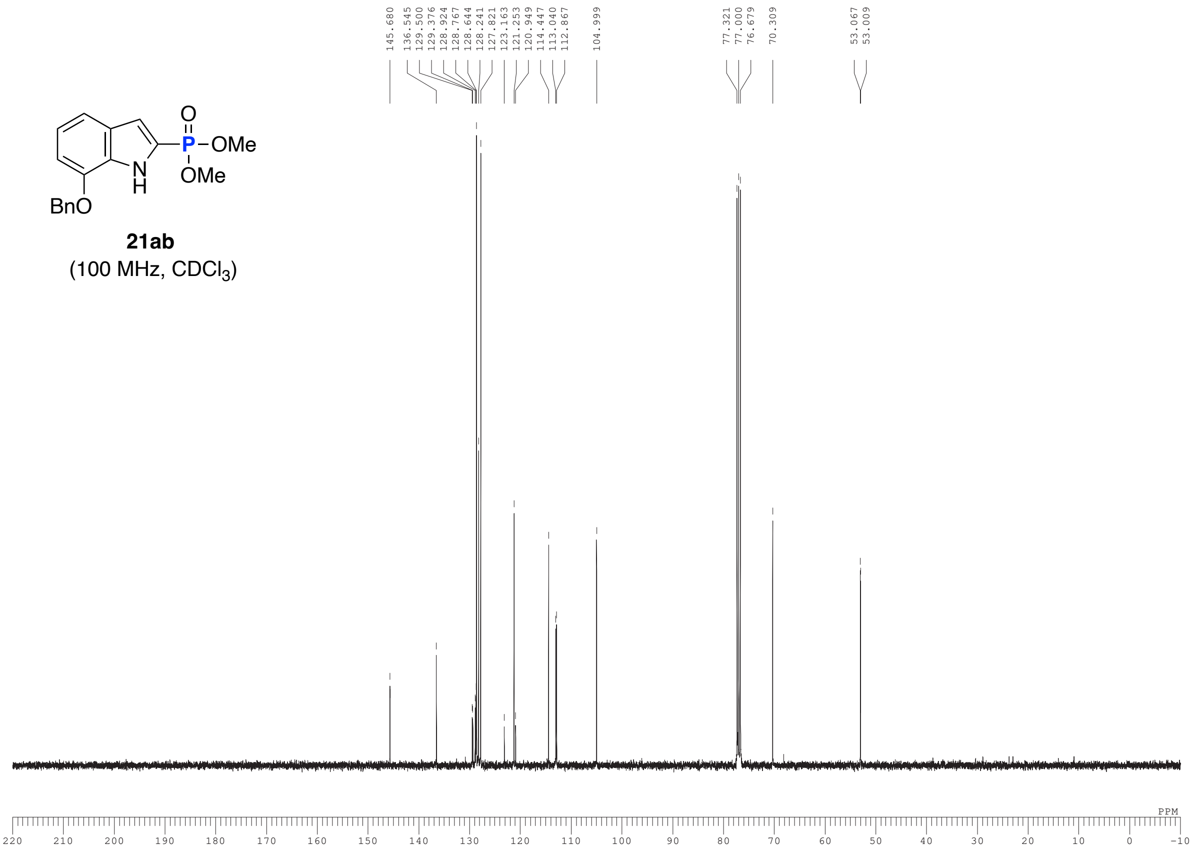

21ab
(100 MHz, $CDCl_3$)

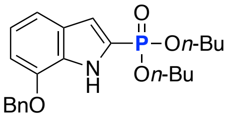

21ac
(400 MHz, $CDCl_3$)

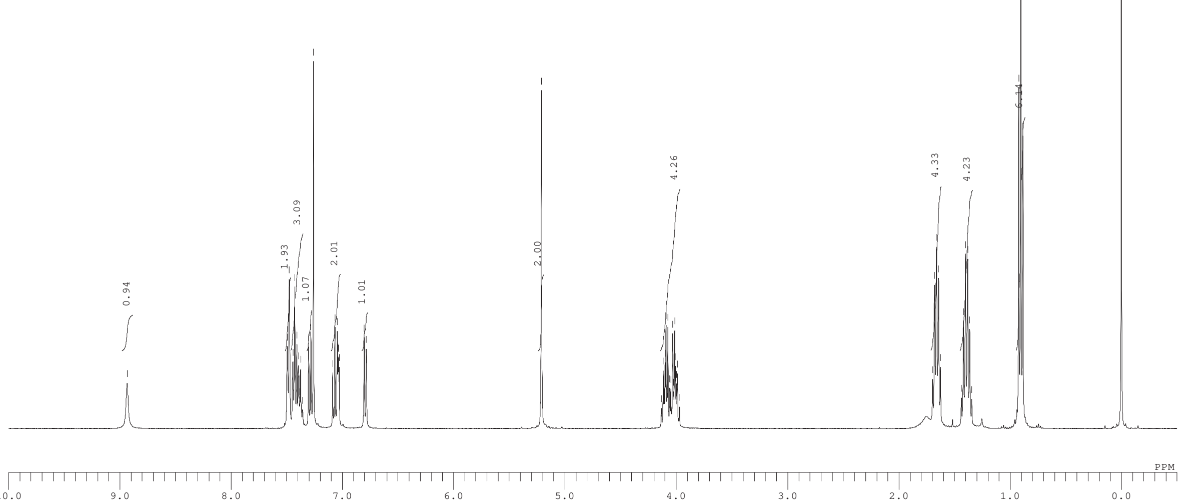

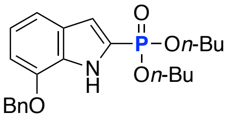

21ac
(100 MHz, $CDCl_3$)

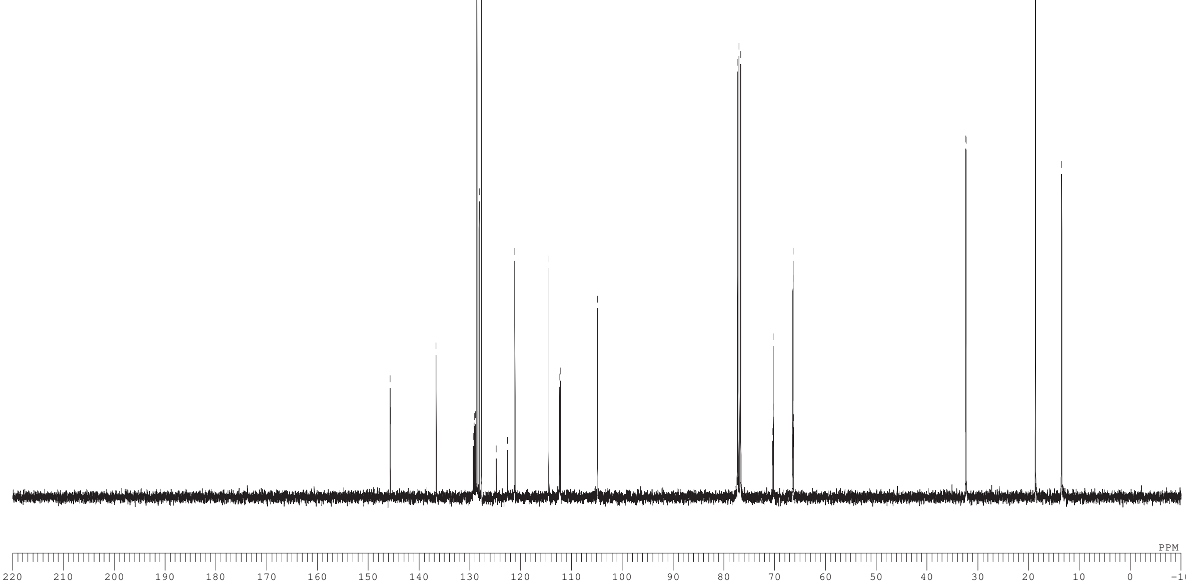

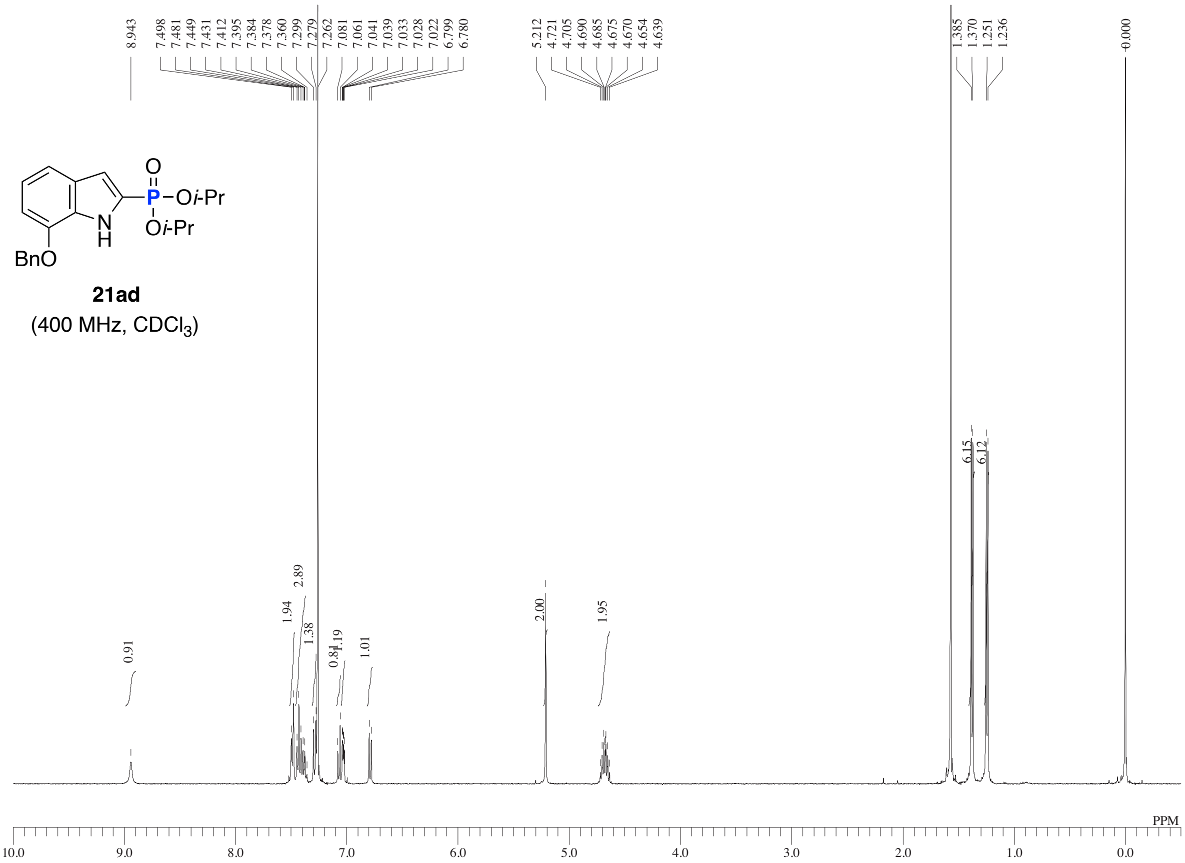

21ad
(400 MHz, $CDCl_3$)

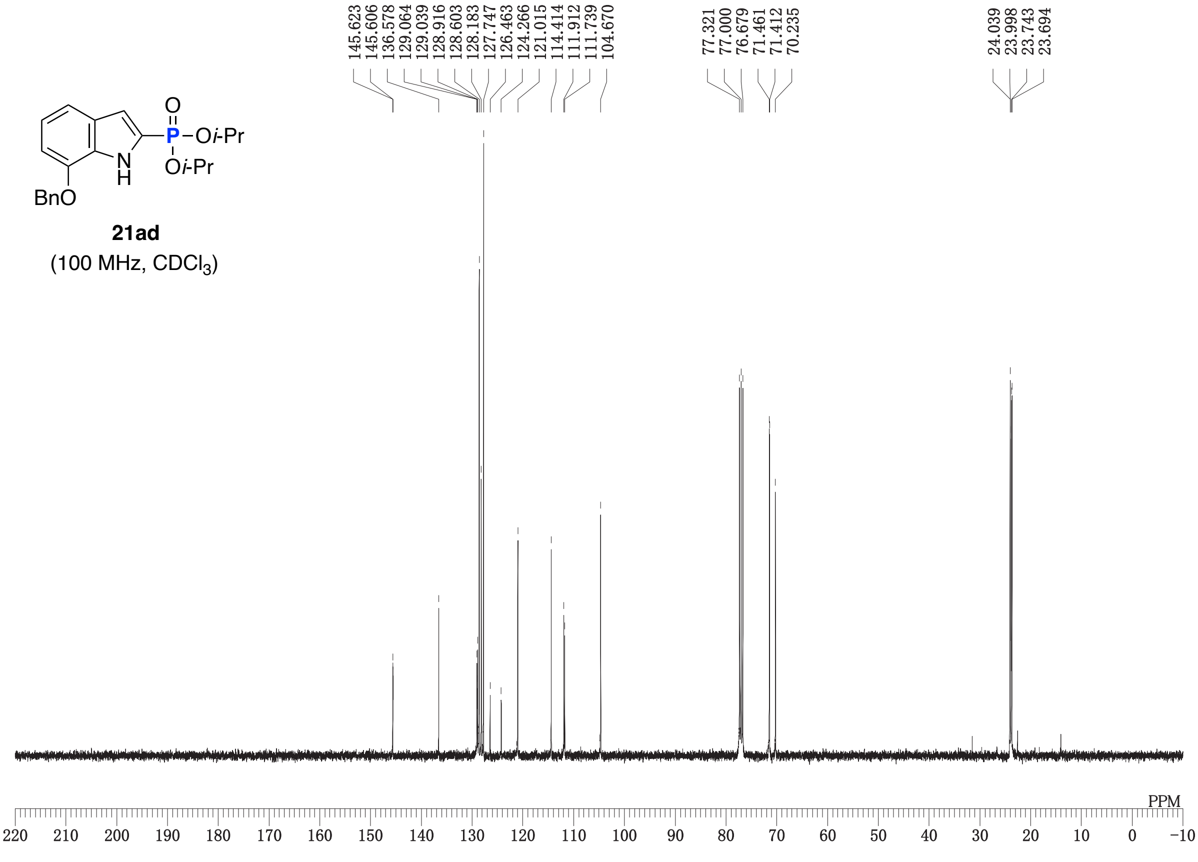

21ad
(100 MHz, $CDCl_3$)

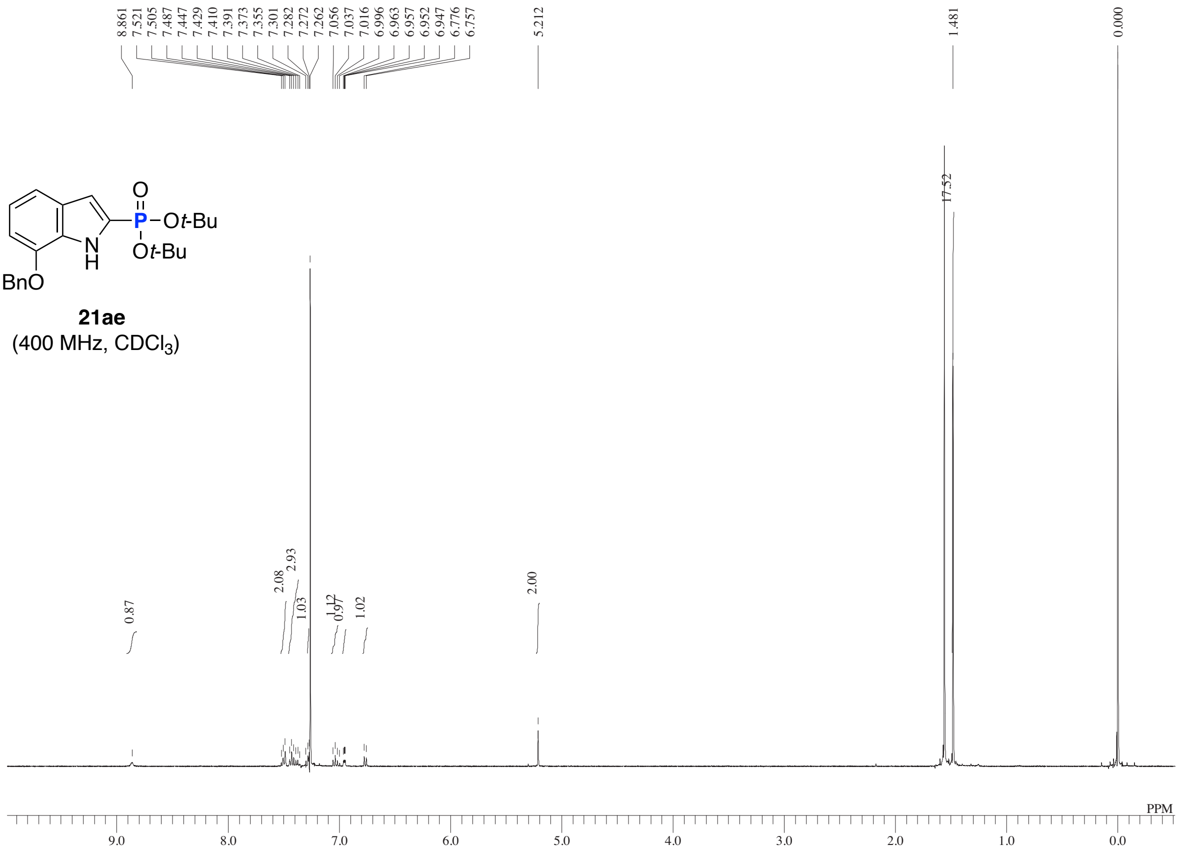
8.861
7.521
7.505
7.487
7.447
7.429
7.410
7.391
7.373
7.355
7.301
7.282
7.272
7.262
7.056
7.037
7.016
6.996
6.963
6.957
6.952
6.947
6.776
6.757
5.212
1.481
0.000
O
P
Ot-Bu
Ot-Bu
N
H
BnO
21ae
(400 MHz, CDCl3)
17.52
0.87
2.08
2.93
1.03
1.12
0.97
1.02
2.00
PPM
9.0
8.0
7.0
6.0
5.0
4.0
3.0
2.0
1.0
0.0

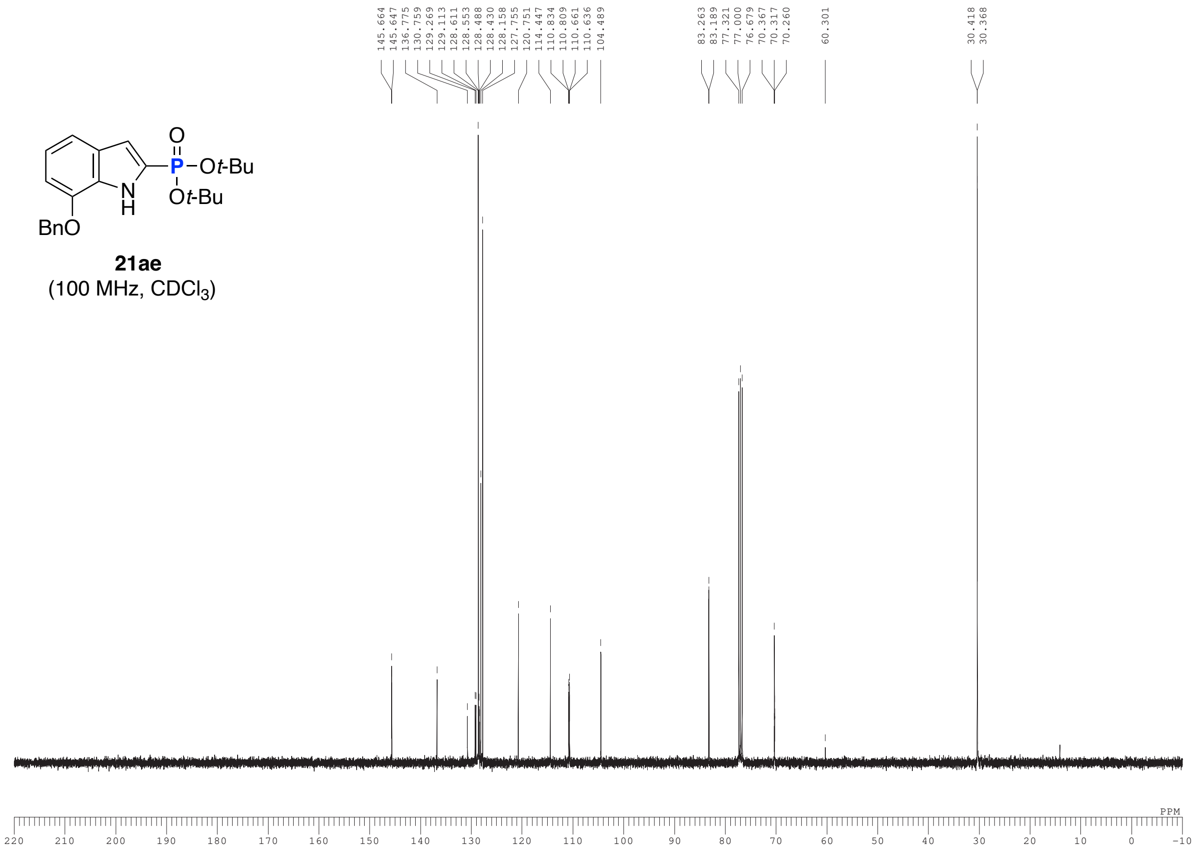
145.664
145.647
136.775
130.759
129.269
129.113
128.611
128.553
128.488
128.430
128.158
127.755
120.751
114.447
110.834
110.809
110.661
110.636
104.489
83.263
83.189
77.321
77.000
76.679
70.367
70.317
70.260
60.301
30.418
30.368
O
P
Ot-Bu
Ot-Bu
N
H
BnO
21ae
(100 MHz, CDCl3)
PPM
220
210
200
190
180
170
160
150
140
130
120
110
100
90
80
70
60
50
40
30
20
10
0
-10

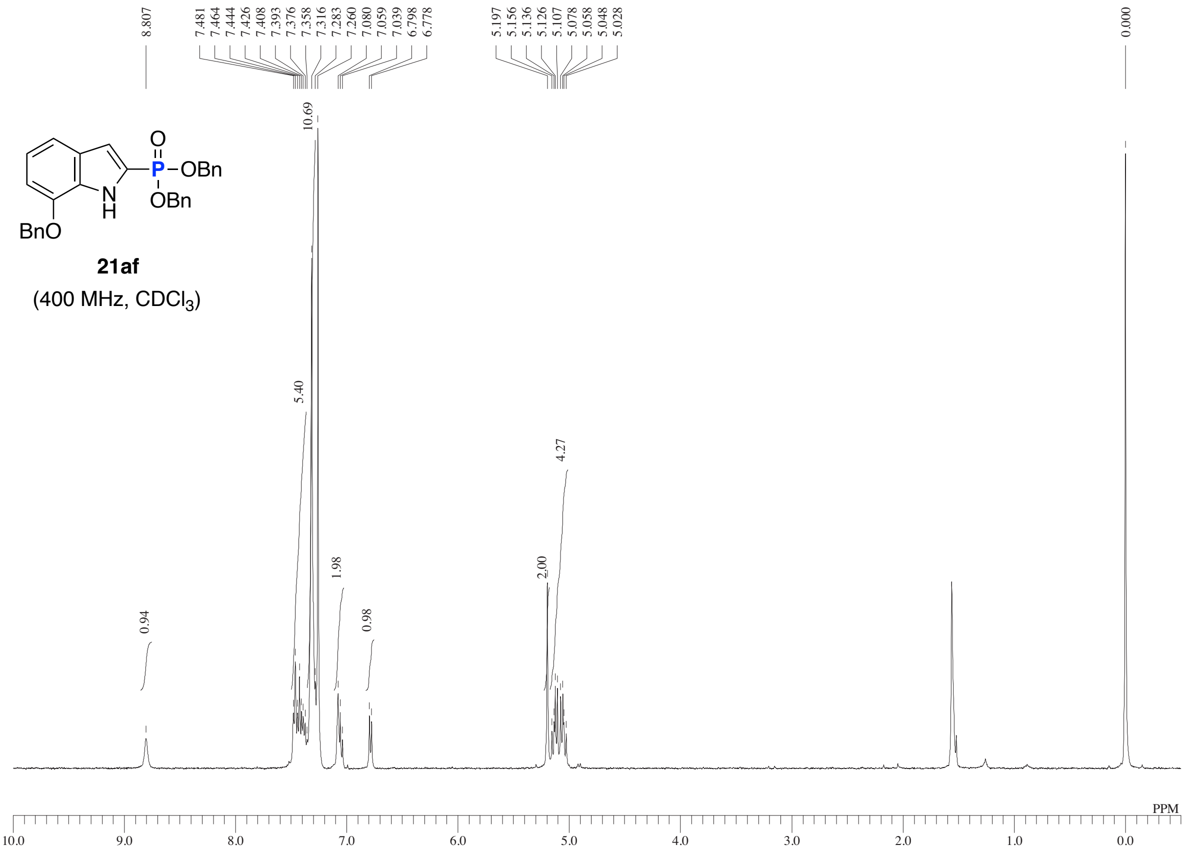

21af

(400 MHz, $CDCl_3$)

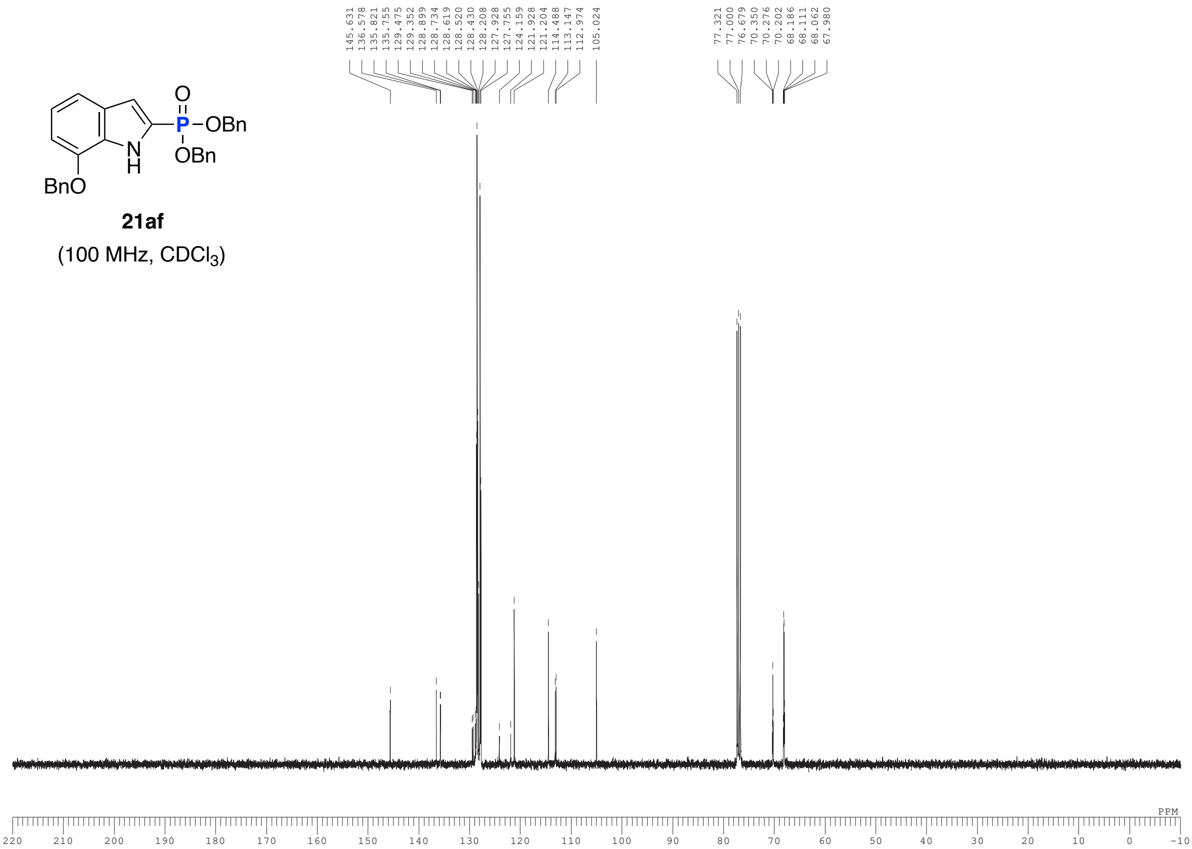

21af

(100 MHz, $CDCl_3$)

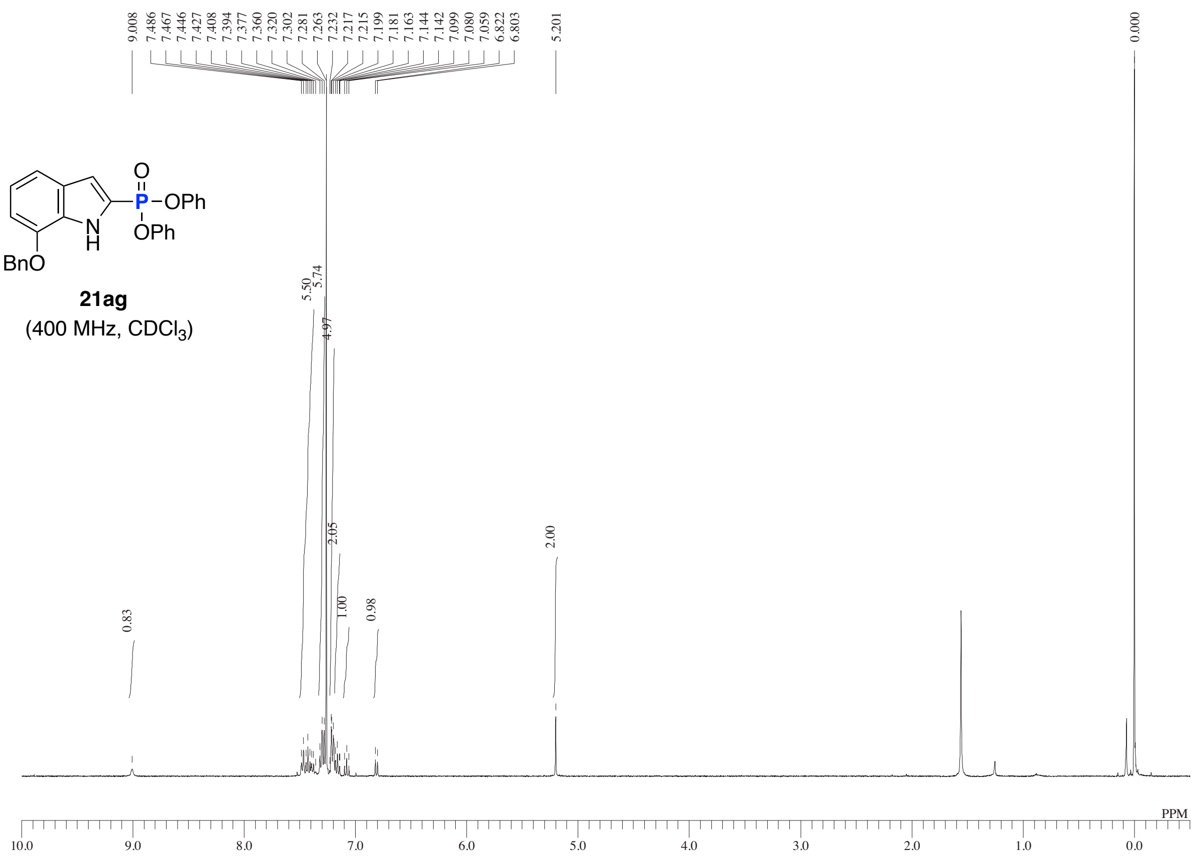
9.008 7.486 7.467 7.446 7.427 7.408 7.394 7.377 7.360 7.320 7.302 7.281 7.263 7.232 7.217 7.215 7.199 7.181 7.163 7.144 7.142 7.099 7.080 7.059 6.822 6.803
5.201
0.000
P
OPh
OPh
O
N
H
BnO
21ag
(400 MHz, CDCl3)
0.83
5.50
5.74
4.97
2.05
1.00
0.98
2.00
PPM
10.0
9.0
8.0
7.0
6.0
5.0
4.0
3.0
2.0
1.0
0.0

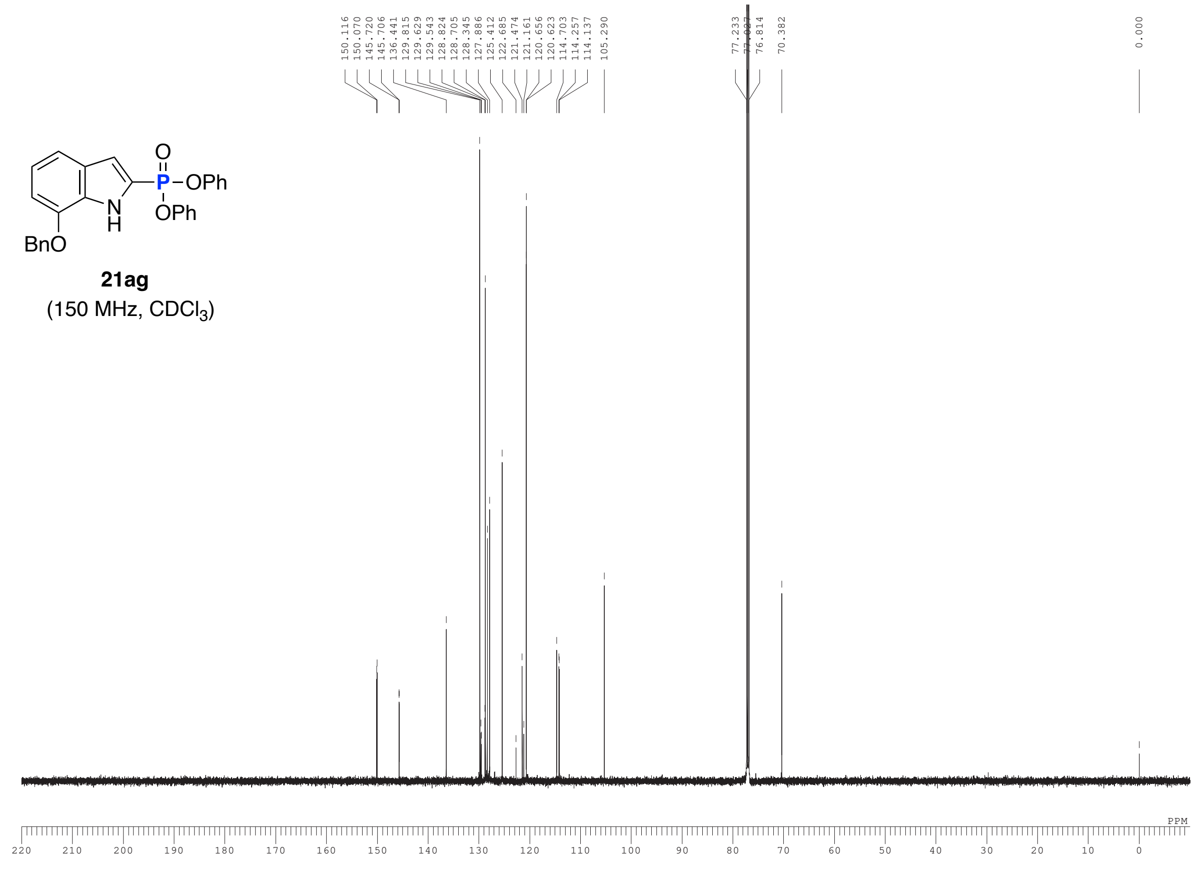
150.116 150.070 145.720 145.706 136.441 129.815 129.629 129.543 128.824 128.705 128.345 127.886 125.412 122.685 121.474 121.161 120.656 120.623 114.703 114.257 114.137 105.290
77.233
[illegible]
76.814
70.382
0.000
P
OPh
OPh
O
N
H
BnO
21ag
(150 MHz, CDCl3)
PPM
220
210
200
190
180
170
160
150
140
130
120
110
100
90
80
70
60
50
40
30
20
10
0

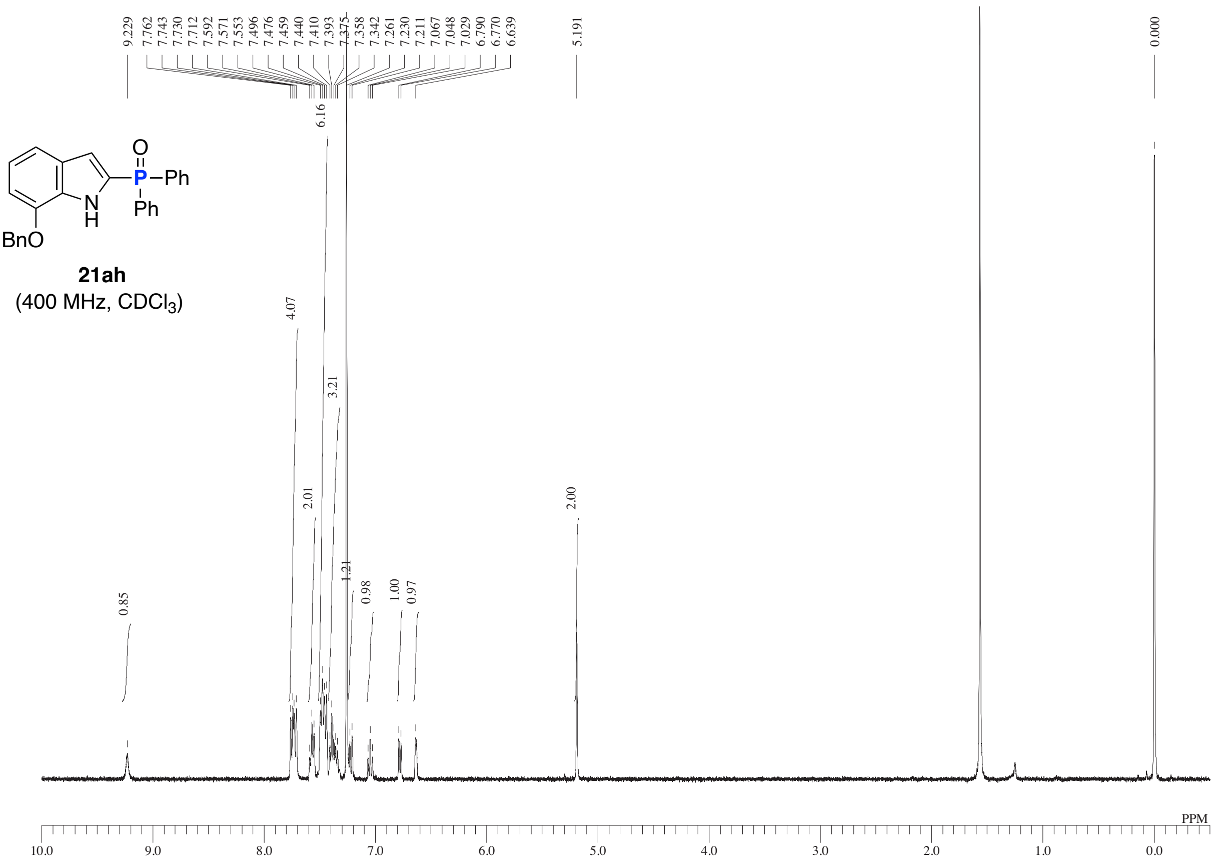
9.229
7.762
7.743
7.730
7.712
7.592
7.571
7.553
7.496
7.476
7.459
7.440
7.410
7.393
7.375
7.358
7.342
7.261
7.230
7.211
7.067
7.048
7.029
6.790
6.770
6.639
5.191
0.000
O
P
Ph
N
H
BnO
21ah
(400 MHz, CDCl3)
0.85
4.07
2.01
6.16
3.21
1.21
0.98
1.00
0.97
2.00
PPM
10.0
9.0
8.0
7.0
6.0
5.0
4.0
3.0
2.0
1.0
0.0

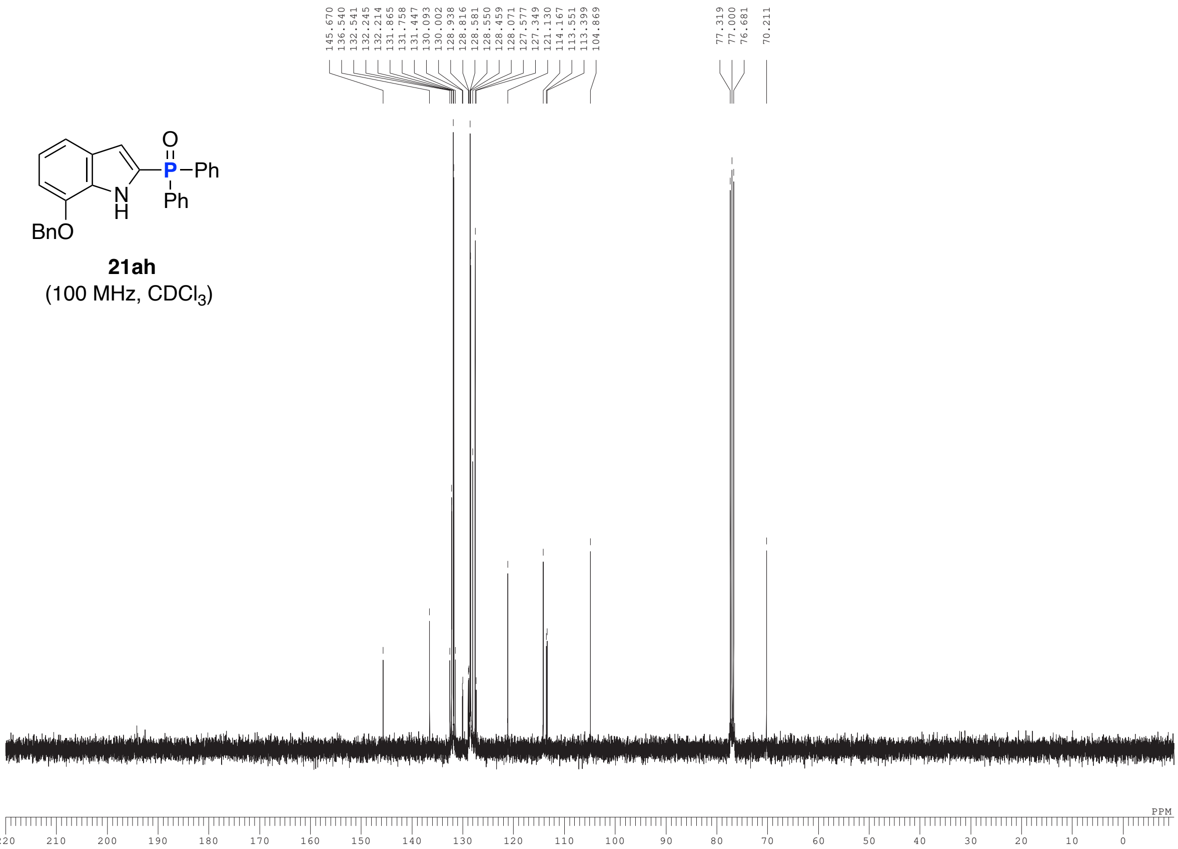
145.670
136.540
132.541
132.245
132.214
131.865
131.758
131.447
130.093
130.002
128.938
128.816
128.581
128.550
128.459
128.071
127.577
127.349
121.130
114.167
113.551
113.399
104.869
77.319
77.000
76.681
70.211
O
P
Ph
N
H
BnO
21ah
(100 MHz, CDCl3)
PPM
220
210
200
190
180
170
160
150
140
130
120
110
100
90
80
70
60
50
40
30
20
10
0

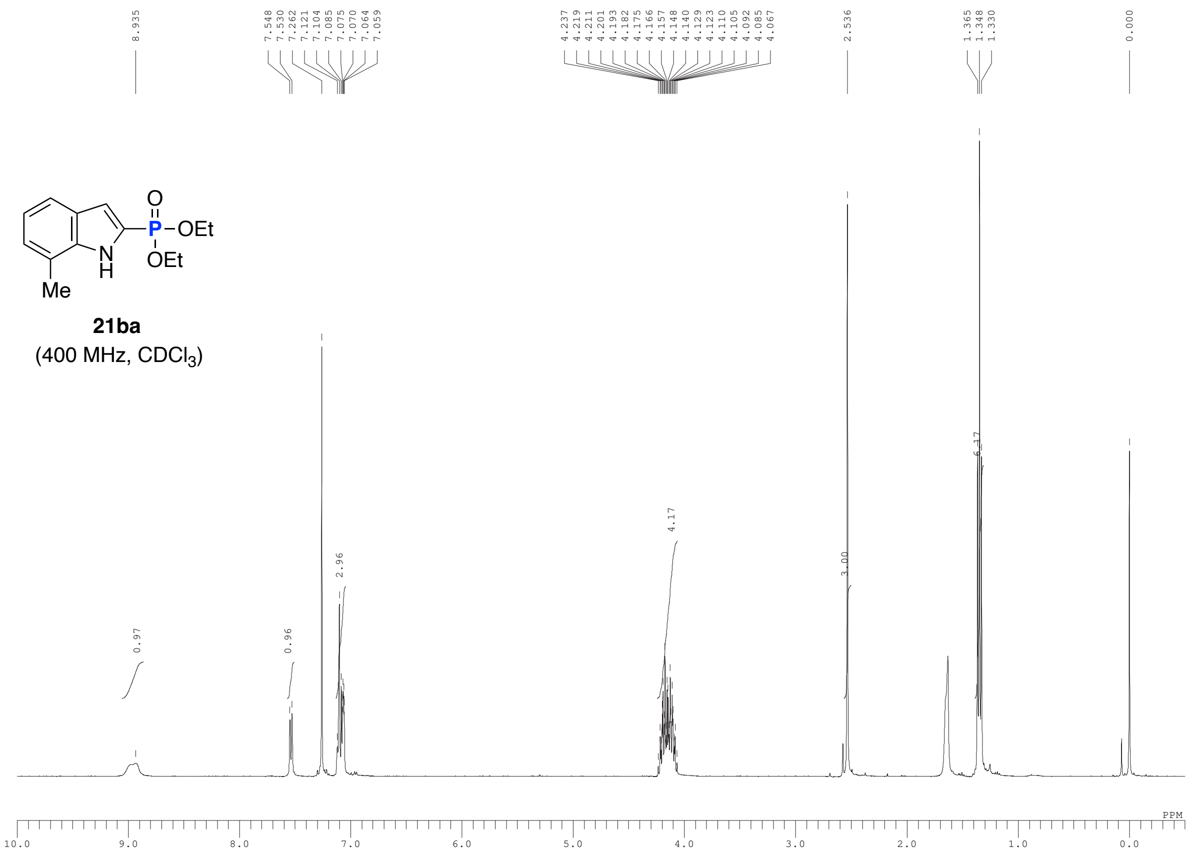

OEt
OEt
O
P
N
H
Me
21ba
(400 MHz, CDCl3)
PPM

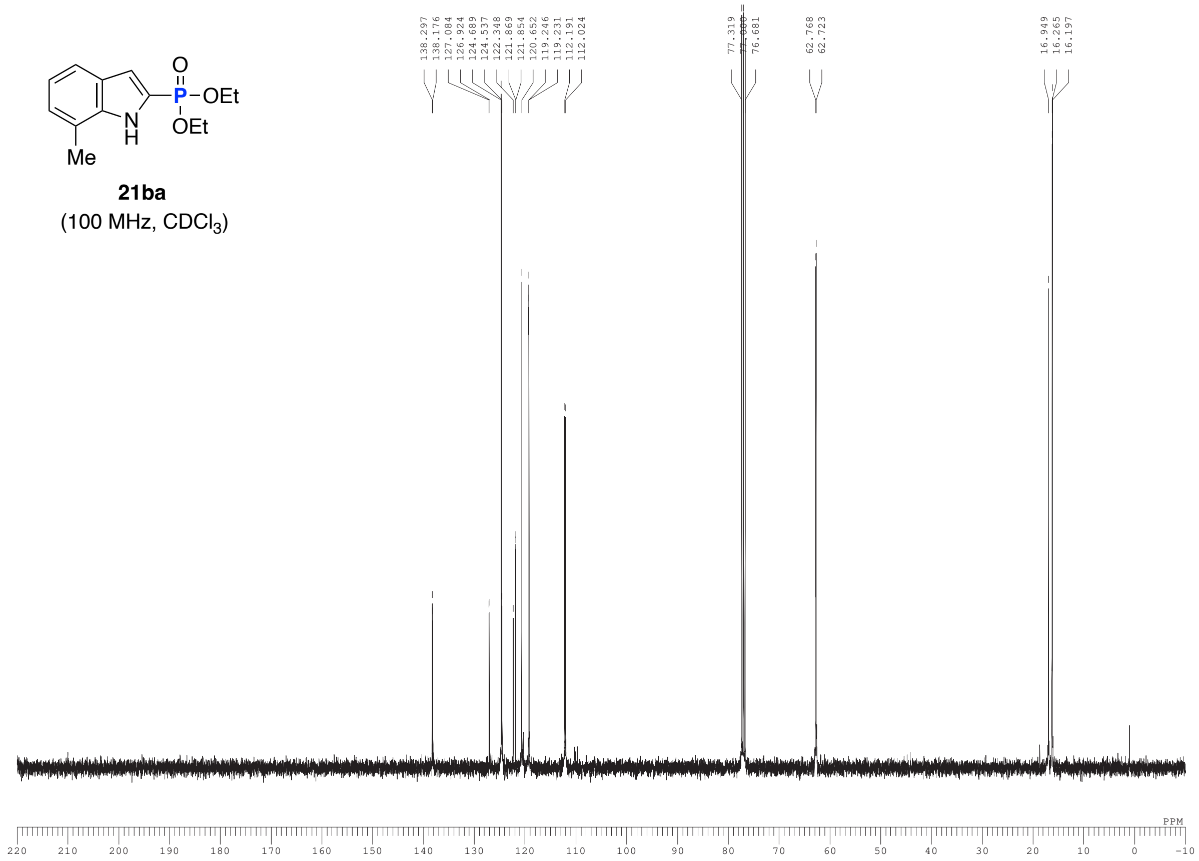

OEt
OEt
O
P
N
H
Me
21ba
(100 MHz, CDCl3)
PPM

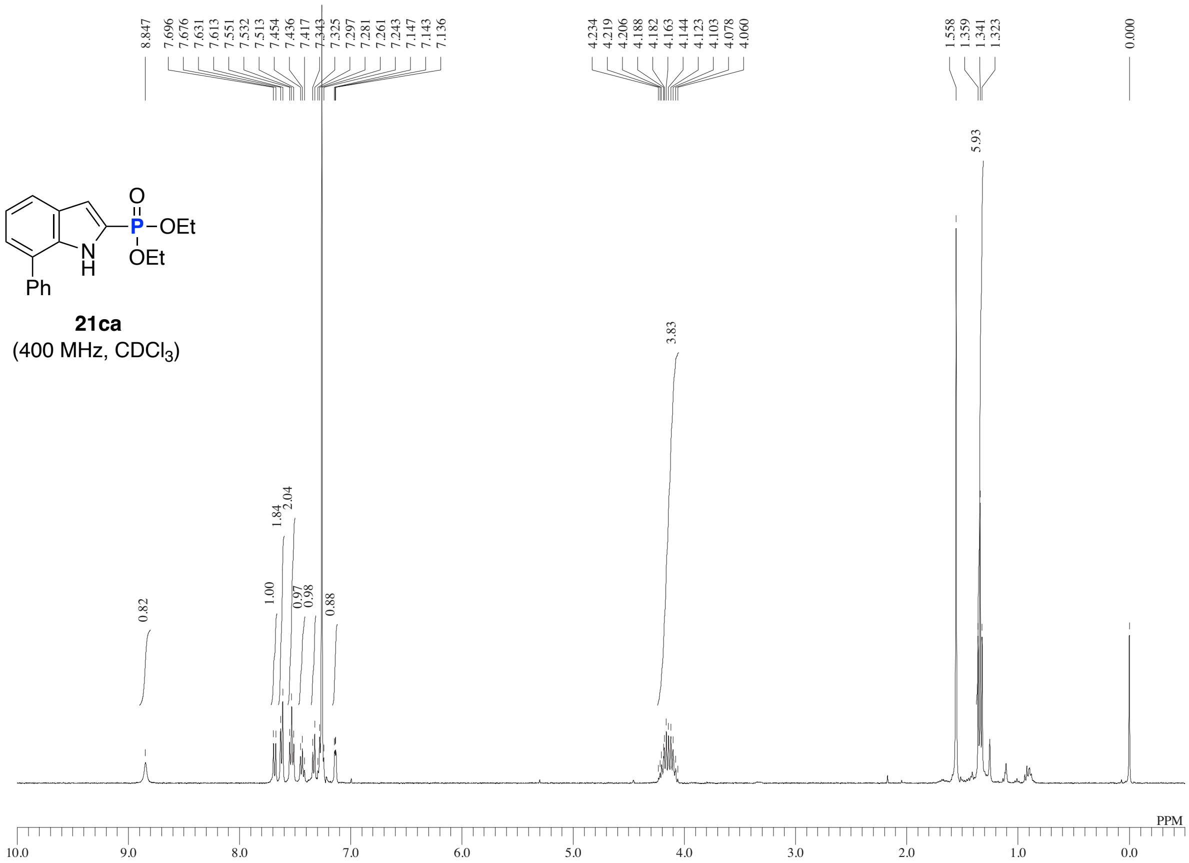

21ca
(400 MHz, $CDCl_3$)

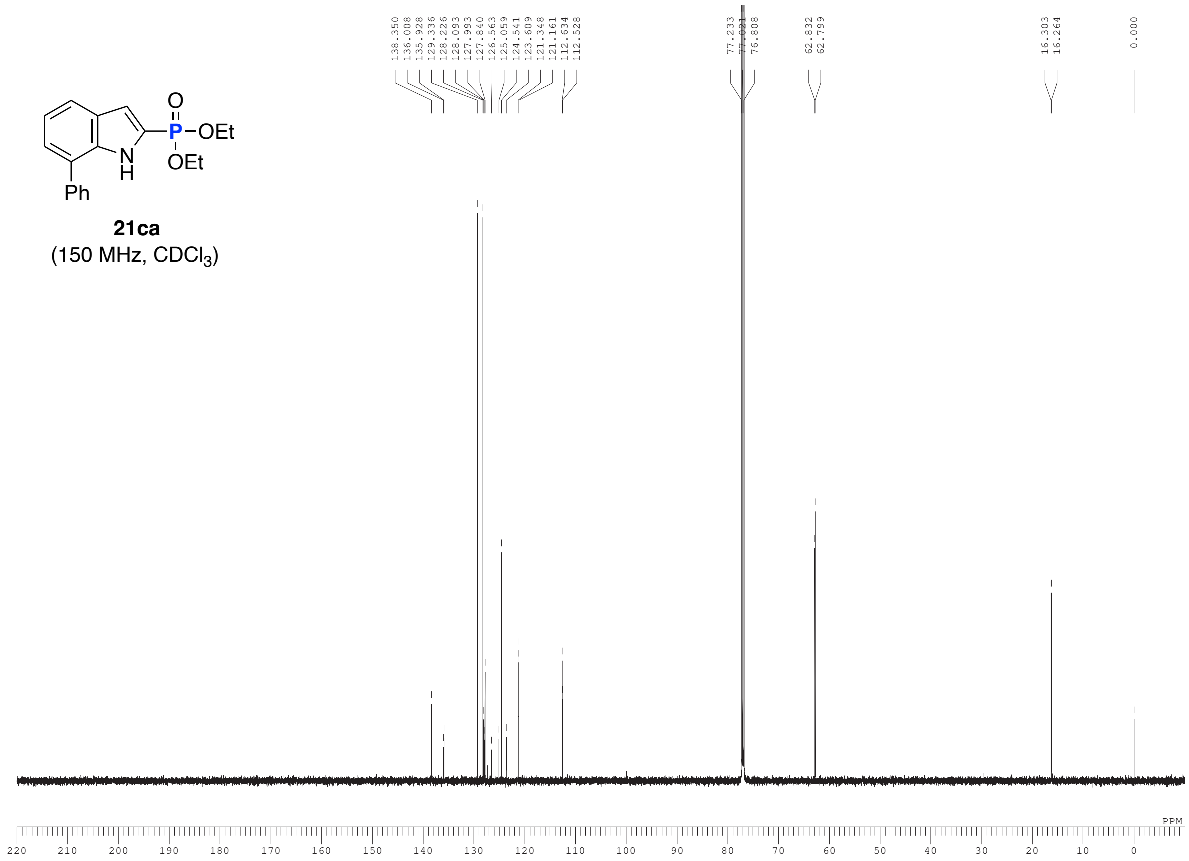

21ca
(150 MHz, $CDCl_3$)

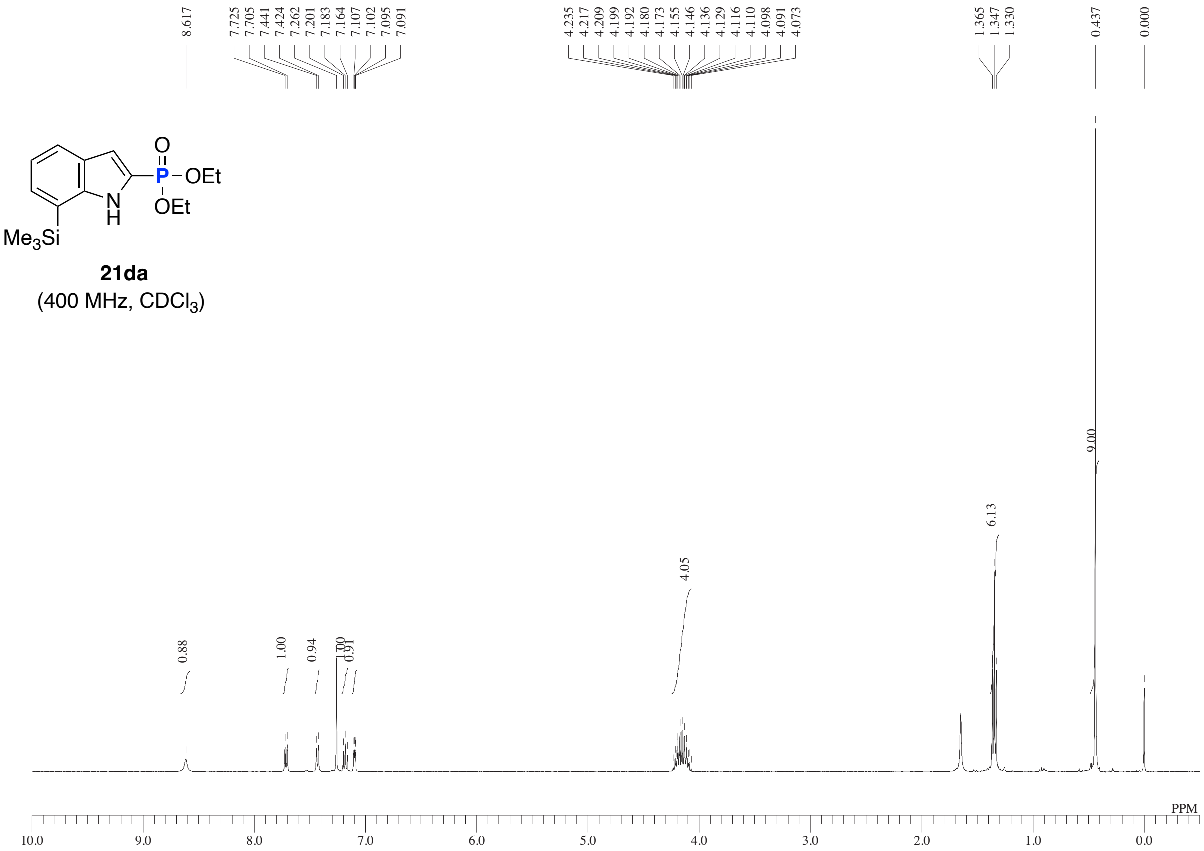
21da
(400 MHz, CDCl$_3$)
8.617
7.725 7.705 7.441 7.424 7.262 7.201 7.183 7.164 7.107 7.102 7.095 7.091
4.235 4.217 4.209 4.199 4.192 4.180 4.173 4.155 4.146 4.136 4.129 4.116 4.110 4.098 4.091 4.073
1.365 1.347 1.330
0.437
0.000
0.88 1.00 0.94 1.00 0.91 4.05 6.13 9.00
PPM
10.0 9.0 8.0 7.0 6.0 5.0 4.0 3.0 2.0 1.0 0.0

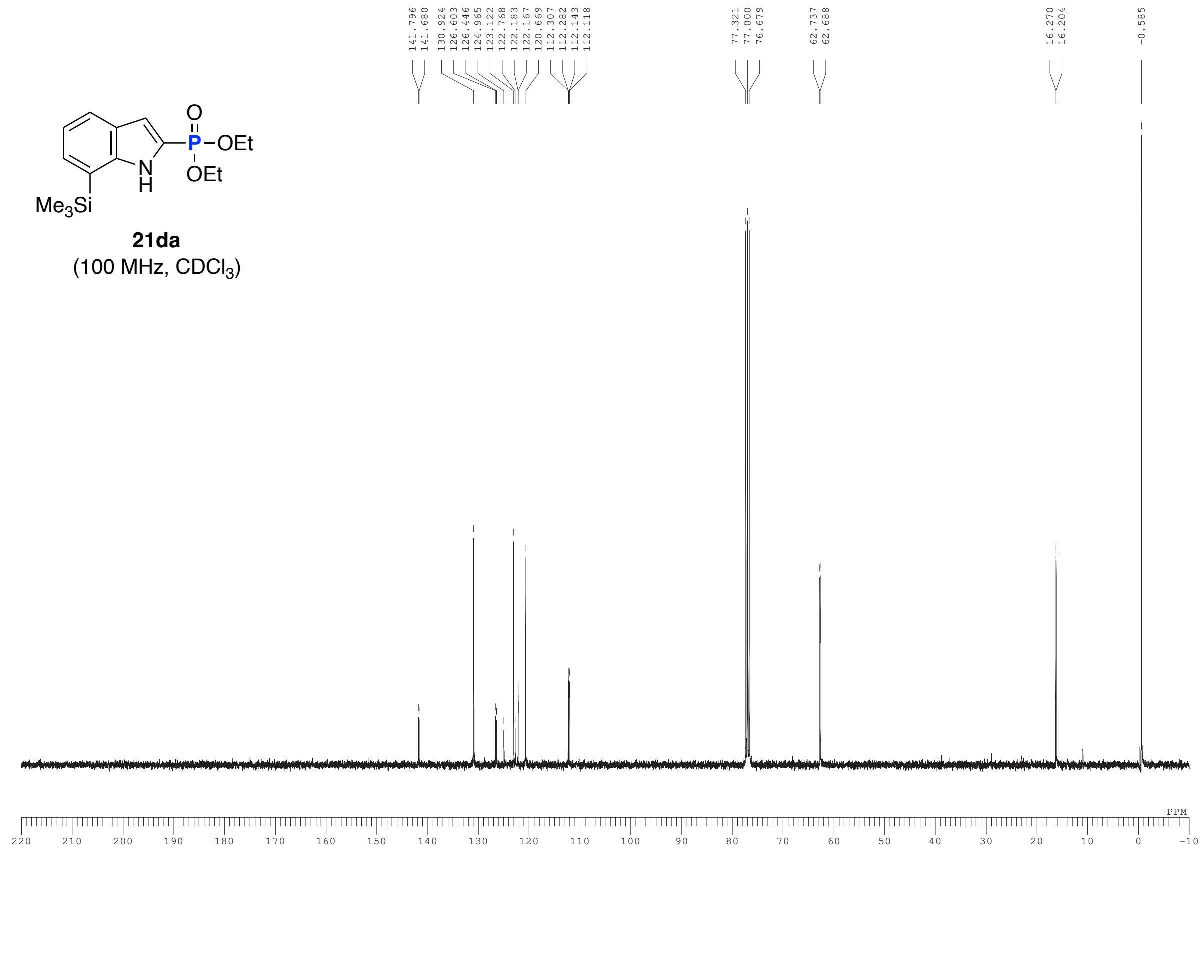
21da
(100 MHz, CDCl$_3$)
141.796 141.680 130.924 126.603 126.446 124.965 123.122 122.768 122.183 122.167 120.669 112.307 112.282 112.143 112.118
77.321 77.000 76.679
62.737 62.688
16.270 16.204
-0.585
PPM
220 210 200 190 180 170 160 150 140 130 120 110 100 90 80 70 60 50 40 30 20 10 0 -10

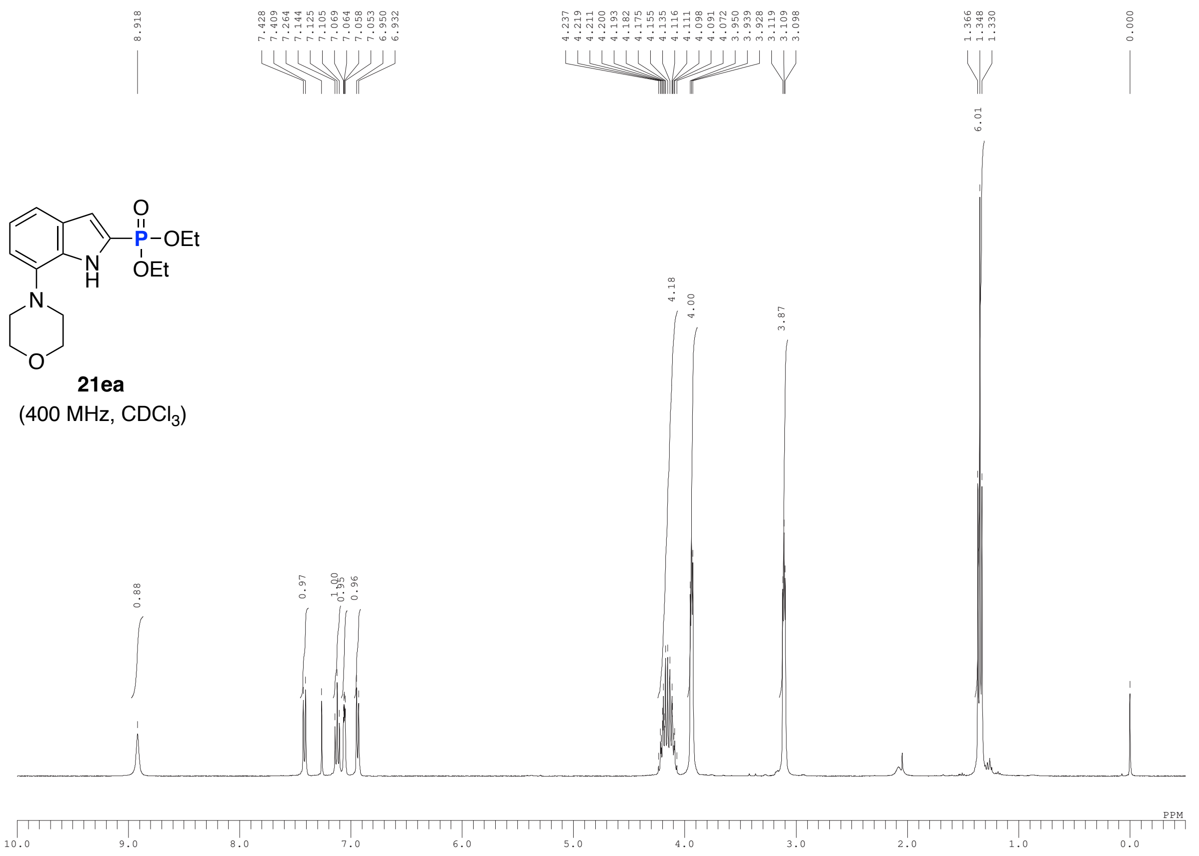
21ea
(400 MHz, CDCl3)
8.918
7.428
7.409
7.264
7.144
7.125
7.105
7.069
7.064
7.058
7.053
6.950
6.932
4.237
4.219
4.211
4.200
4.193
4.182
4.175
4.155
4.135
4.116
4.111
4.098
4.091
4.072
3.950
3.939
3.928
3.119
3.109
3.098
1.366
1.348
1.330
0.000
0.88
0.97
1.00
0.95
0.96
4.18
4.00
3.87
6.01
PPM

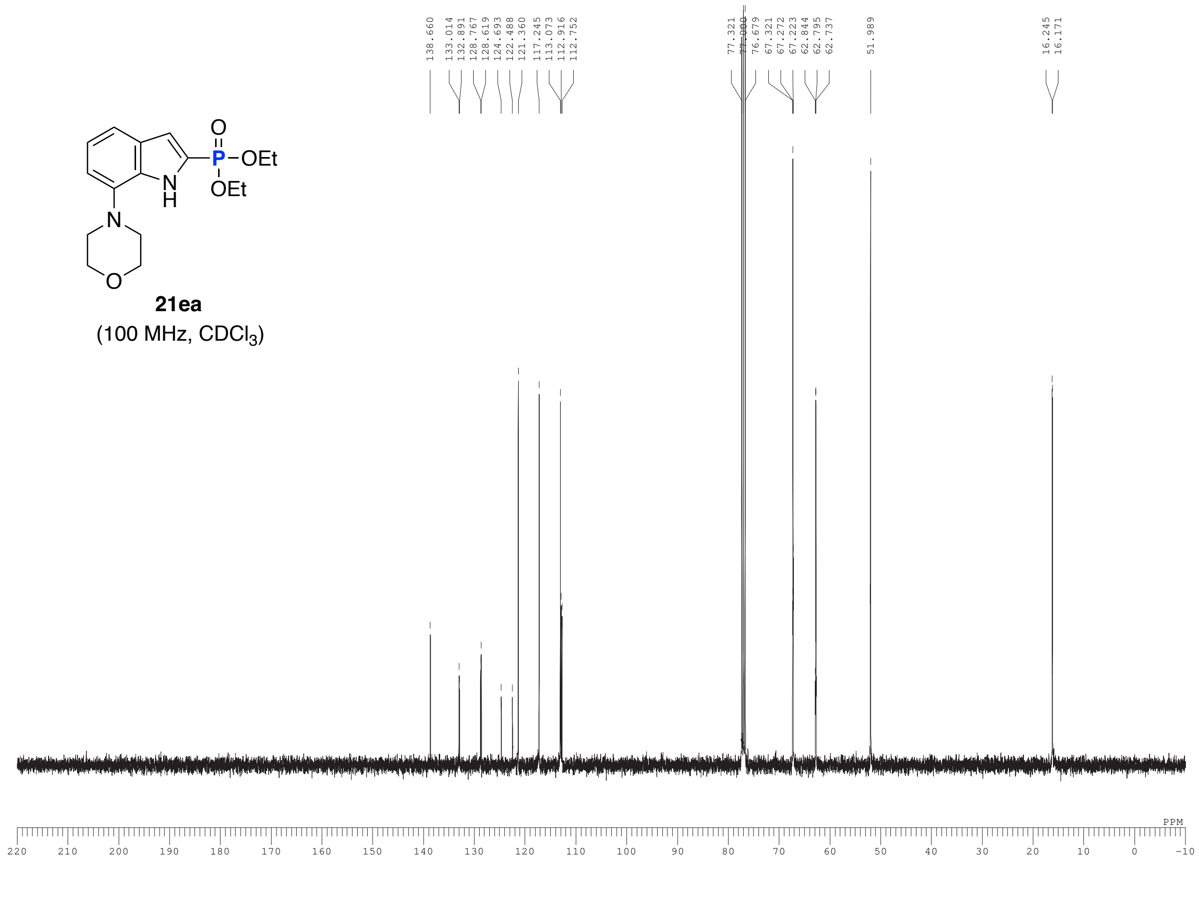
21ea
(100 MHz, CDCl3)
138.660
133.014
132.891
128.767
128.619
124.693
122.488
121.360
117.245
113.073
112.916
112.752
77.321
77.000
76.679
67.321
67.272
67.223
62.844
62.795
62.737
51.989
16.245
16.171
PPM

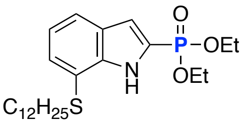

21fa
(400 MHz, $CDCl_3$)

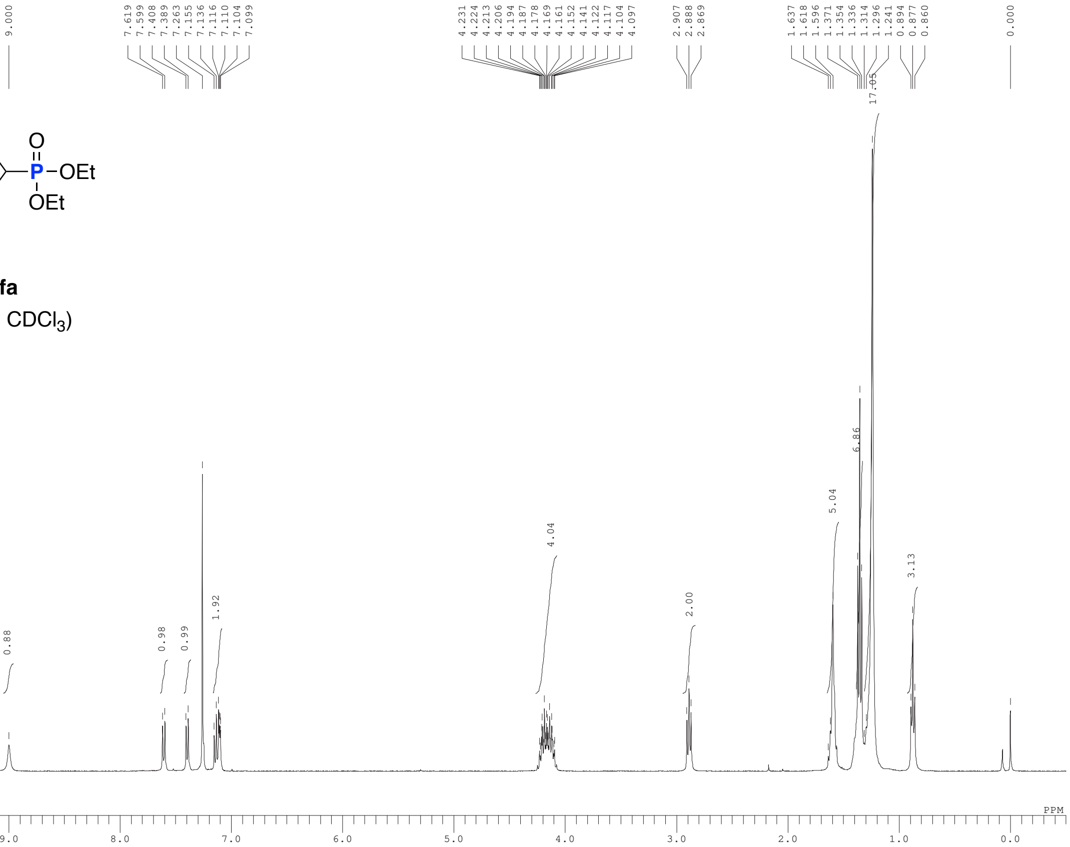

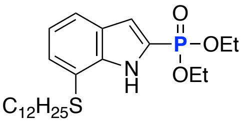

21fa
(150 MHz, $CDCl_3$)

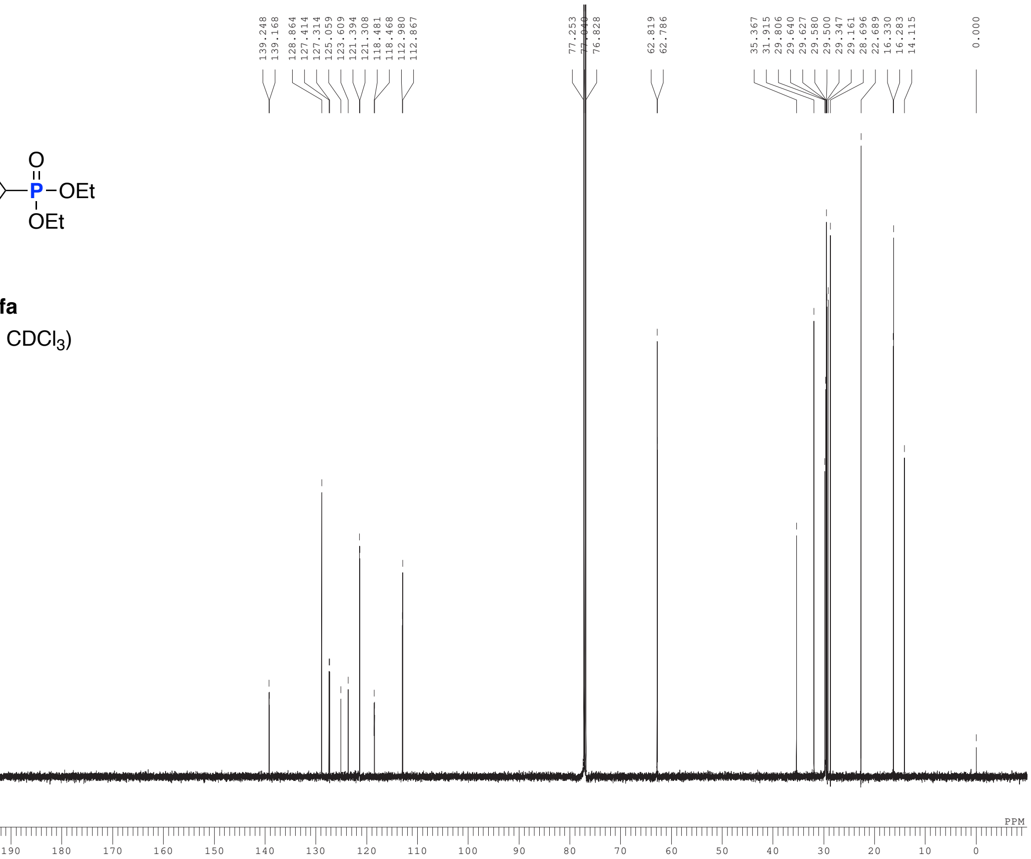

21ga
(400 MHz, $CDCl_3$)

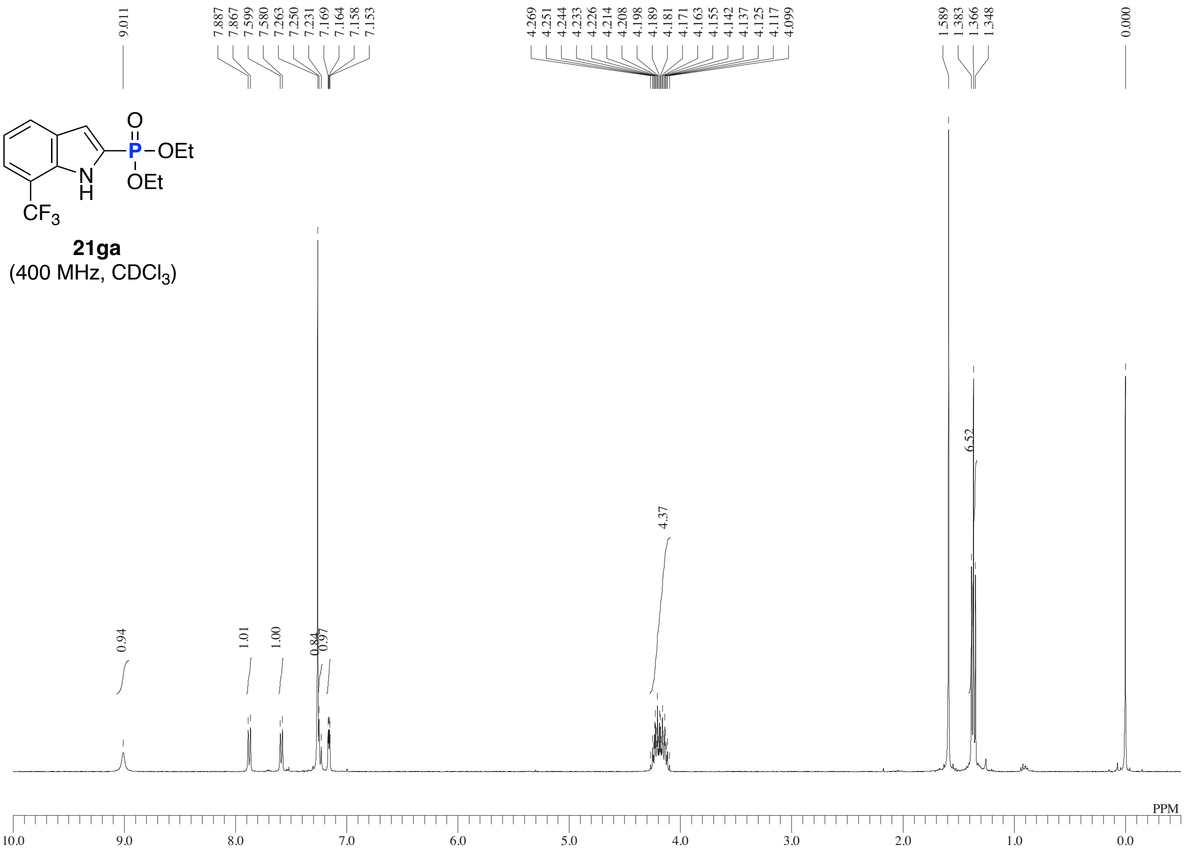

21ga
(150 MHz, $CDCl_3$)

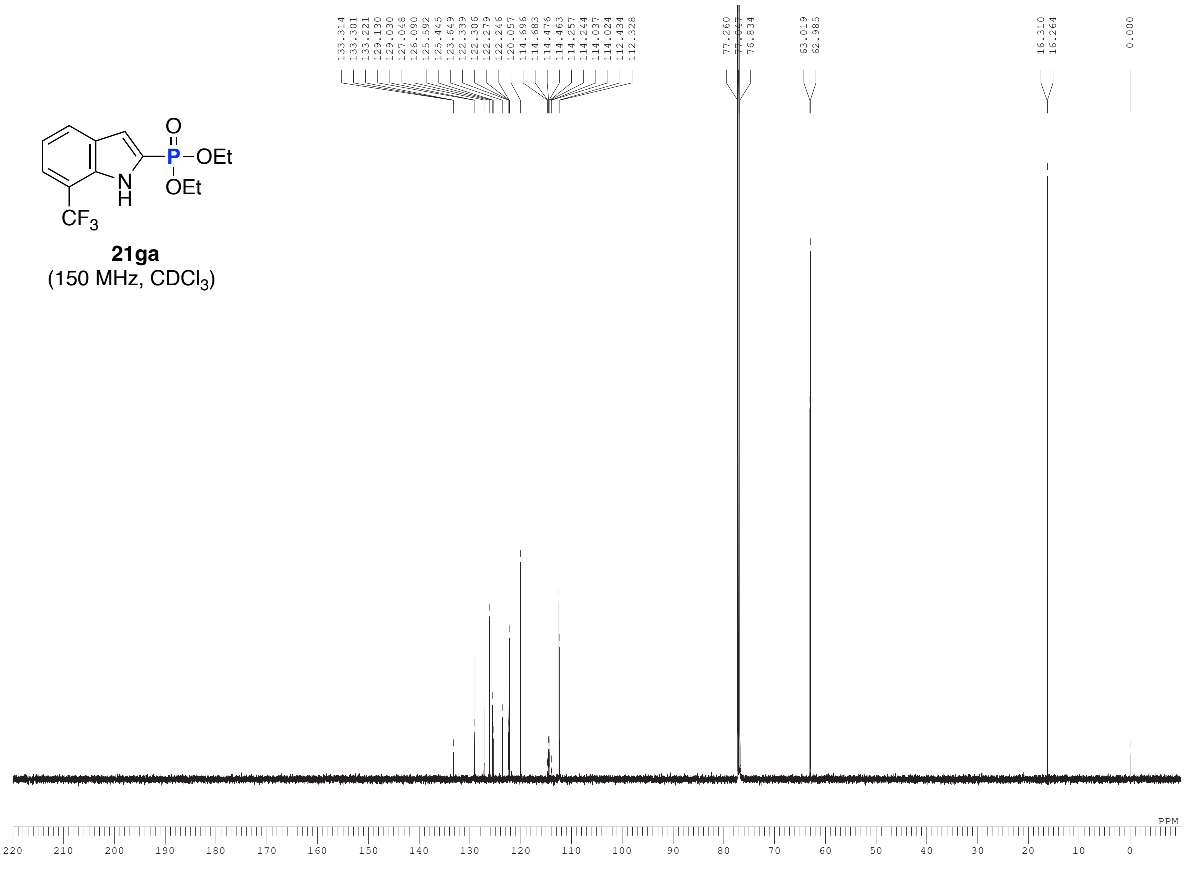

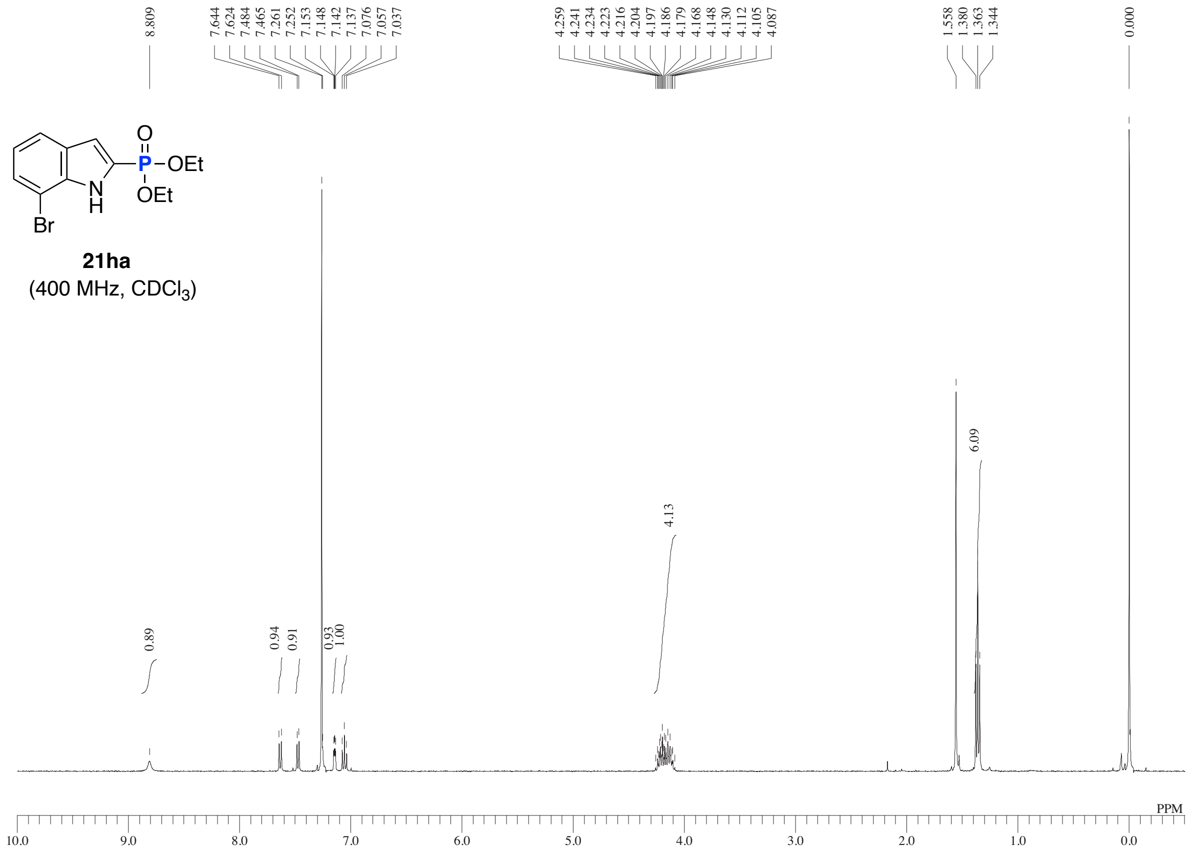

21ha
(400 MHz, $CDCl_3$)

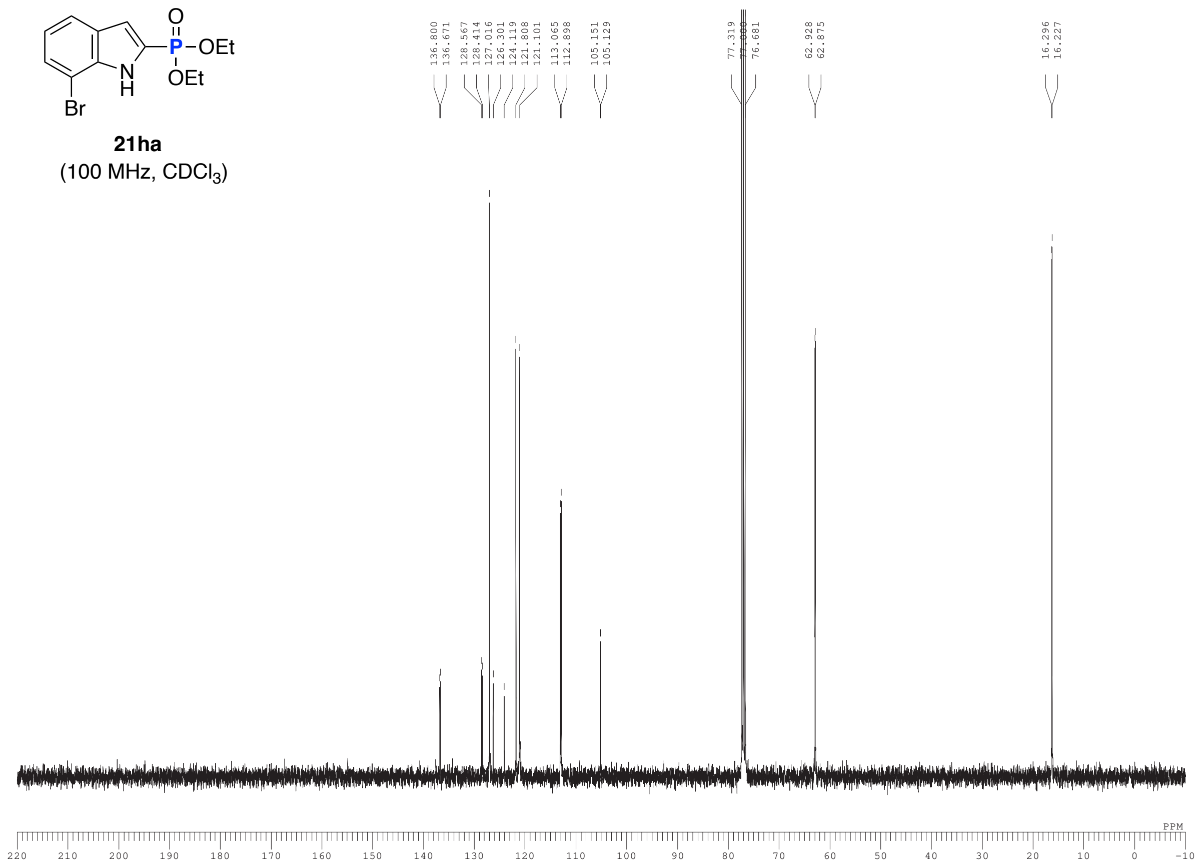

21ha
(100 MHz, $CDCl_3$)

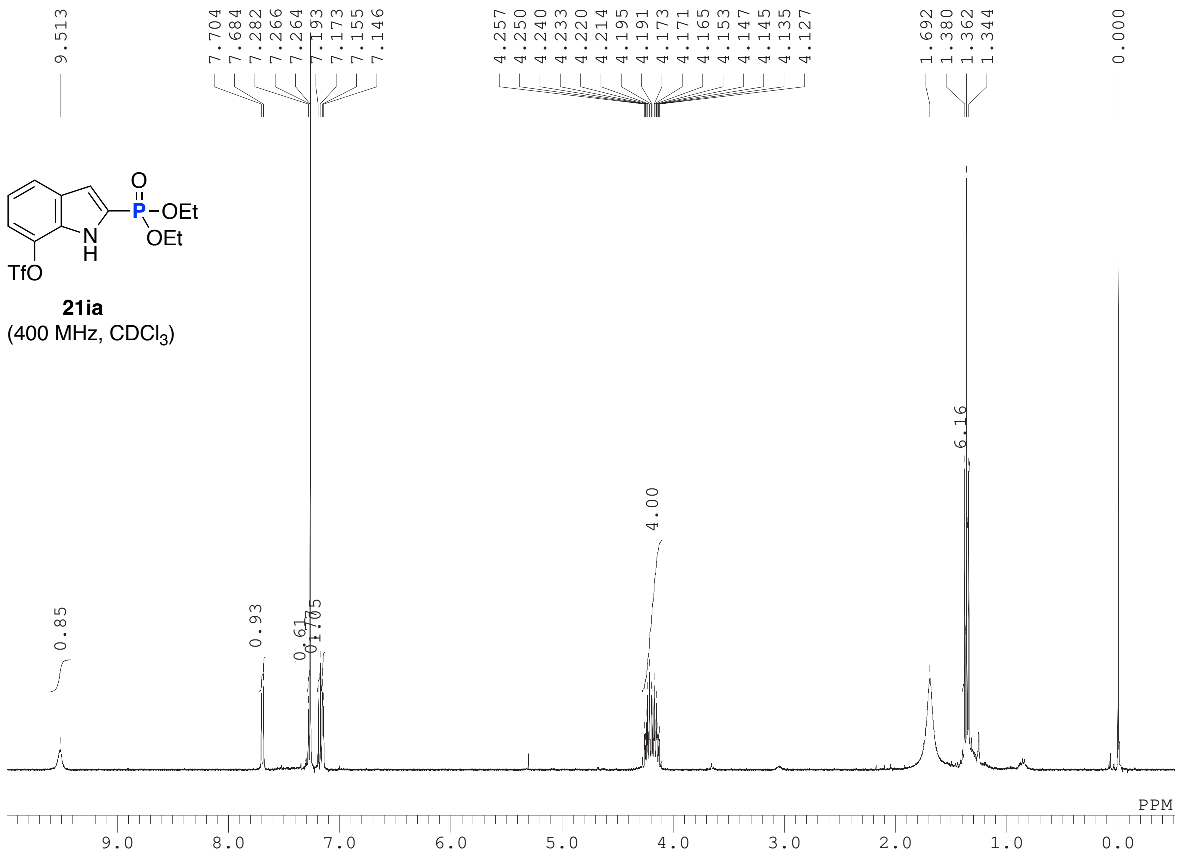

21ia
(400 MHz, $CDCl_3$)

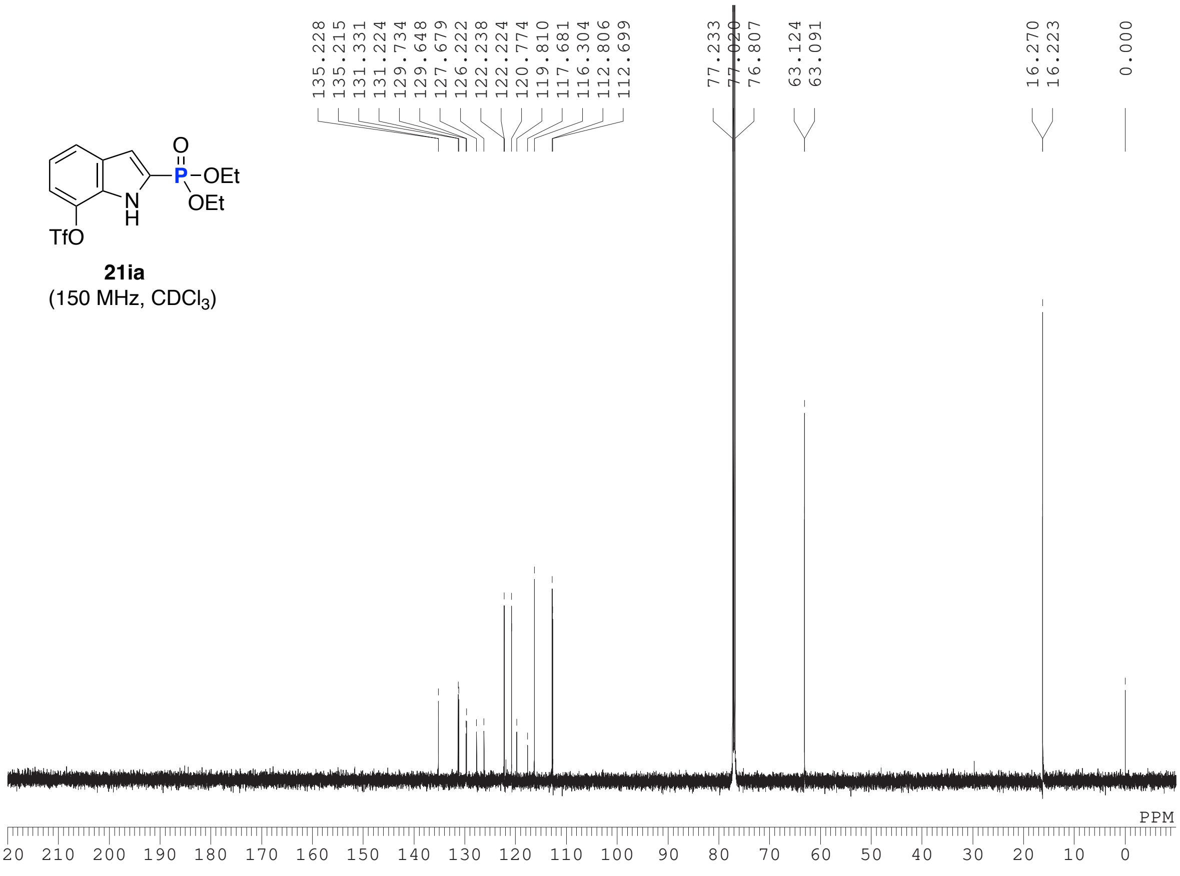

21ia
(150 MHz, $CDCl_3$)

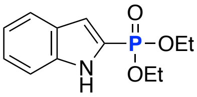

21ja
(400 MHz, $CDCl_3$)

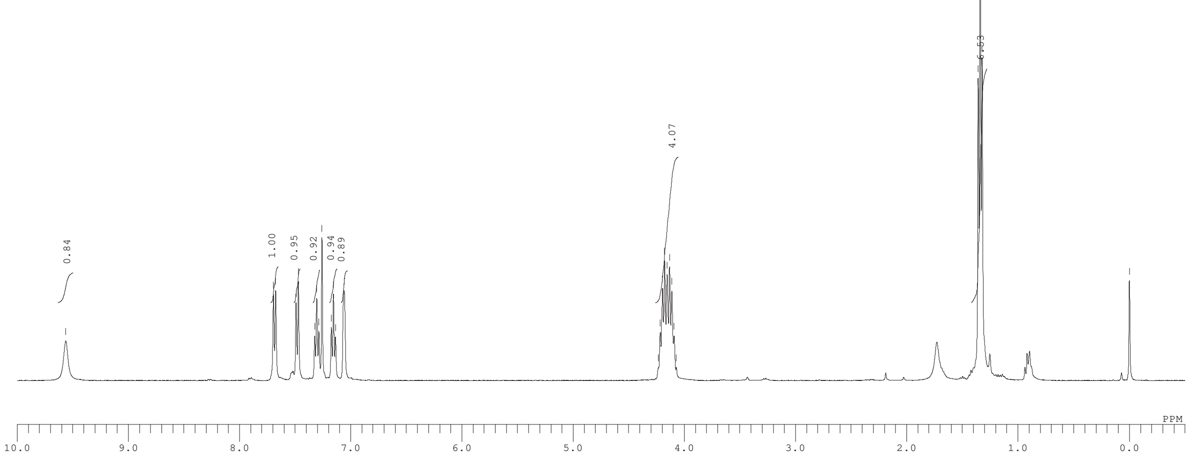

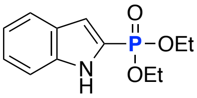

21ja
(100 MHz, $CDCl_3$)

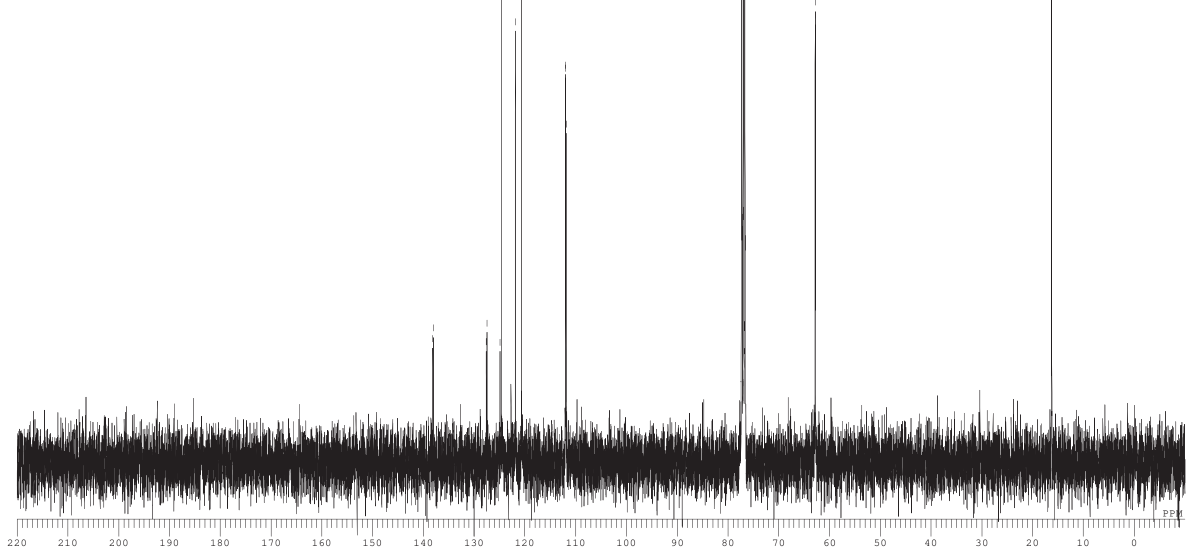

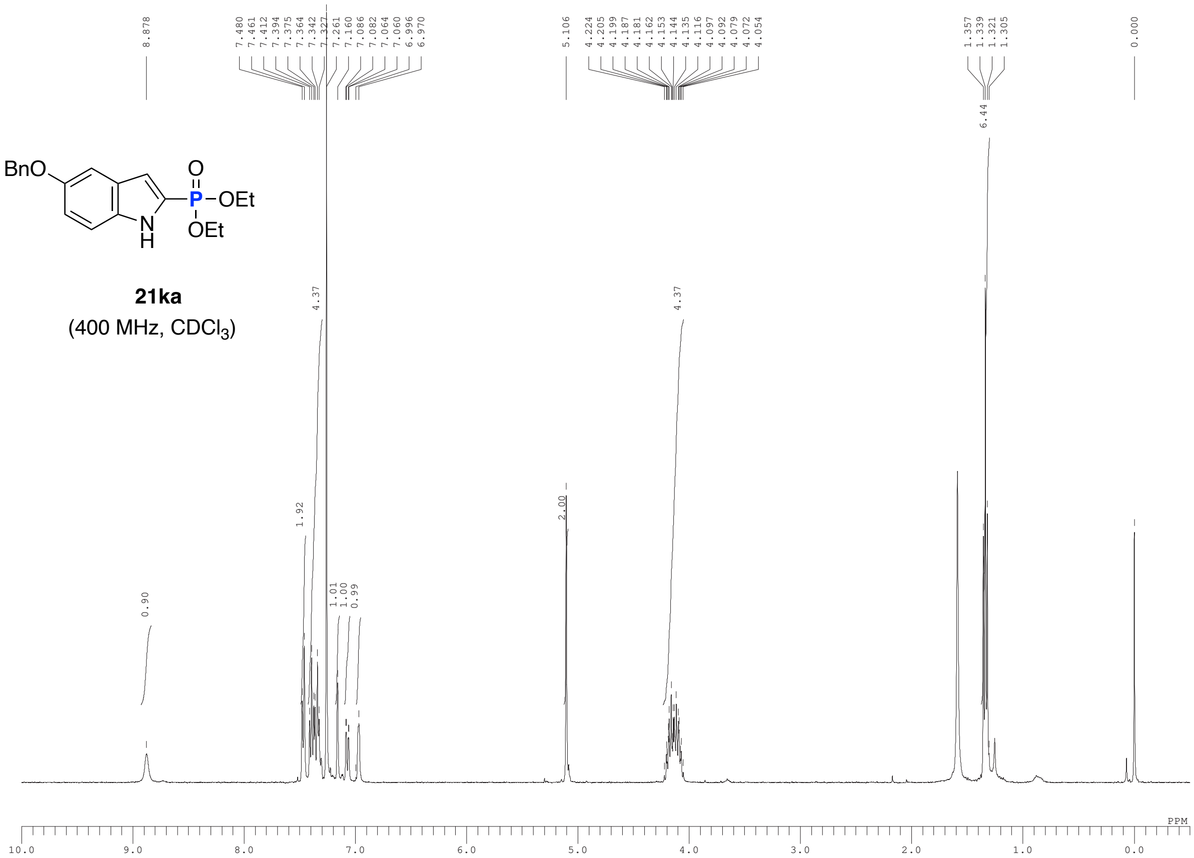
BnO
OEt
OEt
N
H
21ka
(400 MHz, CDCl3)
PPM

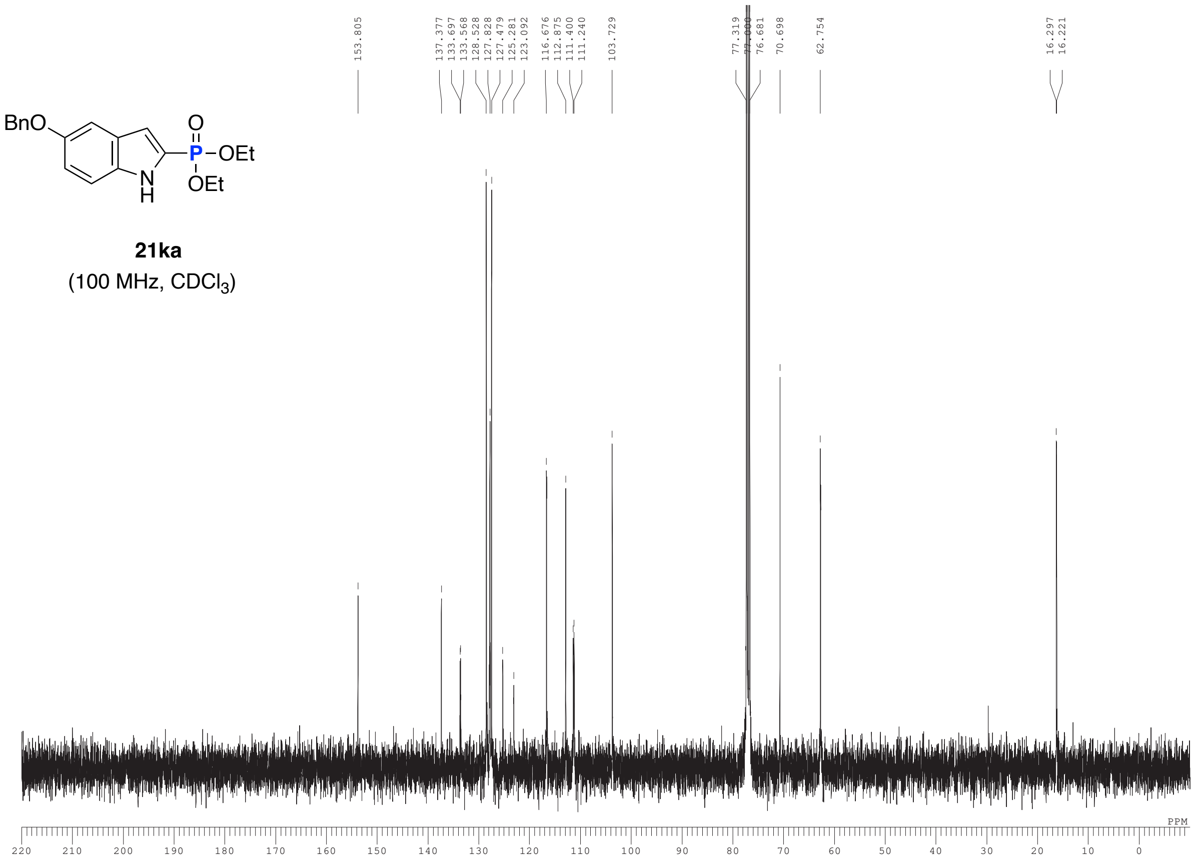
BnO
OEt
OEt
N
H
21ka
(100 MHz, CDCl3)
PPM

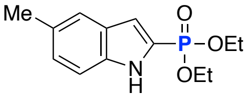

21la
(400 MHz, $CDCl_3$)

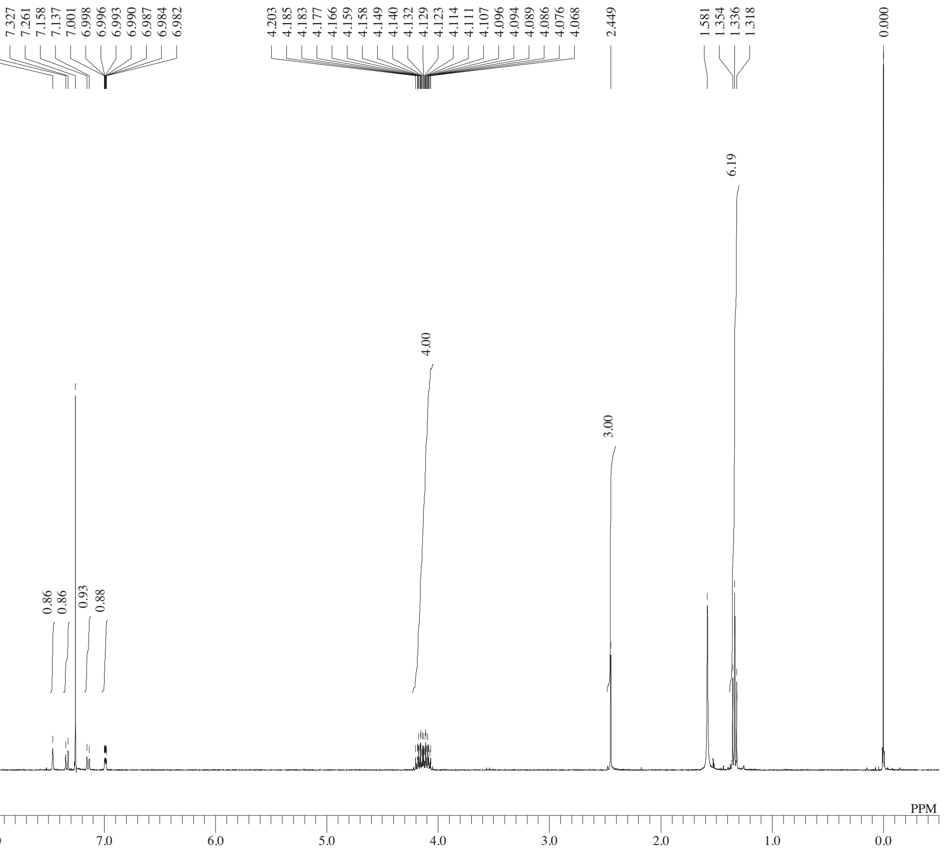

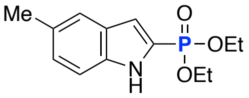

21la
(150 MHz, $CDCl_3$)

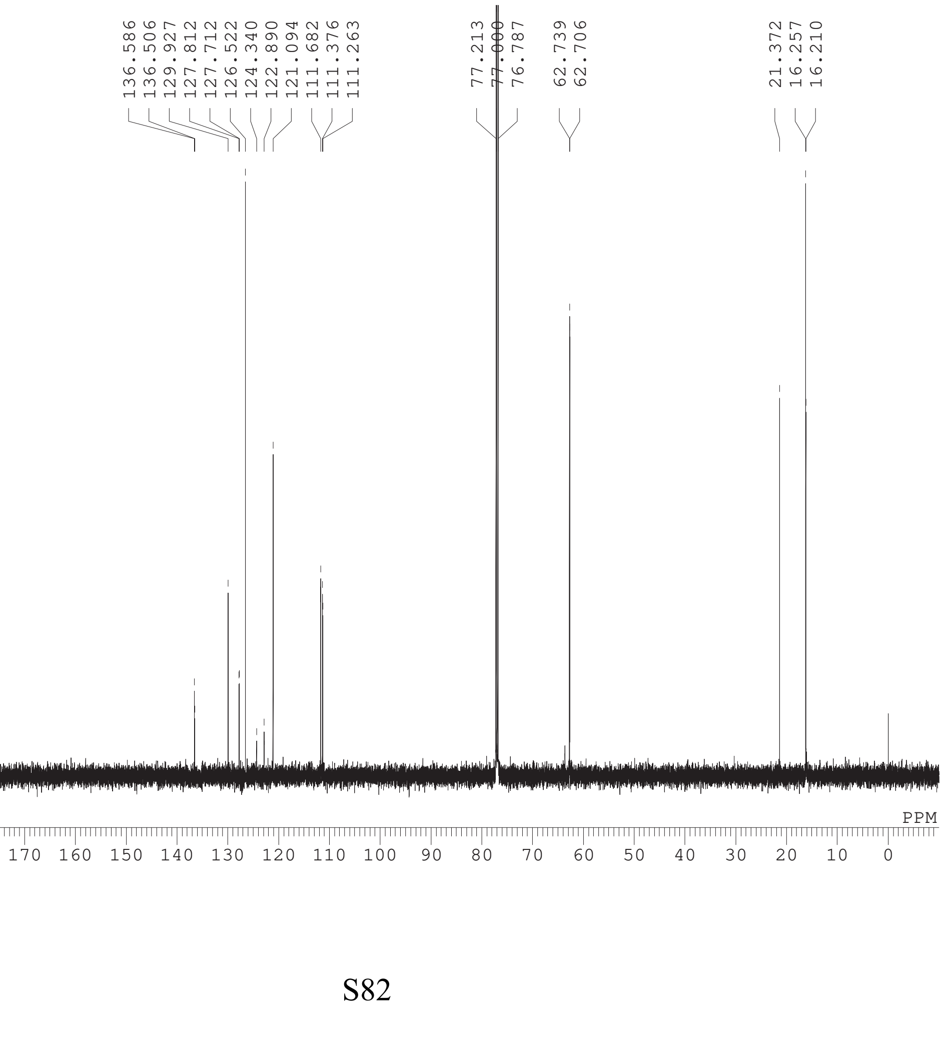

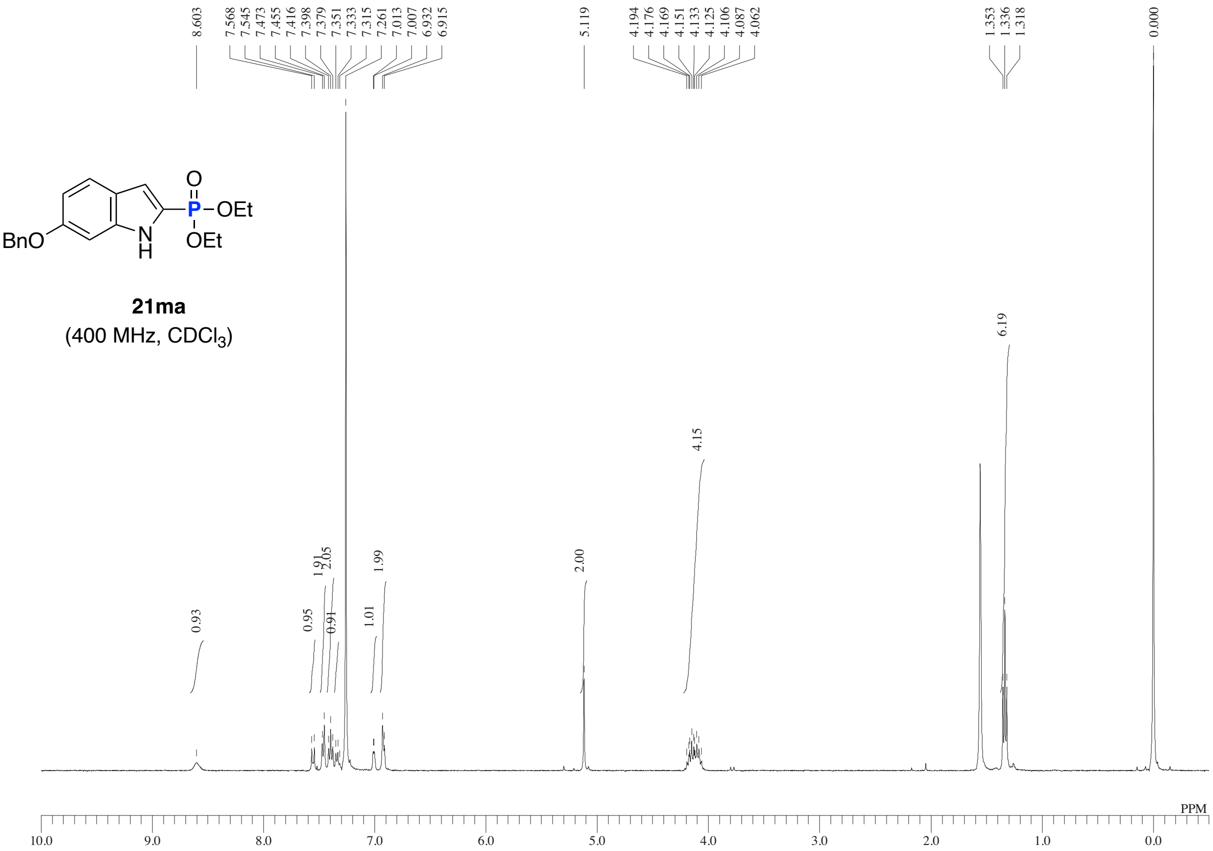

21ma
(400 MHz, $CDCl_3$)

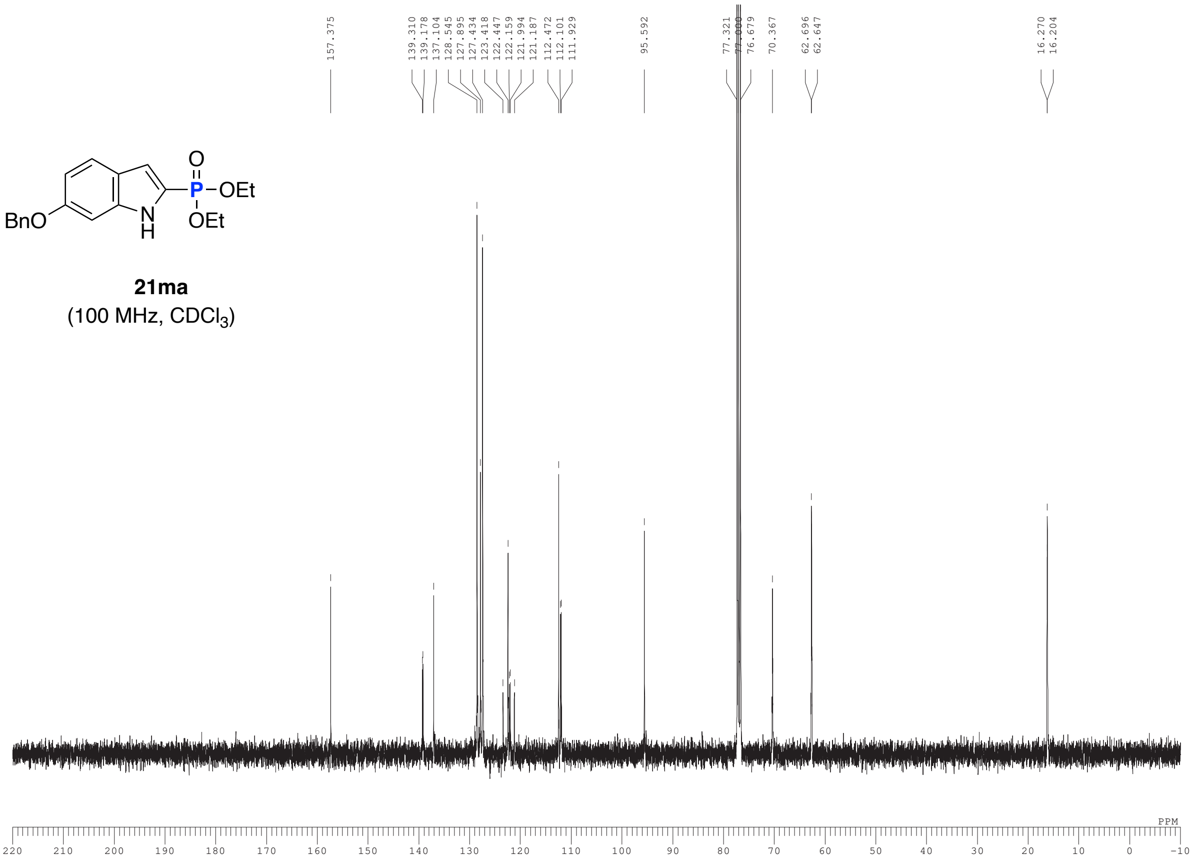

21ma
(100 MHz, $CDCl_3$)

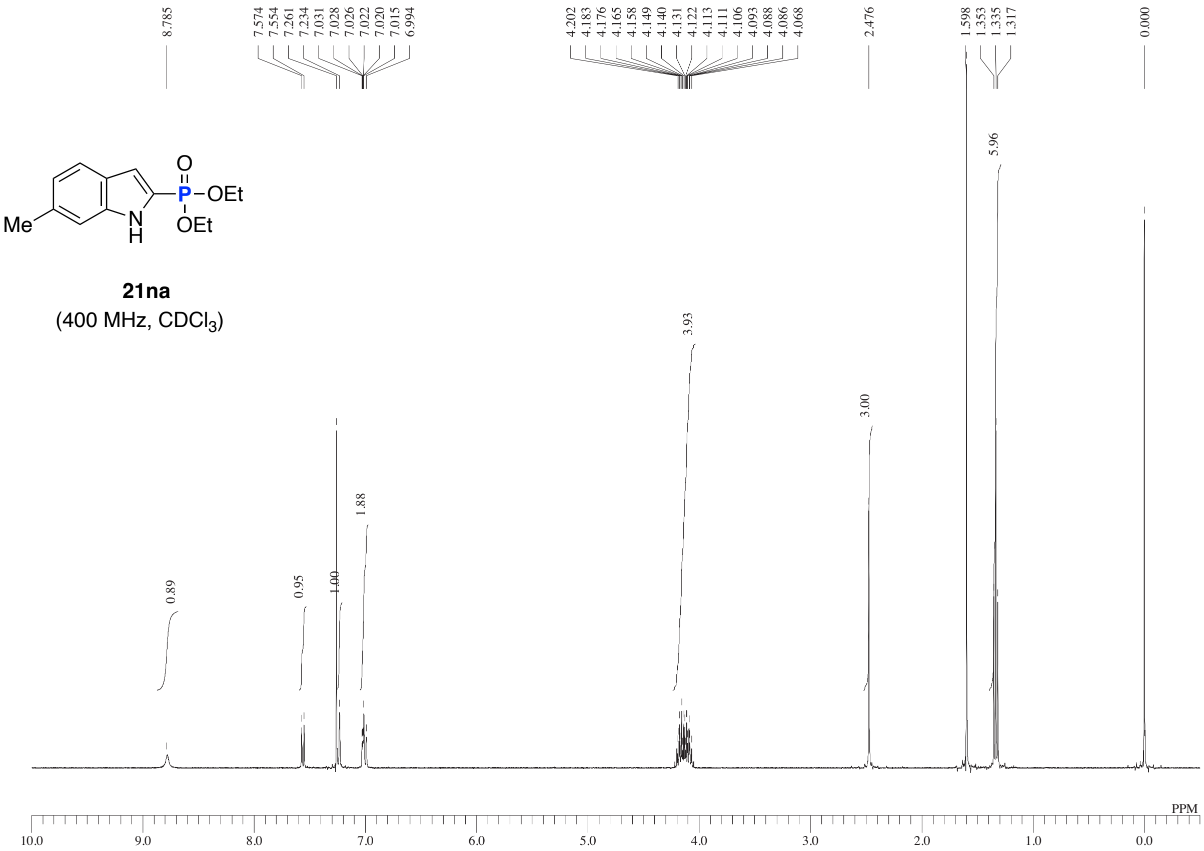
Me
N H
O
P
OEt
OEt
21na
(400 MHz, CDCl3)
8.785
7.574 7.554 7.261 7.234 7.031 7.028 7.026 7.022 7.020 7.015 6.994
4.202 4.183 4.176 4.165 4.158 4.149 4.140 4.131 4.122 4.113 4.111 4.106 4.093 4.088 4.086 4.068
2.476
1.598 1.353 1.335 1.317
0.000
0.89 0.95 1.00 1.88 3.93 3.00 5.96
PPM
10.0 9.0 8.0 7.0 6.0 5.0 4.0 3.0 2.0 1.0 0.0

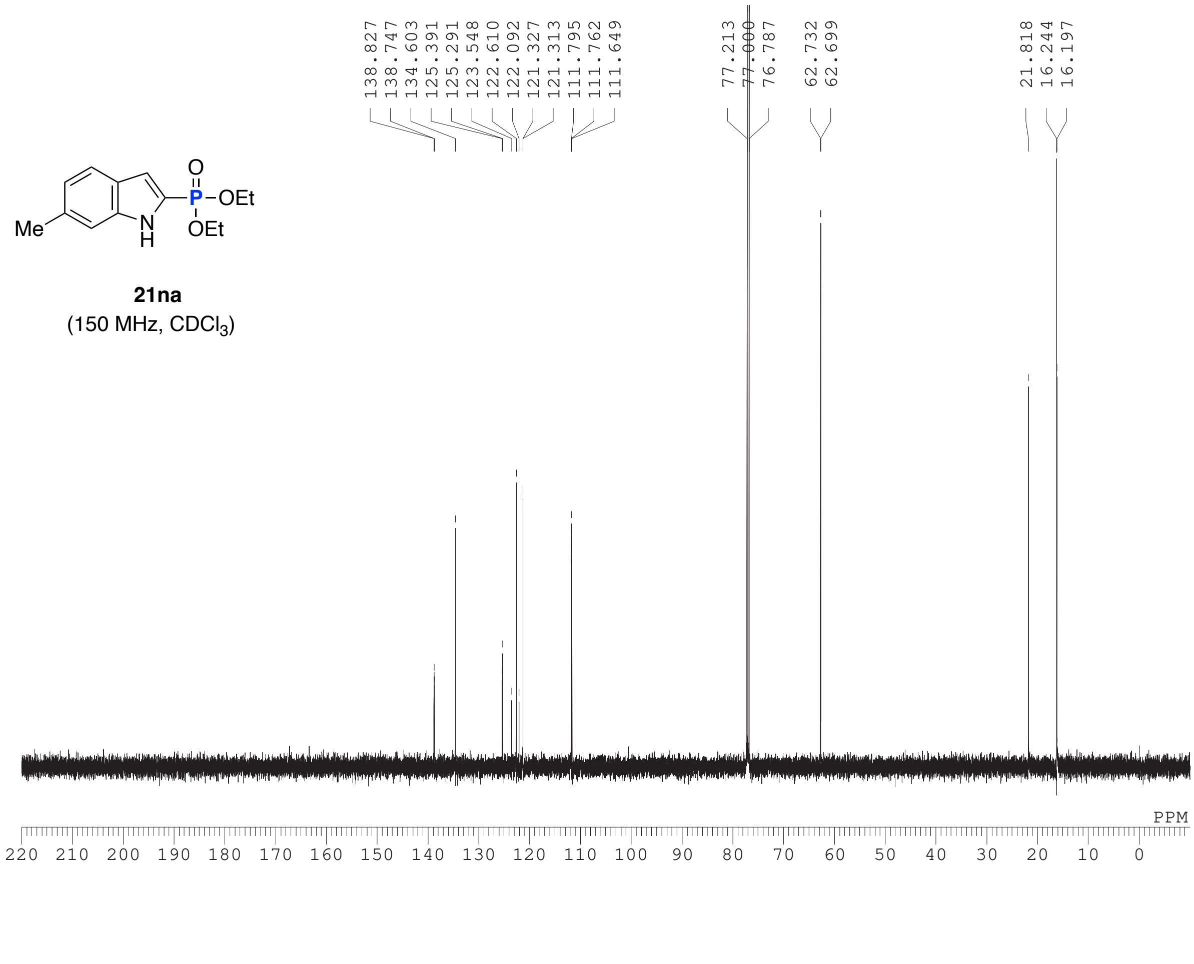
Me
N H
O
P
OEt
OEt
21na
(150 MHz, CDCl3)
138.827 138.747 134.603 125.391 125.291 123.548 122.610 122.092 121.327 121.313 111.795 111.762 111.649
77.213 77.000 76.787
62.732 62.699
21.818 16.244 16.197
PPM
220 210 200 190 180 170 160 150 140 130 120 110 100 90 80 70 60 50 40 30 20 10 0

21oa
(400 MHz, $CDCl_3$)

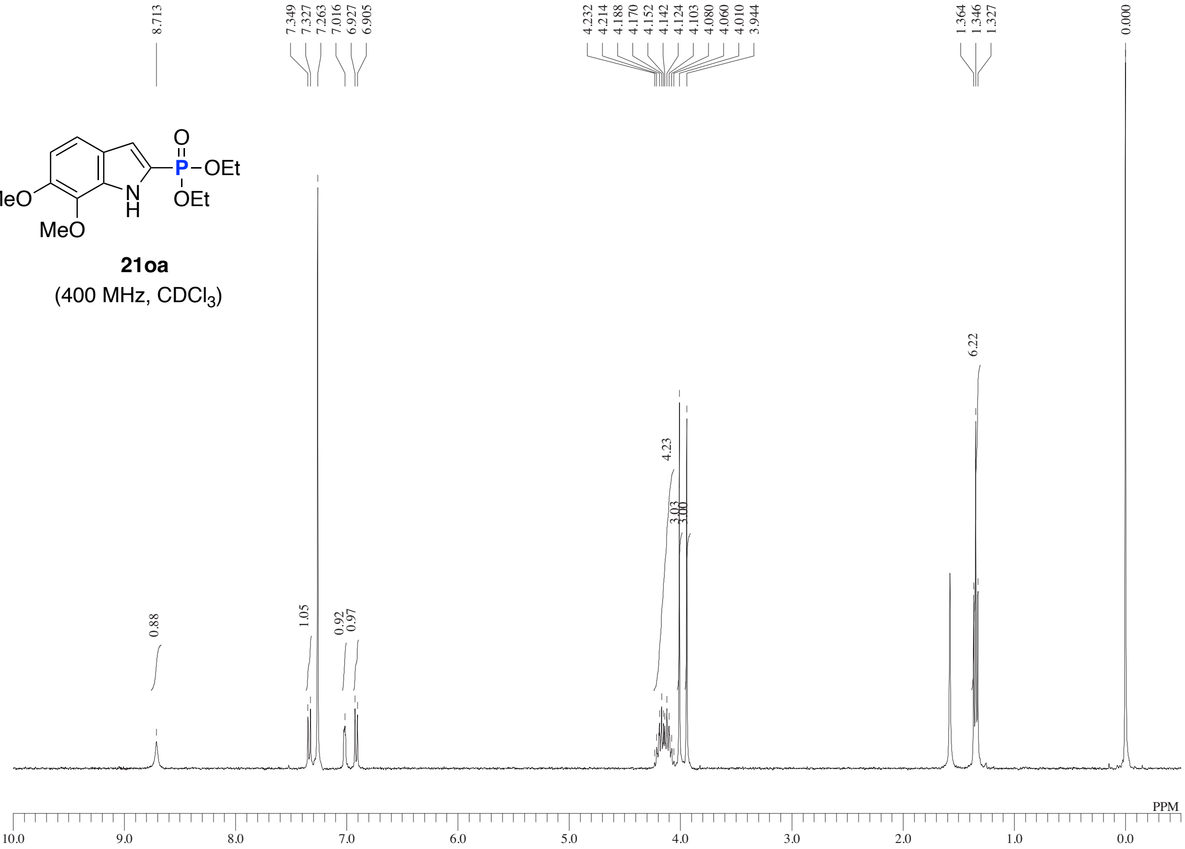

21oa
(100 MHz, $CDCl_3$)

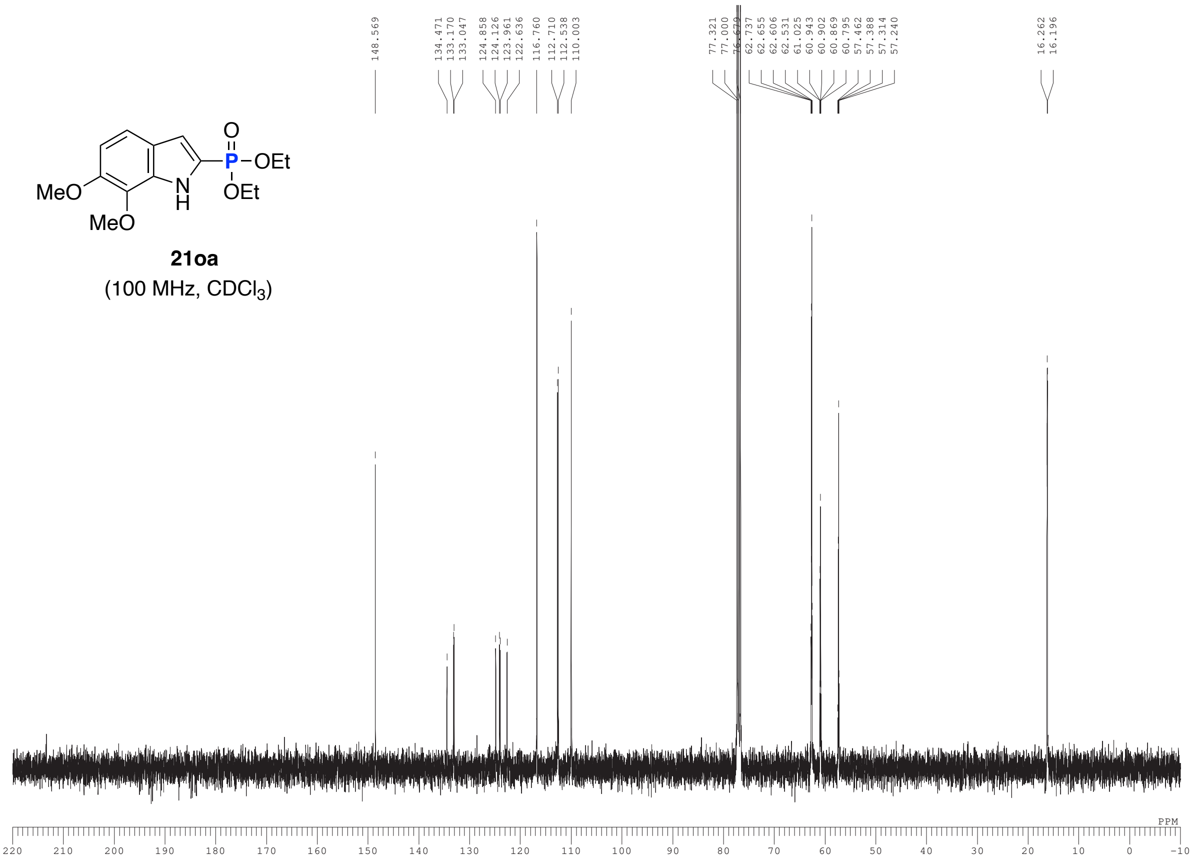

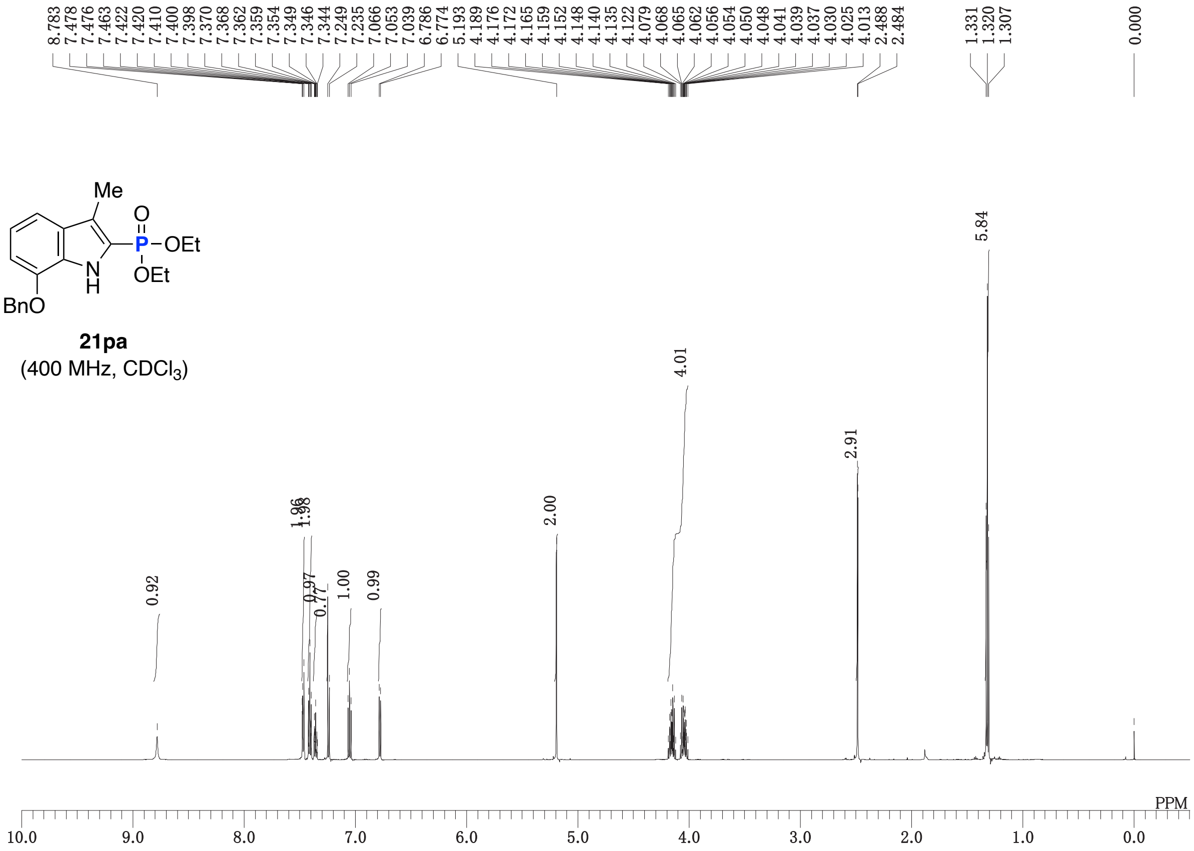

21pa
(400 MHz, $CDCl_3$)

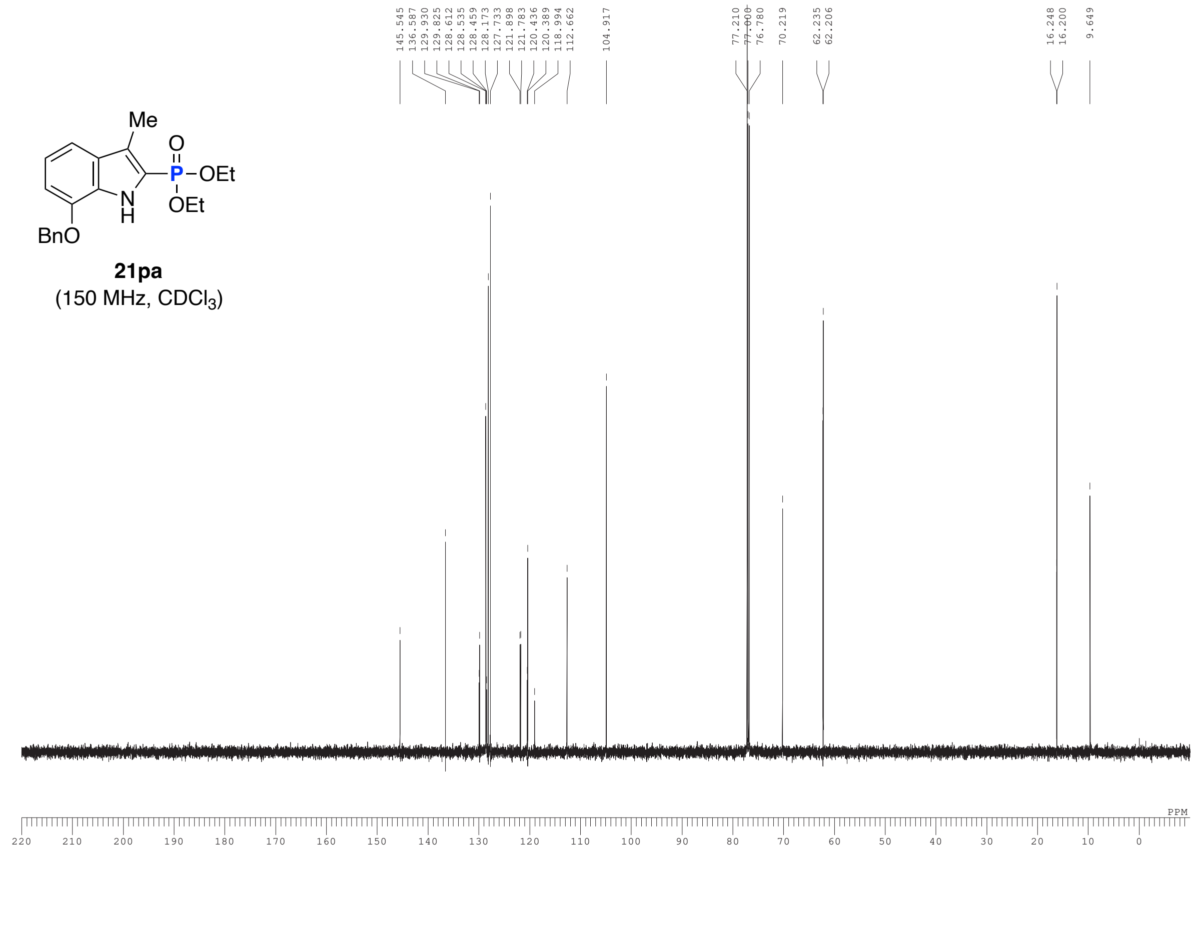

21pa
(150 MHz, $CDCl_3$)

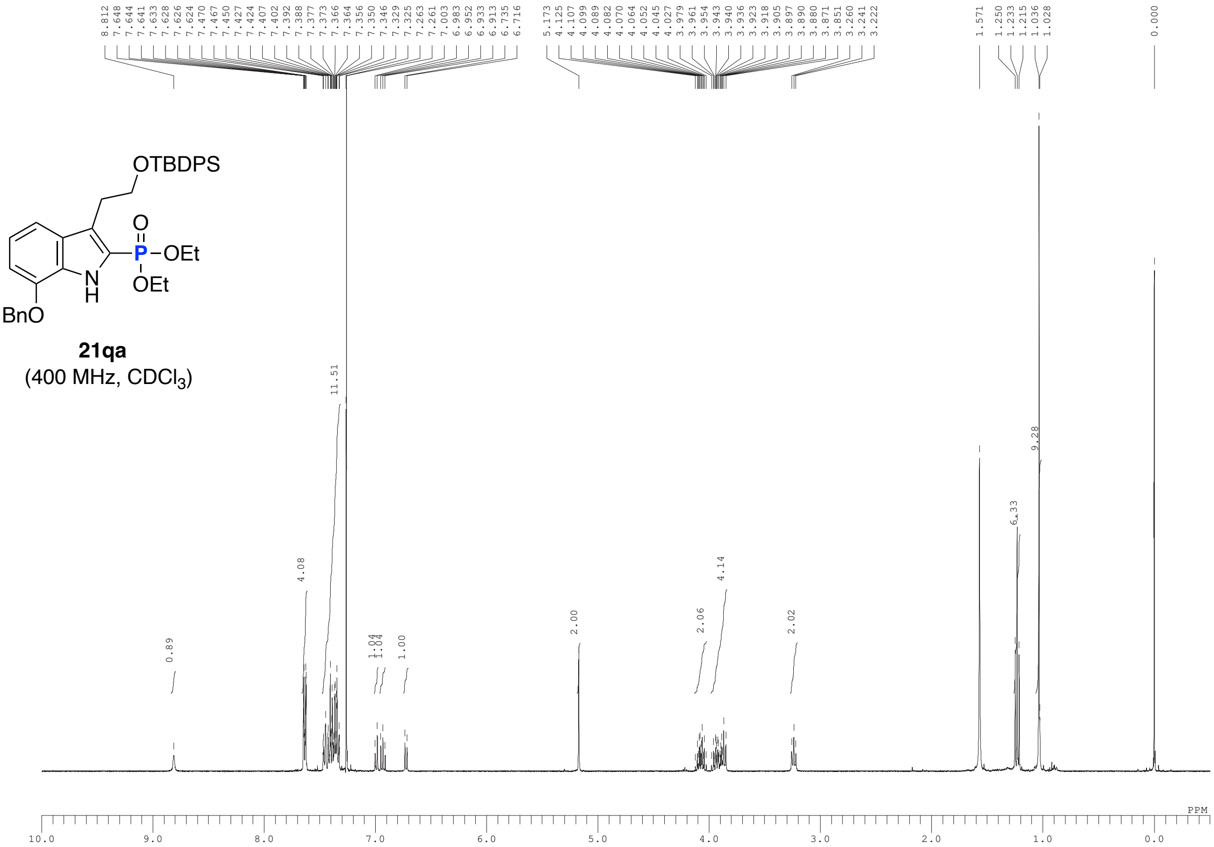
8.812 7.648 7.644 7.641 7.633 7.628 7.626 7.624 7.470 7.467 7.450 7.427 7.424 7.407 7.402 7.392 7.388 7.377 7.373 7.366 7.364 7.356 7.350 7.346 7.329 7.325 7.263 7.261 7.003 6.983 6.952 6.933 6.913 6.735 6.716
5.173 4.125 4.107 4.099 4.089 4.082 4.070 4.064 4.052 4.045 4.027 3.979 3.961 3.954 3.943 3.940 3.936 3.923 3.918 3.905 3.897 3.890 3.880 3.871 3.851 3.260 3.241 3.222
1.571 1.250 1.233 1.215 1.036 1.028
0.000
OTBDPS
O
P
OEt
N
H
OEt
BnO
21qa
(400 MHz, $CDCl_3$)
0.89
4.08
11.51
1.04
1.04
1.00
2.00
2.06
4.14
2.02
6.33
9.28
PPM
10.0 9.0 8.0 7.0 6.0 5.0 4.0 3.0 2.0 1.0 0.0

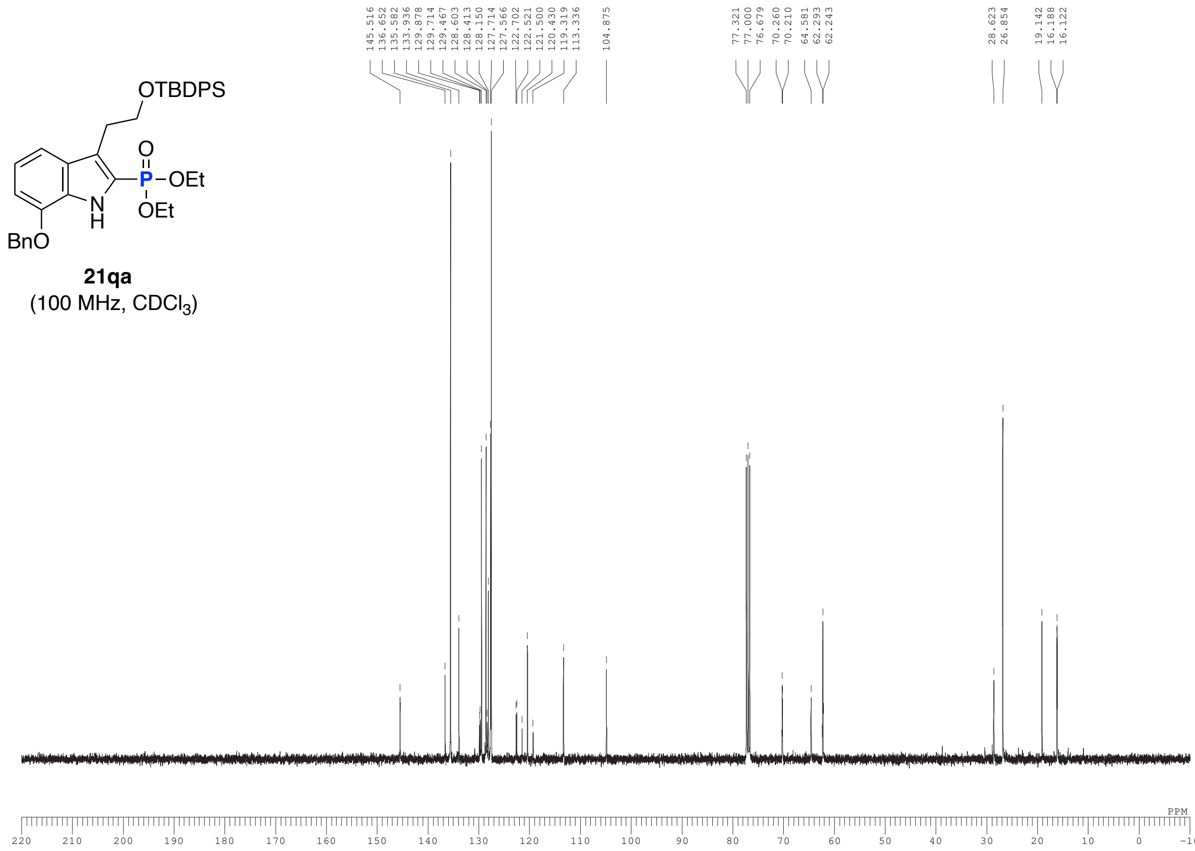
145.516 136.652 135.582 133.936 129.878 129.714 129.467 128.603 128.413 128.150 127.714 127.566 122.702 122.521 121.500 120.430 119.319 113.336 104.875
77.321 77.000 76.679 70.260 70.210 64.581 62.293 62.243
28.623 26.854 19.142 16.188 16.122
OTBDPS
O
P
OEt
N
H
OEt
BnO
21qa
(100 MHz, $CDCl_3$)
PPM
220 210 200 190 180 170 160 150 140 130 120 110 100 90 80 70 60 50 40 30 20 10 0 −10

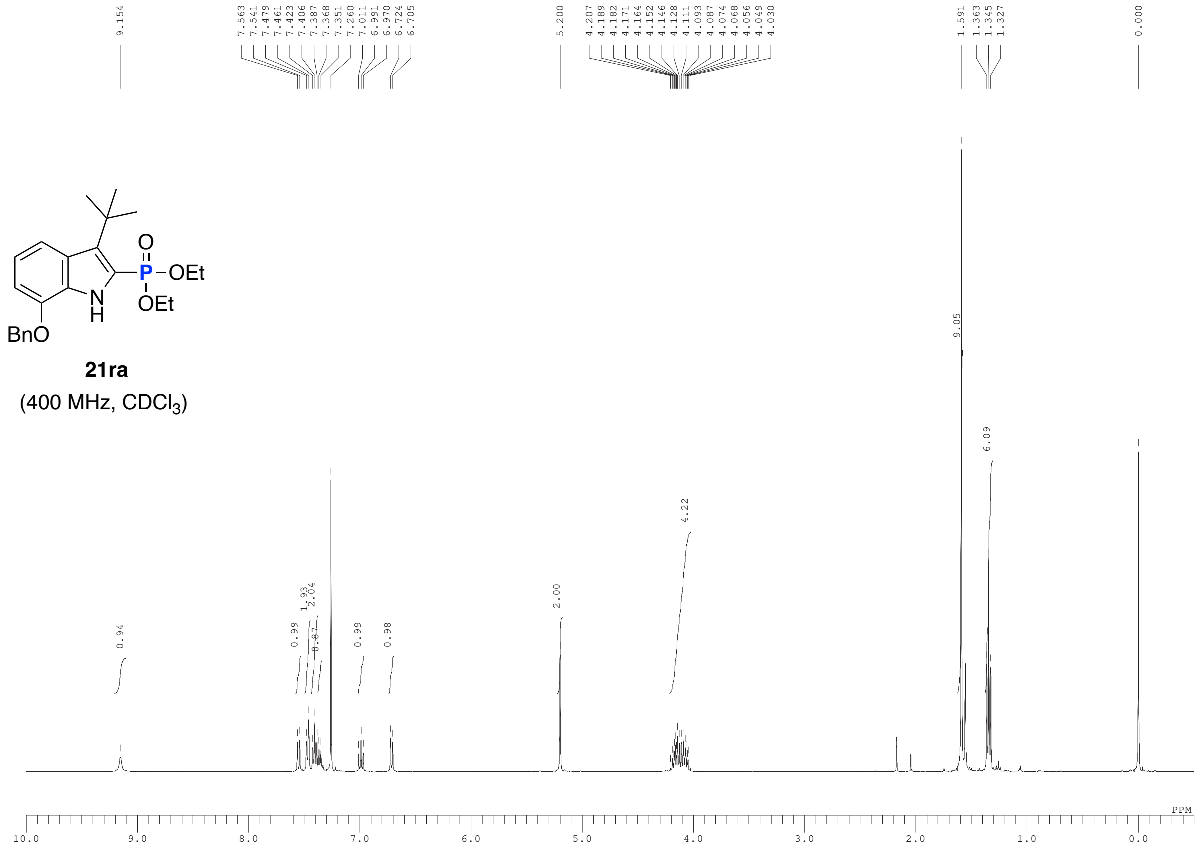
O
P
OEt
OEt
N
H
BnO
21ra
(400 MHz, CDCl3)
9.154
7.563
7.541
7.479
7.461
7.423
7.406
7.387
7.368
7.351
7.260
7.011
6.991
6.970
6.724
6.705
5.200
4.207
4.189
4.182
4.171
4.164
4.152
4.146
4.128
4.111
4.093
4.087
4.074
4.068
4.056
4.049
4.030
1.591
1.363
1.345
1.327
0.000
0.94
0.99
1.93
2.04
0.87
0.99
0.98
2.00
4.22
9.05
6.09
PPM
10.0
9.0
8.0
7.0
6.0
5.0
4.0
3.0
2.0
1.0
0.0

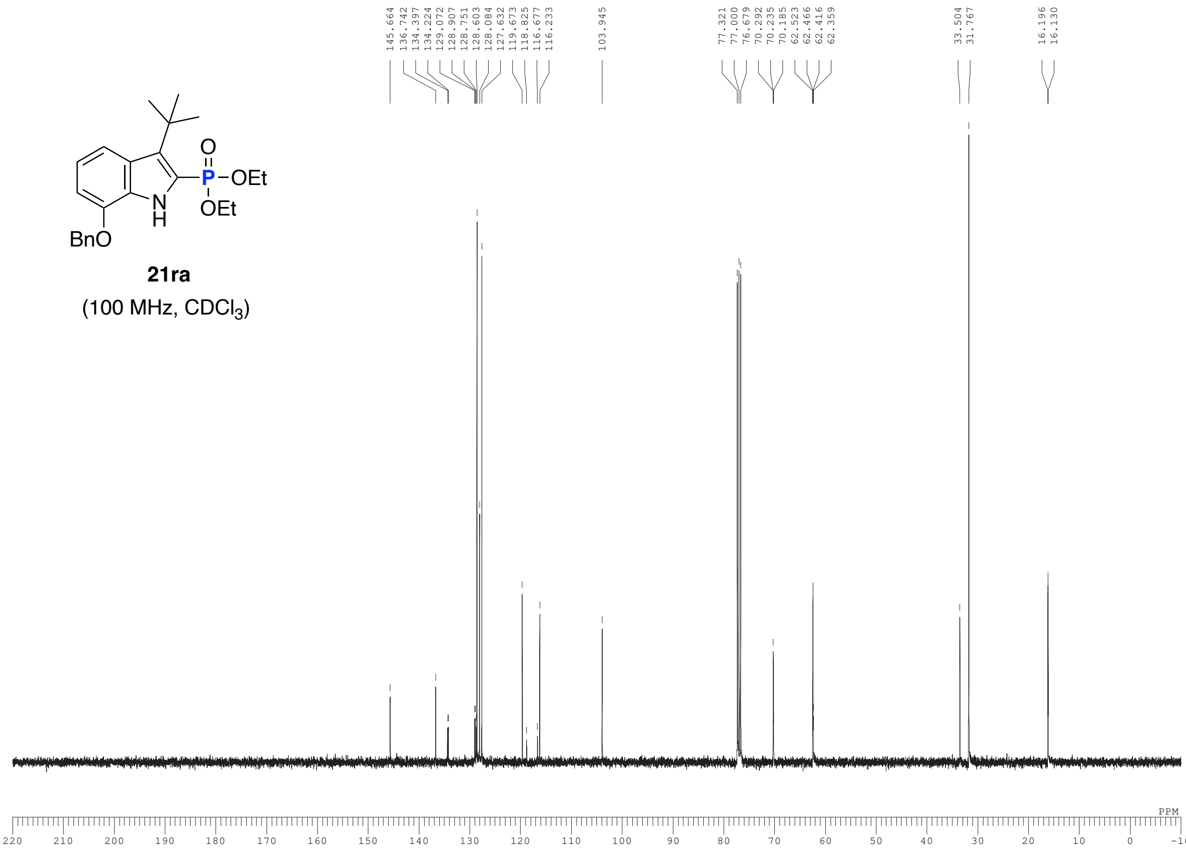
O
P
OEt
OEt
N
H
BnO
21ra
(100 MHz, CDCl3)
145.664
136.742
134.397
134.224
129.072
128.907
128.751
128.603
128.084
127.632
119.673
118.825
116.677
116.233
103.945
77.321
77.000
76.679
70.292
70.235
70.185
62.523
62.466
62.416
62.359
33.504
31.767
16.196
16.130
PPM
220
210
200
190
180
170
160
150
140
130
120
110
100
90
80
70
60
50
40
30
20
10
0
−10

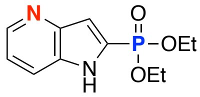

21sa
(600 MHz, $CDCl_3$)

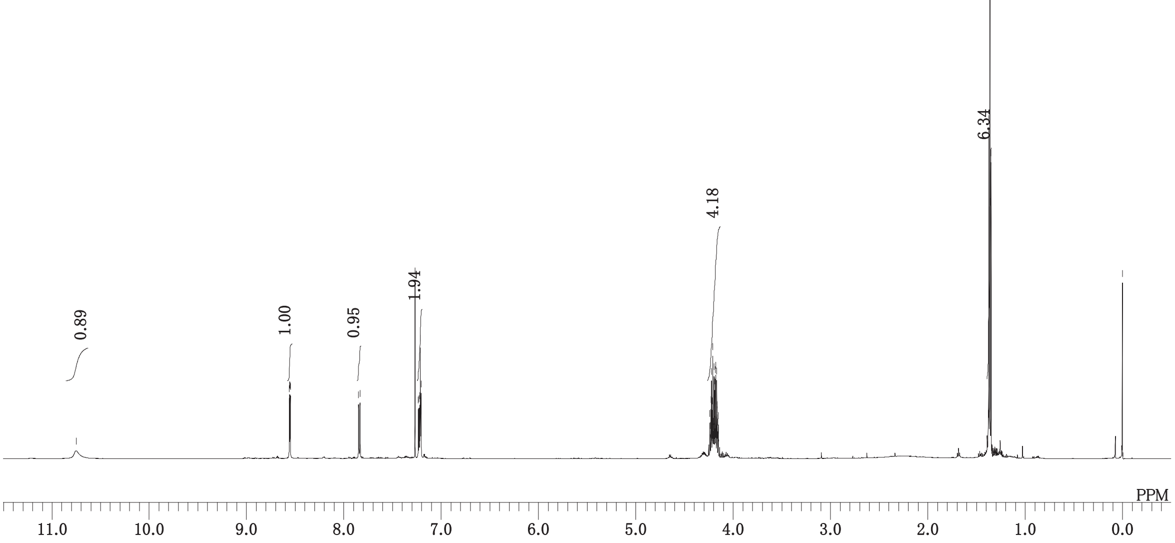

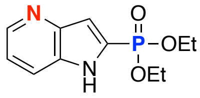

21sa
(150 MHz, $CDCl_3$)

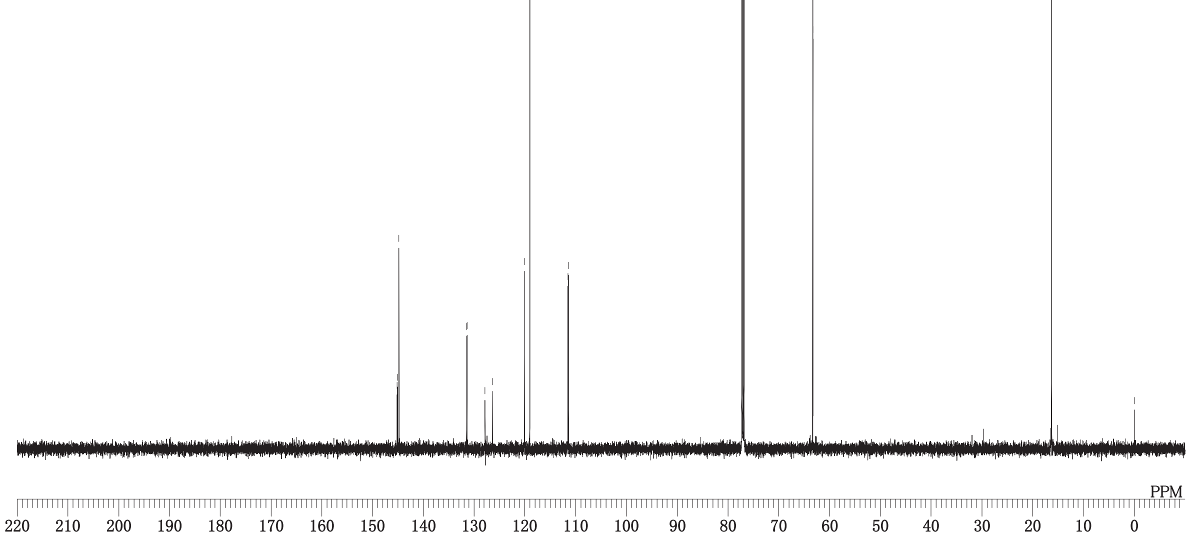

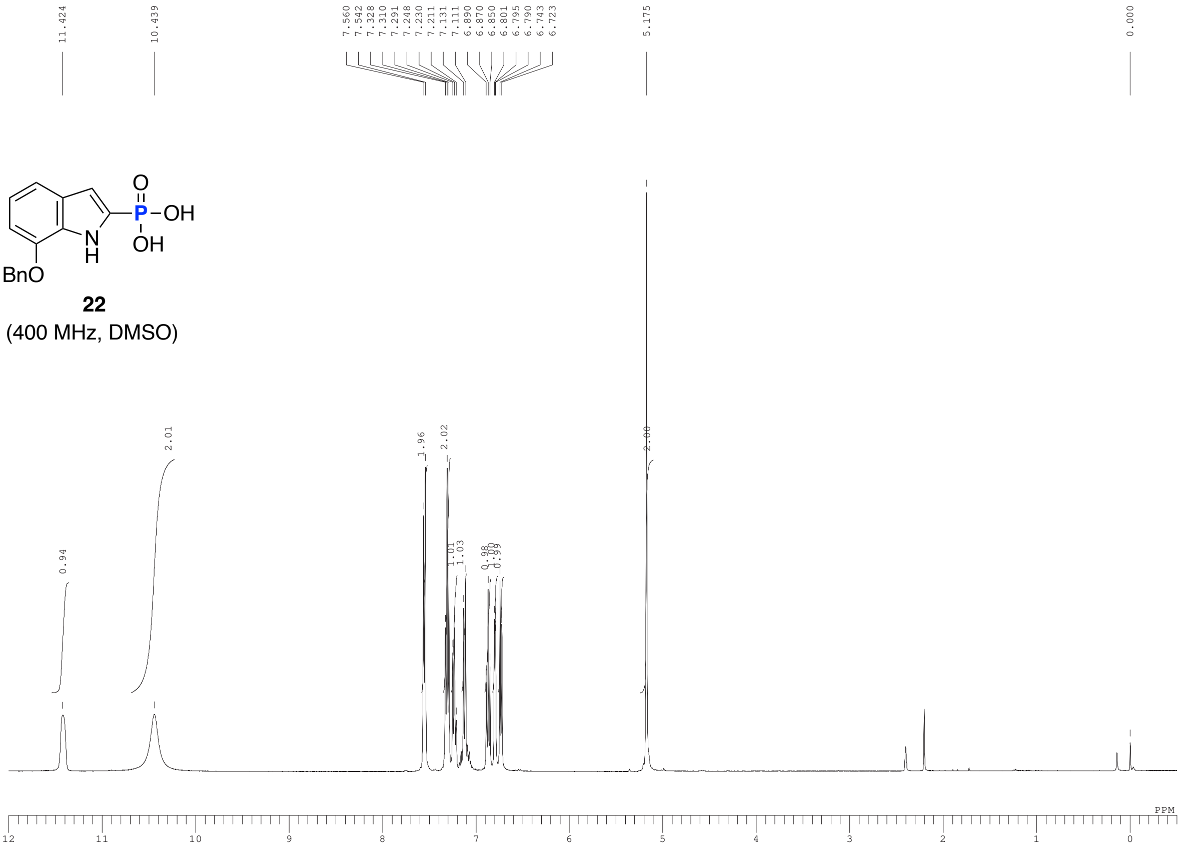

22
(400 MHz, DMSO)

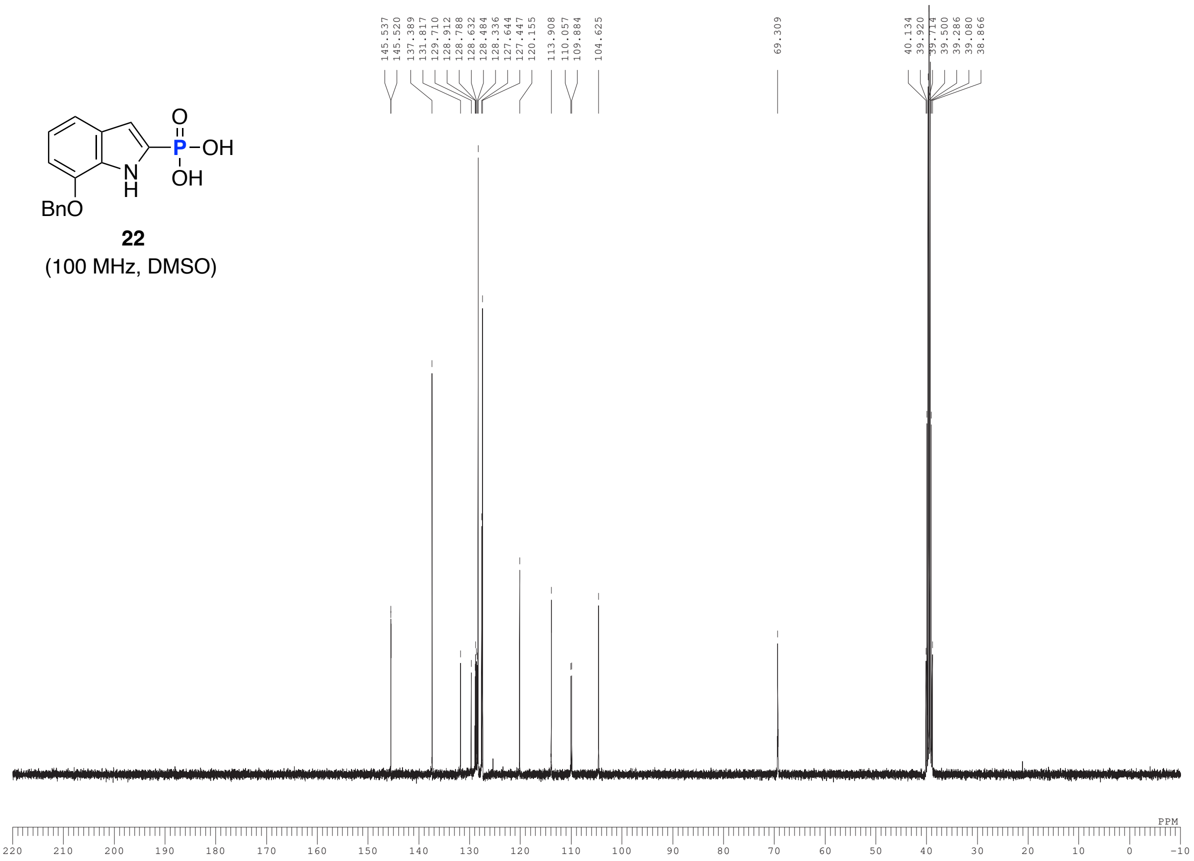

22
(100 MHz, DMSO)

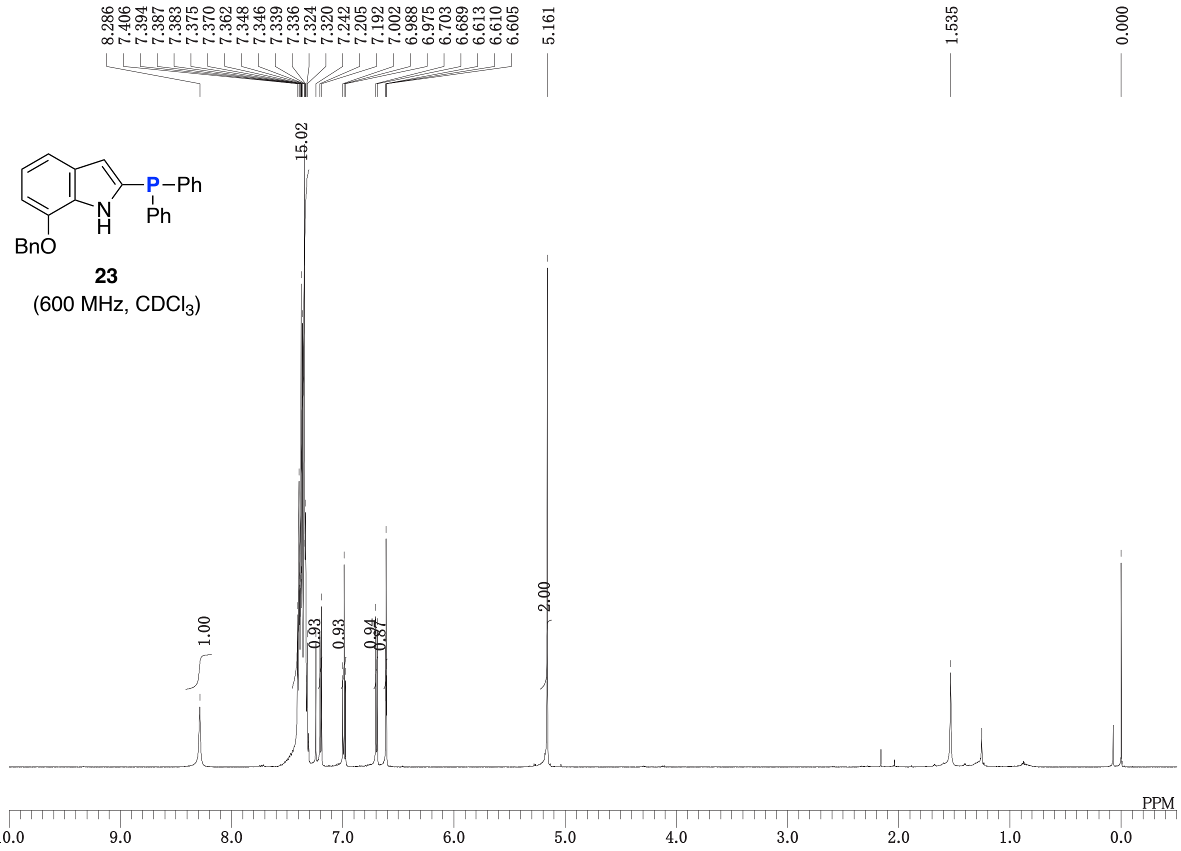
8.286
7.406
7.394
7.387
7.383
7.375
7.370
7.362
7.348
7.346
7.339
7.336
7.324
7.320
7.242
7.205
7.192
7.002
6.988
6.975
6.703
6.689
6.613
6.610
6.605
5.161
1.535
0.000
P
Ph
Ph
N
H
BnO
23
(600 MHz, CDCl3)
15.02
1.00
0.93
0.93
0.94
0.87
2.00
PPM
10.0
9.0
8.0
7.0
6.0
5.0
4.0
3.0
2.0
1.0
0.0

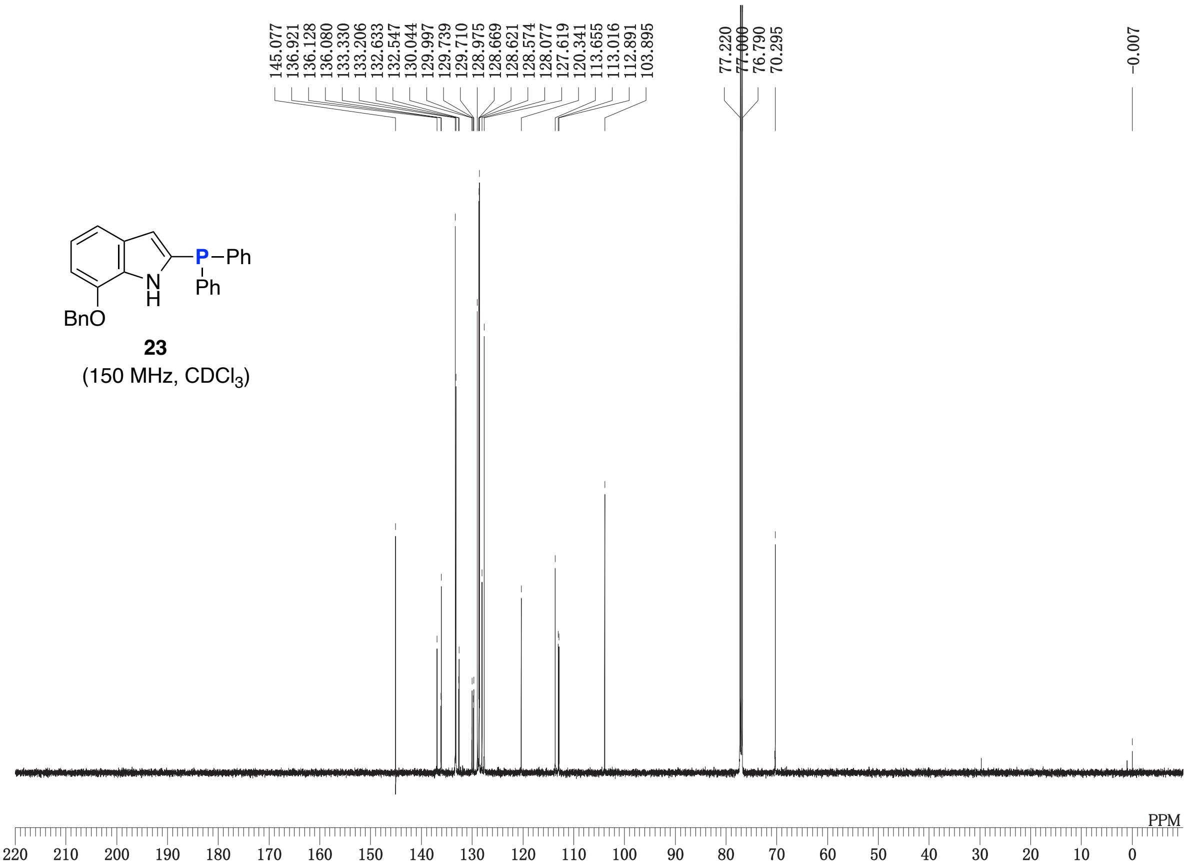
145.077
136.921
136.128
136.080
133.330
133.206
132.633
132.547
130.044
129.997
129.739
129.710
128.975
128.669
128.621
128.574
128.077
127.619
120.341
113.655
113.016
112.891
103.895
77.220
77.000
76.790
70.295
−0.007
P
Ph
Ph
N
H
BnO
23
(150 MHz, CDCl3)
PPM
220
210
200
190
180
170
160
150
140
130
120
110
100
90
80
70
60
50
40
30
20
10
0

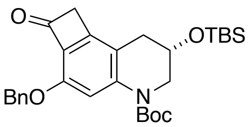

28
(400 MHz, $CDCl_3$)

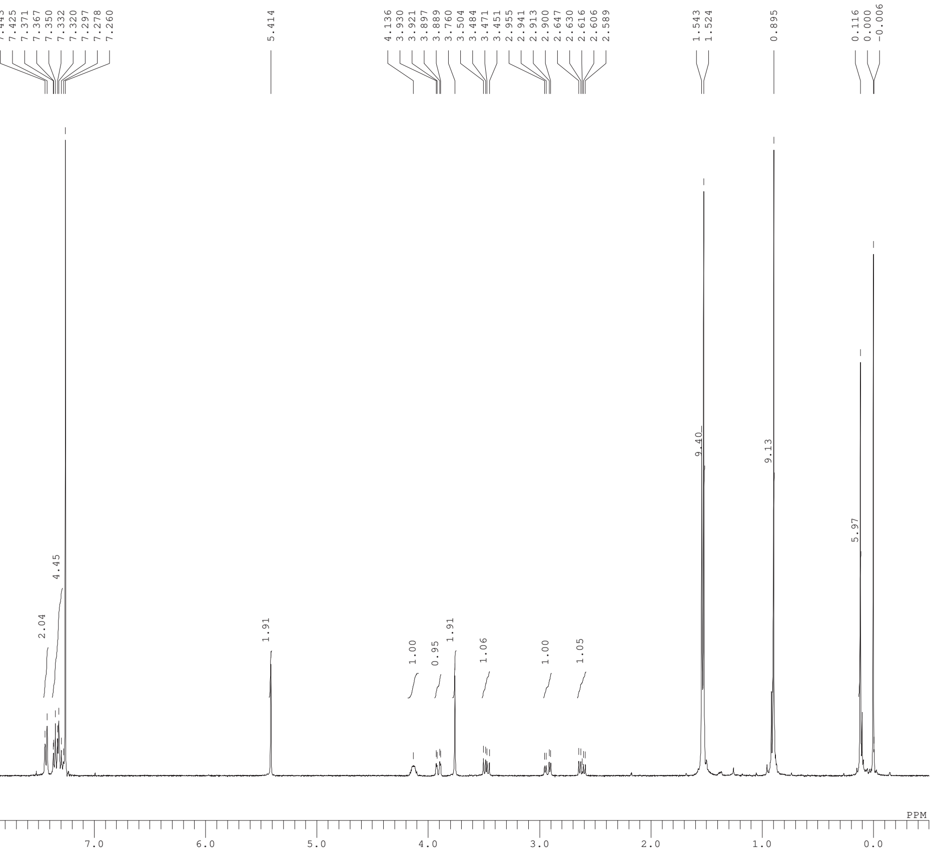

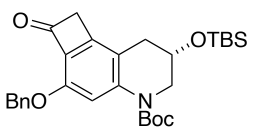

28
(100 MHz, $CDCl_3$)

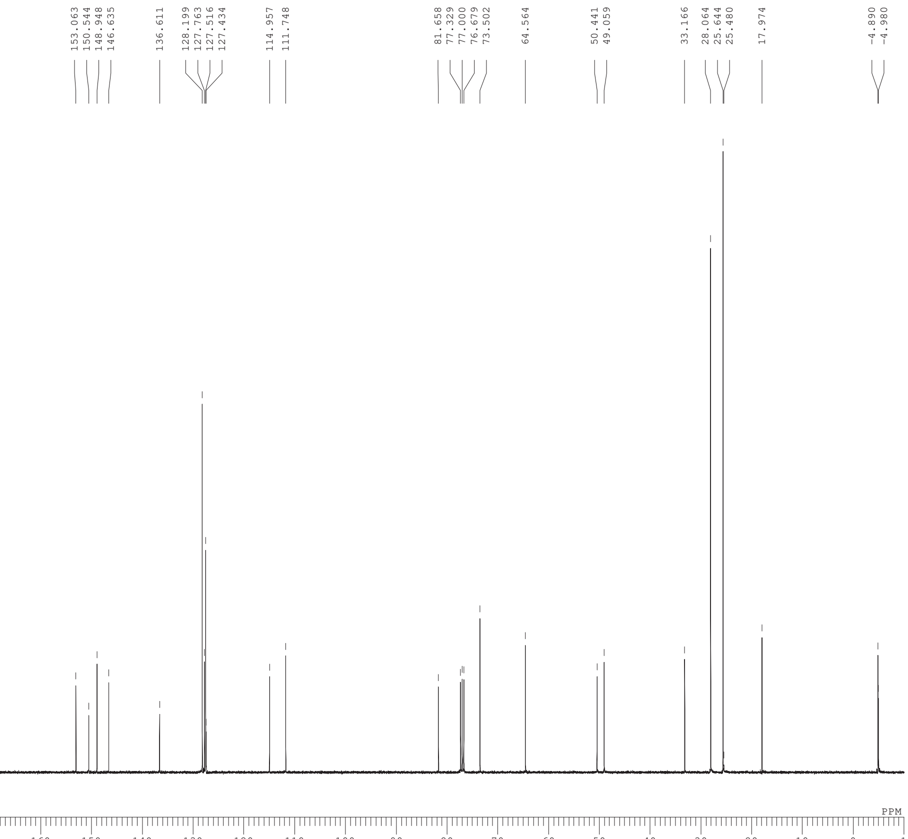

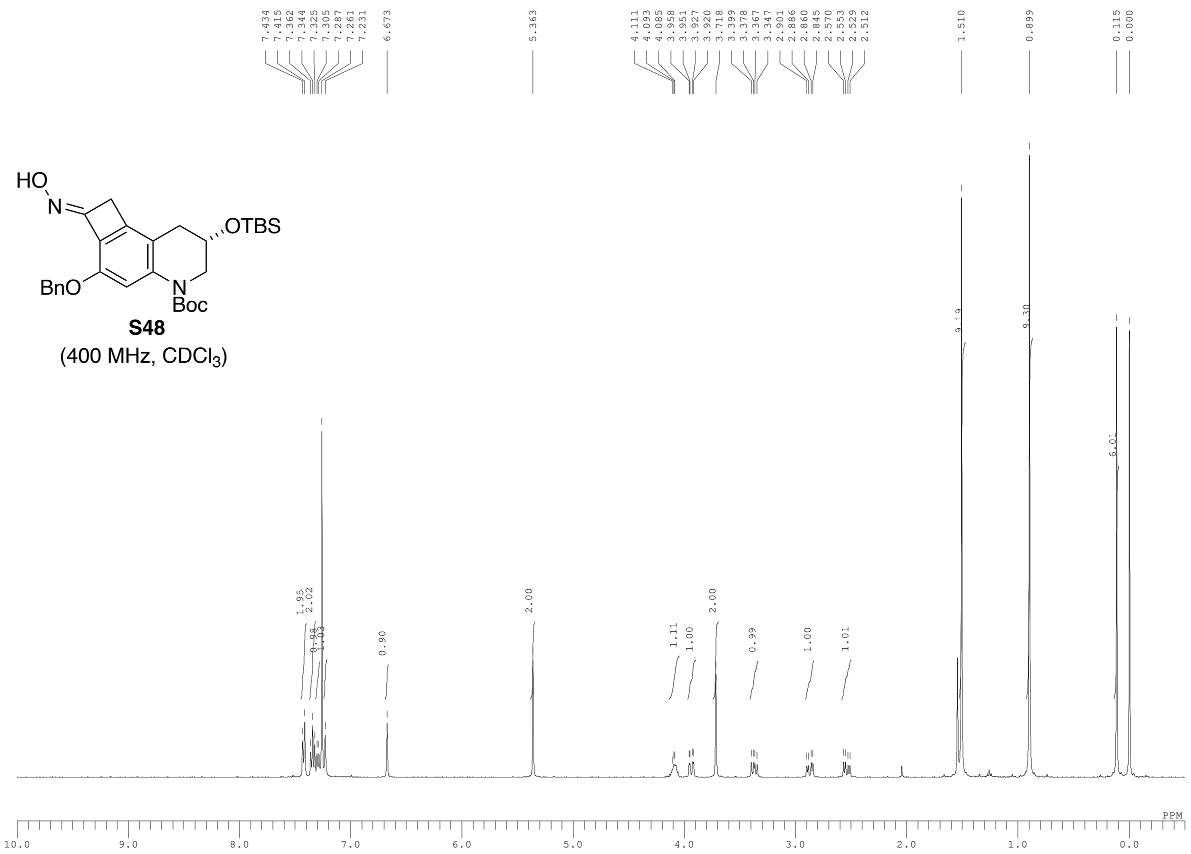

S48
(400 MHz, $CDCl_3$)

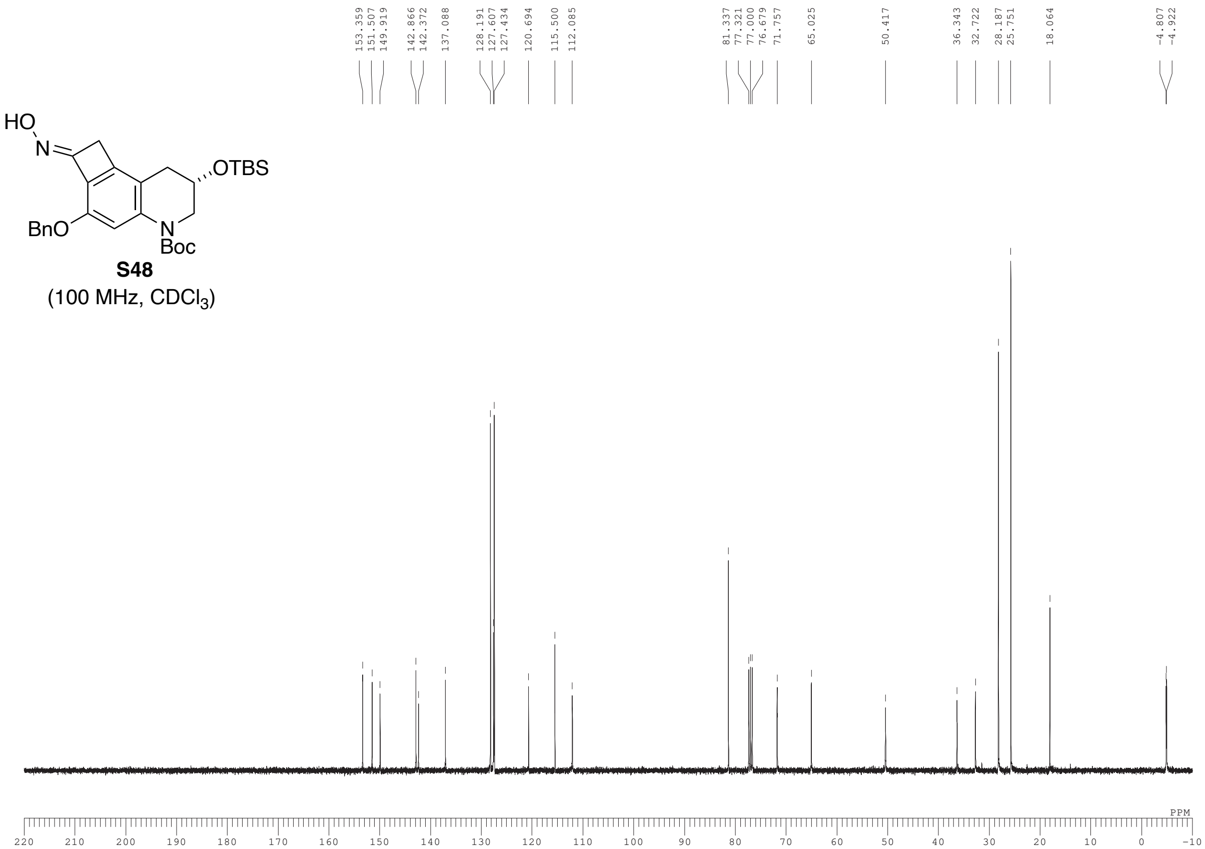

S48
(100 MHz, $CDCl_3$)

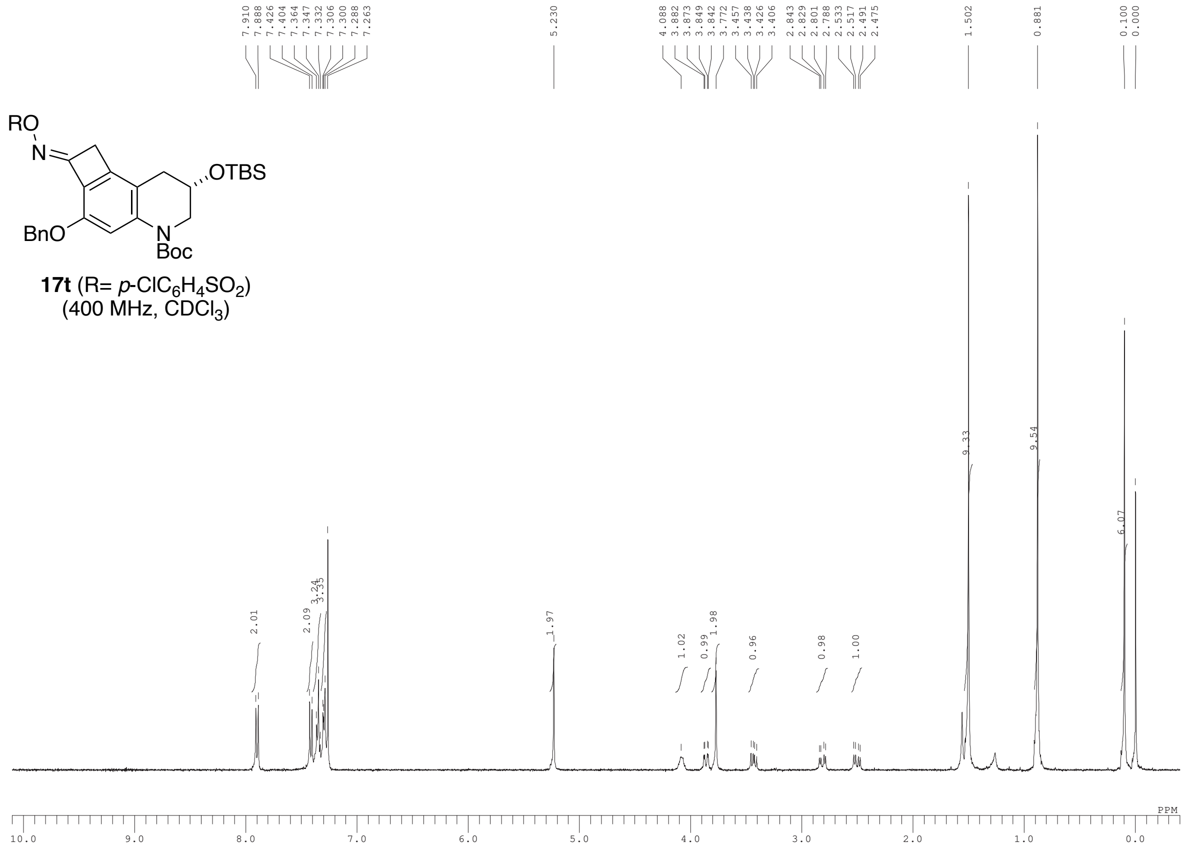

17t (R= *p*-ClC$_6$H$_4$SO$_2$)
(400 MHz, CDCl$_3$)

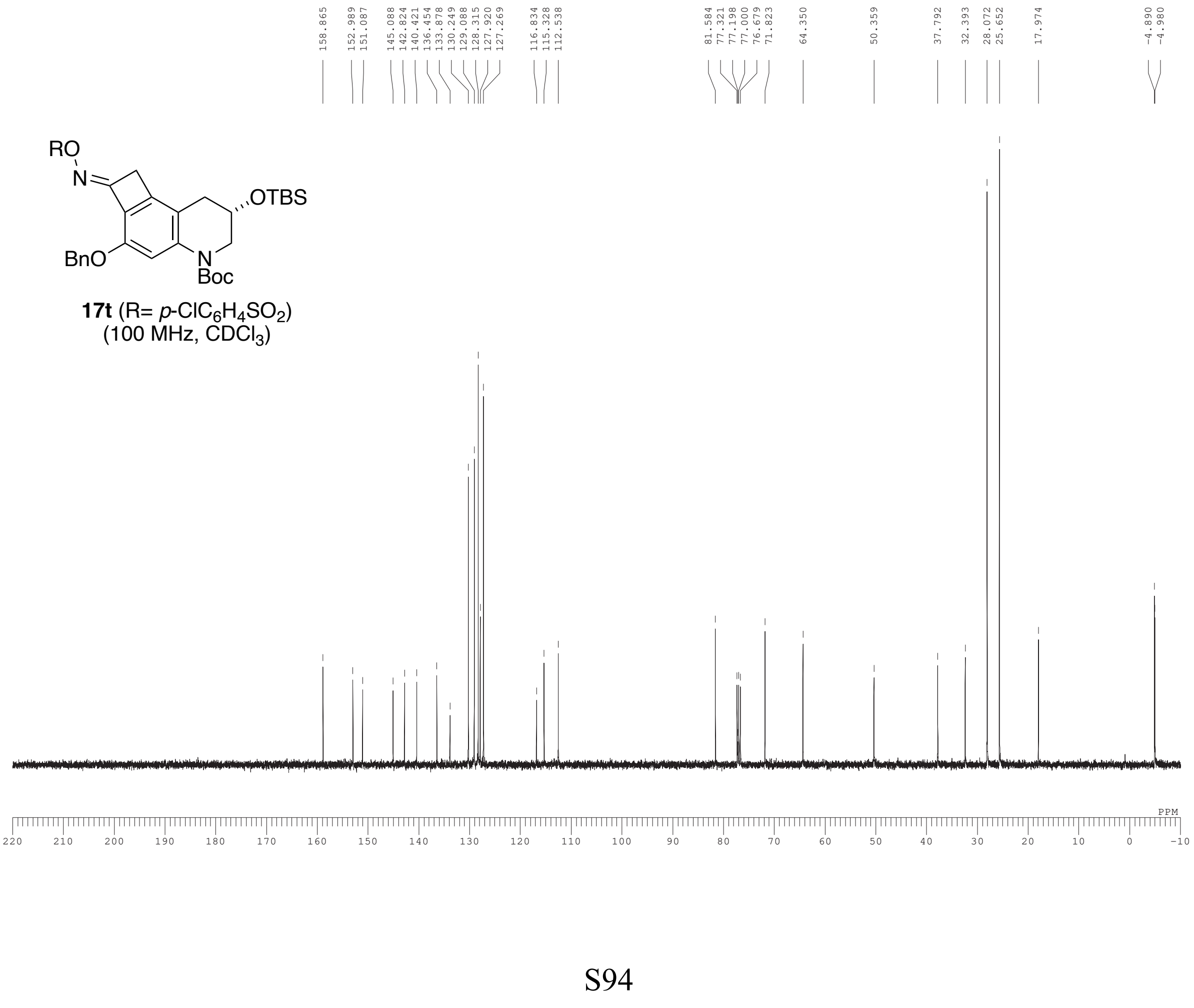

17t (R= *p*-ClC$_6$H$_4$SO$_2$)
(100 MHz, CDCl$_3$)

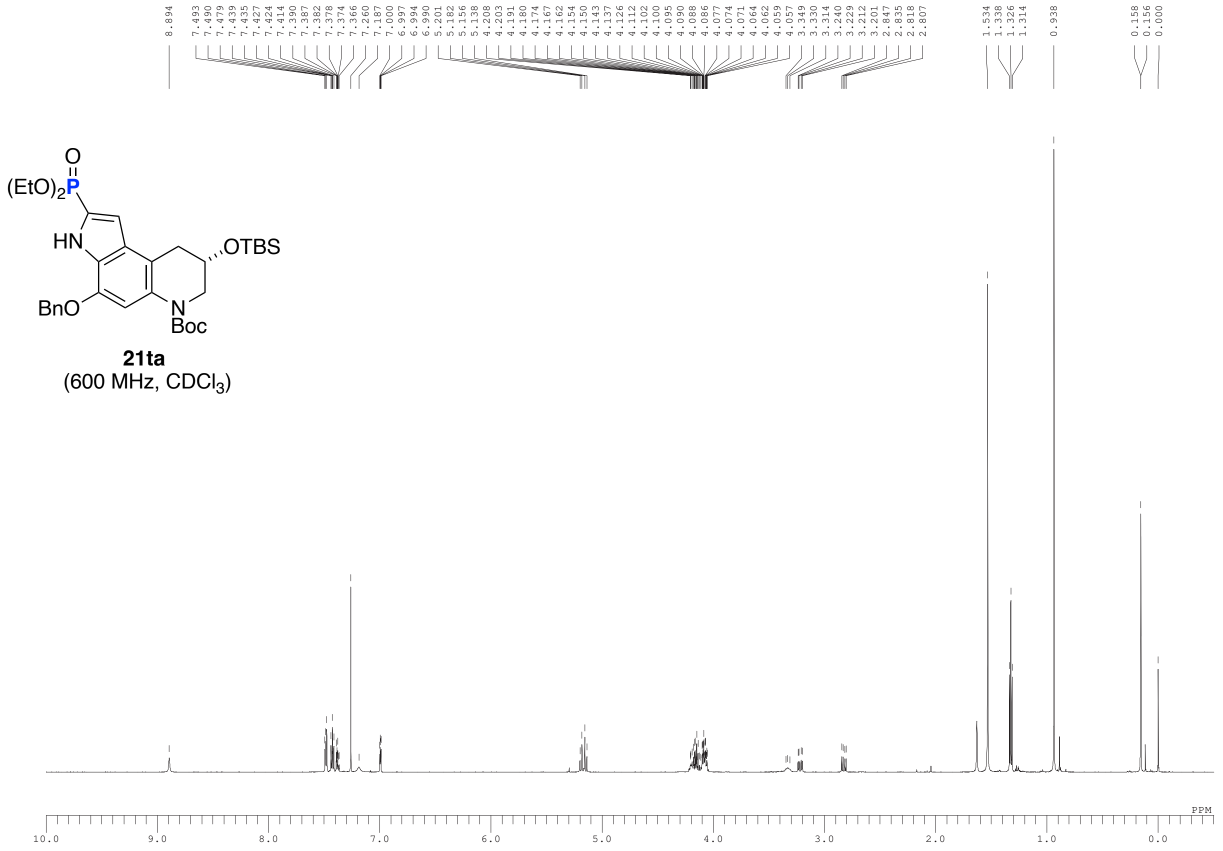

21ta
(600 MHz, $CDCl_3$)

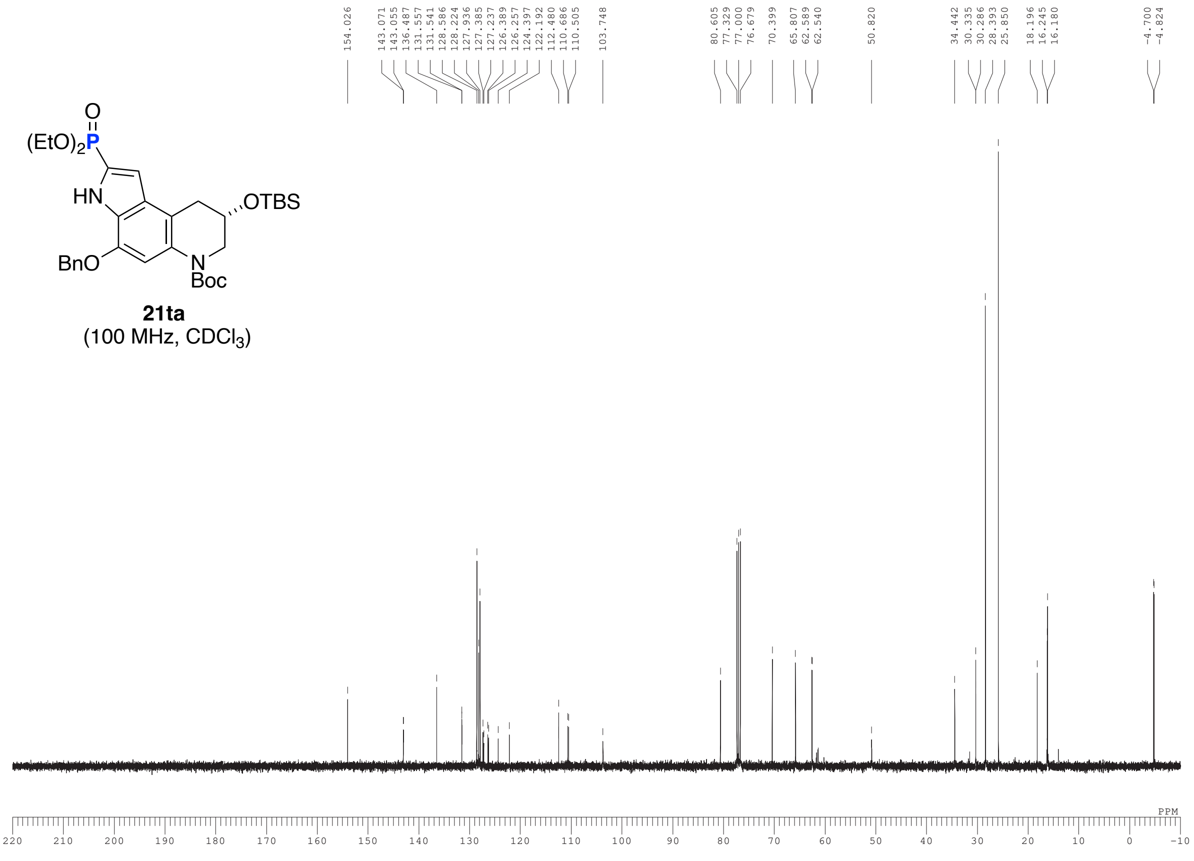

21ta
(100 MHz, $CDCl_3$)

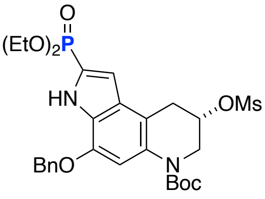

29
(400 MHz, $CDCl_3$)

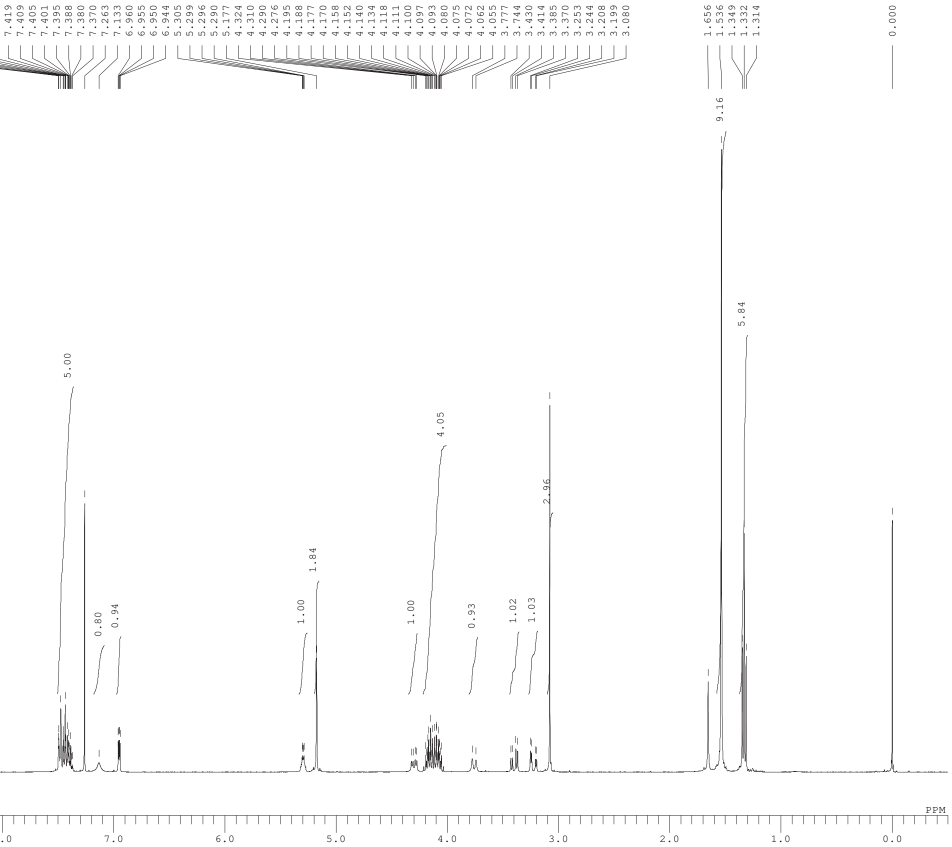

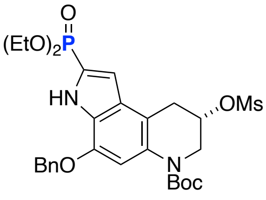

29
(150 MHz, $CDCl_3$)

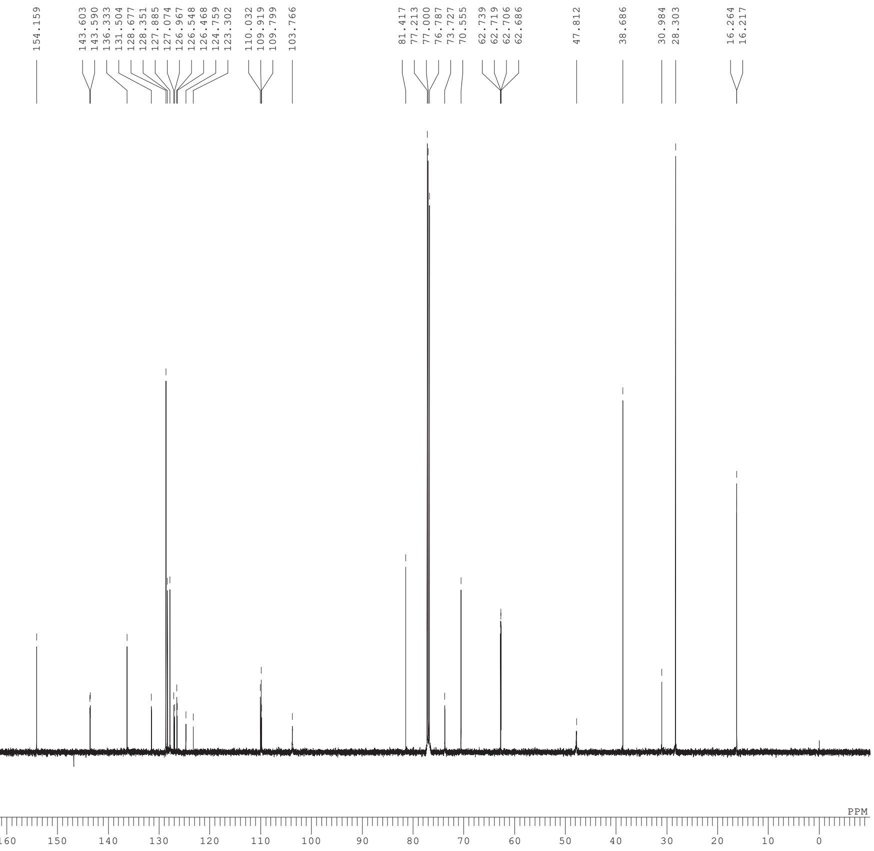

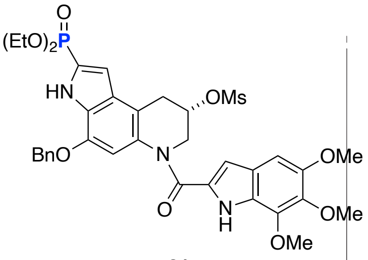

31
(600 MHz, $CDCl_3$)

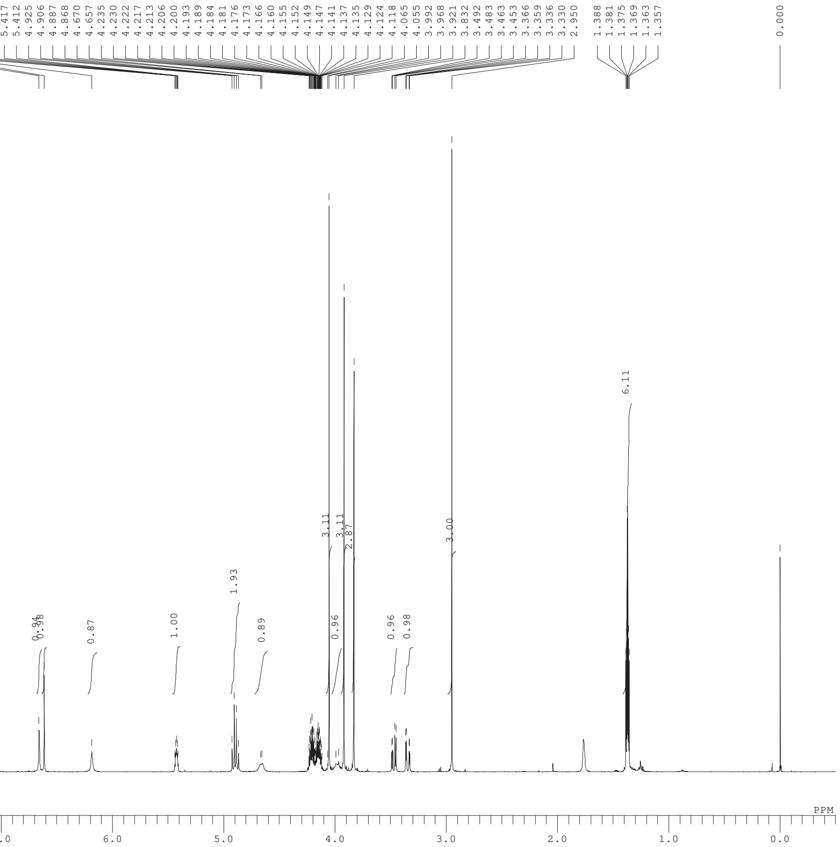

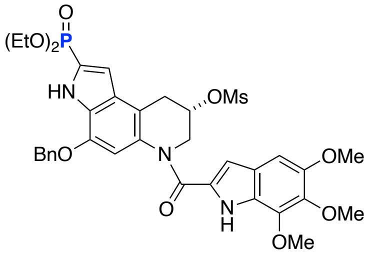

31
(150 MHz, $CDCl_3$)

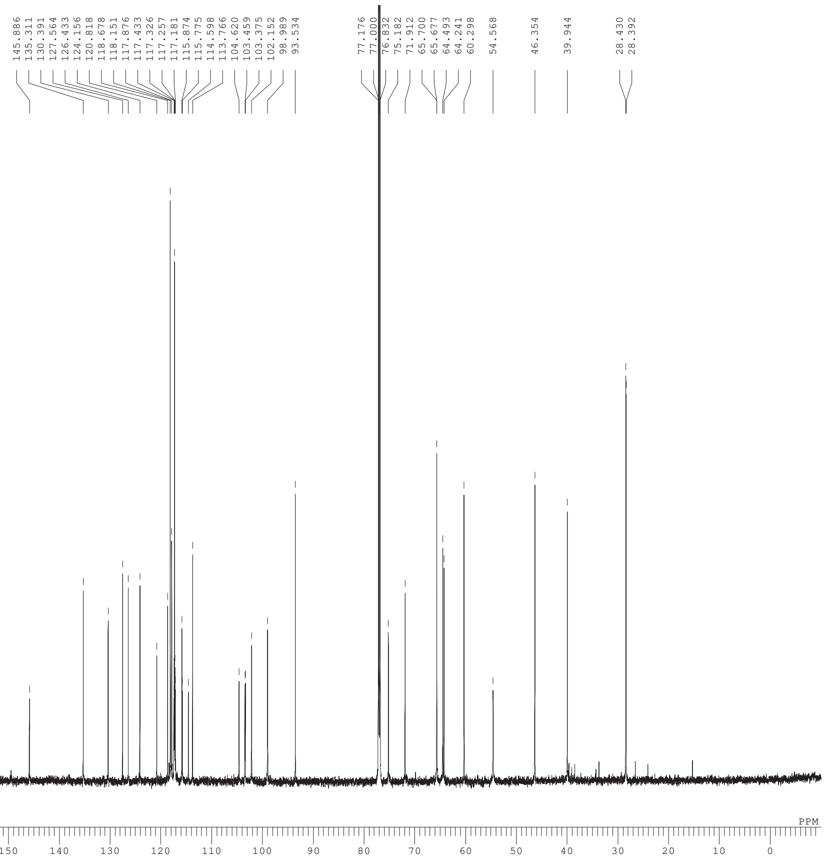

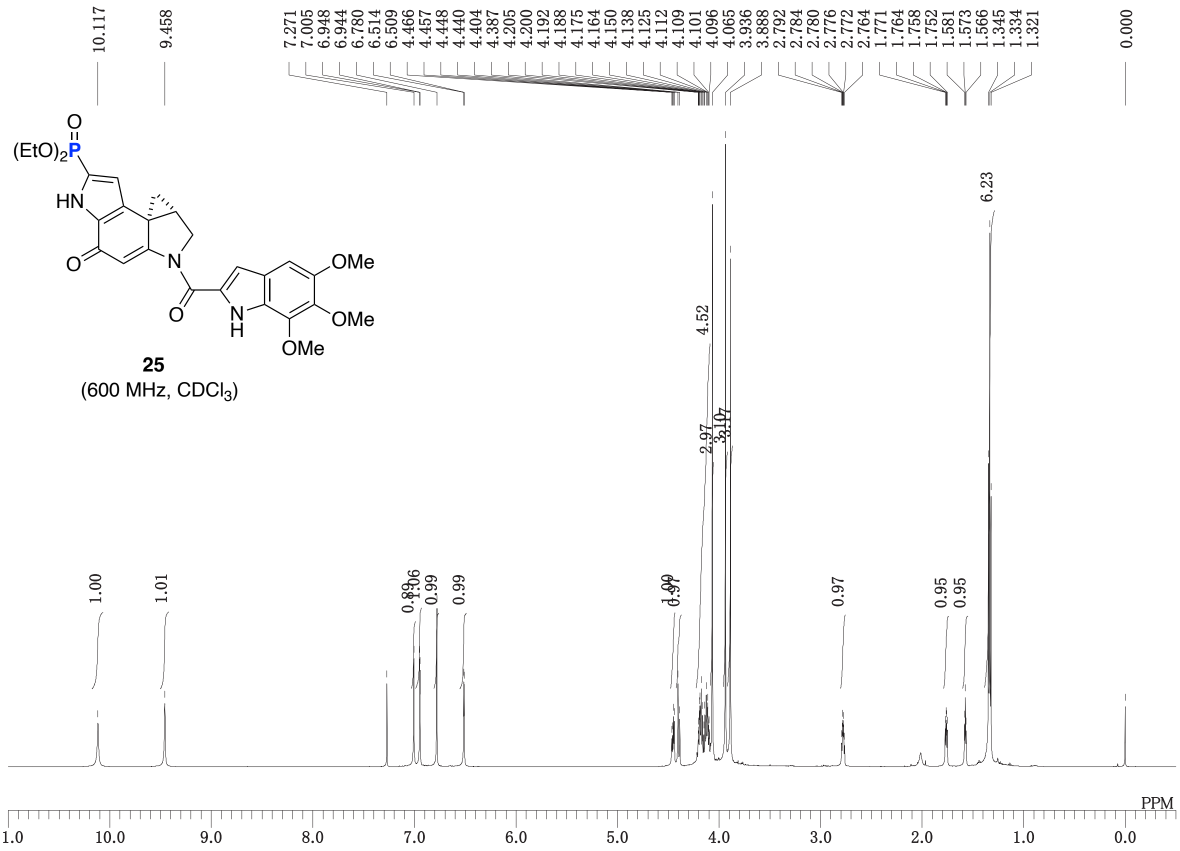

25
(600 MHz, $CDCl_3$)

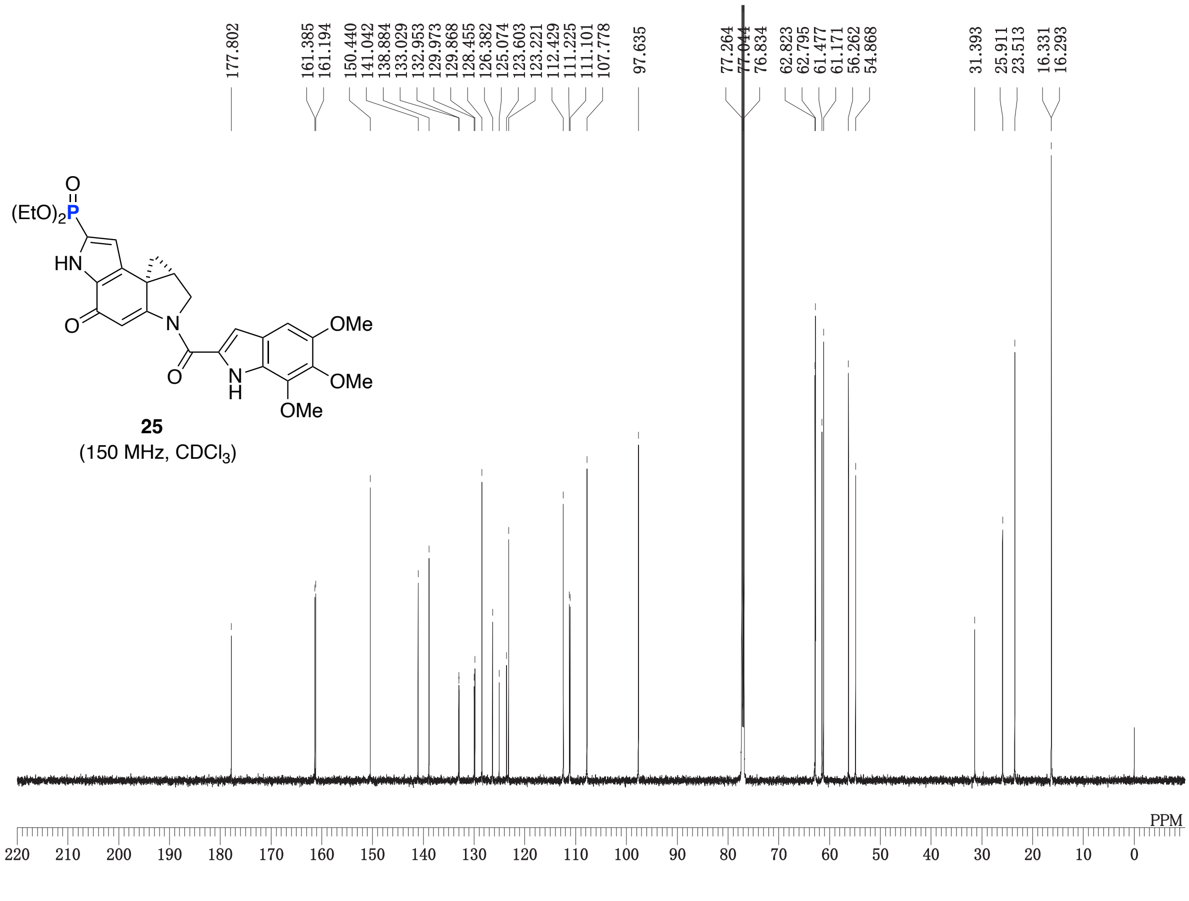

25
(150 MHz, $CDCl_3$)

6. ^{31}P NMR Spectra

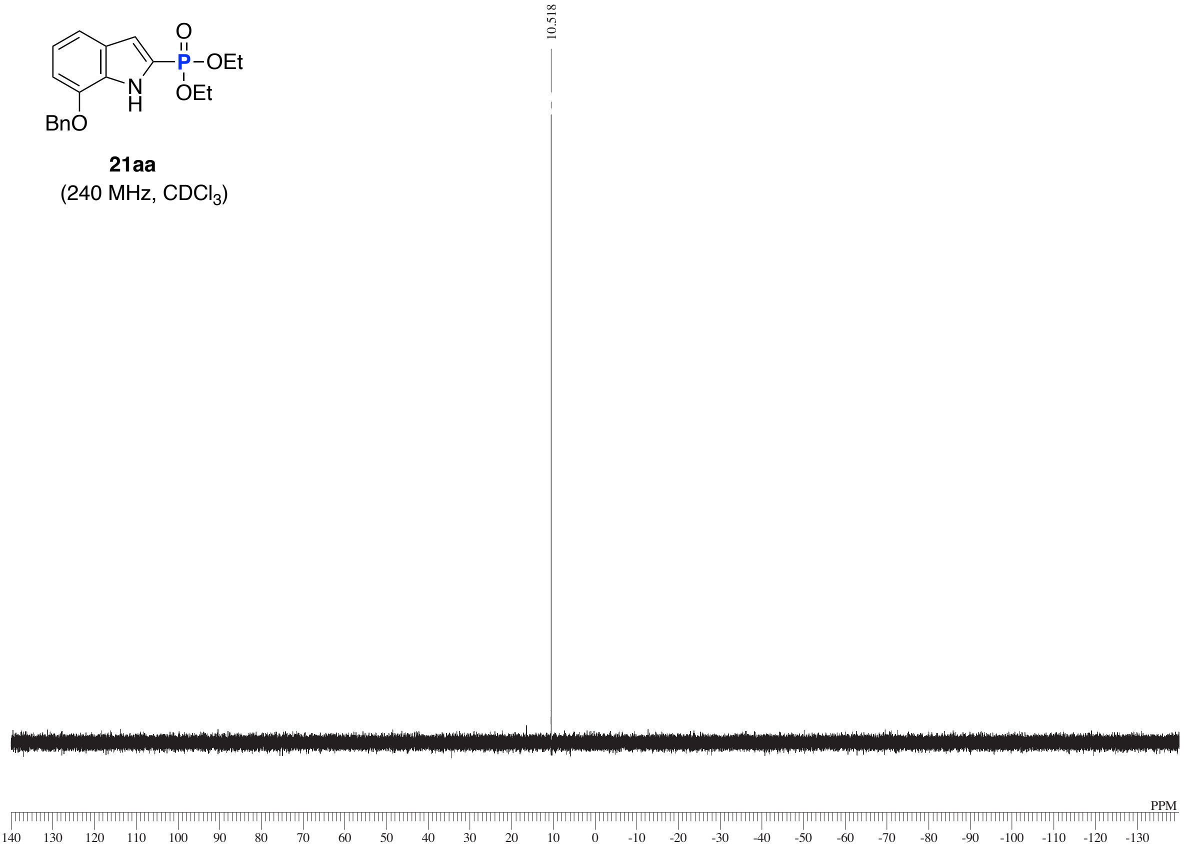

21aa
(240 MHz, $CDCl_3$)

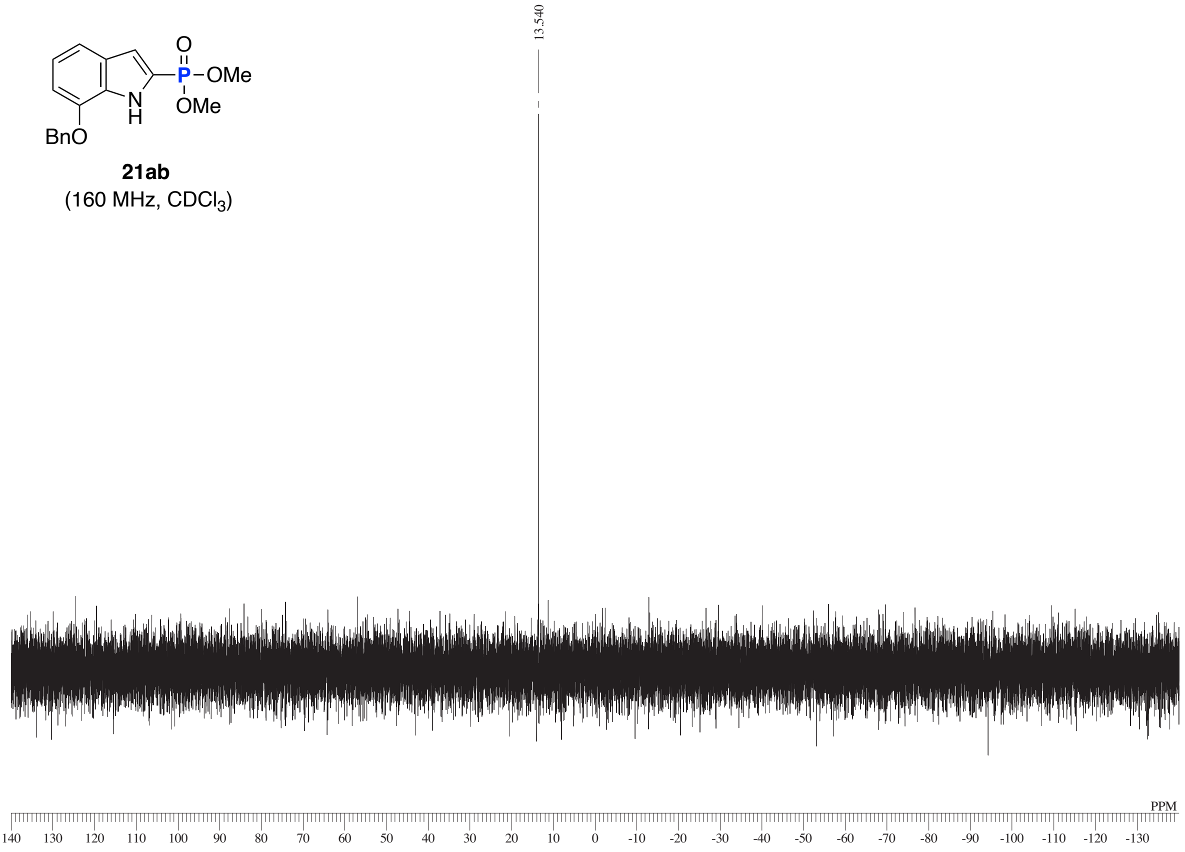

21ab
(160 MHz, $CDCl_3$)

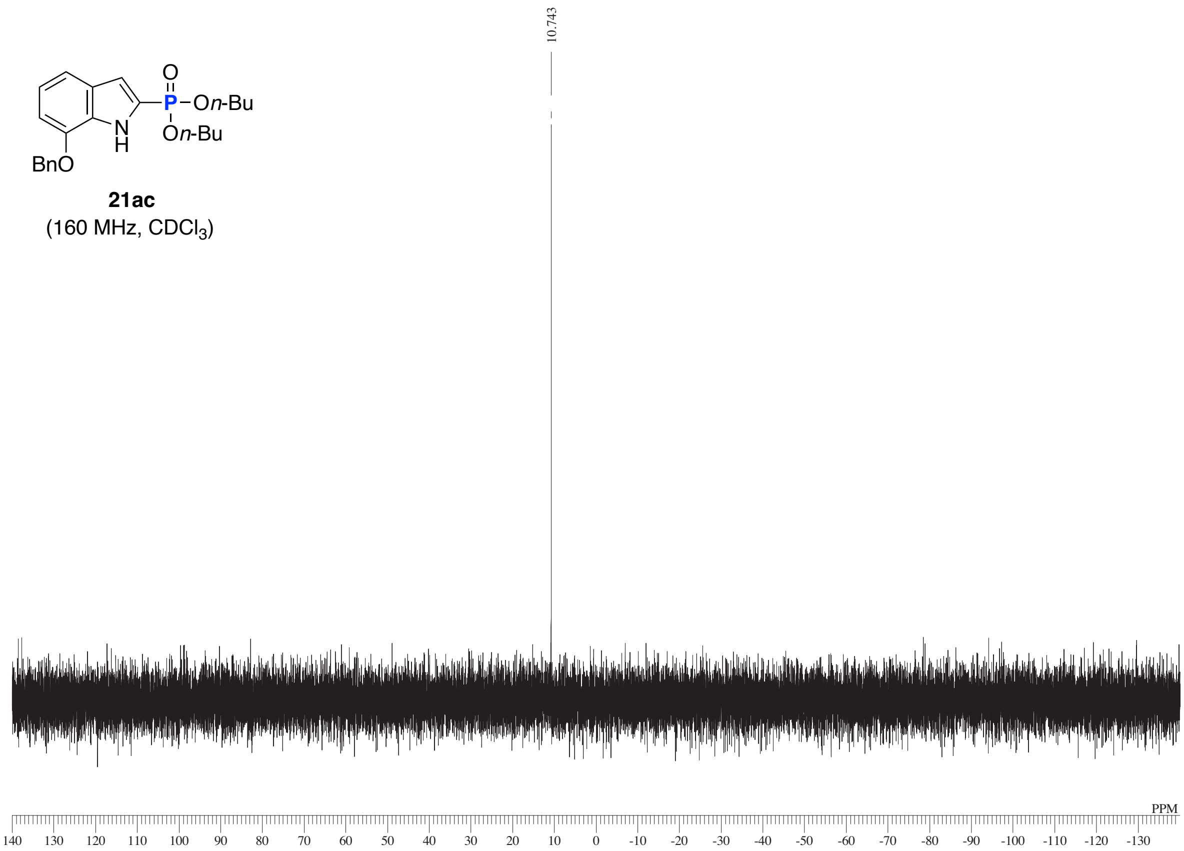
10.743
O
P
On-Bu
On-Bu
N
H
BnO
21ac
(160 MHz, CDCl3)
PPM
140 130 120 110 100 90 80 70 60 50 40 30 20 10 0 -10 -20 -30 -40 -50 -60 -70 -80 -90 -100 -110 -120 -130

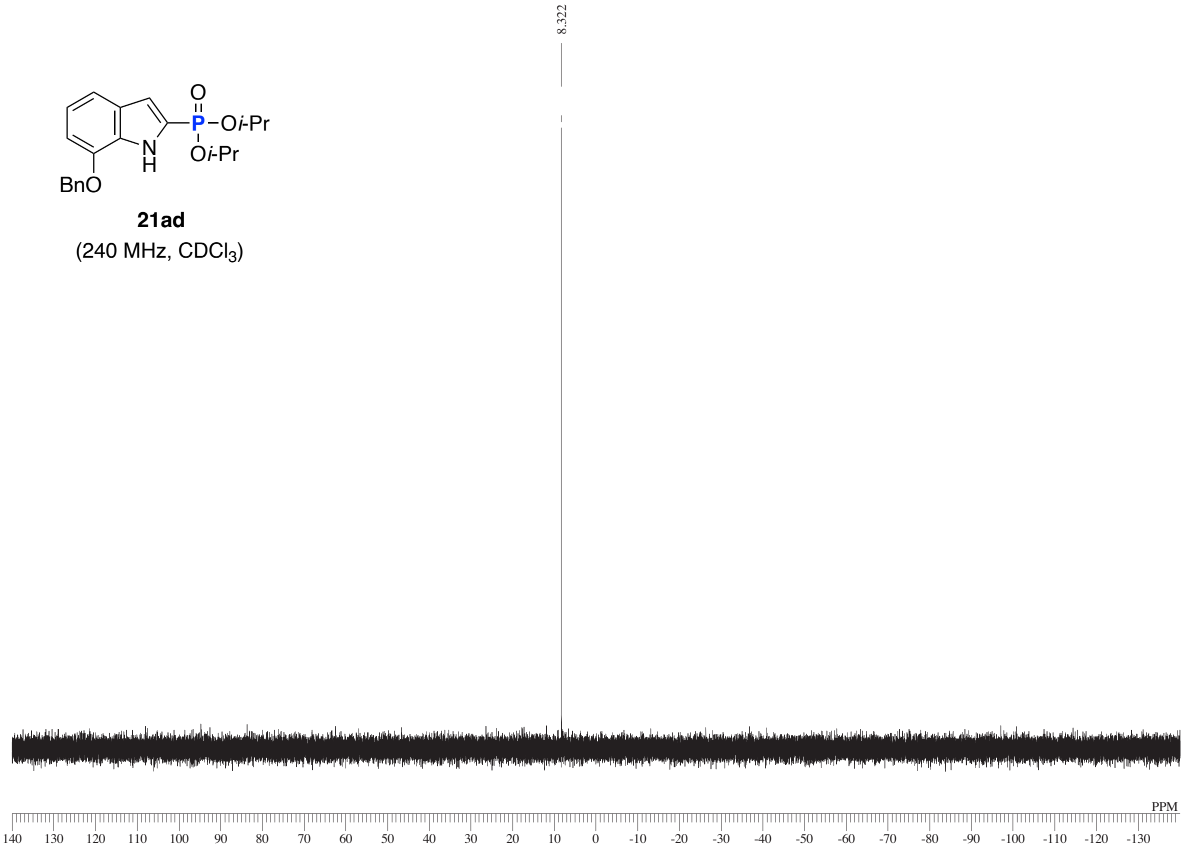
8.322
O
P
Oi-Pr
Oi-Pr
N
H
BnO
21ad
(240 MHz, CDCl3)
PPM
140 130 120 110 100 90 80 70 60 50 40 30 20 10 0 -10 -20 -30 -40 -50 -60 -70 -80 -90 -100 -110 -120 -130

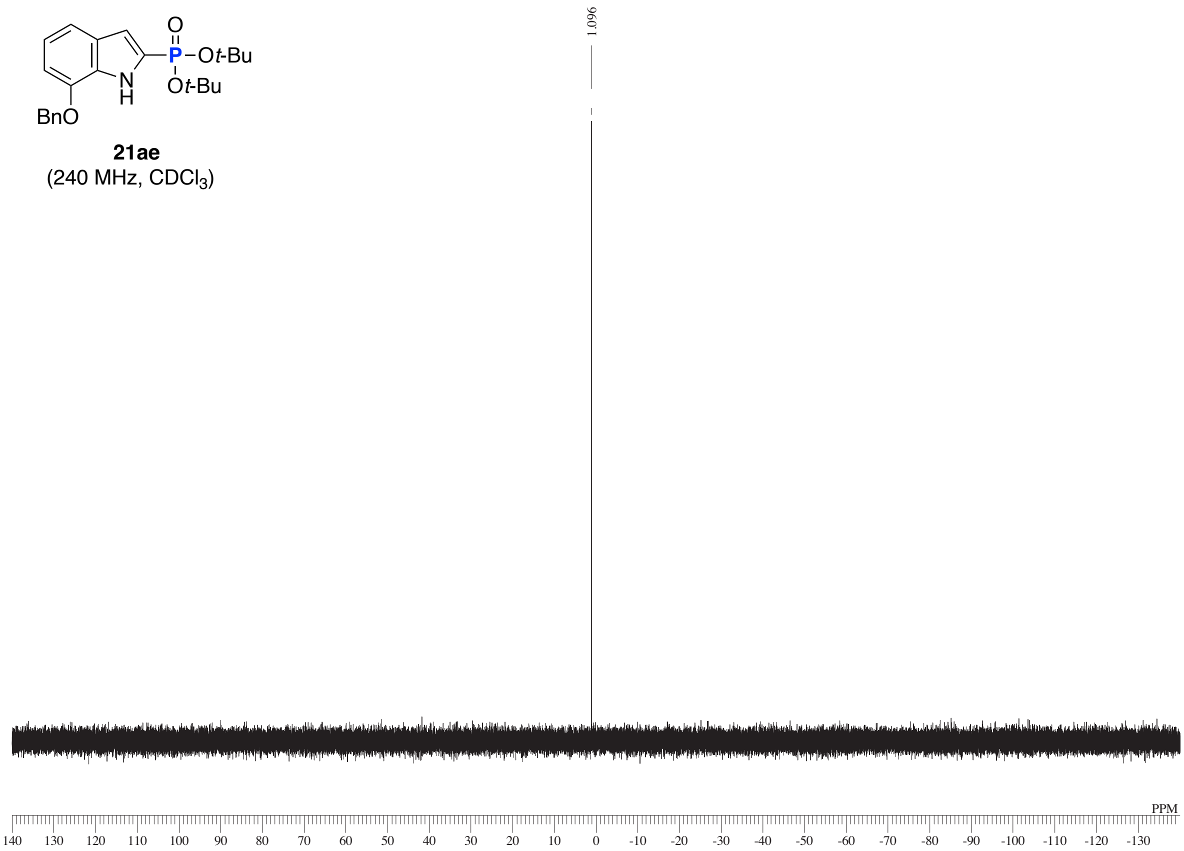

O
P
Ot-Bu
Ot-Bu
N
H
BnO
21ae
(240 MHz, CDCl3)
1.096
PPM
140 130 120 110 100 90 80 70 60 50 40 30 20 10 0 -10 -20 -30 -40 -50 -60 -70 -80 -90 -100 -110 -120 -130

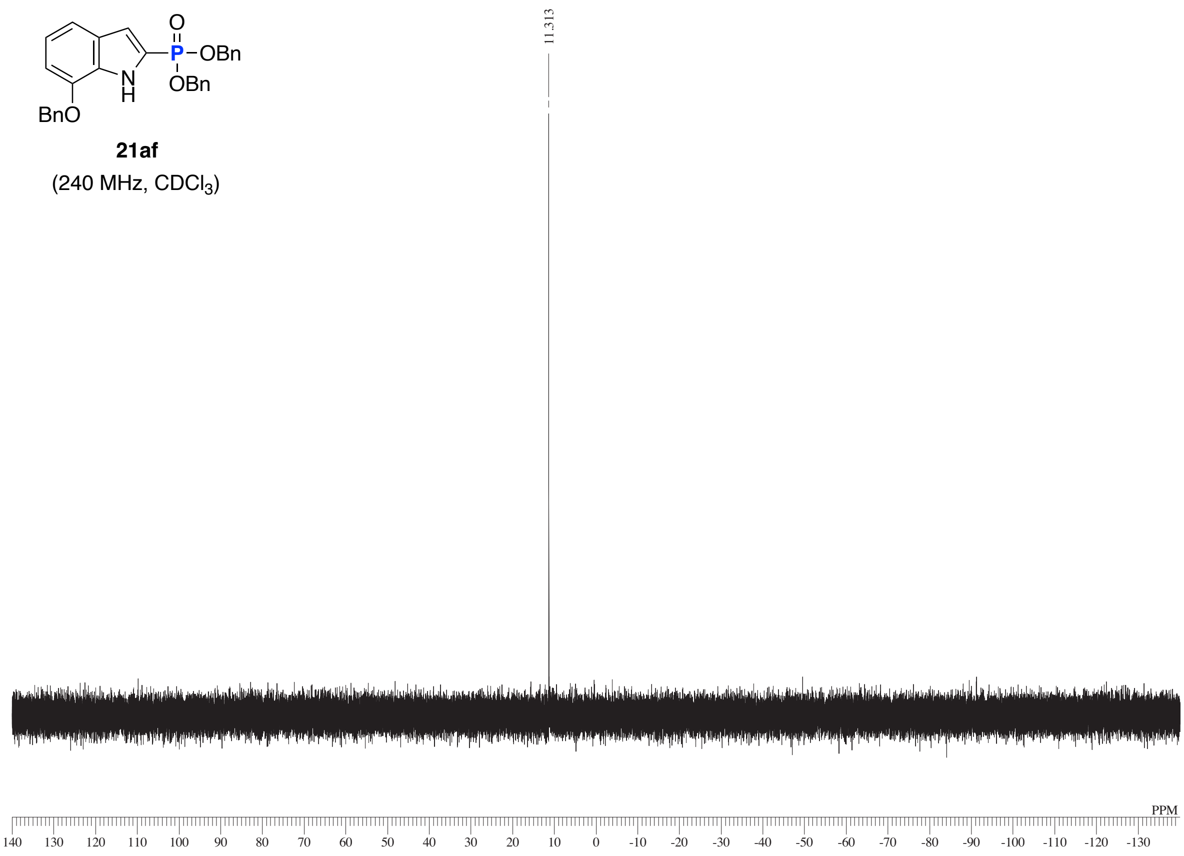

O
P
OBn
OBn
N
H
BnO
21af
(240 MHz, CDCl3)
11.313
PPM
140 130 120 110 100 90 80 70 60 50 40 30 20 10 0 -10 -20 -30 -40 -50 -60 -70 -80 -90 -100 -110 -120 -130

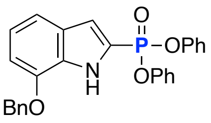

21ag
(240 MHz, $CDCl_3$)

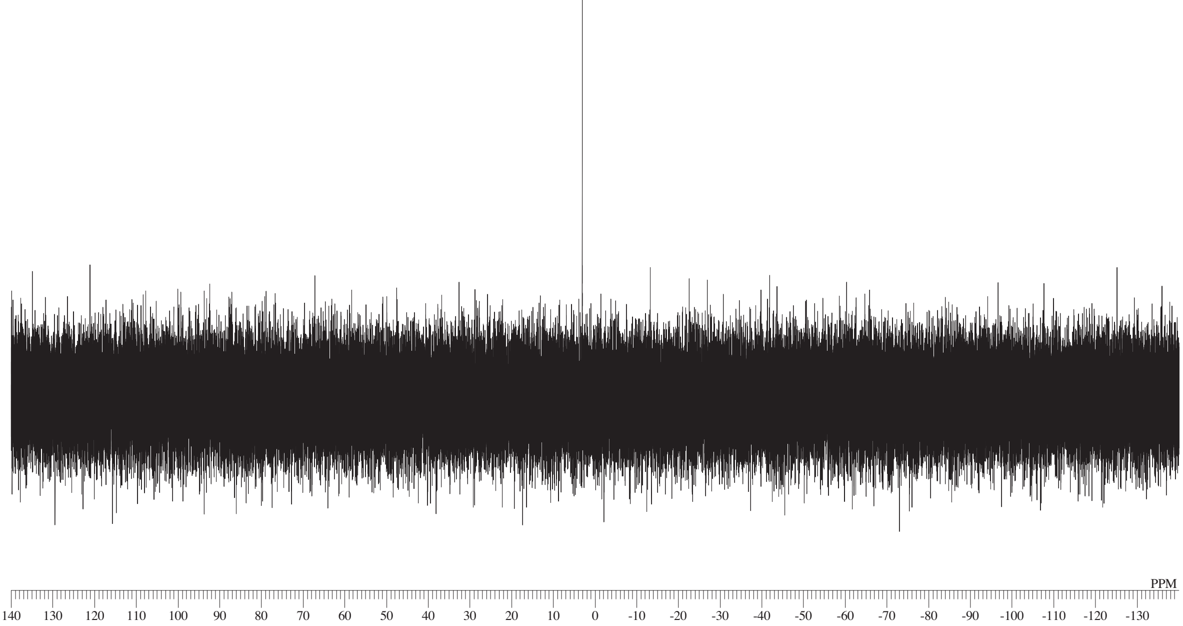

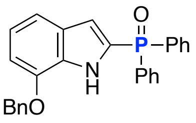

21ah
(160 MHz, $CDCl_3$)

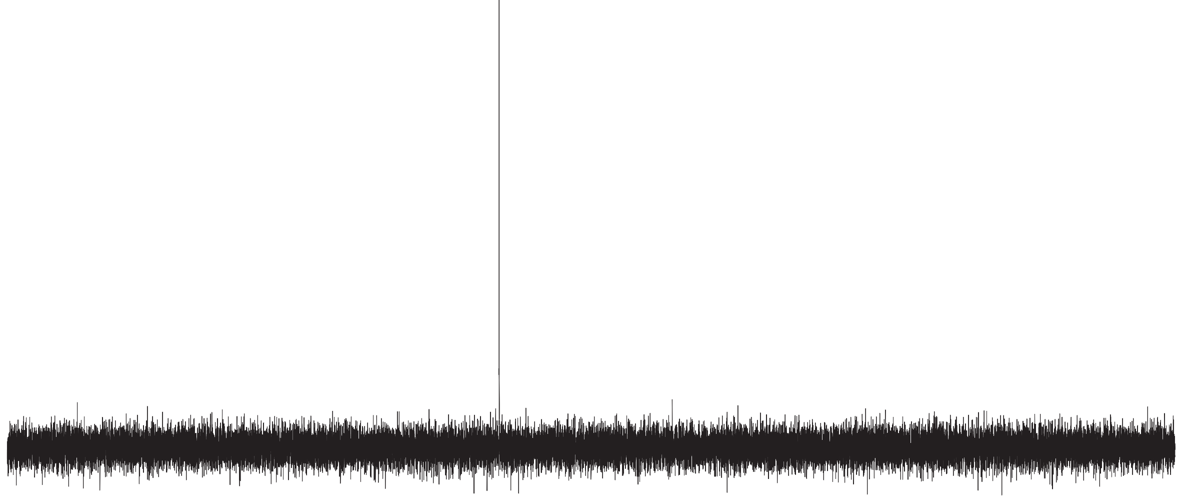

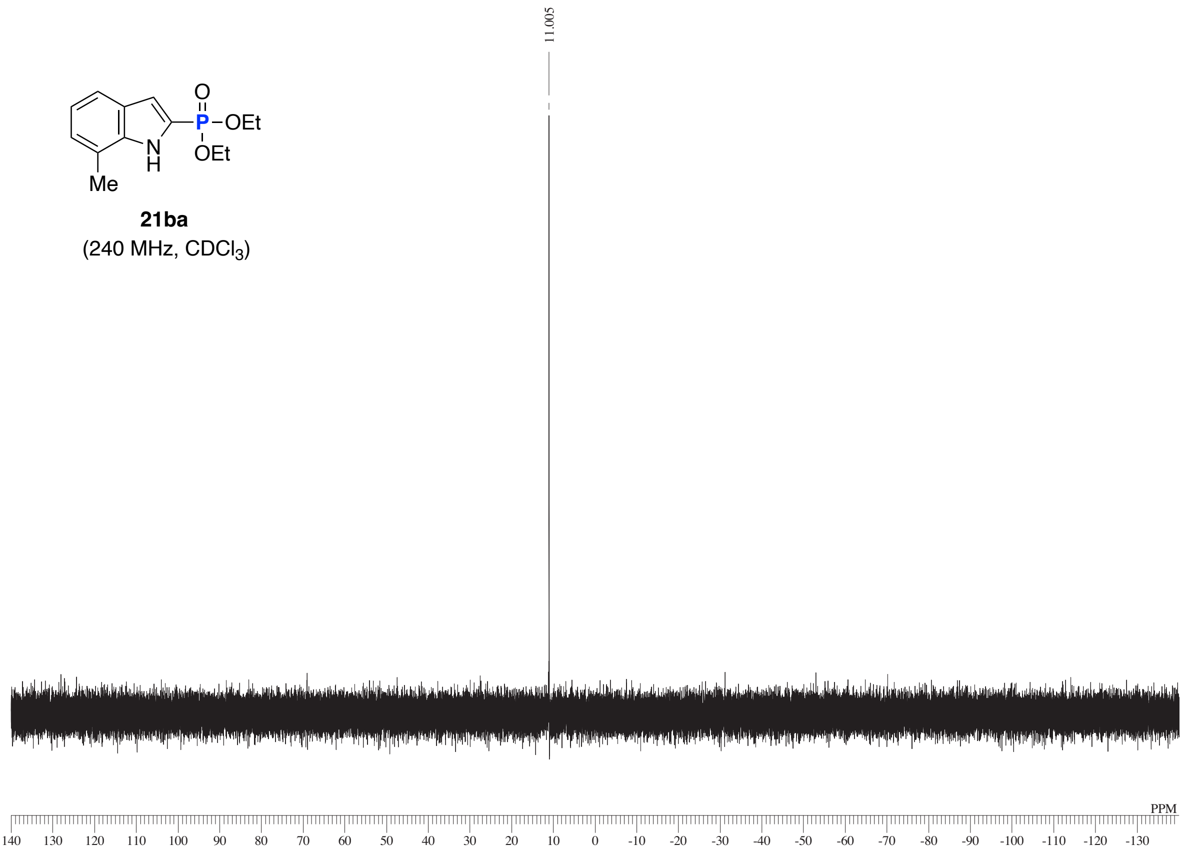

21ba
(240 MHz, $CDCl_3$)

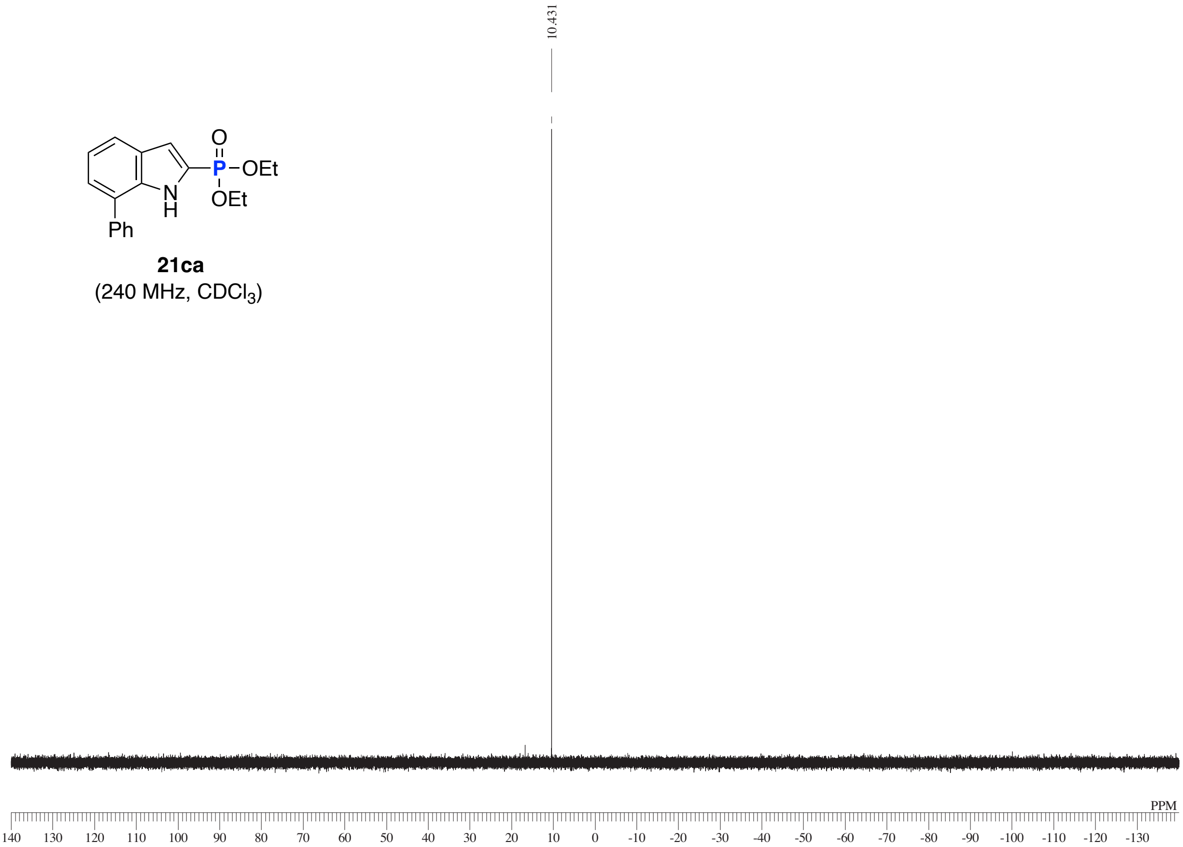

21ca
(240 MHz, $CDCl_3$)

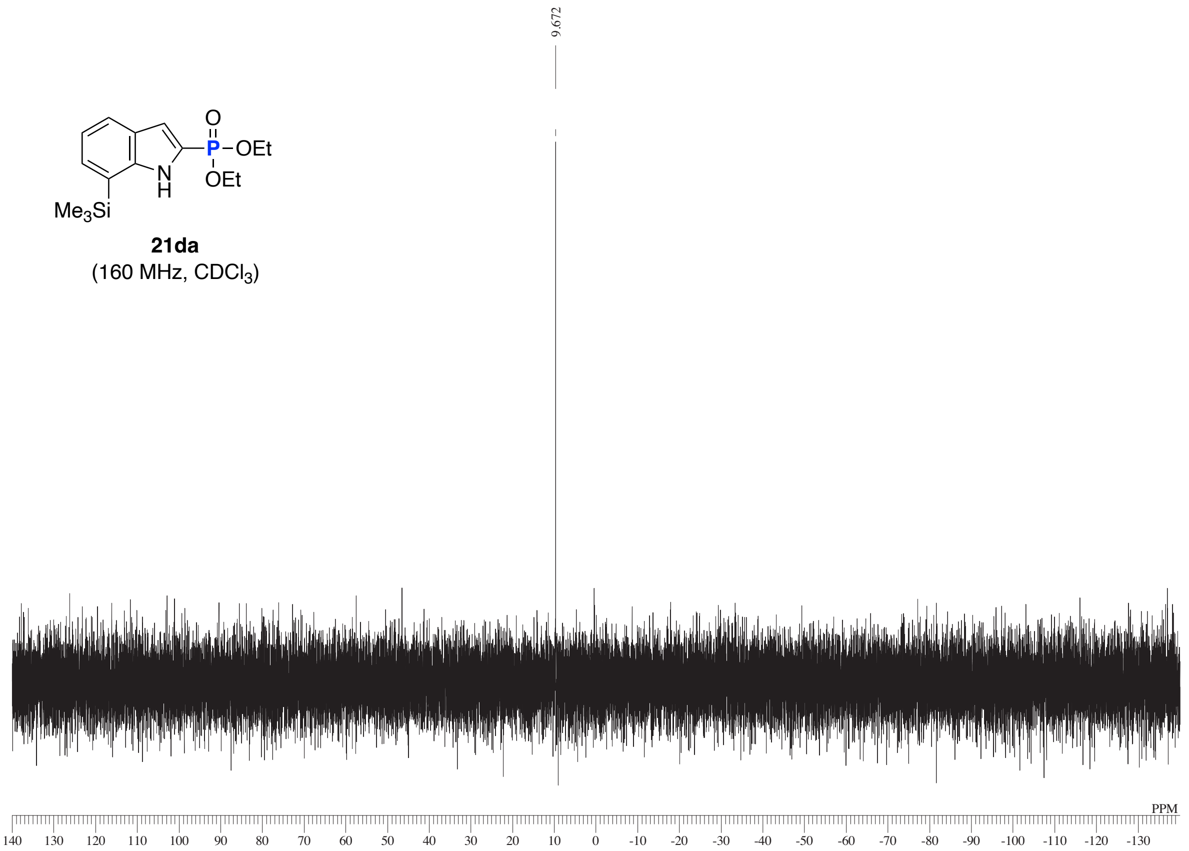
9.672
O
P
OEt
OEt
N
H
Me_3Si
21da
(160 MHz, $CDCl_3$)
PPM
140 130 120 110 100 90 80 70 60 50 40 30 20 10 0 -10 -20 -30 -40 -50 -60 -70 -80 -90 -100 -110 -120 -130

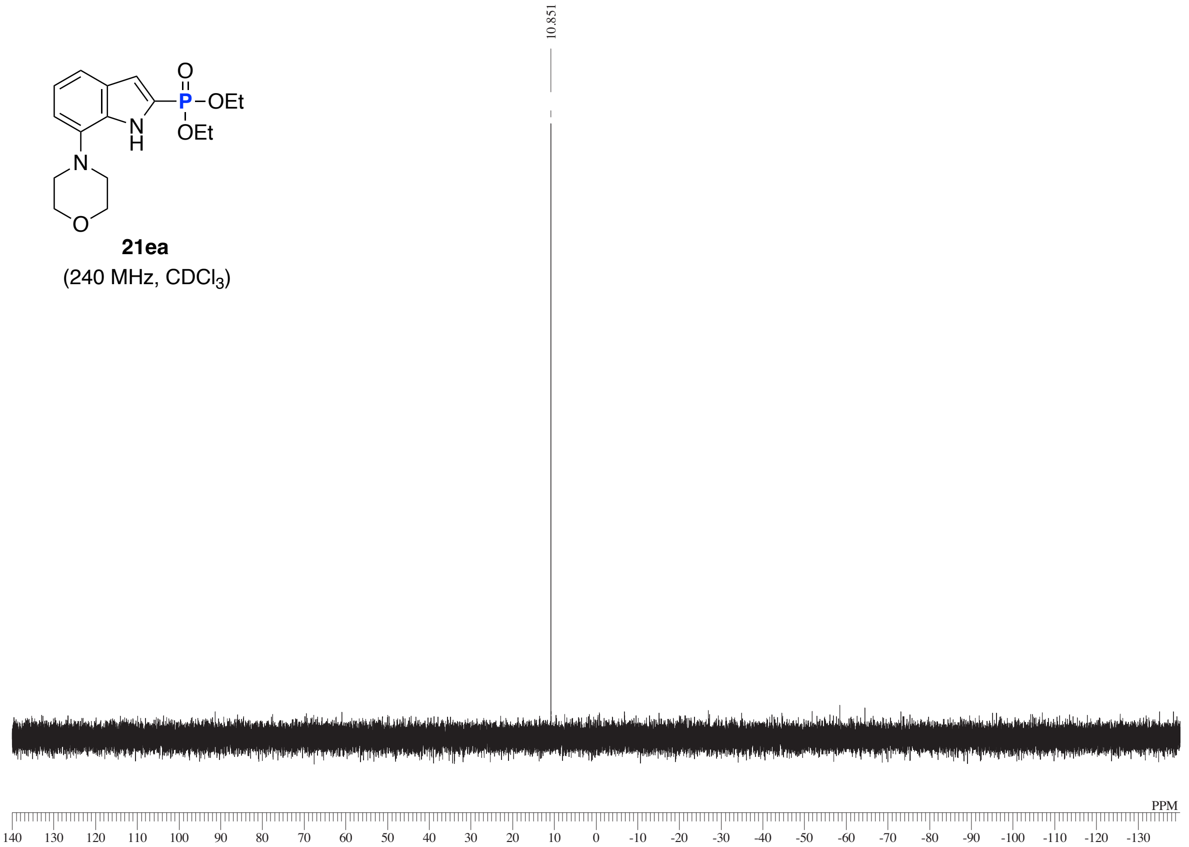
10.851
O
P
OEt
OEt
N
H
N
O
21ea
(240 MHz, $CDCl_3$)
PPM
140 130 120 110 100 90 80 70 60 50 40 30 20 10 0 -10 -20 -30 -40 -50 -60 -70 -80 -90 -100 -110 -120 -130

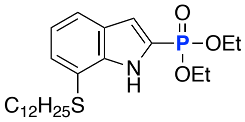

21fa

(240 MHz, $CDCl_3$)

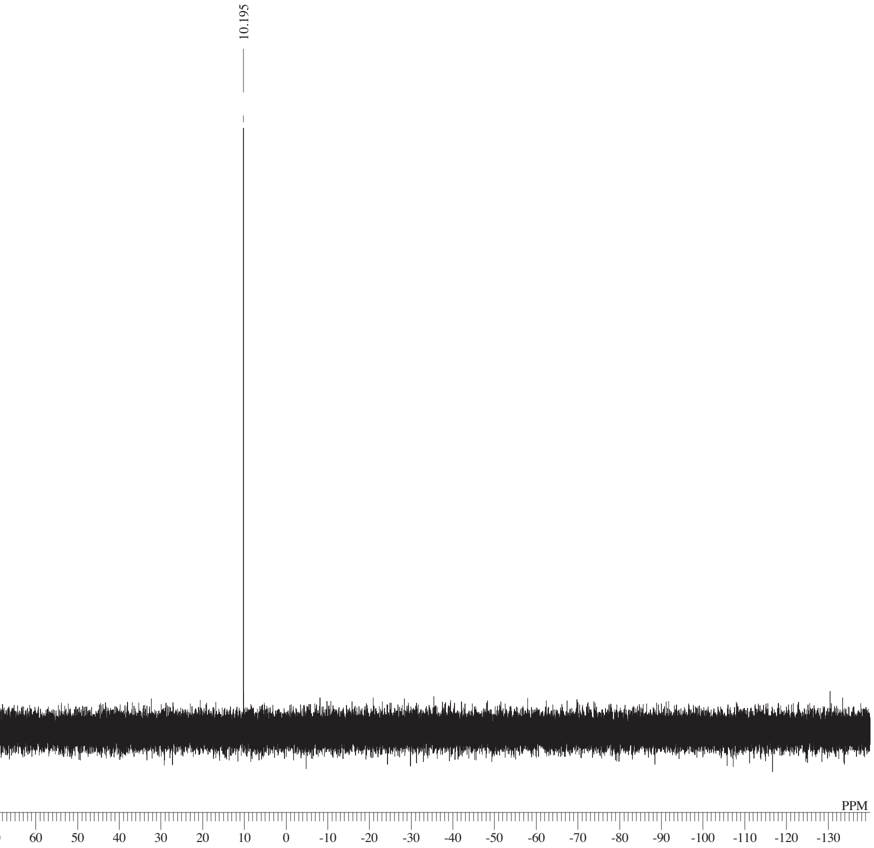

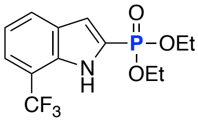

21ga

(160 MHz, $CDCl_3$)

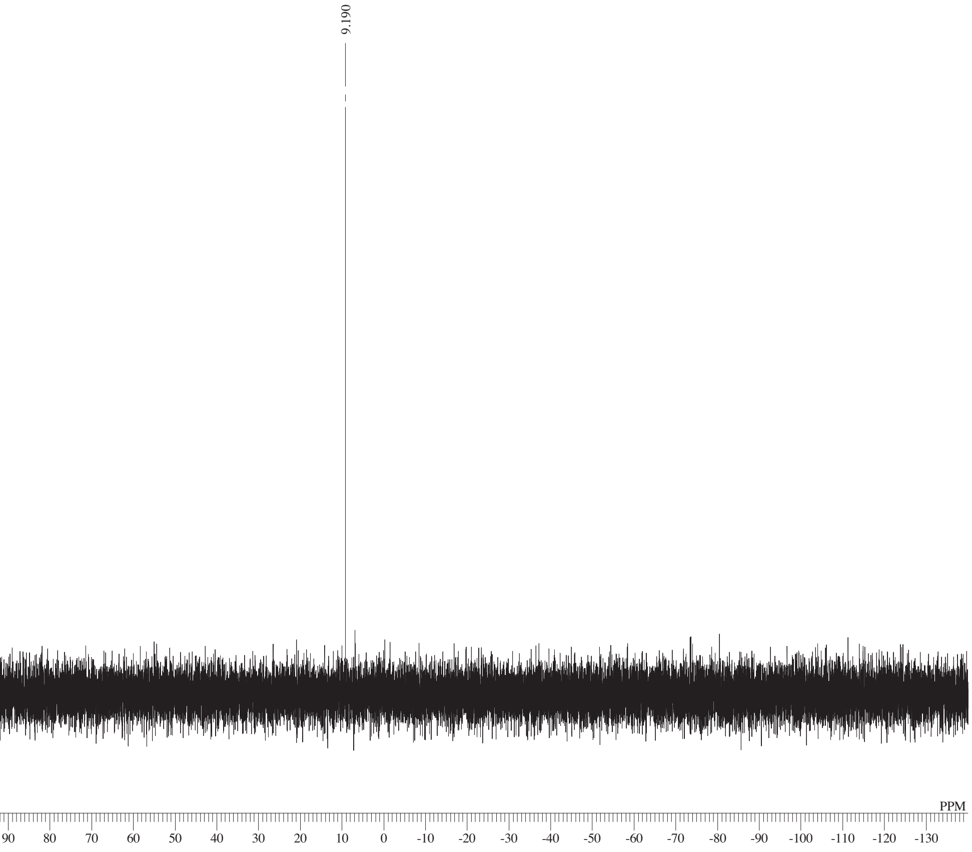

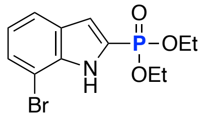

21ha
(160 MHz, $CDCl_3$)

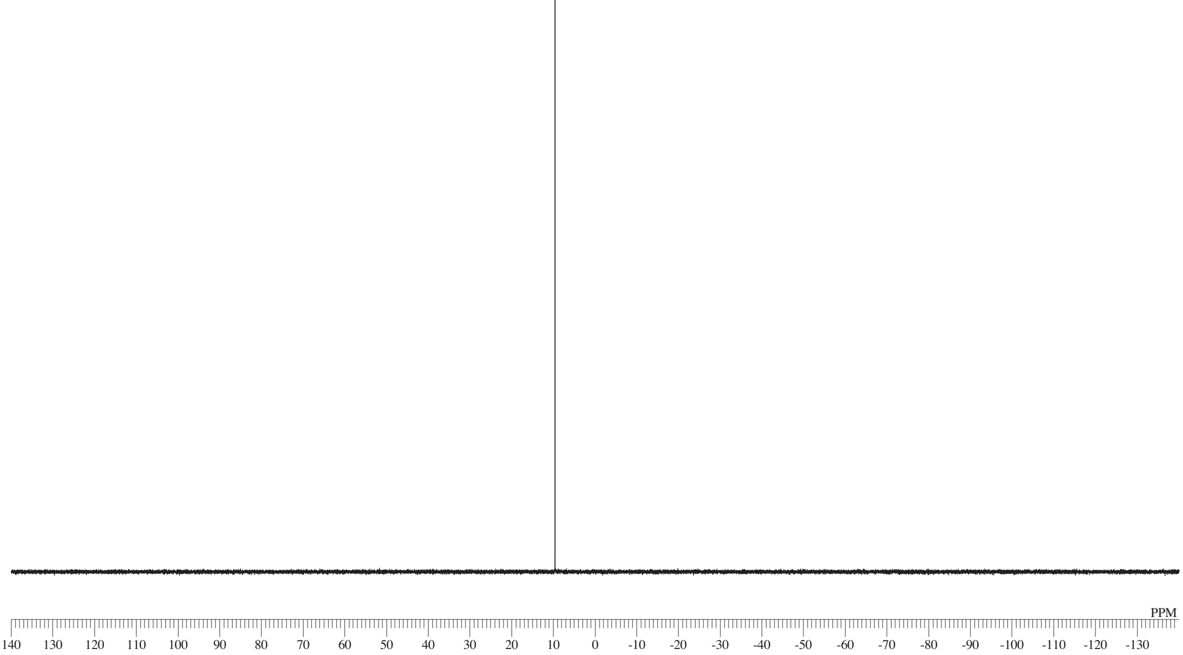

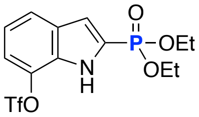

21ia
(240 MHz, $CDCl_3$)

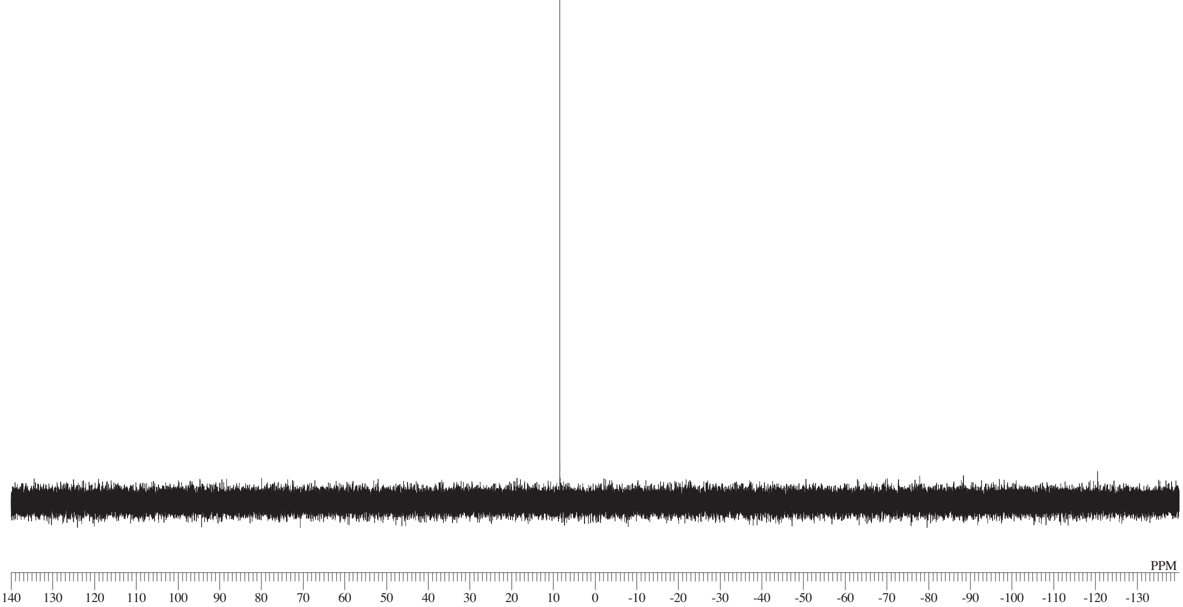

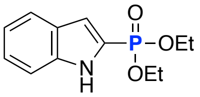

21ja
(160 MHz, $CDCl_3$)

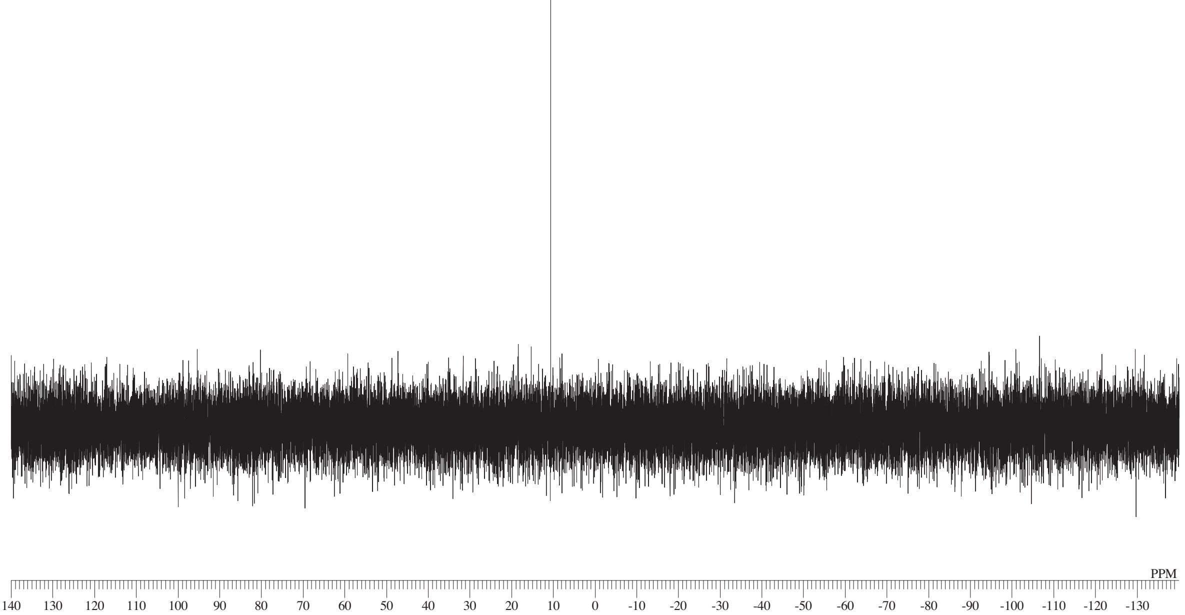

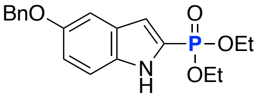

21ka
(240 MHz, $CDCl_3$)

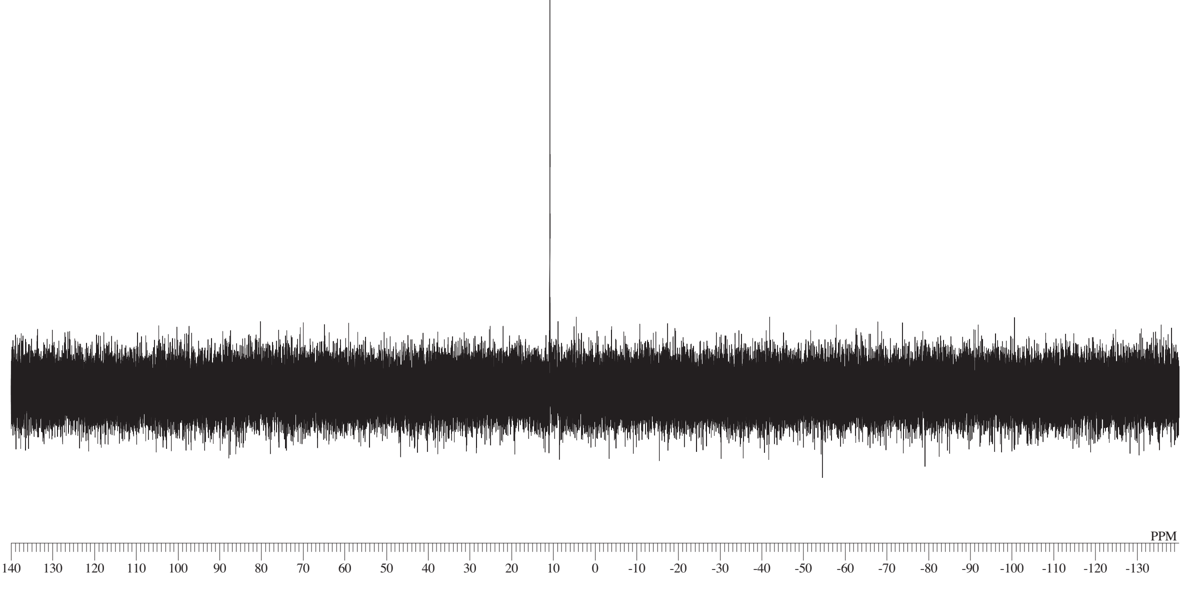

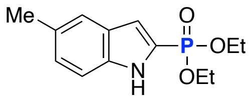

21la
(240 MHz, $CDCl_3$)

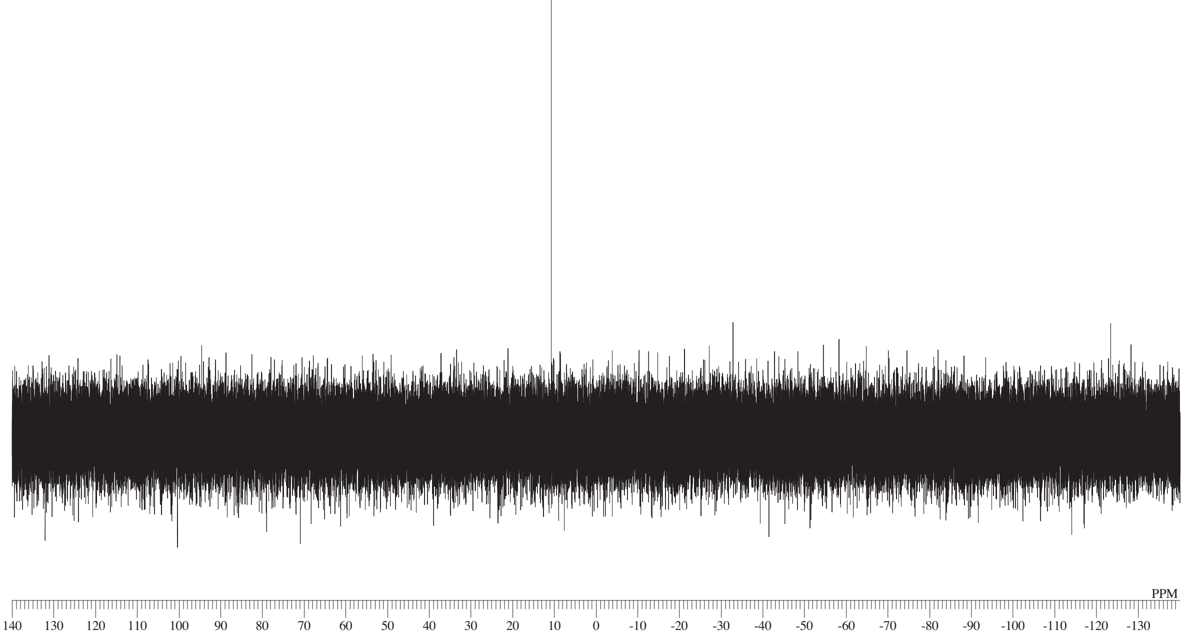

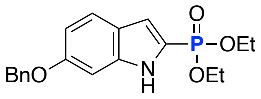

21ma
(160 MHz, $CDCl_3$)

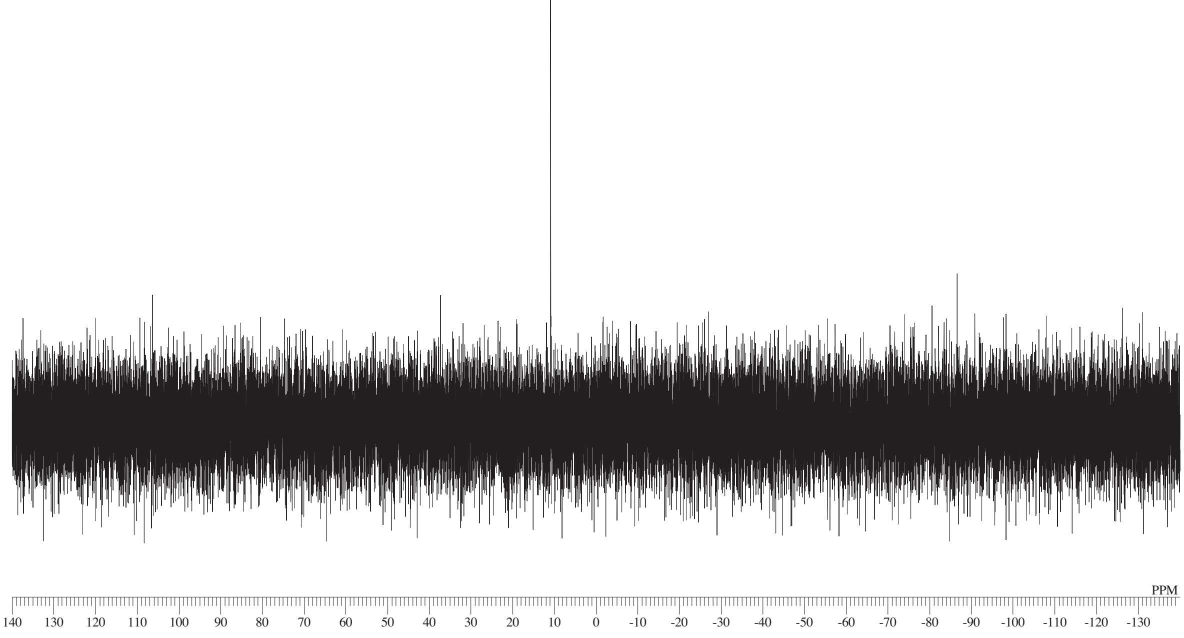

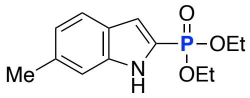

21na
(240 MHz, $CDCl_3$)

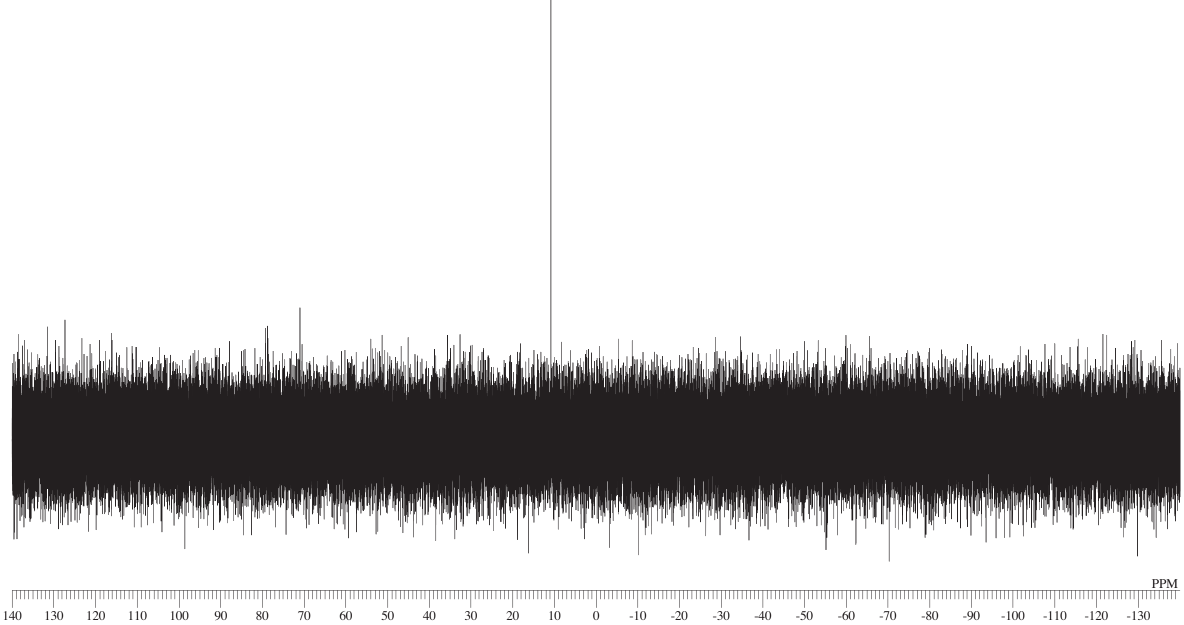

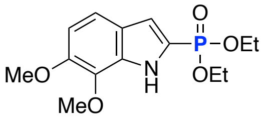

21oa
(240 MHz, $CDCl_3$)

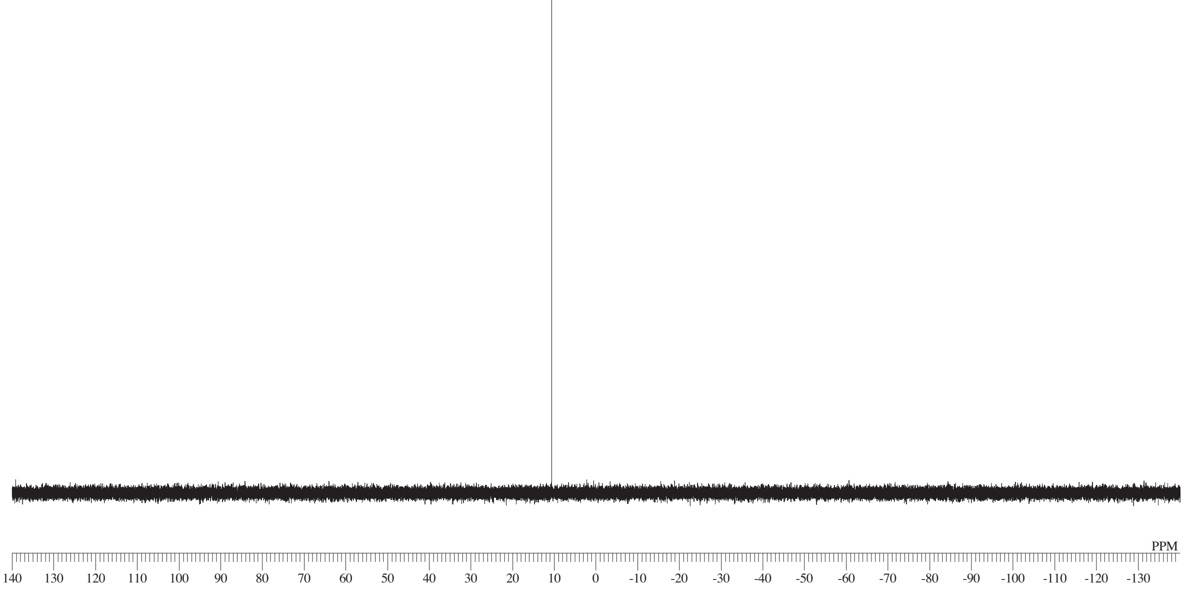

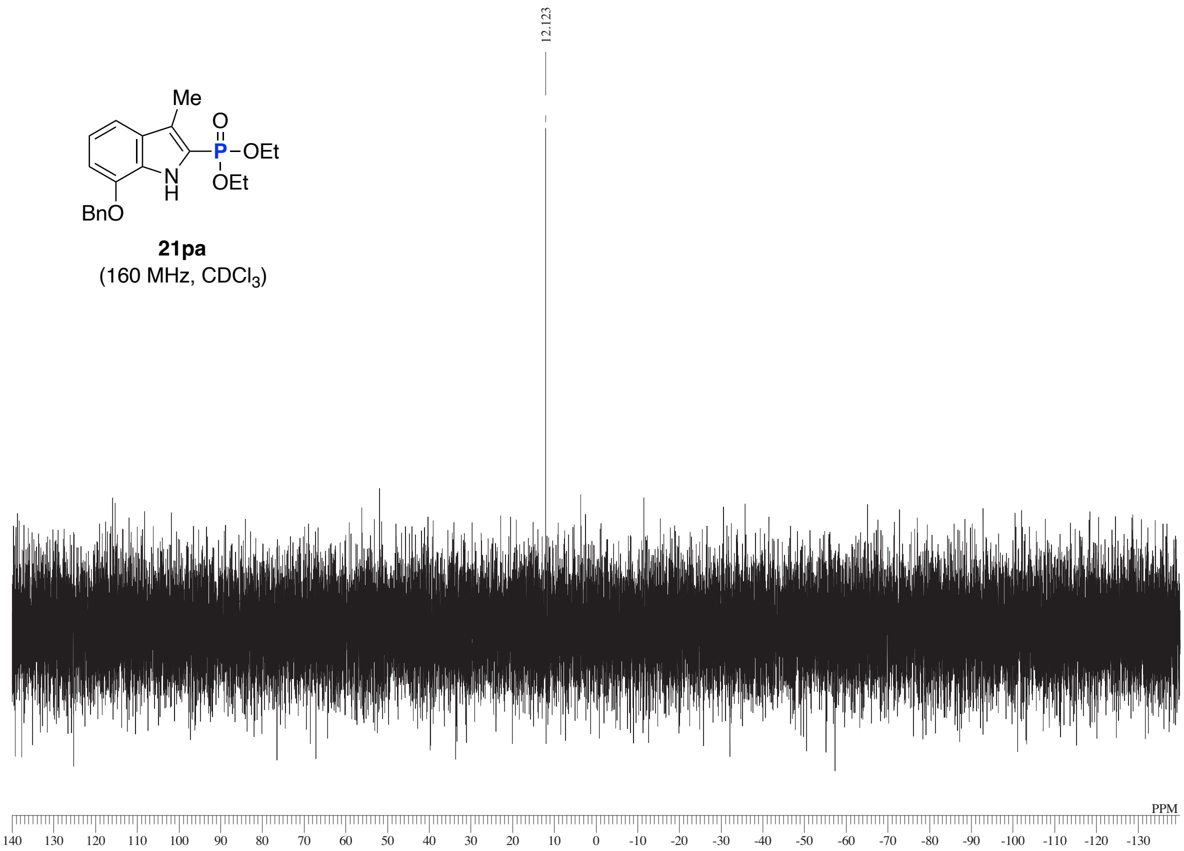

21pa
(160 MHz, $CDCl_3$)

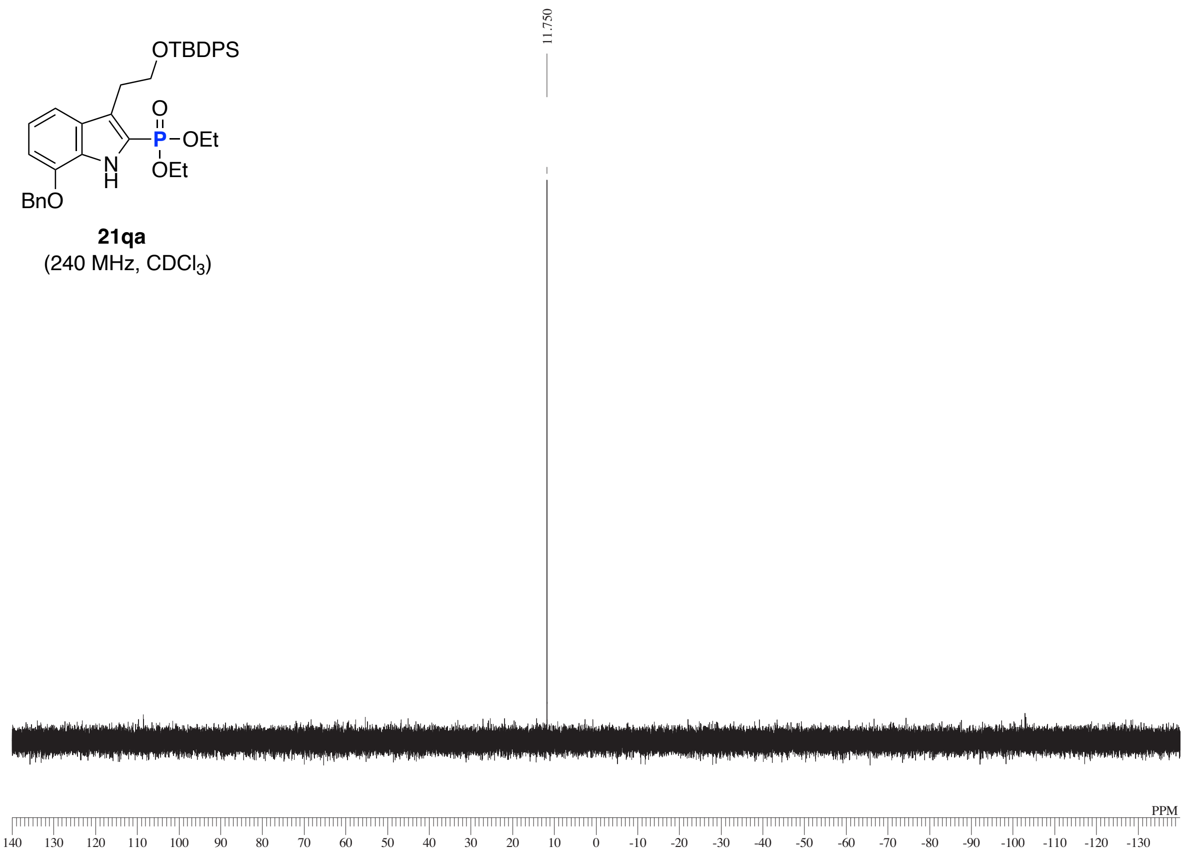

21qa
(240 MHz, $CDCl_3$)

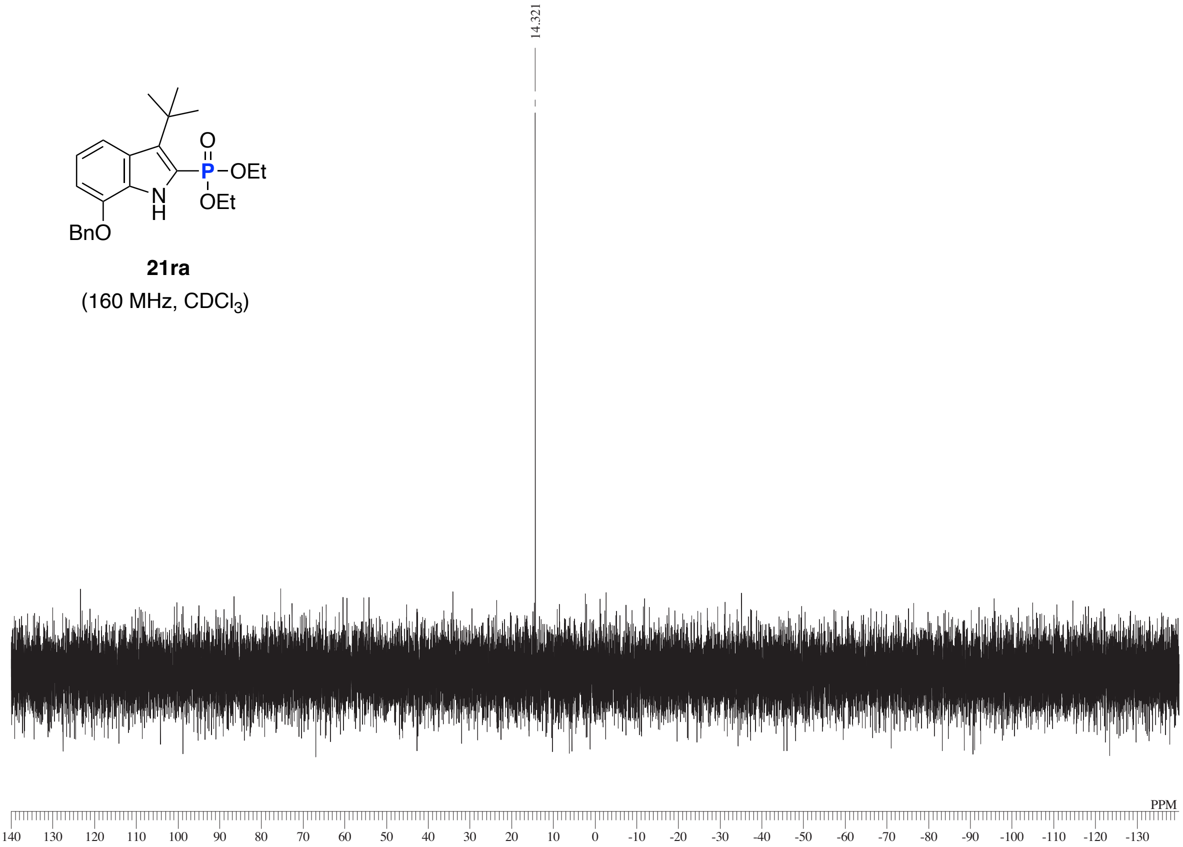

21ra

(160 MHz, $CDCl_3$)

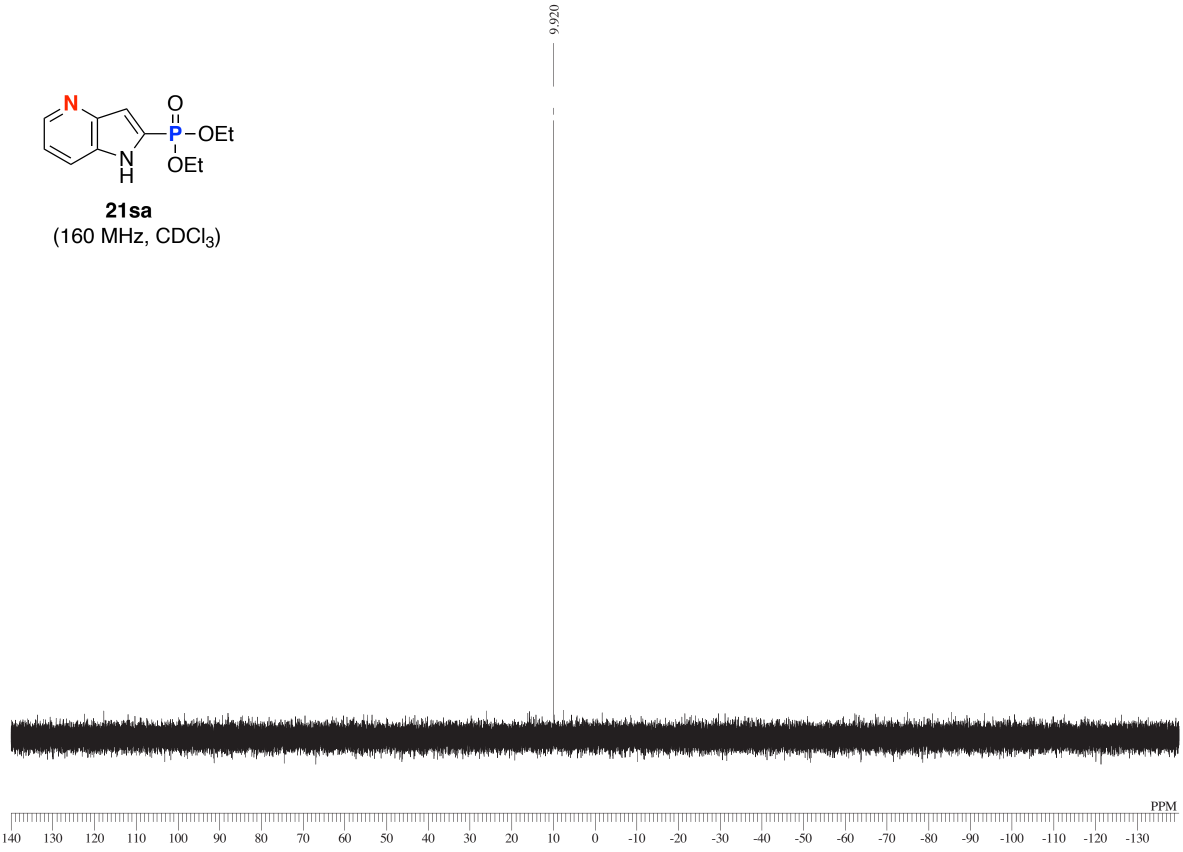

21sa

(160 MHz, $CDCl_3$)

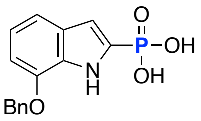

22
(160 MHz, DMSO)

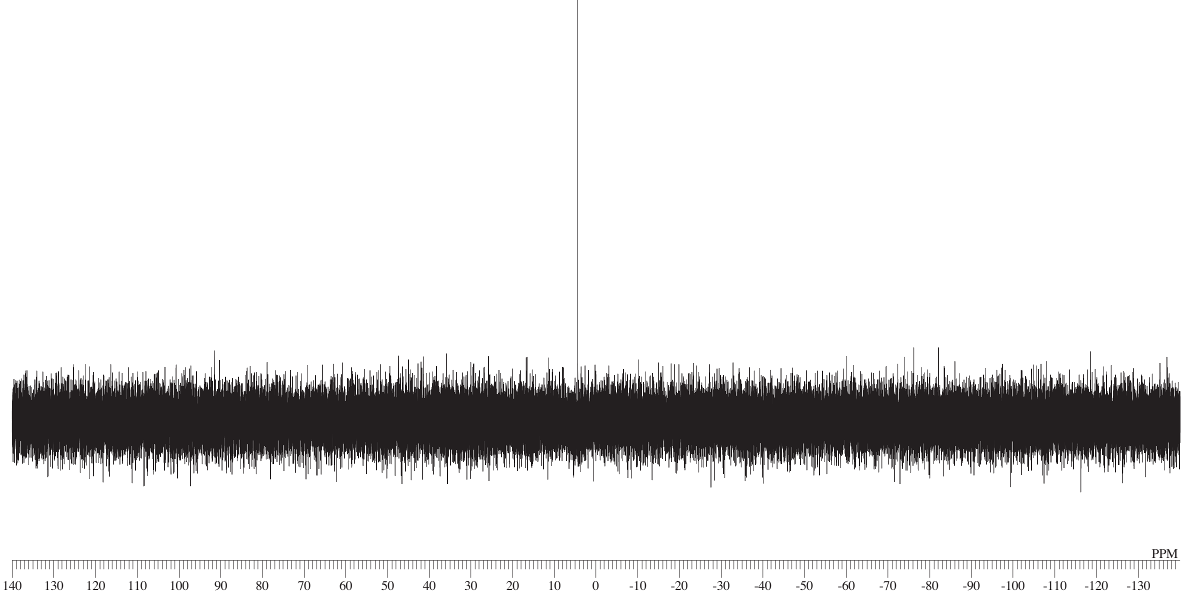

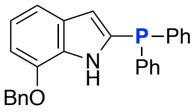

23
(160 MHz, $CDCl_3$)

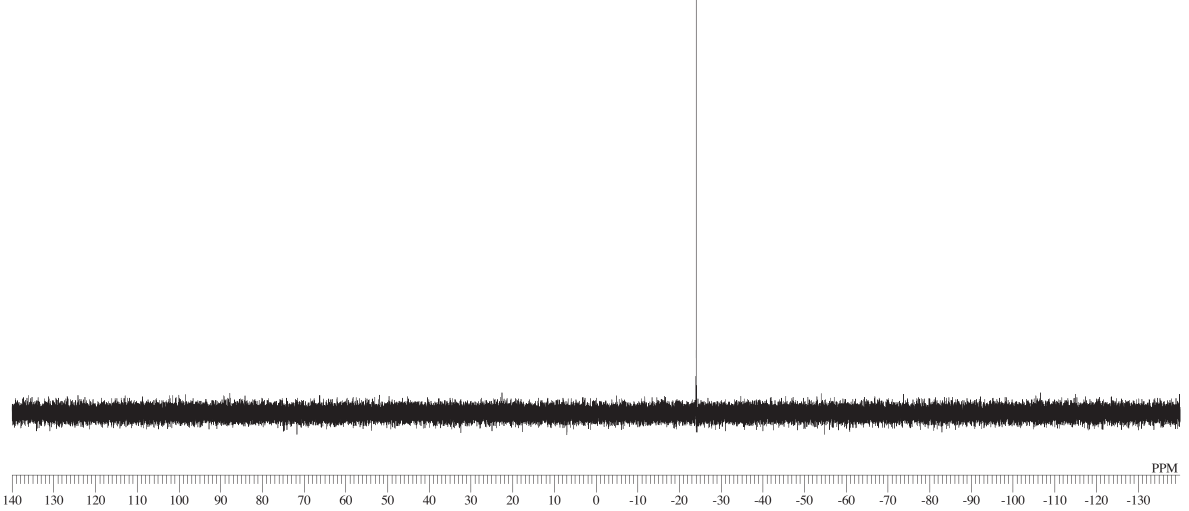

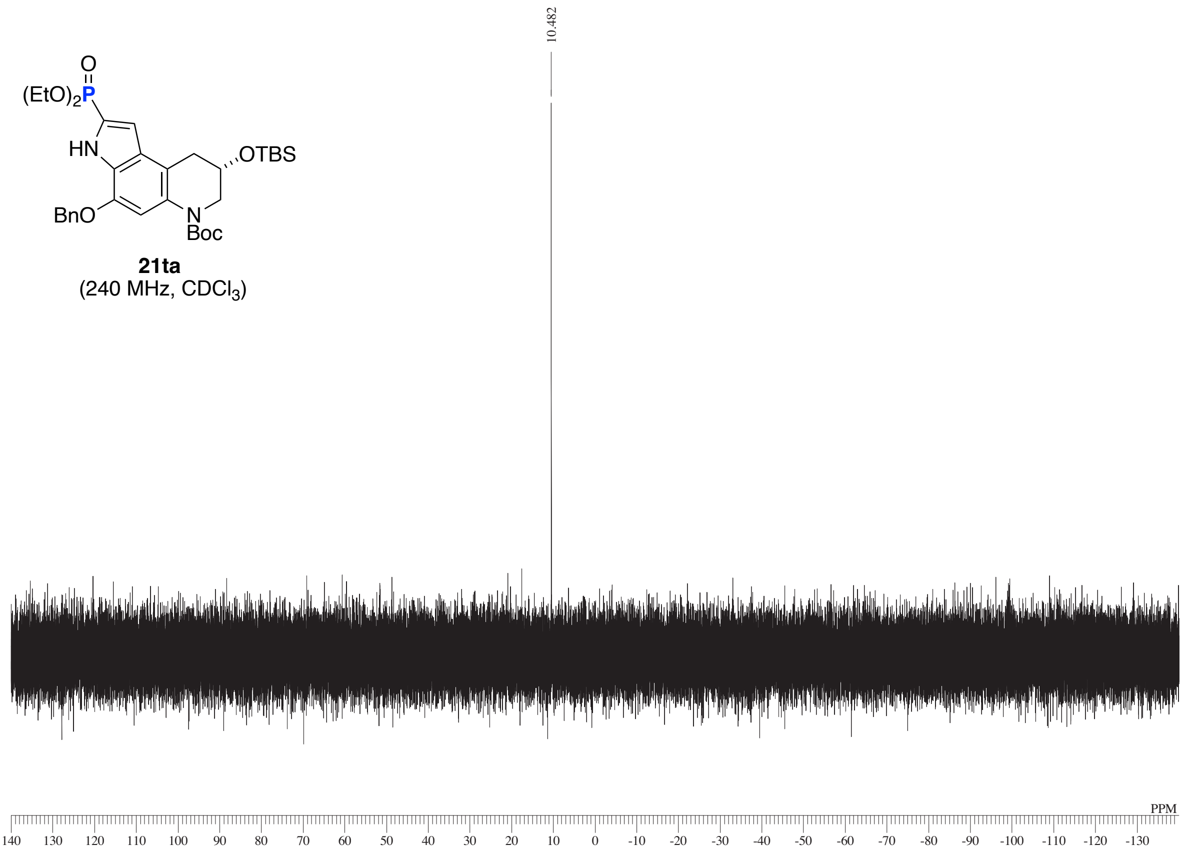

21ta
(240 MHz, $CDCl_3$)

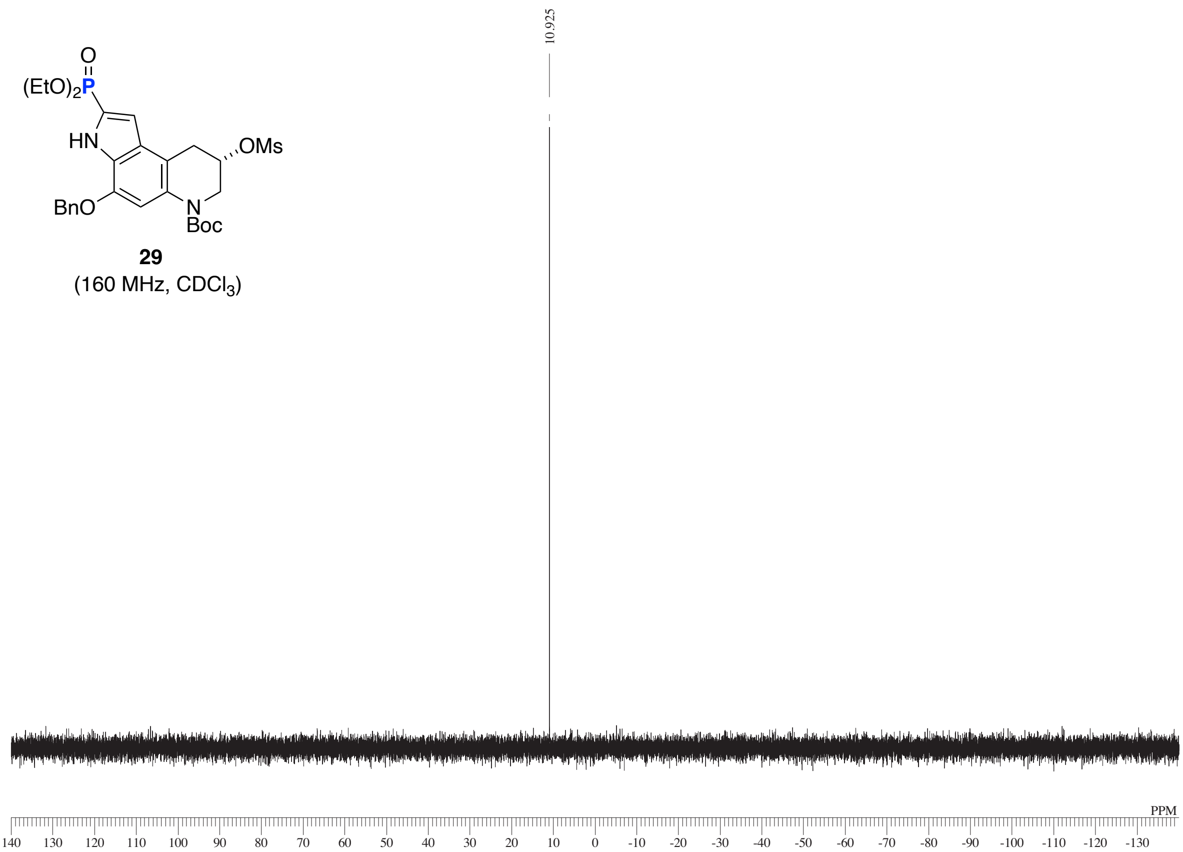

29
(160 MHz, $CDCl_3$)

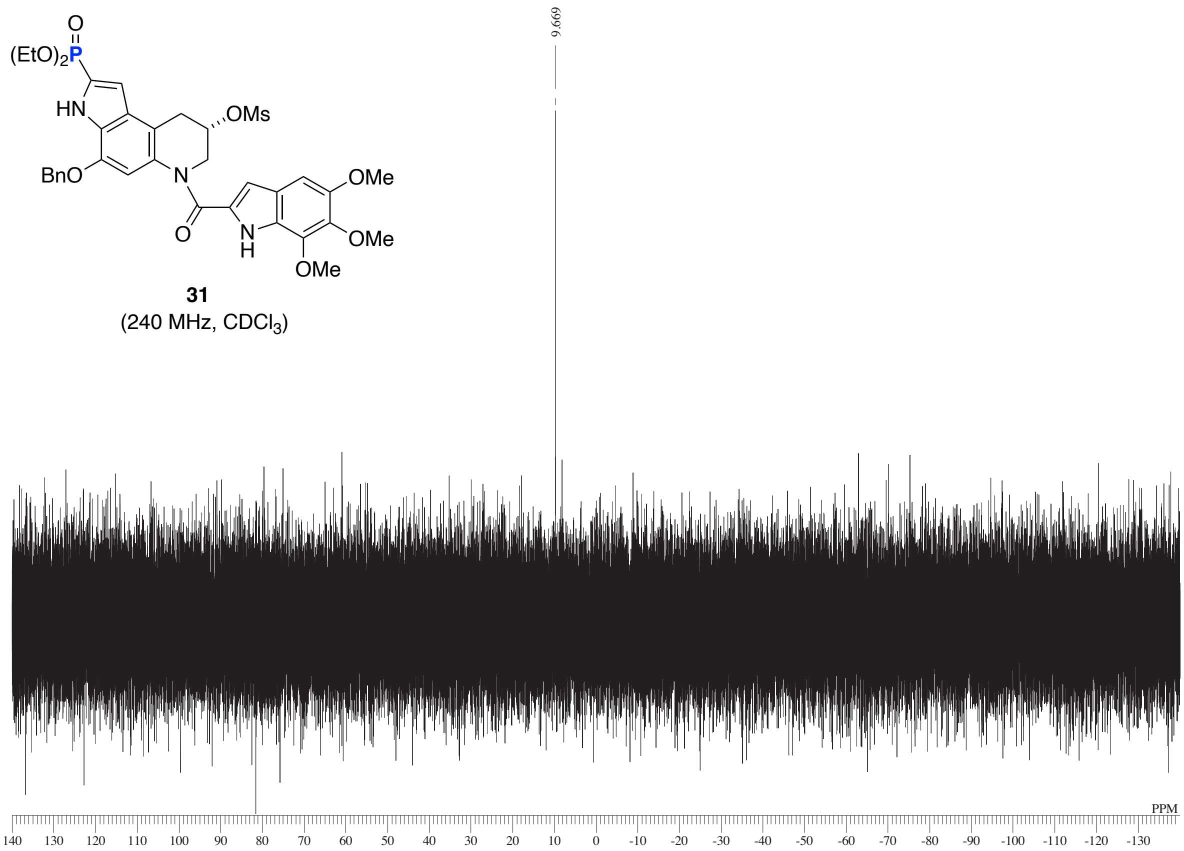

31
(240 MHz, $CDCl_3$)

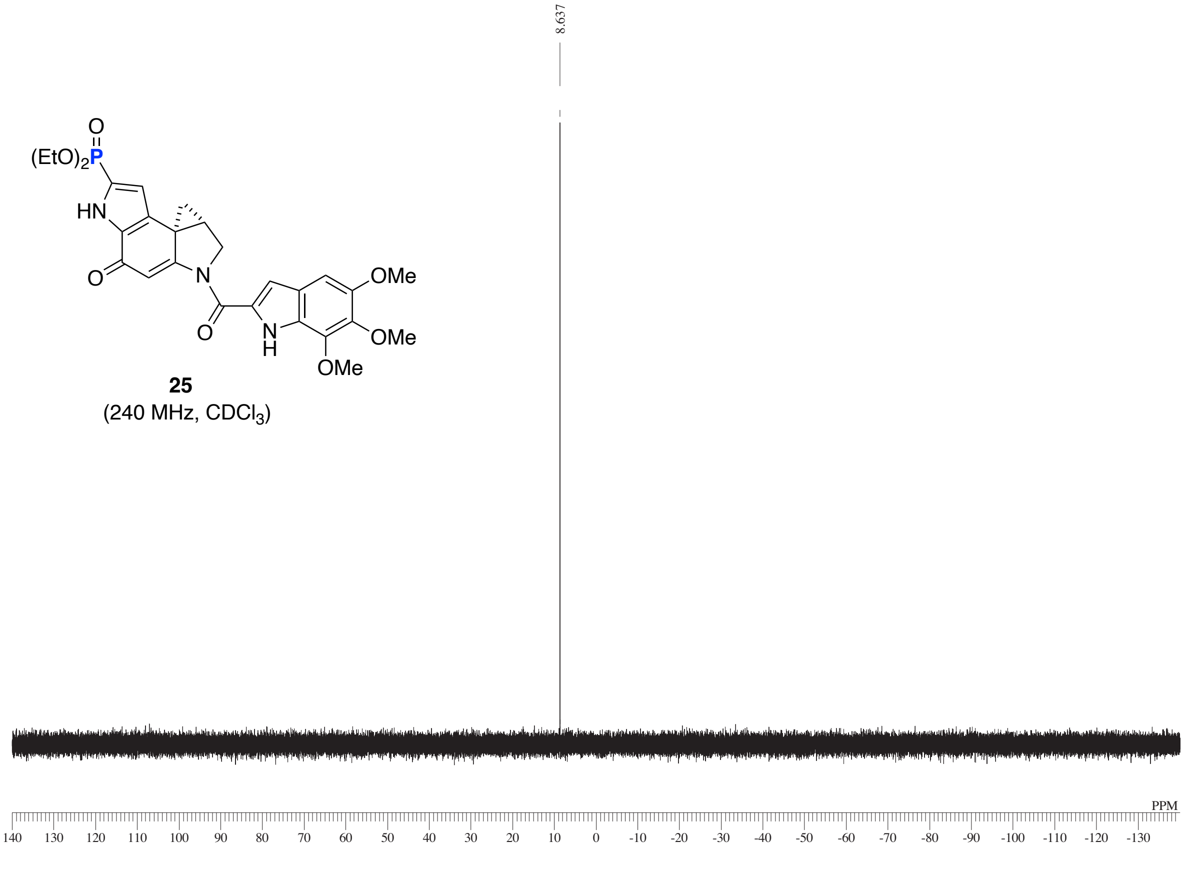

25
(240 MHz, $CDCl_3$)
